# Prediction models for COVID-19 disease outcomes

**DOI:** 10.1080/22221751.2024.2361791

**Published:** 2024-06-03

**Authors:** Cynthia Y. Tang, Cheng Gao, Kritika Prasai, Tao Li, Shreya Dash, Jane A. McElroy, Jun Hang, Xiu-Feng Wan

**Affiliations:** a Center for Influenza and Emerging Infectious Diseases, University of Missouri, Columbia, Missouri, USA; b Molecular Microbiology and Immunology, School of Medicine, University of Missouri, Columbia, Missouri, USA; c Bond Life Sciences Center, University of Missouri, Columbia, Missouri, USA; d Institute for Data Science and Informatics, University of Missouri, Columbia, Missouri, USA; e Department of Electrical Engineering & Computer Science, College of Engineering, University of Missouri, Columbia, Missouri, USA; f Viral Diseases Branch, Walter Reed Army Institute of Research, Silver Spring, Maryland, USA; g Family and Community Medicine, University of Missouri, Columbia, Missouri, USA

**Keywords:** Long COVID, machine learning, personalized medicine, predictive model for COVID-19, COVID-19 prediction, disease outcome prediction

## Abstract

SARS-CoV-2 has caused over 6.9 million deaths and continues to produce lasting health consequences. COVID-19 manifests broadly from no symptoms to death. In a retrospective cross-sectional study, we developed personalized risk assessment models that predict clinical outcomes for individuals with COVID-19 and inform targeted interventions. We sequenced viruses from SARS-CoV-2-positive nasopharyngeal swab samples between July 2020 and July 2022 from 4450 individuals in Missouri and retrieved associated disease courses, clinical history, and urban-rural classification. We integrated this data to develop machine learning-based predictive models to predict hospitalization, ICU admission, and long COVID.

The mean age was 38.3 years (standard deviation = 21.4) with 55.2% (*N* = 2453) females and 44.8% (*N* = 1994) males (not reported, *N* = 4). Our analyses revealed a comprehensive set of predictors for each outcome, encompassing human, environment, and virus genome-wide genetic markers. Immunosuppression, cardiovascular disease, older age, cardiac, gastrointestinal, and constitutional symptoms, rural residence, and specific amino acid substitutions were associated with hospitalization. ICU admission was associated with acute respiratory distress syndrome, ventilation, bacterial co-infection, rural residence, and non-wild type SARS-CoV-2 variants. Finally, long COVID was associated with hospital admission, ventilation, and female sex.

Overall, we developed risk assessment models that offer the capability to identify patients with COVID-19 necessitating enhanced monitoring or early interventions. Of importance, we demonstrate the value of including key elements of virus, host, and environmental factors to predict patient outcomes, serving as a valuable platform in the field of personalized medicine with the potential for adaptation to other infectious diseases.

## GRAPHICAL ABSTRACT

Model summary and motivation. Individuals infected with SARS-CoV-2 experience a wide spectrum of clinical manifestations ranging from no symptoms to death. Using the Virus-Human Outcomes Prediction (ViHOP) algorithm, we aim to utilize the individual’s clinical characteristics, the individual’s location, and the infecting SARS-CoV-2 virus characteristics obtained by whole genome sequencing to determine their likelihood of admission to the hospital, admission to the intensive care unit (ICU), or experiencing long COVID. This model allows clinicians to identify at-risk patients for further monitoring and/or early treatment.

**Figure:**
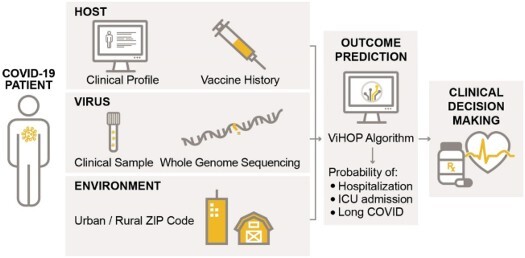

## Introduction

The coronavirus disease of 2019 (COVID-19), caused by the severe acute respiratory syndrome coronavirus 2 (SARS-CoV-2), has resulted in devastating global health impacts [1]. The Centers for Disease Control and Prevention (CDC) has reported a cumulative 6.2 million hospitalizations due to COVID-19 in the United States (US) alone [2]. While some individuals experience severe health consequences including death from COVID-19, others may exhibit mild to no symptoms [3, 4]. In addition to a wide array of acute clinical manifestations, long COVID has become a debilitating chronic complication for many, and approximately 7.5% of US adults have reported long-term sequelae from the infection at least 3 months after initial infection [5].

Early outpatient interventions, including transfusion of polyclonal convalescent plasma,[6] anti-SARS-CoV-2 monoclonal therapy, [7] Paxlovid, [8] remote monitoring, [9] and early administration of supplemental oxygen [10] have been shown to improve COVID-19 outcomes, indicating the value of identifying individuals who are at risk for poor outcomes. While many attempts have been made to characterize the disease severity of COVID-19, predicting clinical outcomes remains challenging due to the wide heterogeneity and complexity of host and virus factors. A variety of human factors [11–14] such as age, comorbidities, immune status, and urban-rural classification, [15–24] and virus factors [25, 26] including SARS-CoV-2 variants and mutations have been associated with outcomes. However, most studies analyze only host [27–29] or virus factors independently or incorporate limited human and virus combined data [30–33]. Further, while public databases provide a wealth of information and are often used, they suffer from inconsistencies in data collection, reporting, and breadth, limiting such analyses [34, 35]. Without the integration of host, virus, and environment factors, understanding how to most effectively predict patient outcomes will continue to remain incomplete.

We conducted a retrospective cohort study to curate an integrated dataset that uniquely incorporates the epidemiologic triad containing virus, human, and environmental data. Our primary objective was to utilize this unique dataset and develop machine learning algorithms that can predict an individual’s likelihood of hospitalization, intensive care unit (ICU) admission, and long COVID. From this study, we constructed the COVID-19 Virus-Human Outcomes Prediction (ViHOP) model, a personalized clinical risk assessment tool for predicting COVID-19 clinical outcomes. Overall, our models have the potential to assist clinicians in identifying high-risk COVID-19 patients, facilitating a more personalized approach for earlier interventions and monitoring.

## Methods

### Summary of study design

**Figure 1.**
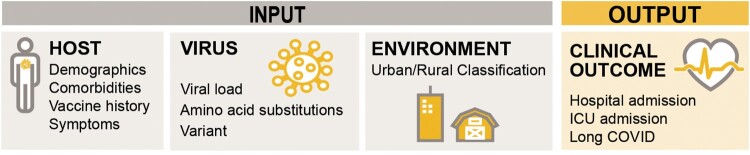
Features used for model training and selection. The model inputs included host, virus, and environment data. Host features were collected from electronic health records, virus features were collected using whole genome sequencing, and environmental features included the individual’s urban-rural classification based on their home ZIP Code. The model outcomes included hospital admission, ICU admission, long COVID, evaluated separately.

We conducted a retrospective human cohort study, using secondary SARS-CoV-2-positive nasopharyngeal swab samples and host information collected from electronic health records (EHR). SARS-CoV-2-positive specimens were collected from University of Missouri Health Care (UMHC) and CoxHealth between July 1, 2020, and July 31, 2022. Overall, 4471 total samples comprising 4,450 unique individuals were collected. We performed whole genome sequencing on these viruses to extract virus characteristics, which included cycle threshold (ct)-value, amino acid substitutions, and variant name, and chart reviews from the EHR to extract human characteristics, which included demographics, comorbidities, vaccination history, and disease course (Figure 1), environmental information (patient’s residential ZIP Code that was assigned a 4-level rural-urban commuting area (RUCA) classification: urban, rural as large, small and isolated town) [36]. The combination of these virus, human, and environment data was used to develop a machine learning model to predict three key primary outcomes of COVID-19: hospitalization, ICU admission, and long COVID (Figure 1). We use the CDC’s broad definition of long COVID as the continuation of signs, symptoms, and conditions at least four weeks after the initial COVID-19 infection [37].

### Ethical approval

This study has been reviewed by the University of Missouri Institutional Review Board (#2025449, #2049364).

### Problem formulation

Utilizing the combination of host, virus, and environmental data, we developed a machine learning model, the ViHOP algorithm, to predict clinical outcomes. We formulated this as a classification type of machine learning problem, where the task was to classify whether or not an individual with COVID-19 was going to become hospitalized, admitted to the ICU, or experience long COVID. We defined an individual experiencing long COVID as those with a follow-up appointment that reported sustained COVID-19 symptoms between 4-8 weeks after the nasopharyngeal swab date in consistency with the CDC’s broad definition of long COVID as the continuation of signs, symptoms, and conditions at least four weeks after the initial COVID-19 infection [37]. These outcomes were encoded as binary target variables where yes = 1 and no = 0. The models learned from training datasets (see Model Training and Validation) which included individuals who experienced these outcomes and those who did not. Predictor variables included host features such as demographics (age, sex, race, and ethnicity), comorbidities (pregnancy, immunosuppression, having any factors associated with metabolic syndrome (i.e. obesity, hypertension, diabetes, and hyperlipidaemia), cardiovascular disease, hepatic disease, chronic lung disease, or mental health disorder), vaccination (influenza or COVID-19 defined as vaccinated at least two weeks prior to swab collection), and COVID-19 symptoms and complications (i.e. ventilation or cardiopulmonary resuscitation required for management, sepsis, acute respiratory distress syndrome, or co-infection with another bacteria or virus during hospitalization), virus features including cycle threshold (ct)-values, variant, and amino acid substitutions, and environment features including urban-rural ZIP Code classification. These features are further detailed in Data Pre-Processing. Performance was evaluated using the area under the receiver operating characteristic (AUROC) score, which reflects how well the classifier distinguished between the positive and negative classes.

### Data collection

We collected clinical, virus, and environmental data in association with secondary SARS-CoV-2-positive nasopharyngeal swab samples.

#### Sample collection

SARS-CoV-2-positive nasopharyngeal swab samples were collected from University of Missouri Health Care (UMHC) and CoxHealth inpatient, ambulatory, and emergency department encounters between July 1, 2020, and July 31, 2022. Samples were tested for SARS-CoV-2 in clinical diagnostic laboratories using SARS-CoV-2 RNA, molecular tests, rapid antigen tests, and nucleic acid amplification tests. Sample collection targeted three swabs per ZIP Code per week for temporospatial representation based on availability. Inclusion criteria involved a positive SARS-CoV-2 diagnostic test and available nasopharyngeal swab sample. Overall, 4,471 total samples from 4,450 individuals were collected including 20 individuals with two or more swabs. For the 20 individuals who had more than one swab within 60 days, the first swab with sufficient RNA for genome sequencing was included in the analyses. One individual had two swabs during two separate infections, and each was analyzed as a separate encounter.

#### Clinical and environmental data

Clinical and environmental data were extracted by the University of Missouri NextGen Biomedical Informatics Center from the electronic health records (EHR) of individuals associated with the collected samples and manually validated and further supplemented by the study team. All clinical and environment data were captured in a secure REDCap (Research Electronic Data Capture) database.

#### Virus data

Virus genome data were collected by performing whole genome sequencing on the viruses extracted from the nasopharyngeal swabs. The Juno system (Fluidigm Corporation, CA, USA) was used with 47 pairs of custom-designed specific primers targeting the reference sequence, Wuhan-Hu-1 (Accession Number: NC_045512.2) and paired with real-time reverse transcription polymerase chain reaction test (RT–PCR) to amplify whole virus genomes [38]. Amplicon libraries were prepared using the Illumina DNA Prep kit, followed by sequencing with the MiSeq Reagent Kit v3 (600-cycle) and MiSeq sequencing system or the NovaSeq Reagent Kit SP v1.5 (300-cycle) on NovaSeq 6000 instrument (Illumina, San Diego, California, USA). Adapters were trimmed using the BBDuk software from the BBMap v.39.01 package [39]. A k-mer of 17 was used with a maximum Hamming distance of 1, and trimming was restricted to the 30 terminal bases. Trimmed sequences were then assembled using the Iterative Refinement Meta-Assembler (IRMA)[40] CoV module with a quality score of 20. The amended global alignments to the profile hidden Markov model (HMM) sequences (a2 m) were extracted as the consensus sequences, and sequences with a coverage breadth of ≥ 50% were included for subsequent analyses. Of the 4471 swabs collected, 2015 samples were adequately sequenced, representing 2014 individuals. The sequence quality data of whole genome sequencing for each sample are shown in Supplementary Table 1. Amino acid substitutions were extracted from the assembled consensus sequences using Nextclade v2.14.0 and SARS-CoV-2 reference dataset v2023-05-10T12:00:00Z [41]. Sequences determined by NextClade to be “mediocre” or “poor” quality frameshifts were re-assembled using CLC Genomics Workbench v22.0.1, where reads were trimmed with a quality limit of 0.5. After reassembly, sequences still determined by NextClade to be of “poor” quality frameshifts were excluded from analyses. Variant names as defined by the World Health Organization for each sequence were also extracted from NextClade, and variant frequencies detected among study samples are summarized in Supplementary Table 2. Ct-values were determined using reverse transcription-polymerase chain reaction.

### Data preprocessing and feature engineering

Datasets were preprocessed using feature encoding, imputation, and re-sampling.

#### Variables and feature encoding

Host features include demographics (age, sex, race, ethnicity, and urban-rural ZIP Code classification), comorbidities (pregnancy, immunosuppression, any factors associated with metabolic syndrome (i.e. obesity, hypertension, diabetes, and hyperlipidaemia), cardiovascular disease, hepatic disease, chronic lung disease, or mental health disorder), vaccination (influenza or COVID-19 defined as vaccinated at least two weeks prior to swab collection), and COVID-19 complications (i.e. ventilation or cardiopulmonary resuscitation required for management, sepsis, acute respiratory distress syndrome, or co-infection with another bacteria or virus during hospitalization). Individuals were considered partially vaccinated against COVID-19 if they began but did not complete the primary COVID-19 vaccine series, fully vaccinated if they completed the primary vaccine series, and boosted if they received additional shots after completing the primary COVID-19 vaccine series.

Urban-rural ZIP Code classifications were determined using secondary Rural-Urban Commuting Area (RUCA) codes defined by the Federal Office of Rural Health Policy (FORHP), U.S. Department of Agriculture’s Economic Research Service (ERS), and Washington, Wyoming, Alaska, Montana and Idaho (WWAMI) Rural Health Research Center [42]. Each United States ZIP Code is assigned one of 33 RUCA codes based on population density, levels of urbanization and journey-to-work commuting. The WWAMI Rural Health Research Center further aggregates these RUCA codes into four categories: urban, large rural, small rural town, and isolated small rural town [36]. Feature encoding strategies for each feature are detailed in Supplementary Table 3. Individuals who were hospitalized were screened for the reason for admission. Briefly, binary variables were left unchanged, age and ct-values were binned into ordinal categories through label encoding, and COVID-19 vaccinations and urban-rural classification, which we classified as ordinal categories were also encoded using label encoding. Individuals with COVID-19 reported as incidental findings were excluded from the analyses.

Virus features include amino acid substitutions throughout the consensus viral genome and viral load, represented by ct-value. Amino acid substitutions were identified and encoded as binary features (0 = absent, 1 = present). Missing or ambiguous bases were imputed as 0.5. Features and feature encoding are further defined and detailed in Supplementary Table 3. Further, the variant name of the virus was also used as a feature and analyzed separately from the amino acid substitutions to avoid collinearity.

We also sought to determine whether the co-occurrence of Spike gene substitutions may have affected outcomes and thus incorporated them by using pairwise, 1-norm, and 2-norm methods to generate additional datasets to evaluate optimal encoding of virus features. These feature combinations were analyzed as interaction terms and encoded as follows: Let *i* and *j* be two feature indices and $X_{i}$ and $X_{j}$ be their representing single features. We constructed the co-features of sites *i* and *j* by three strategies: pairwise product of vector $X_{i}$ and $X_{j}$, 1-norm of vector $X_{i}$ and $X_{j}$, and 2-norm of vector $X_{i}$ and $X_{j}$. For example, if the vector of each single feature is $X_{i}=(X_{i}^{1},\;X_{i}^{2},\cdots ,X_{i}^{K}{)}^{T}$ and $X_{j}=(X_{j}^{1},\;X_{j}^{2},\cdots ,X_{j}^{K}{)}^{T}$, then the corresponding pairwise product co-feature is represented as $X_{\mathrm{ij}}=(X_{i}^{1}\cdot X_{j}^{1},X_{i}^{2}\cdot X_{j}^{2},\cdots ,X_{i}^{K}\cdot X_{j}^{K}{)}^{T}$, the 1-norm co-feature is $X_{\mathrm{ij}}=(\mathrm{abs}(X_{i}^{1}+X_{j}^{1}),\mathrm{abs}(X_{i}^{2}+X_{j}^{2}),\cdots ,\mathrm{abs}(X_{i}^{K}+X_{j}^{K}){)}^{T}$, the 2-norm co-feature is $X_{\mathrm{ij}}=(\mathrm{sqrt}({\left(X_{i}^{1}\right)}^{2}+{\left(X_{j}^{1}\right)}^{2}),;\mathrm{sqrt}((X_{i}^{2}{)}^{2}+{\left(X_{j}^{2}\right)}^{2}),\cdots ,\mathrm{sqrt}({\left(X_{i}^{K}\right)}^{2}+{\left(X_{j}^{K}\right)}^{2}){)}^{T}$. For these co-features, we concentrated on residues that are in physical proximity under the assumption that these amino acids are more likely to interact and collaboratively influence the protein’s function. Our primary focus was on residues in the Spike protein. Feature interactions were determined based on the proximity of residues as mapped in the PDB 6VXX spike glycoprotein structure. Specifically, positions within 7 Angstroms (Å) of one another, denoting proximity,[43] were included as interaction terms in our model. At 7 Å, the following interaction residue pairs were identified: 212-213, 221-222, 307-308, 371-373, 373-375, 375-376, 405-408, 439-440, 439-498, 452-493, 452-494, 493-494, 494-496, 496-498, 498-501, 501-505, 653-655, 950-954, and 981-982. The interaction pairs were analyzed as separate features.

#### Datasets

Clinical features included in the hospital admission datasets were demographics, vaccination history, urban-rural classification, comorbidities, and symptoms. Clinical features included in the ICU admission datasets were demographics, vaccination history, urban-rural classification, comorbidities, symptoms, ventilation requirements, sepsis, acute respiratory distress syndrome, and the presence of a co-infection with another virus and bacteria. Finally, clinical features included in long COVID datasets were demographics, vaccination history, urban-rural classification, comorbidities, symptoms, admission to the hospital, and admission to the ICU. To account for potential biases in feature selection, variables with fewer than 5 events-per-variable were eliminated [44]. A total of seven datasets were generated and analyzed for each outcome: host-only, virus-only (amino acid substitutions), combined virus (amino acid substitutions) and host, combined virus (amino acid substitutions) and host with pairwise spike substitution pairs, combined virus (amino acid substitutions) and host with 1-norm spike substitution pairs, combined virus (amino acid substitutions) and host with 2-norm spike substitution pairs, and combined clinical and virus variant.

#### Missing data

Clinical data often suffer from missing data, and most robust machine learning models cannot handle missing data without additional processing. Filtering missing data introduces bias and decreases statistical power [45]. To account for missing data while preserving all data points, we employed a state-of-the-art Multivariate Imputation by Chained Equations (MICE) [46, 47] technique to impute clinical data using the assumption that the missing data were missing at random (MAR) [45]. Imputed variables include sex, race, ethnicity, urban-rural classification, flu vaccination, COVID-19 vaccination, pregnancy, immunosuppression, metabolic syndrome factors, liver disease, mental health disorder, chronic lung disease, and symptom categories (respiratory, gastrointestinal, cardiac, and constitutional). Factors with minimal missing data, including age, sex, race, ethnicity, urban-rural classification, payer, flu vaccination, COVID-19 vaccination, pregnancy, immunosuppression, metabolic syndrome factors, liver disease, mental health disorder, chronic lung disease, and symptom categories (respiratory, gastrointestinal, cardiac, constitutional) were used to impute those variables. Ct-values were imputed using the average value for each lineage. Specific imputation techniques for each feature are further detailed in Supplementary Table 3. Imputation was performed on RStudio 2023.06.2 + 561 with R v4.2.3.

#### Balancing the datasets

Due to the low number of individuals experiencing poor outcomes (hospitalized, admitted to the ICU, or long COVID) compared with those who did not, we evaluated the effectiveness of re-sampling techniques on the training data [48] to minimize the impacts of class imbalance: [49, 50]. No resampling, random oversampling, and Synthetic Minority Oversampling TEchnique (SMOTE) [51]. Re-sampling was performed using imblearn v0.11.0 in Python v3.7.10.

### Feature selection

Each model was tested with and without feature selection. Feature selection is a strategy for dimensionality reduction and involves selecting the most significant predictors to construct a model. The Least Absolute Shrinkage and Selection Operator (Lasso) is a popular feature selection technique because it can select features while also imposing a penalty on the regression coefficients to regularize the model and mitigate overfitting [52]. Lasso accomplishes this by shrinking coefficients towards zero, and the corresponding predictors are then removed from the model, enabling the identification of significant predictors for each model.

In our pipeline, we trained our models with and without feature selection. We used the Lasso class from the Scikit-learn v 1.2.1 linear_model module in Python v 3.10.9. We defined a range of alpha values (0.001, 0.01, 0.1, 1.0) to test different regularization strengths and performed hyperparameter tuning over these alpha values using GridSearchCV with a 10-fold cross-validation. Model performance was assessed using the area under the Receiver Operating Characteristic (AUROC) curve, then fitted to the training data. The AUROC score ranges from 0 to 1, with a higher AUROC score signifying better model performance. Predictors with non-zero coefficients from the best-performing LASSO estimator were subsequently used for model selection described in the next section. The same features were extracted from the testing dataset for model evaluation described in later sections.

For the best-performing model that did not employ feature selection using Lasso (hospitalization), feature importance was analyzed using SHapley Additive exPlanations (ShAP) values and visualized with a beeswarm plot. To identify the most predictive features for each outcome, we assessed feature importance with ShAP,[53] which quantifies each feature’s contribution to a model’s performance. In the context of our models, a positive ShAP value indicates a positive prediction (i.e. presence of the tested outcome), whereas a negative ShAP value denotes a negative prediction (i.e. absence of the outcome). The magnitude of the ShAP value signifies the impact of the feature on the prediction. We computed and visualized feature importance using Python v3.7.10 and the shap library v0.42.1.

### Model selection

We evaluated seven commonly used machine learning classification models for predicting binary clinical outcomes. Mathematically, we aimed to identify a function Y=f(X:Θ), where Y represents the discrete outcomes for one of three clinical outcomes, X is the input feature data, and Θrepresents the model parameters.

#### Model selection

The tested classifiers included Random Forest, Gradient Boosting, Logistic Regression (including LASSO and Ridge Regression), Naïve Bayes (Gaussian), Decision Tree, Neural Network (Multi-layer Perceptron (MLP)), K-Nearest Neighbors, Support Vector, and Extra Trees. The algorithms were selected based on their demonstrated utility in healthcare prediction models [54–57] and evaluated using Scikit-Learn [58]. Random Forest, which has been commonly used for predicting hospitalizations [59] and ICU admission [60–62], is a decision tree-based model that can run multiple parallel trees. This technique averages predictions of all trees generated, allowing for a reduction of overfitting [63]. Simple decision trees and extra trees were also tested [64]. Gradient Boosting, which has been shown to be effective for predicting ICU admission in the emergency department [65], utilizes multiple weak prediction models, typically decision trees, and combines them to generate a more robust model [66]. Logistic regression predicts the dependent categorical variable as a function of the independent variables. Regularization techniques for logistic regression such as Lasso and Ridge regressions can help with overfitting and were also tested [67, 68]. Lasso in particular has been used by multiple groups for predicting the disease severity of hospitalized patients [69, 70]. Ridge regression has been used to predict hospitalization outcomes for COVID-19 [71] among other clinical applications. Naïve Bayes (Gaussian) utilizes the Bayes theorem with the “naïve” assumption of feature independence [72]. Support vector classification [73] identifies a hyperplane that optimizes the separation of classes in a feature space and can handle high-dimensional data and manage overfitting. K-Nearest Neighbors [74] (KNN) classifies a data point based on the majority class among its nearest neighbours in a feature space. Finally, multi-layer Perceptron (MLP) is a neural network-based technique that forms multiple layers of interconnected neurones to learn complex relationships and can learn non-linear models [75].

#### Parameter tuning

We tuned hyperparameters using GridSearchCV in sklearn with 10-fold cross-validation, allowing us to test all parameter combinations in a predefined parameter grid (Supplementary Table 4) [58]. We adapted parameter grids in consistency with Subudhi et al. [65].

### Model training and evaluation

Each of the datasets was split into stratified (for balanced output classes) and shuffled 80% training and 20% testing datasets. After feature selection and resampling of the training data, we evaluated the machine learning classifiers described in the preceding section to identify and optimize the best models for predicting each outcome. Model development and evaluation were performed using Python v3.7.10, numpy v1.21.6, pandas v1.3.5, and sklearn v1.0.2 [58]. Visualizations for AUROCs were generated using matplotlib v3.3.3.

#### Model training

Each model was subjected to a grid search over a range of hyperparameters listed in Supplementary Table 4 using GridSearchCV with 10-fold cross-validation and optimized for the AUROC. To evaluate the performance of the optimized model, we used the predictors from the test dataset to predict the outcomes and compare them with the target variables.

#### Model evaluation

The primary evaluation metric used to select the best performing model was the Area Under the Receiver Operating Characteristic (AUROC) score,[76] which measures a model’s ability to distinguish between classes with an AUROC score of 0.5 suggesting no discrimination ability, 0.7-0.8 is considered acceptable, 0.8-0.9 is excellent, and >0.9 as outstanding discrimination ability. Additional performance metrics included accuracy, sensitivity/recall, specificity, precision, and F1-score were calculated. Performance scores for each combination of models are reported in Supplementary Table 5 for hospitalization, Supplementary Table 6 for ICU admission, and Supplementary Table 7 for long COVID. The best-performing model was selected based on the model with the highest AUROC score and specificity and sensitivity > 0.6.

### Statistical analysis

To improve the interpretability of our model, we performed multivariable logistic regression analyses for each outcome by examining features selected from the best-performing model using the “sm” module from the Python statsmodels.api package and visualized as forest plots described below. The odds ratios (OR) and adjusted odds ratios (aOR) were calculated with 95% confidence intervals (CI). Significance was defined as *p*-value < 0.05 and 95% confidence intervals that do not include the value of 1. Subsequent forest plots were constructed using the “plt” module from the Python matplotlib.pyplot package.

## Results

### Study population

During the study period between July 2020 and July 2022, we collected 4,471 COVID-19-positive nasopharyngeal specimens representing 4,450 individuals. The characteristics of our study population are summarized in Table 1. The average age of our study population was 38.3 years (standard deviation [SD] = 21.4). Females represented 55.2% (2,453 individuals). The vast majority (*N* = 3,830, 88.6%) of our study population was White, with 350 (8.1%) Black, and 43 (1%) Asian, representative of the statewide demographics [77]. Only 454 (17.9%) individuals had completed the primary COVID-19 vaccine series prior to their swab date. Individuals living in urban areas comprised 63.0% (2,782) of the study population, those in large rural towns comprised 17.5% (771), small rural towns comprised 10.4% (458), and isolated small rural towns comprised 9.2% (407).

**Table 1.** Study population.

	Hospitalization & ICU				Long COVID-19		
	^#^Hospitalized (*N* = 223)	*ICU (N = 100)	Not Hospitalized (*N* = 4228)	Total (*N* = 4451)	No Long COVID (*N* = 3548)	Long COVID (*N* = 175)	Total (*N* = 3723)
**Age, years**							
Mean (SD)	57.777 (21.159)	58.350 (18.437)	37.048 (20.757)	38.301 (21.359)	38.315 (21.564)	50.091 (20.560)	38.869 (21.659)
Range	0.000 - 100.000	0.000 - 88.000	0.000 - 102.000	0.000 - 102.000	0.000 - 102.000	0.000 - 87.000	0.000 - 102.000
**Sex**							
Number missing	0	0	4	4	3	0	3
Female	142 (52.8%)	55 (55.0%)	2311 (55.3%)	2453 (55.2%)	1949 (55.0%)	109 (62.3%)	2058 (55.3%)
Male	127 (47.2%)	45 (45.0%)	1867 (44.7%)	1994 (44.8%)	1596 (45.0%)	66 (37.7%)	1662 (44.7%)
**Race**							
Number missing	6	2	122	128	94	1	95
American Indian/Alaskan Native	0 (0.0%)	0 (0.0%)	4 (0.1%)	4 (0.1%)	2 (0.1%)	0 (0.0%)	2 (0.1%)
Asian	0 (0.0%)	0 (0.0%)	43 (1.1%)	43 (1.0%)	30 (0.9%)	1 (0.6%)	31 (0.9%)
Black	20 (7.6%)	8 (8.2%)	330 (8.1%)	350 (8.1%)	286 (8.3%)	9 (5.2%)	295 (8.1%)
Native Hawaiian/Pacific Islander	0 (0.0%)	0 (0.0%)	8 (0.2%)	8 (0.2%)	6 (0.2%)	0 (0.0%)	6 (0.2%)
Other	3 (1.1%)	1 (1.0%)	85 (2.1%)	88 (2.0%)	62 (1.8%)	2 (1.1%)	64 (1.8%)
White	240 (91.3%)	89 (90.8%)	3590 (88.4%)	3830 (88.6%)	3068 (88.8%)	162 (93.1%)	3230 (89.0%)
**Ethnicity**							
Number missing	1	0	48	49	33	0	33
Hispanic	6 (2.2%)	0 (0.0%)	126 (3.0%)	132 (3.0%)	98 (2.8%)	2 (1.1%)	100 (2.7%)
Not Hispanic	262 (97.8%)	100 (100.0%)	4008 (97.0%)	4270 (97.0%)	3417 (97.2%)	173 (98.9%)	3590 (97.3%)
**Urban-Rural Classification**							
Number missing	1	0	32	33	22	0	22
Isolated	41 (15.3%)	22 (22.0%)	366 (8.8%)	407 (9.2%)	331 (9.4%)	15 (8.6%)	346 (9.3%)
Large rural	61 (22.8%)	28 (28.0%)	710 (17.1%)	771 (17.5%)	619 (17.6%)	30 (17.1%)	649 (17.5%)
Small rural	45 (16.8%)	15 (15.0%)	413 (10.0%)	458 (10.4%)	381 (10.8%)	24 (13.7%)	405 (10.9%)
Urban	121 (45.1%)	35 (35.0%)	2661 (64.1%)	2782 (63.0%)	2195 (62.3%)	106 (60.6%)	2301 (62.2%)
**Received Influenza Vaccine**							
Number missing	164	66	2666	2830	2255	74	2329
Vaccinated	65 (61.9%)	22 (64.7%)	993 (65.5%)	1058 (65.3%)	835 (64.6%)	67 (66.3%)	902 (64.7%)
Unvaccinated	40 (38.1%)	12 (35.3%)	523 (34.5%)	563 (34.7%)	458 (35.4%)	34 (33.7%)	492 (35.3%)
**Received COVID-19 Vaccine**							
Number missing	107	56	1807	1914	1527	49	1576
Boosted	7 (4.3%)	2 (4.5%)	166 (7.0%)	173 (6.8%)	127 (6.3%)	7 (5.6%)	134 (6.2%)
Full	25 (15.4%)	12 (27.3%)	429 (18.1%)	454 (17.9%)	334 (16.5%)	20 (15.9%)	354 (16.5%)
None	124 (76.5%)	29 (65.9%)	1691 (71.2%)	1815 (71.5%)	1488 (73.6%)	96 (76.2%)	1584 (73.8%)
Partial	6 (3.7%)	1 (2.3%)	89 (3.7%)	95 (3.7%)	72 (3.6%)	3 (2.4%)	75 (3.5%)
**Pregnant**							
Yes	5 (1.9%)	2 (2.0%)	72 (1.7%)	77 (1.7%)	65 (1.8%)	3 (1.7%)	68 (1.8%)
No	264 (98.1%)	98 (98.0%)	4110 (98.3%)	4374 (98.3%)	3483 (98.2%)	172 (98.3%)	3655 (98.2%)
**Immunosuppression**							
Number missing	6	1	489	495	331	3	334
Yes	48 (18.3%)	31 (31.3%)	97 (2.6%)	145 (3.7%)	118 (3.7%)	18 (10.5%)	136 (4.0%)
No	215 (81.7%)	68 (68.7%)	3596 (97.4%)	3811 (96.3%)	3099 (96.3%)	154 (89.5%)	3253 (96.0%)
**Number of Metabolic Syndrome Factors**							
Number missing	6	1	489	495	331	3	334
0	52 (19.8%)	16 (16.2%)	2244 (60.8%)	2296 (58.0%)	1790 (55.6%)	48 (27.9%)	1838 (54.2%)
1	78 (29.7%)	32 (32.3%)	796 (21.6%)	874 (22.1%)	742 (23.1%)	49 (28.5%)	791 (23.3%)
2	69 (26.2%)	26 (26.3%)	365 (9.9%)	434 (11.0%)	380 (11.8%)	36 (20.9%)	416 (12.3%)
3	41 (15.6%)	15 (15.2%)	196 (5.3%)	237 (6.0%)	203 (6.3%)	27 (15.7%)	230 (6.8%)
4	23 (8.7%)	10 (10.1%)	92 (2.5%)	115 (2.9%)	102 (3.2%)	12 (7.0%)	114 (3.4%)
**Cardiovascular Disease**							
Number missing	6	1	489	495	331	3	334
Yes	96 (36.5%)	35 (35.4%)	296 (8.0%)	392 (9.9%)	339 (10.5%)	37 (21.5%)	376 (11.1%)
No	167 (63.5%)	64 (64.6%)	3397 (92.0%)	3564 (90.1%)	2878 (89.5%)	135 (78.5%)	3013 (88.9%)
**Liver Disease**							
Number missing	6	1	489	495	331	3	334
Yes	15 (5.7%)	5 (5.1%)	68 (1.8%)	83 (2.1%)	72 (2.2%)	8 (4.7%)	80 (2.4%)
No	248 (94.3%)	94 (94.9%)	3625 (98.2%)	3873 (97.9%)	3145 (97.8%)	164 (95.3%)	3309 (97.6%)
**Chronic Lung Disease**							
Number missing	6	1	489	495	331	3	334
Yes	63 (24.0%)	22 (22.2%)	397 (10.8%)	460 (11.6%)	398 (12.4%)	40 (23.3%)	438 (12.9%)
No	200 (76.0%)	77 (77.8%)	3296 (89.2%)	3496 (88.4%)	2819 (87.6%)	132 (76.7%)	2951 (87.1%)
**Mental Health Disorder**							
Number missing	6	1	489	495	331	3	334
Yes	72 (27.4%)	24 (24.2%)	915 (24.8%)	987 (24.9%)	869 (27.0%)	70 (40.7%)	939 (27.7%)
No	191 (72.6%)	75 (75.8%)	2778 (75.2%)	2969 (75.1%)	2348 (73.0%)	102 (59.3%)	2450 (72.3%)
**Hospitalization**							
Hospitalized					178 (5.0%)	45 (25.7%)	223 (6.0%)
Not Hospitalized					3370 (95.0%)	130 (74.3%)	3500 (94.0%)
**ICU**							
ICU					88 (2.5%)	12 (6.9%)	100 (2.7%)
Not admitted to ICU					3460 (97.5%)	163 (93.1%)	3623 (97.3%)

*ICU (intensive care unit) admissions are a subset of hospitalized individuals. ^#^Of the 223 individuals hospitalized due to COVID-19, the average time from our collected swab sample to hospitalization was 5.3 days (SD = 3.4) for the 102 individuals hospitalized after the initial swab (98 were collected at hospital admission, 23 were collected after hospital admission), and the average time of hospitalization was 10.1 days (SD = 26.2).

Between July 15, 2020–July 31, 2022, Missouri reported a total of 1,054,060 hospitalizations and 241,979 ICU admissions with hospital encounters peaking in December 2020, August 2021, and January 2022 (Supplementary Figure 1A) [78]. Our study population followed the statewide trends; 223 individuals were hospitalized and 100 were further admitted to the ICU (Supplementary Figure 1A). The predominant variants detected among sequences submitted to GISAID [79, 80] and among our study samples were wild type, Alpha, Delta, and Omicron, and the peaks of each variant among our study samples were likewise consistent with those in the public database (Supplementary Figure 1B, Supplementary Table 2), highlighting the representative and generalizable nature of our dataset. Overall, our study population represents individuals from two hospital systems in Missouri with disease and virus characteristics that reflect statewide trends, allowing us to use this study population to generate generalizable predictive models.

### ViHOP model and risk factors associated with COVID clinical outcomes

**Figure 2.**
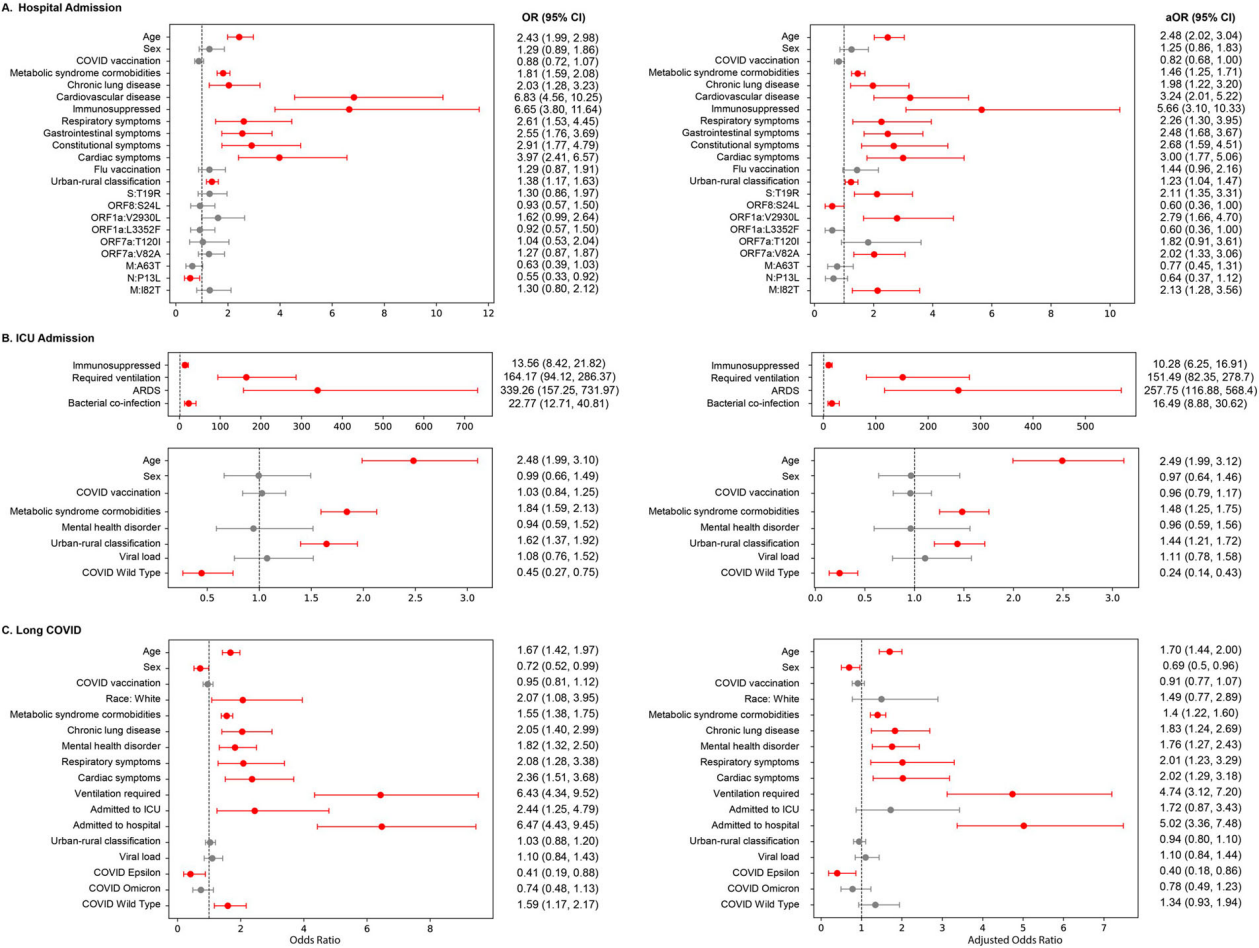
Statistical Analyses of Predictors. Evaluation of statistical associations between the selected predictive features and (A) hospitalization, (B) ICU admission, and (C) long COVID. Each dot represents the odds ratio, and each line represents the 95% confidence interval (CI) for each feature. Statistically significant associations are highlighted in red and defined as a 95% CI that does not overlap 1 (illustrated by the vertical dotted line). The left panel illustrates unadjusted odds ratios, and the right panel illustrates odds ratios adjusted for age, sex, and COVID-19 vaccination. ARDS, acute respiratory distress syndrome.

Using this representative study population, we identified 4450 individuals with clinical data, 2128 individuals with sufficient virus sequencing for hospitalization and ICU admission prediction, and 1798 individuals who had sufficient genome data to predict long COVID. We applied ViHOP to these datasets and obtained AUROC scores of 0.87, 0.98, and 0.79 for hospitalization, ICU admission, and long COVID outcomes, respectively. Detailed performance metrics for these models are presented in Table 2, and statistical analyses of each selected feature are represented in Figure 2. Additional details for all tested models are available in Supplementary Figure 2 and Supplementary Tables 5–7.

**Table 2.** Best performing model metrics.

Outcome	Hospitalization	ICU Admission	Long COVID
Optimal dataset	Combined host, environment, and virus substitutions (1norm)	Combined host, environment, and virus variant	Combined host, environment, and virus variant
Sample sizes in optimal dataset	121 hospitalized, 1983 not hospitalized	46 admitted to ICU, 2,058 not admitted to ICU	168 with long COVID, 3,406 total
Oversampling technique	Random	None	Random
Training/Testing Split*	80%/20%	80%/20%	80%/20%
Feature selection	No	Yes	Yes
Algorithm	SVC	Naïve Bayes	Ridge Regression
Best Model (Hyperparameters)	SVC(C = 1, coef0 = 10.0, probability = True)	GaussianNB()	LogisticRegression(C = 1.5, max_iter = 10000, solver = “saga”)
AUROC	0.87	0.98	0.79
Accuracy	0.86	0.95	0.71
Sensitivity	0.7	0.91	0.71
Specificity	0.87	0.95	0.71
Precision	0.28	0.34	0.09
Recall	0.7	0.91	0.71
F1	0.4	0.5	0.16
F1 Macro	0.66	0.74	0.49

AUROC, area under the receiver operating curve. Performance metrics for all other models are detailed in Supplementary Tables 5–7. *Datasets were stratified for class balance and shuffled for randomness for an 80/20 train/test split. Predictor variables included host features such as demographics (age, sex, race, and ethnicity), comorbidities (pregnancy, immunosuppression, factors associated with metabolic syndrome (i.e. obesity, hypertension, diabetes, and hyperlipidaemia), cardiovascular disease, hepatic disease, chronic lung disease, or mental health disorder), vaccination (influenza or COVID-19 defined as vaccinated at least two weeks prior to swab collection), and COVID-19 symptoms and complications (i.e. ventilation or cardiopulmonary resuscitation required for management, sepsis, acute respiratory distress syndrome, or co-infection with another bacteria or virus during hospitalization), virus features including cycle threshold (ct)-values, variant, and amino acid substitutions, and environment features including urban-rural ZIP Code classification. Additional descriptions and feature encoding details are available in Supplementary Table 3.

Our first objective was to construct the best model to predict hospitalization due to COVID-19 for an individual infected with SARS-CoV-2. The best-performing algorithm was a Support Vector Classifier (SVC) trained on the combined host, virus substitutions (normalized using 1-norm, see Methods), and environment dataset after random oversampling, which performed with an AUROC score of 0.87 (Table 2). A set of clinical factors were identified to be positively associated with hospitalization, including immunosuppression (aOR = 5.66; a 95% CI [3.10, 10.33]), cardiovascular disease (3.24; [2.01, 5.22]), older age (2.48; [2.02, 3.04]), and the presence of cardiac (3.00; [1.77, 5.06]), gastrointestinal (2.48; [1.68, 3.67]), and constitutional (2.68; [1.59 to 4.51]) symptoms (Figure 2(A)). Rural residence (based on ZIP Code) (1.23; [1.04, 1.47]), was positively associated with hospitalization, compared to urban residence. Genetic factors such as amino acid substitutions membrane (M):I82 T (2.13; [1.28, 3.56]), spike (S):T19R (2.11; [1.35, 3.31]), open reading frame(ORF)1a:V2930L (2.79; [1.66, 4.70]), ORF7a:V82A (2.02; [1.33, 3.06]), and ORF8:S24L (0.60; [0.36, 1.00]) were also positively associated with hospitalization.

Next, we optimized our model to predict ICU admission among individuals testing positive for COVID-19. The best-performing algorithm was NaiveBayes on the combined host, environment, and virus variant dataset after feature selection on the training data and no resampling, which performed with an AUROC score of 0.98 (Table 2). For ICU admissions, the clinical factors most strongly associated with a positive correlation include experiencing acute respiratory distress syndrome (ARDS) during hospitalization (257.75; [116.88, 568.40]), requiring ventilation (151.49; [82.35, 278.70]), and co-infection with bacteria (16.49; [8.88, 30.62]) (Figure 2(B)). Similar to hospital admissions, residing in more rural areas was associated with a higher likelihood of ICU admission (1.44; [1.21, 1.72]) compared to residing in more urban areas. Interestingly, SARS-CoV-2 wild-type viruses were negatively associated with ICU admissions (0.24; [0.14 to 0.43]) in comparison to the other variants (Alpha, Beta, Delta, Gamma, Omicron, and Epsilon).

Finally, we predicted long COVID among individuals testing positive for COVID-19. The best-performing model utilized the combined host, environment, and virus variant dataset after feature selection on the training data, random oversampling, followed by ridge regression. The AUROC score was 0.79 (Table 2). Significant predictive features of long COVID included hospital admission (5.02; [3.36, 7.48]), ventilation (4.74; [3.12, 7.20]) and male sex (0.69; [0.50, 0.96]). The COVID-19 epsilon variant (aOR = 0.40, 95% CI = 0.18, 0.86) was less associated with long COVID (Figure 2(C)). In contrast to hospitalization and ICU admission, urban-rural classification did not show a statistically significant association with long COVID.

**Figure 3.**
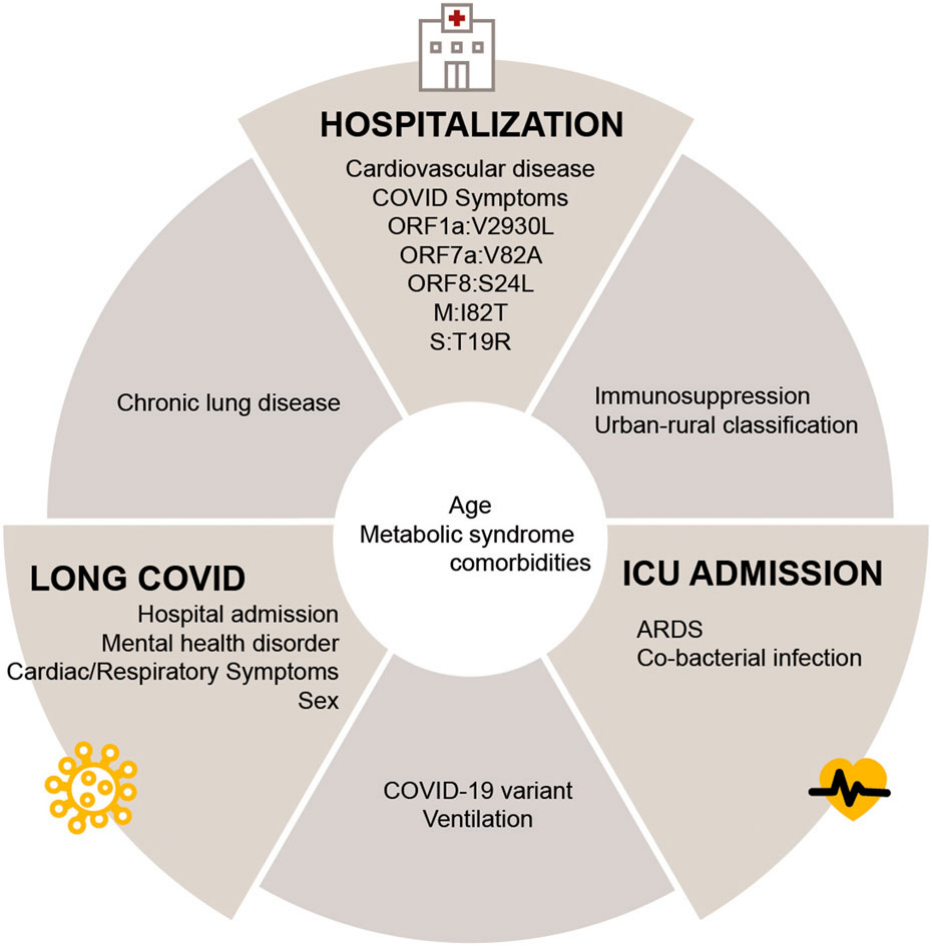
Significant predictors associated with disease outcomes. Features selected from the best-performing models for each outcome (hospitalization, ICU admission, and long COVID) were further analyzed using conventional logistic regression, and significant features were shown. Significant predictive features of multiple outcomes are shown between the respective outcome panels. ARDS, acute respiratory distress syndrome; Symptoms listed under hospitalization included gastrointestinal, constitutional, and cardiac symptoms.

By combining these findings, our results showed common significant features across outcomes (Figure 3). For example, factors such as age and comorbidities associated with metabolic syndrome are common attributes of SARS-CoV-2 infections influencing hospitalization, ICU admission, or long COVID. Chronic lung disease is associated with increased likelihood of hospitalization and long COVID; immunosuppression and rural residence are associated with increased likelihood of both hospitalization and ICU admission; virus variant and ventilation requirements are associated with long COVID and ICU admission.

## Discussion

Personalized medicine, a precision medicine approach, integrates human and environmental factors to tailor disease prevention and treatment and improve patient outcomes. Predominantly implemented in oncology, personalized medicine has had limited implementation in infectious disease [81]. The widely heterogenous constellation of symptoms and complications of microbial infections depends on a multitude of host, virus, and environmental factors, making predicting at-risk individuals challenging. In this study, we developed COVID-19 ViHOP, an ensemble of machine learning algorithms that effectively assesses an individual’s clinical outcome risks, of which we focused on hospitalization, ICU admission, and long COVID. Our findings suggest that the ViHOP portfolio could be useful for clinicians to identify at-risk COVID-19 patients for targeted interventions and lay the groundwork at the frontiers of personalized medicine in infectious diseases.

It is well documented that host factors (e.g. age, comorbidities, and immune status), [11–14] urban-rural classification, [15–24] and viral factors (e.g. SARS-CoV-2 variants and mutations) [25, 26, 82–85] have been linked to patient outcomes. However, most studies tend to focus exclusively on either host or virus factors, or they incorporate only a limited combination of human and viral data [27–33]. In this study, we developed and applied the ViHOP model and attempted to assess whether integrating clinical, viral, and environmental characteristics enhances the predictive capabilities for three key outcomes: hospitalization, ICU admission, and long COVID. Our holistic analyses revealed that all three types of factors – clinical, viral, and environment – are associated with hospitalization and ICU admission, and both clinical and viral factors are associated with all three outcomes, although the specific combination of factors varied depending on the outcome (Figures 2 and 3). Immunosuppression was associated with hospitalization and ICU admission, whereas cardiovascular disease and the appearance of COVID-19 symptoms uniquely predicted hospitalization. Chronic lung disease predicted hospitalization and long COVID. Hospitalization, mental health disorders, female sex, and respiratory and cardiac symptoms were associated with long COVID. Finally, requiring ventilation predicted both ICU admission and long COVID, whereas having ARDS or a bacterial co-infection uniquely predicted ICU admission. The clinical and environmental factors we identified align with those previously reported in the literature [7, 86, 87]. The consistency of our findings with previously identified factors further supports the strength and validity of our models.

Through further analyses, the amino acid substitutions associated with hospitalization risks, such as ORF1a:V2930L, ORF7a:V82A, M:I82 T, and S:T19R, were predominantly located within Delta variants and ORF8:S24L was predominantly located within Wild Type and Epsilon variants in our dataset although these amino acid substitutions were still circulating in human populations until late 2023 (Supplementary Figure 3) Of these amino acid substitutions, S:T19R is mapped to the “supersite” of the N-terminal domain (NTD), which is targeted by anti-NTD neutralizing antibodies, suggesting a potential role in immune evasion [88]. Of interest, the occurrence of amino acid substitutions coincides with the number of hospitalizations in Missouri (Supplementary Figures 1 and 3). Nevertheless, additional studies are needed to understand the mechanisms by which these mutations influence clinical outcomes. In contrast to predicting hospitalization, the COVID-19 variant was sufficient for predicting long COVID and ICU admission. Overall, these findings indicate that, by combining with clinical and residential location factors, basic variant subtyping may be effective for predicting ICU admissions and long COVID, whereas high-resolution whole genome sequencing provides more benefits in assessing hospitalization risks.

Of importance, we saw that urban-rural classification was selected as a predictor for all three outcomes, and living in more urban areas was less associated with both hospital and ICU admission compared to rural residency, supporting that residential aspects such as less access to healthcare and a culture of less healthcare-seeking behaviours may serve an important role in disease outcomes [89, 90]. Rural populations have experienced higher incidences of COVID-19 disease and mortality [18–24, 89, 91, 92]. Rural communities have also been shown to be an important source of virus evolution and spread [93]. Our findings indicate the need for targeted attention and interventions specifically tailored to rural communities.

Our study has potential limitations inherent in the use of electronic health data, such as small sample sizes, possible misclassification, and instances of missing data [94]. To mitigate these potential issues, we employed a combination of automatic data extraction and manual validation to enhance the robustness and reliability of the data used in the analyses. In addition, the majority of data used in this study are from the state of Missouri, and the total number of patients, particularly ICU cases, is relatively small. To minimize the impacts of imbalances in datasets, we implemented oversampling and data imputation methods during the model development phase, as detailed in the Supplementary Methods. Additionally, it is important to note that our study relied on secondary sampling of COVID-19-positive nasopharyngeal swabs, leading to a bias towards individuals who sought healthcare. This group often includes those who are symptomatic, have sufficient healthcare coverage, and reside near healthcare centres. Consequently, this approach may lead to an underestimation of the total number of cases particularly from rural areas. Further refinement of the ViHOP models would benefit from additional longitudinal and prospective multi-centre cohort studies. These studies could provide more comprehensive data, enhancing the accuracy and generalizability of the models.

In summary, this study introduces COVID-19 ViHOP, a portfolio of personalized clinical risk assessment models that can predict the probability of hospitalization, ICU admission, and long COVID for individuals who test positive for COVID-19. This information has the potential to allow clinicians to identify patients with COVID-19 who are at high risk of developing poor outcomes and make informed and personalized decisions for additional monitoring and interventions. While we focused on three primary outcomes, this approach has the potential to be expanded to additional outcome measures such as mortality. Furthermore, this approach can be applied to emerging COVID-19 variants, and we expect to continue updating the models as new SARS-CoV-2 variants emerge. Finally, this model may have utility for other infectious agents. With its ability to integrate multiple data types to predict an individual’s probability of poor outcomes, this tool may serve as a valuable platform in the field of personalized medicine for infectious diseases.

## Supplementary Material

Supplemental Material

Supplemental Material

Supplemental Material

## Data sharing statement

All virus genome data is publicly available on GISAID as the following accession IDs: [Submission in progress]. De-identified clinical data used in this study, the data dictionary, and relevant codes are accessible in the supplementary files.
