# Supplementary material for: Prediction models for COVID-19 disease outcomes
Source: Emerg Microbes Infect. 2024 Jun 3;13(1):2361791. doi: 10.1080/22221751.2024.2361791 (PMC11182058; doi:10.1080/22221751.2024.2361791)
Supplement: Supplemental Material [file TEMI_A_2361791_SM2863.docx]

**Supplementary Figure**


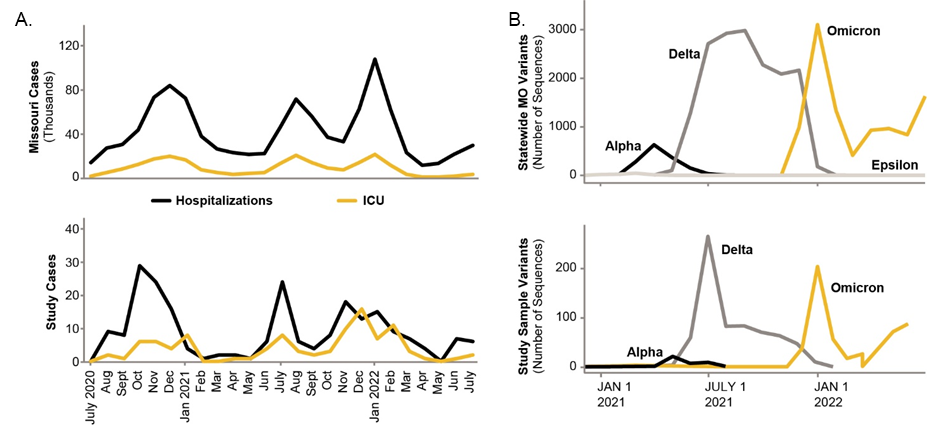


**Supplementary Figure 1. Comparison of COVID-19 trends between statewide Missouri and study sample data.** During the study period between July 2020 and July 2022, a total of 4,307,334 hospital encounters due to COVID-19 were reported from the COVID-19 Hospital Data from the National Hospital Care Survey.^1^ In Missouri, data from the Missouri Department of Health and Senior Services (MDHSS), which became available starting on July 15, 2020-July 31, 2022 revealed a total of 1,054,060 hospitalizations and 241,979 ICU admissions with hospital encounters peaking in December 2020, August 2021, and January 2022.^2^ A. Monthly statewide COVID-19 confirmed hospitalizations and intensive care unit (ICU) admissions reported by the MDHSS (above), and monthly hospitalizations and ICU admissions among study samples (below). B. Statewide predominant variants (outbreak.info) (above) and monthly predominant variants among study samples (below). Both plots in each panel share the x-axis.


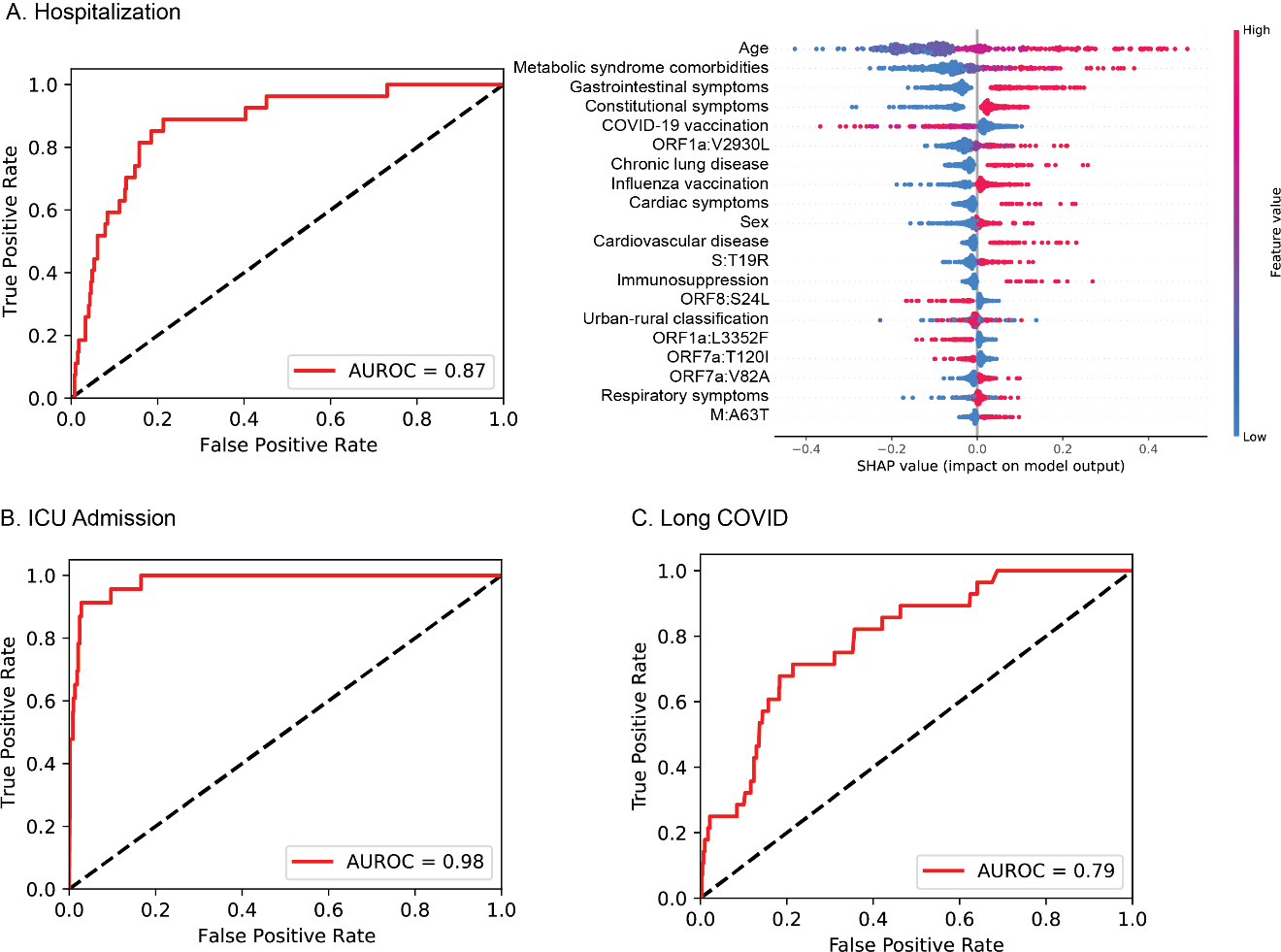


**Supplementary Figure 2**. Area under the receiver operating curves (AUROC) for the top performing models for A) Hospitalization (left), B) ICU admission, and C) Long COVID. Because feature selection was not utilized in the best performing model predicting hospitalization, the right panel of A) shows the beeswarm plot for hospitalization illustrating feature importance. ShAP (SHapley Additive exPlanations) values demonstrating feature importance (right) are shown where the y-axis represents the top 20 features ranked from top to bottom by the highest to lowest mean(|ShAP|) reflecting the strength of each feature’s contribution towards predictions. Positive ShAP indicates positive prediction (hospitalization). Negative ShAP indicates negative prediction (no hospitalization). Feature values, represented by color, indicate raw feature values. Each dot represents an instance (individual), and those with the same or similar ShAP values are stacked vertically.


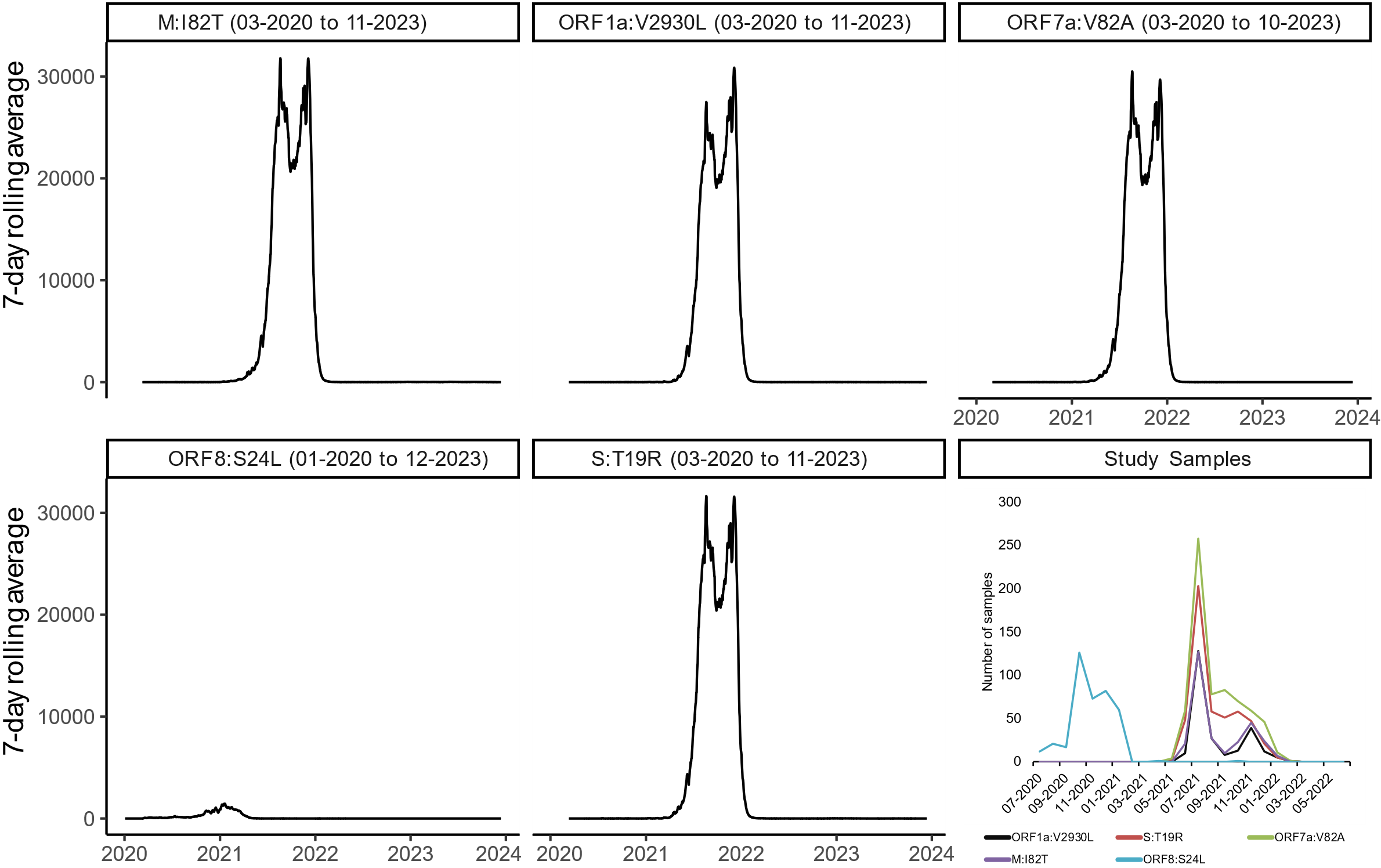


**Supplementary Figure 3**. Worldwide prevalence of statistically significant amino acid substitutions. The 7-day rolling average of samples containing each substitution in GISAID. Data was extracted from OutbreakInfo R v.0.2.0. Dates of first and last detection as of January 1, 2024 for each substitution are shown in parentheses. The bottom right panel shows the total number of monthly samples containing each substitution within our study samples.

**Supplementary Tables**

**Supplementary Table 1. SARS-CoV-2 whole genome sequencing quality**

| **BarCode** | **Swab Date** | **R1 Reads** | **R2 Reads** | **PassQC Reads** | **Matched Reads** | **Total Reads** | **Mean Depth** | **Min Depth** | **Max Depth** |
| --- | --- | --- | --- | --- | --- | --- | --- | --- | --- |
| 1425xA10 | 7/1/2020 | 217919 | 217926 | 432671 | 403959 | 403959 | 2019.53 | 2 | 6660 |
| 1425xE1 | 7/1/2020 | 234169 | 234184 | 465194 | 447394 | 447394 | 2218.10 | 1 | 7368 |
| 1432xE3 | 7/2/2020 | 236520 | 236526 | 469853 | 464447 | 464447 | 2298.25 | 1 | 7482 |
| 1432xH8 | 7/1/2020 | 208295 | 208318 | 413940 | 411969 | 411969 | 1158.58 | 1 | 6728 |
| 1436xB10 | 7/1/2020 | 280164 | 280211 | 556865 | 554350 | 554350 | 2800.99 | 15 | 9415 |
| 1436xE10 | 7/3/2020 | 333840 | 333857 | 664236 | 641766 | 641766 | 3169.69 | 1 | 10730 |
| 1436xE11 | 7/2/2020 | 225485 | 225496 | 448142 | 441396 | 441396 | 2157.19 | 8 | 7080 |
| 1436xE6 | 7/2/2020 | 171161 | 171168 | 340517 | 334988 | 334988 | 1665.25 | 1 | 5369 |
| 1436xE8 | 7/2/2020 | 225550 | 225555 | 448124 | 438721 | 438721 | 2164.29 | 1 | 7202 |
| 1436xH6 | 7/2/2020 | 216879 | 216892 | 430703 | 419593 | 419593 | 2081.66 | 4 | 6924 |
| 1437xH6 | 7/2/2020 | 204064 | 204054 | 407245 | 391123 | 391123 | 1843.00 | 3 | 6506 |
| 1438xA11 | 7/2/2020 | 219378 | 219374 | 436793 | 11841 | NA | 101.67 | 2 | 368 |
| 1438xA5 | 7/2/2020 | 160146 | 160143 | 318417 | 314014 | 314014 | 1585.61 | 1 | 5342 |
| 1446xH9 | 7/3/2020 | 198789 | 198791 | 395099 | 379409 | 379409 | 1888.69 | 1 | 6286 |
| 1452xG2 | 7/5/2020 | 199352 | 199332 | 395776 | 378491 | 378491 | 1882.28 | 1 | 6209 |
| 1453xB8 | 7/5/2020 | 200704 | 200702 | 400500 | 393686 | 393686 | 1876.82 | 1 | 6229 |
| 1453xB9 | 7/5/2020 | 230466 | 230458 | 459913 | 456671 | 456671 | 2137.83 | 1 | 7067 |
| 1453xE2 | 7/5/2020 | 217108 | 217103 | 432841 | 428099 | 428099 | NA | NA | NA |
| 1453xH10 | 7/5/2020 | 261993 | 262012 | 520477 | 515384 | 515384 | 2544.85 | 8 | 8400 |
| 1463xC12 | 7/6/2020 | 200215 | 200214 | 398120 | 392484 | 392484 | 1924.62 | 2 | 6383 |
| 1463xC2 | 7/6/2020 | 209503 | 209505 | 416279 | 411064 | 411064 | 2030.91 | 3 | 7031 |
| 1463xD12 | 7/6/2020 | 226437 | 226447 | 450508 | 446252 | 446252 | 2202.51 | 1 | 7228 |
| 1466xB11 | 7/6/2020 | 186229 | 186251 | 370159 | 369650 | 369650 | 1781.92 | 1 | 5544 |
| 1466xB3 | 7/6/2020 | 227766 | 227763 | 454633 | 450489 | 450489 | 2147.14 | 1 | 7085 |
| 1466xH2 | 7/6/2020 | 200209 | 160341 | 351185 | 350452 | 350452 | 1855.53 | 2 | 6219 |
| 1469xA1 | 7/6/2020 | 260130 | 260138 | 517312 | 515288 | 515288 | 2549.86 | 1 | 8765 |
| 1475xC11 | 7/7/2020 | 208570 | 208587 | 414672 | 413739 | 413739 | 2009.23 | 1 | 6807 |
| 1475xC9 | 7/6/2020 | 257521 | 257536 | 511869 | 501523 | 501523 | 2447.86 | 1 | 8115 |
| 1475xD8 | 7/7/2020 | 198133 | 198153 | 393706 | 391627 | 391627 | 1923.10 | 1 | 6238 |
| 1475xE3 | 7/7/2020 | 207748 | 207761 | 412722 | 410969 | 410969 | 2012.67 | 1 | 6862 |
| 1479xG9 | 7/7/2020 | 257194 | 257205 | 512625 | 506340 | 506340 | 2446.98 | 1 | 7342 |
| 1481xA11 | 7/7/2020 | 231286 | 231287 | 460020 | 443607 | 443607 | 2210.48 | 1 | 7509 |
| 1481xD2 | 7/7/2020 | 331138 | 331142 | 660386 | 653667 | 653667 | 3094.50 | 1 | 10726 |
| 1481xH5 | 7/7/2020 | 217885 | 217895 | 433057 | 425867 | 425867 | 2076.25 | 1 | 7232 |
| 1482xB4 | 7/7/2020 | 299033 | 299051 | 595710 | 591885 | 591885 | 2896.74 | 2 | 9713 |
| 1483xA7 | 7/7/2020 | 244441 | 244432 | 487825 | 483395 | 483395 | 2381.97 | 1 | 8215 |
| 1483xF2 | 7/7/2020 | 177286 | 177283 | 352598 | 351136 | 351136 | 1743.20 | 1 | 5834 |
| 1484xA3 | 7/7/2020 | 216400 | 216394 | 430462 | 422229 | 422229 | 2046.16 | 2 | 6990 |
| 1484xB12 | 7/7/2020 | 238818 | 238810 | 476559 | 448282 | 448282 | 2134.42 | 4 | 7442 |
| 1484xE9 | 7/7/2020 | 146286 | 146277 | 290920 | 284974 | 284974 | 1412.29 | 1 | 4756 |
| 1499xA4 | 7/8/2020 | 235000 | 235000 | 468836 | 463794 | 463794 | 2162.78 | 1 | 7458 |
| 1499xA8 | 7/8/2020 | 169278 | 169267 | 336216 | 290178 | 290178 | 1415.89 | 4 | 4878 |
| 1499xC10 | 7/8/2020 | 218446 | 218447 | 434150 | 420141 | 420141 | 2071.04 | 10 | 7083 |
| 1499xC11 | 7/8/2020 | 116803 | 210519 | 325701 | 320775 | 320775 | 1890.63 | 7 | 6289 |
| 1499xD3 | 7/8/2020 | 206926 | 206924 | 411702 | 352103 | 352103 | 1645.23 | 1 | 5667 |
| 1499xF5 | 7/8/2020 | 167016 | 167016 | 330422 | 296246 | 296246 | 1479.99 | 1 | 4855 |
| 1499xF7 | 7/8/2020 | 211755 | 211762 | 420855 | 410590 | 410590 | 2071.64 | 1 | 6769 |
| 1499xH8 | 7/8/2020 | 208384 | 208401 | 413985 | 388315 | 388315 | 1858.78 | 1 | 5991 |
| 1501xA6 | 7/8/2020 | 219742 | 219773 | 436008 | 423556 | 423556 | 2087.28 | 10 | 7195 |
| 1501xG3 | 7/8/2020 | 239624 | 239652 | 475602 | 472346 | 472346 | 2344.06 | 1 | 7840 |
| 1502xA3 | 7/8/2020 | 180521 | 180514 | 358744 | 352527 | 352527 | 1698.23 | 1 | 5774 |
| 1502xB4 | 7/8/2020 | 256723 | 256728 | 509963 | 502030 | 502030 | 2459.27 | 5 | 8334 |
| 1502xE7 | 7/8/2020 | 232091 | 232106 | 461582 | 459937 | 459937 | 2224.62 | 5 | 7354 |
| 1503xA7 | 7/8/2020 | 269930 | 269925 | 537429 | 465394 | 465394 | 2311.91 | 1 | 8238 |
| 1514xE7 | 7/9/2020 | 169105 | 169120 | 336360 | 334812 | 334812 | 1655.68 | 8 | 5569 |
| 1515xH4 | 7/9/2020 | 276792 | 276804 | 549579 | 540481 | 540481 | 2695.92 | 1 | 9283 |
| 1516xB10 | 7/9/2020 | 234053 | 234079 | 465076 | 460909 | 460909 | 2257.51 | 2 | 7788 |
| 1516xC4 | 7/9/2020 | 191187 | 191207 | 379458 | 367769 | 367769 | 1851.09 | 1 | 6114 |
| 1516xD7 | 7/9/2020 | 217077 | 217090 | 430669 | 396859 | 396859 | 1949.85 | 6 | 6500 |
| 1516xF10 | 7/9/2020 | 221386 | 221390 | 439981 | 422348 | 422348 | 2098.36 | 4 | 7151 |
| 1516xG4 | 7/9/2020 | 246055 | 246091 | 488616 | 476182 | 476182 | 2202.96 | 1 | 7193 |
| 1516xG7 | 7/9/2020 | 218850 | 218849 | 435386 | 433158 | 433158 | 2079.67 | 1 | 7091 |
| 1518xB3 | 7/9/2020 | 277220 | 277222 | 550406 | 506113 | 506113 | 2525.24 | 3 | 8662 |
| 1518xE12 | 7/9/2020 | 194116 | 194127 | 385290 | 364878 | 364878 | 1416.46 | 1 | 4658 |
| 1518xG10 | 7/9/2020 | 244085 | 244078 | 487164 | 479604 | 479604 | 2235.07 | 1 | 7535 |
| 1529xC1 | 7/8/2020 | 258199 | 258204 | 514778 | 507156 | 507156 | 2518.10 | 1 | 8582 |
| 1529xF8 | 7/8/2020 | 323707 | 323719 | 644402 | 642672 | 642672 | 3144.73 | 14 | 10689 |
| 1530xF7 | 7/9/2020 | 231254 | 231259 | 459916 | 451624 | 451624 | 2169.77 | 1 | 7352 |
| 1573xB2 | 7/12/2020 | 191730 | 191758 | 380668 | 379319 | 379319 | 1871.42 | 1 | 5512 |
| 20COX5001NPS | 8/12/2020 | 1006352 | 1006442 | 2011059 | 2003799 | 2003799 | 8525.99 | 4 | 22306 |
| 20COX5006NPS | 8/12/2020 | 988384 | 988471 | 1974295 | 1618904 | 1618904 | 796.15 | 4 | 2211 |
| 20COX5008NPS | 8/12/2020 | 1105537 | 1105632 | 2209436 | 2192124 | 2192124 | 9295.90 | 3 | 25223 |
| 20COX5009NPS | 8/12/2020 | 1338386 | 1338461 | 2674933 | 2563759 | 2563759 | 10987.19 | 1 | 29561 |
| 20COX5010NPS | 8/12/2020 | 1013213 | 1013249 | 2024903 | 2019182 | 2019182 | 7112.36 | 1 | 23432 |
| 20COX5011NPS | 8/12/2020 | 1001476 | 1001693 | 2001161 | 1988338 | 1988338 | 32.74 | 1 | 96 |
| 20COX5014NPS | 8/12/2020 | 1296386 | 1296542 | 2590745 | 2583218 | 2583218 | 10936.18 | 3 | 30946 |
| 20COX5019NPS | 8/13/2020 | 1309959 | 1310077 | 2617729 | 142907 | 142907 | 700.64 | 1 | 3919 |
| 20COX5020NPS | 8/14/2020 | 970855 | 970959 | 1940145 | 1930263 | 1930263 | 8146.63 | 3 | 23905 |
| 20COX5021NPS | 8/13/2020 | 1272019 | 1272261 | 2541140 | 2511195 | 2511195 | 10540.09 | 1 | 30597 |
| 20COX5022NPS | 8/13/2020 | 1885920 | 1886724 | 3769473 | 3599434 | 3599434 | 9950.25 | 1 | 35866 |
| 20COX5024NPS | 8/13/2020 | 1068812 | 1068939 | 2135370 | 2056877 | 2056877 | 8786.48 | 1 | 25036 |
| 20COX5025NPS | 8/13/2020 | 1273033 | 1273174 | 2543200 | 2321623 | 2321623 | 9819.07 | 1 | 33575 |
| 20COX5026NPS | 8/13/2020 | 1033370 | 1033398 | 2065359 | 1953974 | 1953974 | 8332.37 | 1 | 29561 |
| 20COX5027NPS | 8/13/2020 | 1540468 | 1540985 | 3078395 | 2609848 | 2609848 | 8250.37 | 1 | 28939 |
| 20COX5029NPS | 8/13/2020 | 1469387 | 1469874 | 2936866 | 2819546 | 2819546 | 10006.67 | 1 | 24379 |
| 20COX5030NPS | 8/13/2020 | 904525 | 905796 | 1807898 | 1519803 | 1519803 | 5128.00 | 2 | 17075 |
| 20COX5031NPS | 8/12/2020 | 1641994 | 1642728 | 3281272 | 2986131 | 2986131 | 11537.77 | 1 | 46790 |
| 20COX5032NPS | 8/13/2020 | 1131402 | 1131448 | 2260888 | 2188493 | 2188493 | 9327.44 | 1 | 27505 |
| 20COX5033NPS | 8/13/2020 | 1129403 | 1129502 | 2256924 | 2202991 | 2202991 | 9351.16 | 1 | 26241 |
| 20COX5034NPS | 8/13/2020 | 1066354 | 1066486 | 2130337 | 2074507 | 2074507 | 8852.78 | 2 | 30329 |
| 20COX5036NPS | 8/13/2020 | 1187075 | 1187242 | 2372067 | 2258986 | 2258986 | 9599.91 | 4 | 38228 |
| 20COX5037NPS | 8/13/2020 | 1048028 | 1047970 | 2092330 | 624214 | 624214 | 2671.71 | 1 | 9016 |
| 20COX5039NPS | 8/13/2020 | 956458 | 957261 | 1910809 | 1524154 | 1524154 | 5986.28 | 1 | 20142 |
| 20COX5042NPS | 8/13/2020 | 1423411 | 1424514 | 2844784 | 2614796 | 2614796 | 8105.35 | 1 | 27497 |
| 20COX5043NPS | 8/13/2020 | 830865 | 831449 | 1659466 | 942848 | 942848 | 56183.43 | 1 | 161756 |
| 20COX5045NPS | 8/13/2020 | 931242 | 931362 | 1861027 | 1806376 | 1806376 | 7689.31 | 1 | 28393 |
| 20COX5046NPS | 8/13/2020 | 1004075 | 1004198 | 2005661 | 1663920 | 1663920 | 7097.33 | 1 | 19920 |
| 20COX5047NPS | 8/13/2020 | 1494118 | 1494603 | 2984416 | 1840146 | 1840146 | 7103.45 | 1 | 20353 |
| 20COX5074NPS | 8/18/2020 | 867000 | 867192 | 1732269 | 1684446 | 1684446 | 5581.46 | 4 | 17576 |
| 20COX5075NPS | 8/18/2020 | 877844 | 878002 | 1754414 | 1712269 | 1712269 | 7252.97 | 1 | 22278 |
| 20COX5077NPS | 8/18/2020 | 960360 | 960452 | 1918985 | 1872193 | 1872193 | 7952.55 | 1 | 24908 |
| 20COX5081NPS | 8/17/2020 | 1123497 | 1124531 | 2245198 | 1543849 | 1543849 | 46046.37 | 1 | 136077 |
| 20COX5085NPS | 8/19/2020 | 901889 | 902614 | 1801359 | 1243166 | 1243166 | 4872.04 | 2 | 12922 |
| 20COX5086NPS | 8/19/2020 | 1056848 | 1057011 | 2112072 | 2030488 | 2030488 | 8588.54 | 1 | 28004 |
| 20COX5087NPS | 8/19/2020 | 1691230 | 1692214 | 3380552 | 3153931 | 3153931 | 11822.96 | 4 | 40529 |
| 20COX5088NPS | 8/18/2020 | 1024884 | 1025082 | 2047943 | 2003339 | 2003339 | 7975.62 | 1 | 25771 |
| 20COX5090NPS | 8/19/2020 | 1000132 | 1000559 | 1998896 | 1988901 | 1988901 | 7643.82 | 1 | 26602 |
| 20COX5091NPS | 8/19/2020 | 1218981 | 0 | 1218417 | 903919 | 903919 | 4400.83 | 2 | 11885 |
| 20COX5095NPS | 8/19/2020 | 1066653 | 1083679 | 2127008 | 2026572 | 2026572 | 8628.91 | 1 | 27061 |
| 20COX5096NPS | 8/20/2020 | 1693281 | 1694564 | 1289408 | 1197980 | 1197980 | 5190.13 | 3 | 18280 |
| 20COX5097NPS | 8/20/2020 | 1037943 | 1038068 | 2073929 | 2051271 | 2051271 | 8747.36 | 1 | 31800 |
| 20COX5098NPS | 8/20/2020 | 1342573 | 1343254 | 2661274 | 432504 | 432504 | 1937.45 | 1 | 8767 |
| 20COX5099NPS | 8/20/2020 | 1641278 | 1642011 | 3278903 | 2142138 | 2142138 | 7960.11 | 1 | 20273 |
| 20COX5101NPS | 8/20/2020 | 1330695 | 1330990 | 2656105 | 163591 | 163591 | 667.43 | 5 | 2614 |
| 20COX5106NPS | 8/21/2020 | 992223 | 992394 | 1983042 | 1936141 | 1936141 | 8151.12 | 3 | 26148 |
| 20COX5107NPS | 8/21/2020 | 1426387 | 1426942 | 2850981 | 2826617 | 2826617 | 3367.80 | 1 | 10597 |
| 20COX5108NPS | 8/20/2020 | 1800938 | 1801501 | 3599806 | 3479572 | 3479572 | 13326.66 | 1 | 46951 |
| 20COX5109NPS | 8/21/2020 | 1137421 | 1137897 | 2272857 | 2130047 | 2130047 | 8724.07 | 1 | 32339 |
| 20COX5110NPS | 8/21/2020 | 828872 | 829306 | 1654993 | 780428 | 780428 | 3094.22 | 1 | 12288 |
| 20COX5111NPS | 8/21/2020 | 1589913 | 1590450 | 3177616 | 3115262 | 3115262 | 12237.34 | 2 | 50394 |
| 20COX5112NPS | 8/21/2020 | 1921636 | 1922273 | 3840250 | 3398232 | 3398232 | 13054.42 | 1 | 49452 |
| 20COX5113NPS | 8/21/2020 | 1329879 | 1330253 | 2657932 | 2434023 | 2434023 | 9290.18 | 1 | 40024 |
| 20COX5114NPS | 8/22/2020 | 187328 | 89470 | 276134 | 267635 | 267635 | 1173.46 | 1 | 3941 |
| 20COX5115NPS | 8/22/2020 | 975357 | 975492 | 1948998 | 1907578 | 1907578 | 8191.62 | 1 | 28388 |
| 20COX5116NPS | 8/22/2020 | 1204958 | 1205681 | 2408428 | 2386451 | 2386451 | 764.93 | 1 | 6242 |
| 20COX5117NPS | 8/22/2020 | 1000888 | 1001281 | 1999774 | 1834295 | 1834295 | 7423.33 | 1 | 26732 |
| 20COX5118NPS | 8/22/2020 | 1017129 | 1017586 | 2030621 | 343351 | 343351 | 1350.22 | 1 | 5640 |
| 20COX5120NPS | 8/22/2020 | 1215171 | 1215533 | 2427921 | 2127113 | 2127113 | 8436.98 | 3 | 30599 |
| 20COX5122NPS | 8/22/2020 | 1344613 | 1345071 | 2687258 | 2644310 | 2644310 | 10084.72 | 1 | 40569 |
| 20COX5123NPS | 8/22/2020 | 715976 | 716032 | 1430824 | 1346817 | 1346817 | 5744.31 | 1 | 22071 |
| 20COX5127NPS | 8/27/2020 | 1565632 | 1566163 | 3129122 | 2849854 | 2849854 | 8383.34 | 1 | 32491 |
| 20COX5128NPS | 8/27/2020 | 654712 | 655054 | 1307628 | 840210 | 840210 | 3340.14 | 1 | 16993 |
| 20COX5131NPS | 8/27/2020 | 1441039 | 1441493 | 2879966 | 2820702 | 2820702 | 11473.25 | 1 | 39872 |
| 20COX5133NPS | 8/27/2020 | 1492948 | 1493978 | 2984040 | 2809994 | 2809994 | 7420.31 | 1 | 31864 |
| 20COX5134NPS | 8/28/2020 | 1678917 | 1680247 | 3355363 | 3232064 | 3232064 | 6111.83 | 1 | 26202 |
| 20COX5136NPS | 8/28/2020 | 1217776 | 1218549 | 2433607 | 1888249 | 1888249 | 5462.41 | 3 | 23133 |
| 20COX5137NPS | 8/28/2020 | 744937 | 745462 | 1487219 | 712505 | 712505 | 2775.69 | 18 | 14046 |
| 20COX5139NPS | 8/28/2020 | 1852767 | 1853631 | 3703185 | 3527013 | 3527013 | 13552.78 | 1 | 48436 |
| 20COX5141NPS | 8/29/2020 | 1416981 | 1417857 | 2832143 | 2792417 | 2792417 | 10684.50 | 1 | 38223 |
| 20COX5143NPS | 8/29/2020 | 1318903 | 1319254 | 2635914 | 2599874 | 2599874 | 10277.89 | 1 | 35897 |
| 20COX5145NPS | 8/29/2020 | 850049 | 850209 | 1698371 | 1416450 | 1416450 | 5813.94 | 2 | 24067 |
| 20COX5150NPS | 9/6/2020 | 1591990 | 1592569 | 3180971 | 2377208 | 2377208 | 9268.27 | 1 | 35812 |
| 20COX5151NPS | 9/7/2020 | 1531204 | 1531888 | 3060016 | 2888791 | 2888791 | 11208.36 | 2 | 38640 |
| 20COX5152NPS | 9/6/2020 | 1438274 | 1439005 | 2874537 | 2782491 | 2782491 | 10683.23 | 1 | 47642 |
| 20COX5153NPS | 9/6/2020 | 1317206 | 1317720 | 2632789 | 2472166 | 2472166 | 9539.53 | 1 | 35159 |
| 20COX5154NPS | 9/6/2020 | 1633192 | 1633762 | 3264088 | 3185132 | 3185132 | 12476.60 | 1 | 42117 |
| 20COX5157NPS | 9/6/2020 | 553449 | 553702 | 1104535 | 205445 | 205445 | 843.88 | 2 | 4073 |
| 20COX5160NPS | 9/7/2020 | 1144774 | 1144885 | 2287199 | 1994696 | 1994696 | 8431.82 | 1 | 25648 |
| 20COX5161NPS | 9/7/2020 | 845669 | 845777 | 1689916 | 1590994 | 1590994 | 6729.95 | 1 | 19661 |
| 20COX5162NPS | 9/8/2020 | 955481 | 955629 | 1909242 | 1865190 | 1865190 | 1237.86 | 1 | 8586 |
| 20COX5163NPS | 9/7/2020 | 893545 | 893673 | 1785560 | 1692446 | 1692446 | 7175.01 | 1 | 24222 |
| 20COX5164NPS | 9/7/2020 | 1172840 | 1174159 | 2342019 | 1084242 | 1084242 | 4079.70 | 3 | 22031 |
| 20COX5166NPS | 9/7/2020 | 1123811 | 1124370 | 2245882 | 2068962 | 2068962 | 5102.96 | 1 | 14842 |
| 20COX5171NPS | 9/7/2020 | 1184986 | 1185140 | 2367143 | 2046171 | 2046171 | 8666.48 | 1 | 29984 |
| 20COX5172NPS | 9/7/2020 | 1024446 | 1024626 | 2046901 | 2000799 | 2000799 | 8498.16 | 9 | 29872 |
| 20COX5173NPS | 9/7/2020 | 1697463 | 1698414 | 3392998 | 3305147 | 3305147 | 12783.44 | 1 | 44487 |
| 20COX5174NPS | 9/7/2020 | 1210401 | 1210839 | 2418989 | 2271306 | 2271306 | 8933.91 | 7 | 31549 |
| 20COX5178NPS | 9/7/2020 | 1525504 | 1526200 | 3048956 | 2933188 | 2933188 | 11268.76 | 1 | 37820 |
| 20COX5179NPS | 9/8/2020 | 966122 | 966256 | 1930862 | 1912796 | 1912796 | 8060.24 | 2 | 25397 |
| 20COX5180NPS | 9/8/2020 | 2141278 | 2142204 | 4279003 | 4139129 | 4139129 | 15980.32 | 1 | 64973 |
| 20COX5181NPS | 9/8/2020 | 1007577 | 1007750 | 2013551 | 1961593 | 1961593 | 8252.93 | 3 | 31322 |
| 20COX5183NPS | 9/8/2020 | 1744144 | 1744709 | 3484736 | 2602235 | 2602235 | 10139.50 | 3 | 31242 |
| 20COX5187NPS | 9/8/2020 | 1031947 | 1032099 | 2062212 | 1988714 | 1988714 | 8331.00 | 1 | 30098 |
| 20COX5189NPS | 9/8/2020 | 935361 | 935570 | 1868983 | 1813980 | 1813980 | 7568.88 | 1 | 34068 |
| 20COX5191NPS | 9/8/2020 | 1329007 | 1329490 | 2654664 | 1937278 | 1937278 | 7563.28 | 1 | 28545 |
| 20COX5193NPS | 9/8/2020 | 1376244 | 1377027 | 2748672 | 2657698 | 2657698 | 10588.18 | 1 | 30629 |
| 20COX5195NPS | 9/8/2020 | 997799 | 997979 | 1993140 | 1019783 | 1019783 | 4099.67 | 1 | 12884 |
| 20COX5196NPS | 9/8/2020 | 1330445 | 1330831 | 2658957 | 2556708 | 2556708 | 10050.00 | 1 | 36429 |
| 20COX5198NPS | 9/8/2020 | 1396353 | 1396761 | 2791119 | 2776352 | 2776352 | 10759.73 | 2 | 39126 |
| 20COX5199NPS | 9/8/2020 | 954696 | 954807 | 1907717 | 1890427 | 1890427 | 7955.03 | 1 | 29889 |
| 20COX5205NPS | 9/9/2020 | 1192663 | 1192818 | 2383528 | 2322408 | 2322408 | 9513.24 | 1 | 32648 |
| 20COX5206NPS | 9/9/2020 | 1366592 | 1366766 | 2729802 | 2145113 | 2145113 | 8129.01 | 1 | 31459 |
| 20COX5207NPS | 9/9/2020 | 980222 | 980343 | 1958815 | 1889547 | 1889547 | 5105.78 | 1 | 27035 |
| 20COX5208NPS | 9/9/2020 | 1059687 | 1059924 | 2116461 | 1838749 | 1838749 | 5532.64 | 5 | 27027 |
| 20COX5209NPS | 9/9/2020 | 1256539 | 1256777 | 2511014 | 2269843 | 2269843 | 5816.62 | 1 | 34649 |
| 20COX5211NPS | 9/9/2020 | 1386388 | 1386838 | 2769923 | 2003973 | 2003973 | 8020.25 | 2 | 28281 |
| 20COX5214NPS | 9/9/2020 | 1420604 | 1421055 | 2838915 | 2707992 | 2707992 | 6047.52 | 1 | 38895 |
| 20COX5216NPS | 9/9/2020 | 1409646 | 1410297 | 2817739 | 2794150 | 2794150 | 6814.76 | 1 | 34991 |
| 20COX5218NPS | 9/9/2020 | 1714102 | 1718981 | 3426447 | 3199631 | 3199631 | 8870.71 | 1 | 36822 |
| 20COX5219NPS | 9/9/2020 | 1482668 | 1483262 | 2963048 | 2915908 | 2915908 | 6897.15 | 1 | 44454 |
| 20COX5220NPS | 9/9/2020 | 1379467 | 1379953 | 2756880 | 2618047 | 2618047 | 10344.09 | 1 | 42166 |
| 20COX5223NPS | 9/28/2020 | 772776 | 772821 | 1542373 | 5660 | 5660 | 478.91 | 1 | 748 |
| 20COX5224NPS | 9/29/2020 | 1914529 | 1914920 | 3826107 | 3289918 | 3289918 | 7646.88 | 1 | 42877 |
| 20COX5225NPS | 9/29/2020 | 1406163 | 1406650 | 2810240 | 2582557 | 2582557 | 7193.85 | 3 | 33417 |
| 20COX5227NPS | 9/29/2020 | 1527902 | 1528377 | 3054123 | 2960738 | 2960738 | 7610.18 | 1 | 46792 |
| 20COX5229NPS | 9/29/2020 | 1400790 | 1401213 | 2798469 | 2067591 | 2067591 | 4866.55 | 1 | 34052 |
| 20COX5230NPS | 9/29/2020 | 1626111 | 1626670 | 3249860 | 3141593 | 3141593 | 7864.62 | 1 | 39332 |
| 20COX5231NPS | 9/28/2020 | 1216834 | 1217056 | 2429989 | 1590820 | 1590820 | 6520.75 | 1 | 30206 |
| 20COX5237NPS | 9/29/2020 | 1532240 | 1532956 | 3062571 | 3026254 | 3026254 | 11835.16 | 1 | 39088 |
| 20COX5246NPS | 9/30/2020 | 918858 | 918996 | 1835354 | 841182 | 841182 | 3390.75 | 1 | 14101 |
| 20COX5247NPS | 9/30/2020 | 1121642 | 1125691 | 2242721 | 2164488 | 2164488 | 7063.32 | 1 | 28685 |
| 20COX5248NPS | 9/30/2020 | 1410917 | 1411436 | 2819766 | 2763013 | 2763013 | 10715.66 | 1 | 41090 |
| 20COX5249NPS | 9/30/2020 | 1640461 | 1640804 | 3278629 | 3238784 | 3238784 | 12198.82 | 3 | 42814 |
| 20COX5251NPS | 9/30/2020 | 1596204 | 1596769 | 3188852 | 2779969 | 2779969 | 11056.73 | 3 | 44059 |
| 20COX5252NPS | 9/30/2020 | 1189060 | 1189291 | 2374724 | 998850 | 998850 | 3955.04 | 1 | 14602 |
| 20COX5254NPS | 9/30/2020 | 1043863 | 1044011 | 2085135 | 1390647 | 1390647 | 5644.62 | 1 | 29427 |
| 20COX5255NPS | 9/30/2020 | 1094417 | 1094473 | 2183704 | 70679 | 70679 | 290.08 | 1 | 1257 |
| 20COX5258NPS | 10/1/2020 | 1607226 | 1608660 | 3212497 | 3135628 | 3135628 | 11717.60 | 1 | 40775 |
| 20COX5262NPS | 10/1/2020 | 29263092 | 29278170 | 58462826 | 52167103 | 52167103 | 208266.86 | 1 | 1002986 |
| 20COX5263NPS | 10/1/2020 | 29671259 | 29683739 | 59286256 | 56682996 | 56682996 | 135078.16 | 1 | 884385 |
| 20COX5264NPS | 10/1/2020 | 62578866 | 62607103 | 125013513 | 115251230 | 115251230 | 187918.81 | 1 | 954075 |
| 20COX5265NPS | 10/1/2020 | 27557429 | 27565851 | 54963685 | 1397445 | 1397445 | 4956.37 | 1 | 79726 |
| 20COX5267NPS | 10/1/2021 | 29748336 | 29759386 | 59422702 | 54180338 | 54180338 | 167202.28 | 1 | 675989 |
| 20COX5268NPS | 10/1/2020 | 33922653 | 33936434 | 67788157 | 66633225 | 66633225 | 215248.42 | 1 | 986545 |
| 20COX5269NPS | 10/1/2020 | 26514101 | 26525044 | 52984471 | 49411393 | 49411393 | 101670.07 | 1 | 654355 |
| 20COX5271NPS | 10/1/2020 | 35031162 | 35042528 | 69965634 | 45207169 | 45207169 | 92746.97 | 1 | 713336 |
| 20COX5273NPS | 10/1/2020 | 34244925 | 34262099 | 68331561 | 2755123 | 2755123 | 7372.28 | 1 | 112377 |
| 20COX5274NPS | 10/1/2020 | 8628427 | 8630488 | 17219953 | 632719 | 632719 | 2125.54 | 1 | 13387 |
| 20COX5285NPS | 10/1/2020 | 861648 | 861644 | 1721800 | 1671681 | 1671681 | 7469.35 | 3 | 20425 |
| 20COX5287NPS | 10/2/2020 | 754431 | 754424 | 1507773 | 1438108 | 1438108 | 6405.94 | 1 | 17471 |
| 20COX5290NPS | 10/2/2020 | 986708 | 986747 | 1971788 | 1956522 | 1956522 | 4462.91 | 1 | 25715 |
| 20COX5291NPS | 10/2/2020 | 952266 | 952276 | 1903044 | 1895298 | 1895298 | 6741.13 | 1 | 24449 |
| 20COX5292NPS | 10/2/2020 | 828078 | 828088 | 1655038 | 1630640 | 1630640 | 7273.66 | 1 | 20107 |
| 20COX5294NPS | 10/1/2020 | 900461 | 900456 | 1799494 | 1780982 | 1780982 | 7952.91 | 1 | 22278 |
| 20COX5295NPS | 10/2/2020 | 892440 | 892425 | 1783953 | 1748485 | 1748485 | 6167.60 | 2 | 21988 |
| 20COX5299NPS | 10/2/2020 | 913898 | 913930 | 1825912 | 1757627 | 1757627 | 7742.13 | 1 | 22951 |
| 20COX5301NPS | 10/2/2020 | 799054 | 799026 | 1597203 | 1573227 | 1573227 | 5549.11 | 1 | 20298 |
| 20COX5305NPS | 10/2/2020 | 817102 | 817073 | 1633211 | 1551351 | 1551351 | 5388.32 | 1 | 19359 |
| 20COX5310NPS | 10/2/2020 | 784665 | 784688 | 1567371 | 1488915 | 1488915 | 6576.51 | 2 | 17705 |
| 20COX5316NPS | 10/2/2020 | 793454 | 793460 | 1585686 | 1546698 | 1546698 | 6904.58 | 1 | 19437 |
| 20COX5318NPS | 10/2/2020 | 808084 | 808074 | 1614755 | 1496220 | 1496220 | 5207.27 | 4 | 19356 |
| 20COX5319NPS | 10/2/2020 | 722076 | 722055 | 1443341 | 1413211 | 1413211 | 6287.64 | 4 | 17464 |
| 20COX5321NPS | 10/2/2020 | 824410 | 824405 | 1647423 | 1614870 | 1614870 | 5616.53 | 1 | 19955 |
| 20COX5323NPS | 10/5/2020 | 697409 | 697405 | 1393809 | 1323099 | 1323099 | 5997.25 | 1 | 19884 |
| 20COX5324NPS | 10/5/2020 | 1559474 | 1560094 | 3113948 | 567738 | 567738 | 19689.11 | 1 | 84849 |
| 20COX5325NPS | 10/6/2020 | 783001 | 782998 | 1564414 | 1468616 | 1468616 | 6481.99 | 1 | 18998 |
| 20COX5326NPS | 10/6/2020 | 830545 | 830545 | 1659958 | 1638811 | 1638811 | 7262.94 | 1 | 19823 |
| 20COX5334NPS | 10/6/2020 | 819504 | 819442 | 1638217 | 1616538 | 1616538 | 7116.76 | 1 | 20449 |
| 20COX5338NPS | 10/6/2020 | 813190 | 813181 | 1625474 | 1610653 | 1610653 | 6013.03 | 1 | 21169 |
| 20COX5339NPS | 10/6/2020 | 927193 | 927205 | 1852867 | 1830616 | 1830616 | 5398.07 | 1 | 22615 |
| 20COX5345NPS | 10/6/2020 | 874458 | 874461 | 1747407 | 1713309 | 1713309 | 5056.88 | 2 | 21662 |
| 20COX5347NPS | 10/6/2020 | 790462 | 790468 | 1579861 | 1553951 | 1553951 | 5825.51 | 2 | 18285 |
| 20COX5348NPS | 10/6/2020 | 781737 | 781743 | 1562422 | 1550550 | 1550550 | 6883.94 | 3 | 19064 |
| 20COX5350NPS | 10/7/2020 | 889855 | 889866 | 1778325 | 1770422 | 1770422 | 6732.50 | 2 | 22356 |
| 20COX5358NPS | 10/7/2020 | 951793 | 951792 | 1902067 | 1884936 | 1884936 | 7132.37 | 1 | 23257 |
| 20COX5359NPS | 10/7/2020 | 753402 | 753378 | 1505775 | 1474265 | 1474265 | 5572.89 | 1 | 19117 |
| 20COX5360NPS | 10/7/2020 | 777404 | 777424 | 1553170 | 1537262 | 1537262 | 6789.06 | 1 | 18724 |
| 20COX5361NPS | 10/7/2020 | 846440 | 846439 | 1691802 | 1675559 | 1675559 | 7384.17 | 1 | 20905 |
| 20COX5362NPS | 10/6/2020 | 871148 | 871150 | 1740820 | 1699322 | 1699322 | 6444.35 | 1 | 22578 |
| 20COX5363NPS | 10/7/2020 | 763820 | 763799 | 1526472 | 1447867 | 1447867 | 6470.72 | 1 | 17962 |
| 20COX5365NPS | 10/7/2020 | 1029585 | 1029593 | 2057592 | 2041739 | 2041739 | 7639.81 | 1 | 25677 |
| 20COX5366NPS | 10/7/2020 | 785656 | 785684 | 1570126 | 1562125 | 1562125 | 6669.41 | 2 | 18867 |
| 20COX5367NPS | 10/7/2020 | 832143 | 832132 | 1662950 | 1655007 | 1655007 | 6216.90 | 2 | 19974 |
| 20COX5368NPS | 10/6/2020 | 972630 | 972409 | 1942607 | 347450 | 347450 | 26568.95 | 1 | 64398 |
| 20COX5371NPS | 10/8/2020 | 681913 | 681934 | 1362577 | 1355054 | 1355054 | 5938.39 | 1 | 17151 |
| 20COX5374NPS | 10/8/2020 | 850384 | 850390 | 1699678 | 1694485 | 1694485 | 6406.91 | 1 | 21432 |
| 20COX5376NPS | 10/8/2020 | 921257 | 921250 | 1841106 | 1803136 | 1803136 | 7815.67 | 2 | 23393 |
| 20COX5377NPS | 10/8/2020 | 892077 | 892095 | 1782841 | 1769317 | 1769317 | 7857.70 | 2 | 22181 |
| 20COX5378NPS | 10/8/2020 | 779292 | 779320 | 1557604 | 1526934 | 1526934 | 6710.05 | 1 | 21242 |
| 20COX5380NPS | 10/8/2020 | 796987 | 796986 | 1592343 | 1576570 | 1576570 | 6072.42 | 1 | 20199 |
| 20COX5382NPS | 10/8/2020 | 952015 | 952130 | 1902170 | 1413488 | 1413488 | 66288.00 | 1 | 229178 |
| 20COX5383NPS | 10/8/2020 | 855069 | 855085 | 1708171 | 1700098 | 1700098 | 7705.85 | 1 | 22321 |
| 20COX5385NPS | 10/8/2020 | 849621 | 849619 | 1697893 | 1685187 | 1685187 | 7569.67 | 2 | 21790 |
| 20COX5386NPS | 10/8/2020 | 818054 | 818099 | 1634403 | 1568056 | 1568056 | 6845.15 | 1 | 19364 |
| 20COX5387NPS | 10/8/2020 | 800032 | 800038 | 1598539 | 1588972 | 1588972 | 7196.42 | 1 | 21231 |
| 20COX5390NPS | 10/8/2020 | 844660 | 844680 | 1687821 | 1675992 | 1675992 | 6419.96 | 1 | 20406 |
| 20COX5396NPS | 10/8/2020 | 1149413 | 1149143 | 2295271 | 1014286 | 1014286 | 59606.32 | 1 | 150955 |
| 20COX5399NPS | 10/9/2020 | 717197 | 717195 | 1433125 | 1379042 | 1379042 | 6180.51 | 1 | 18349 |
| 20COX5400NPS | 10/9/2020 | 1448308 | 1448890 | 2892909 | 1242780 | 1242780 | 34971.34 | 1 | 198512 |
| 20COX5401NPS | 10/9/2020 | 803582 | 803591 | 1605585 | 1582644 | 1582644 | 3592.70 | 1 | 19444 |
| 20COX5403NPS | 10/9/2020 | 864572 | 864583 | 1727614 | 1723322 | 1723322 | 6526.92 | 1 | 22918 |
| 20COX5404NPS | 10/9/2020 | 846789 | 846795 | 1692135 | 1683611 | 1683611 | 6873.10 | 1 | 20529 |
| 20COX5409NPS | 10/9/2020 | 793991 | 794018 | 1585704 | 1559310 | 1559310 | 6938.82 | 5 | 19687 |
| 20COX5410NPS | 10/9/2020 | 702963 | 702965 | 1404551 | 1373799 | 1373799 | 5903.45 | 1 | 16472 |
| 20COX5411NPS | 10/9/2020 | 783577 | 783580 | 1566145 | 1530381 | 1530381 | 6776.66 | 1 | 18316 |
| 20COX5412NPS | 10/9/2020 | 746460 | 746474 | 1491633 | 1481875 | 1481875 | NA | NA | NA |
| 20COX5413NPS | 10/9/2020 | 953132 | 953151 | 1904683 | 1886395 | 1886395 | 4568.02 | 1 | 24213 |
| 20COX5416NPS | 10/9/2020 | 856662 | 856671 | 1711556 | 1661743 | 1661743 | 5151.44 | 3 | 24079 |
| 20COX5419NPS | 10/9/2020 | 983323 | 983329 | 1965040 | 1941159 | 1941159 | 6189.09 | 1 | 24560 |
| 20COX5420NPS | 10/9/2020 | 1009697 | 1009730 | 2017477 | 1941773 | 1941773 | 8585.49 | 5 | 23275 |
| 20COX5421NPS | 10/9/2020 | 874145 | 874171 | 1746802 | 1720236 | 1720236 | 7758.25 | 1 | 20856 |
| 20COX5423NPS | 10/9/2020 | 665224 | 665222 | 1329623 | 1316390 | 1316390 | 2998.94 | 1 | 16787 |
| 20COX5424NPS | 10/9/2020 | 905008 | 905026 | 1807773 | 1628904 | 1628904 | 7352.75 | 1 | 21116 |
| 20COX5427NPS | 10/9/2020 | 908910 | 908924 | 1816386 | 1769477 | 1769477 | 7378.01 | 1 | 22540 |
| 20COX5428NPS | 10/9/2020 | 911042 | 911037 | 1820217 | 1794843 | 1794843 | NA | NA | NA |
| 20COX5431NPS | 10/9/2020 | 859352 | 859365 | 1717306 | 1707252 | 1707252 | 5416.29 | 1 | 21508 |
| 20COX5432NPS | 10/9/2020 | 862574 | 862583 | 1723668 | 1715648 | 1715648 | 7723.22 | 3 | 21669 |
| 20COX5436NPS | 10/9/2020 | 1088237 | 1088309 | 2173409 | 782570 | 782570 | 46765.25 | 1 | 123587 |
| 20COX5438NPS | 10/9/2020 | 868699 | 868704 | 1736166 | 1727133 | 1727133 | 7528.16 | 1 | 22126 |
| 20COX5440NPS | 10/9/2020 | 1275773 | 1276273 | 2549341 | 1989173 | 1989173 | 50566.43 | 1 | 137370 |
| 20COX5442NPS | 10/9/2020 | 740820 | 740832 | 1480615 | 1453663 | 1453663 | 6384.80 | 1 | 18716 |
| 20COX5444NPS | 10/10/2020 | 818126 | 818129 | 1634688 | 1576181 | 1576181 | 6700.70 | 2 | 18077 |
| 20COX5445NPS | 10/10/2020 | 856366 | 856380 | 1711088 | 1704030 | 1704030 | 7617.61 | 1 | 21618 |
| 20COX5446NPS | 10/10/2020 | 781663 | 781657 | 1562120 | 1555115 | 1555115 | 6894.12 | 1 | 18444 |
| 20COX5447NPS | 10/10/2020 | 938419 | 938433 | 1875527 | 1870276 | 1870276 | 8306.81 | 3 | 24216 |
| 20COX5448NPS | 10/9/2020 | 1256820 | 1257000 | 2510613 | 1330801 | 1330801 | 62050.46 | 1 | 247152 |
| 20COX5451NPS | 10/10/2020 | 761181 | 761186 | 1521059 | 1517203 | 1517203 | 6784.63 | 10 | 19060 |
| 20COX5452NPS | 10/10/2020 | 711183 | 711186 | 1421555 | 1419348 | 1419348 | 6329.33 | 1 | 18056 |
| 20COX5453NPS | 10/10/2020 | 804818 | 804810 | 1608507 | 1584237 | 1584237 | 7059.85 | 1 | 19765 |
| 20COX5454NPS | 10/10/2020 | 762284 | 762298 | 1523211 | 1506195 | 1506195 | 6777.54 | 1 | 18991 |
| 20COX5455NPS | 10/10/2020 | 761651 | 761647 | 1522111 | 1487429 | 1487429 | 6687.58 | 1 | 18597 |
| 20COX5458NPS | 10/10/2020 | 780632 | 780656 | 1560076 | 1549324 | 1549324 | 6818.56 | 1 | 16195 |
| 20COX5459NPS | 10/10/2020 | 872269 | 872273 | 1743241 | 1729825 | 1729825 | 7707.88 | 1 | 21904 |
| 20COX5460NPS | 10/10/2020 | 901885 | 901862 | 1802018 | 1781296 | 1781296 | 7970.15 | 1 | 22105 |
| 20COX5462NPS | 10/10/2020 | 902814 | 902815 | 1803757 | 1750151 | 1750151 | 7755.83 | 1 | 22636 |
| 20COX5464NPS | 10/10/2020 | 711405 | 711410 | 1421733 | 1369805 | 1369805 | 6041.57 | 1 | 18030 |
| 20COX5466NPS | 10/10/2020 | 814004 | 813989 | 1627001 | 1602193 | 1602193 | 7136.17 | 1 | 19603 |
| 20COX5469NPS | 10/10/2020 | 842620 | 842628 | 1683915 | 1675753 | 1675753 | 7422.63 | 1 | 20299 |
| 20COX5470NPS | 10/10/2020 | 830260 | 830279 | 1659460 | 1652609 | 1652609 | 7347.03 | 2 | 19906 |
| 20COX5471NPS | 10/10/2020 | 737575 | 737549 | 1474181 | 1449928 | 1449928 | 6521.36 | 2 | 18553 |
| 20COX5472NPS | 10/10/2020 | 831475 | 831477 | 1661865 | 1648308 | 1648308 | 7312.76 | 3 | 19595 |
| 20COX5473NPS | 10/10/2020 | 913571 | 913568 | 1825415 | 1788134 | 1788134 | 8033.76 | 9 | 21320 |
| 20COX5474NPS | 10/10/2020 | 855998 | 855998 | 1710505 | 1681565 | 1681565 | 7520.83 | 2 | 20190 |
| 20COX5478NPS | 10/10/2020 | 865434 | 865417 | 1729855 | 1677376 | 1677376 | 7509.84 | 1 | 20288 |
| 20COX5479NPS | 10/10/2020 | 1092541 | 1092546 | 2183265 | 2161811 | 2161811 | 9728.20 | 1 | 27863 |
| 20COX5480NPS | 10/10/2020 | 885996 | 885987 | 1770806 | 1739734 | 1739734 | 7863.86 | 1 | 22136 |
| 20COX5485NPS | 10/13/2020 | 813123 | 813144 | 1624864 | 1597037 | 1597037 | 7198.06 | 1 | 18858 |
| 20COX5487NPS | 10/14/2020 | 984512 | 984507 | 1967463 | 1953130 | 1953130 | 8828.35 | 1 | 24308 |
| 20COX5490NPS | 10/13/2020 | 918354 | 918357 | 1835557 | 1808509 | 1808509 | 8169.63 | 1 | 21655 |
| 20COX5491NPS | 10/13/2020 | 902297 | 902293 | 1803377 | 1786117 | 1786117 | 8042.95 | 1 | 23087 |
| 20COX5493NPS | 10/13/2020 | 963248 | 963231 | 1925316 | 1918935 | 1918935 | 8703.78 | 1 | 24844 |
| 20COX5494NPS | 10/13/2020 | 980026 | 980037 | 1958392 | 1945270 | 1945270 | 8816.38 | 1 | 24443 |
| 20COX5495NPS | 10/13/2020 | 930474 | 930480 | 1859532 | 1843701 | 1843701 | 8283.35 | 1 | 22030 |
| 20COX5496NPS | 10/13/2020 | 892760 | 892738 | 1784522 | 1755089 | 1755089 | 7881.06 | 2 | 21699 |
| 20COX5497NPS | 10/13/2020 | 899954 | 899943 | 1798212 | 1657611 | 1657611 | 7477.74 | 1 | 21183 |
| 20COX5498NPS | 10/13/2020 | 955181 | 955207 | 1908561 | 1899534 | 1899534 | 8445.77 | 4 | 23330 |
| 20COX5501NPS | 10/13/2020 | 956418 | 956419 | 1911509 | 1830883 | 1830883 | 8221.27 | 1 | 22178 |
| 20COX5502NPS | 10/13/2020 | 792956 | 792946 | 1584865 | 1573899 | 1573899 | 7116.70 | 2 | 19849 |
| 20COX5514NPS | 10/14/2020 | 836770 | 836778 | 1672333 | 1663677 | 1663677 | 7499.81 | 1 | 19946 |
| 20COX5520NPS | 10/14/2020 | 854859 | 854845 | 1708102 | 1697429 | 1697429 | 7669.03 | 1 | 21610 |
| 20COX5523NPS | 10/15/2020 | 870310 | 870331 | 1738649 | 1718468 | 1718468 | 7705.25 | 1 | 21614 |
| 20COX5524NPS | 10/15/2020 | 855365 | 855366 | 1707667 | 1685120 | 1685120 | 7658.39 | 1 | 21679 |
| 20COX5526NPS | 10/15/2020 | 848450 | 848445 | 1695328 | 1654270 | 1654270 | 7505.89 | 2 | 21211 |
| 20COX5528NPS | 10/15/2020 | 976969 | 976984 | 1952155 | 1939194 | 1939194 | 8852.20 | 2 | 24717 |
| 20COX5530NPS | 10/16/2020 | 1087813 | 1087820 | 2173697 | 2110476 | 2110476 | 9321.76 | 1 | 25223 |
| 20COX5532NPS | 10/15/2020 | 694988 | 694987 | 1388985 | 1357795 | 1357795 | 6089.43 | 2 | 17360 |
| 20COX5533NPS | 10/15/2020 | 4723607 | 4724325 | 9436050 | 8783865 | 8783865 | 38000.14 | 1 | 113851 |
| 20COX5534NPS | 10/16/2020 | 8330550 | 8331896 | 16639824 | 16522894 | 16522894 | 71265.13 | 1 | 196517 |
| 20COX5535NPS | 10/16/2020 | 8260471 | 8261483 | 16504839 | 15728505 | 15728505 | 67808.17 | 1 | 205114 |
| 20COX5536NPS | 10/16/2020 | 5058638 | 5059291 | 10108660 | 9894253 | 9894253 | 42939.63 | 1 | 125901 |
| 20COX5538NPS | 10/16/2020 | 6166631 | 6167385 | 12324198 | 11721131 | 11721131 | 50436.42 | 1 | 148762 |
| 20COX5540NPS | 10/16/2020 | 7711388 | 7712343 | 15406619 | 15031729 | 15031729 | 64556.26 | 1 | 183613 |
| 20COX5542NPS | 10/16/2020 | 4843823 | 4844587 | 9680513 | 9616485 | 9616485 | 41045.68 | 1 | 112023 |
| 20COX5543NPS | 10/16/2020 | 5192299 | 5193476 | 10374844 | 10334066 | 10334066 | 44755.76 | 1 | 122701 |
| 20COX5546NPS | 10/16/2020 | 7516557 | 7517228 | 15023716 | 14441611 | 14441611 | 61955.74 | 1 | 175551 |
| 20COX5555NPS | 10/16/2020 | 4764274 | 4765035 | 9518785 | 9233255 | 9233255 | 39650.29 | 1 | 129443 |
| 20COX5556NPS | 10/16/2020 | 3559428 | 3560016 | 7112393 | 7051916 | 7051916 | 30376.50 | 12 | 86460 |
| 20COX5557NPS | 10/16/2020 | 4272230 | 4272825 | 8538469 | 8492940 | 8492940 | 36167.69 | 1 | 98789 |
| 20COX5561NPS | 10/16/2020 | 3720405 | 3721037 | 7433868 | 7388471 | 7388471 | 31445.40 | 1 | 88472 |
| 20COX5564NPS | 10/16/2020 | 7067652 | 7068386 | 14127090 | 14059496 | 14059496 | 60627.84 | 1 | 170609 |
| 20COX5565NPS | 10/16/2020 | 6113754 | 6113680 | 12218787 | 11869218 | 11869218 | 51207.50 | 1 | 150568 |
| 20COX5568NPS | 10/16/2020 | 6846693 | 6847740 | 13684285 | 13221067 | 13221067 | 56406.32 | 2 | 155890 |
| 20COX5572NPS | 10/16/2020 | 5465820 | 5466533 | 10923020 | 10607783 | 10607783 | 45228.08 | 1 | 136998 |
| 20COX5577NPS | 10/16/2020 | 6548518 | 6549352 | 13082603 | 12986193 | 12986193 | 56693.02 | 1 | 170963 |
| 20COX5581NPS | 10/17/2020 | 11304489 | 11305774 | 22585389 | 21877472 | 21877472 | 93957.11 | 1 | 280008 |
| 20COX5582NPS | 10/17/2020 | 4139039 | 4139541 | 8269406 | 8218710 | 8218710 | 35844.26 | 1 | 95145 |
| 20COX5587NPS | 10/17/2020 | 6110603 | 6111403 | 12210425 | 11824762 | 11824762 | 50804.07 | 1 | 146161 |
| 20COX5588NPS | 10/17/2020 | 7197785 | 7198815 | 14381295 | 13799321 | 13799321 | 58595.53 | 1 | 168001 |
| 20COX5589NPS | 10/17/2020 | 7186718 | 7186580 | 14363752 | 13805782 | 13805782 | 59321.08 | 1 | 185743 |
| 20COX5591NPS | 10/17/2020 | 4831699 | 4832454 | 9655395 | 9439446 | 9439446 | 39649.03 | 1 | 111389 |
| 20COX5592NPS | 10/17/2020 | 6927906 | 6929082 | 13839368 | 13769464 | 13769464 | 58779.69 | 2 | 176135 |
| 20COX5593NPS | 10/17/2020 | 10615948 | 10617094 | 21217459 | 20392957 | 20392957 | 89726.92 | 1 | 262891 |
| 20COX5594NPS | 10/17/2020 | 7100140 | 7101281 | 14181846 | 13481037 | 13481037 | 57608.68 | 1 | 180830 |
| 20COX5596NPS | 10/17/2020 | 5795449 | 5796041 | 11582932 | 11312277 | 11312277 | 48335.34 | 11 | 140571 |
| 20COX5597NPS | 10/17/2020 | 6272805 | 6273242 | 12537979 | 12217705 | 12217705 | 51907.38 | 1 | 152548 |
| 20COX5598NPS | 10/17/2020 | 4818653 | 4819739 | 9630196 | 9506189 | 9506189 | 40343.92 | 12 | 122684 |
| 20COX5599NPS | 10/17/2020 | 5651307 | 5652001 | 11292476 | 10979830 | 10979830 | 47553.93 | 1 | 147859 |
| 20COX5600NPS | 10/17/2020 | 64869 | 208626 | 194223 | 191460 | 191460 | 747.86 | 2 | 2822 |
| 20COX5601NPS | 10/17/2020 | 59189 | 200097 | 196988 | 190835 | 190835 | 782.22 | 5 | 2625 |
| 20COX5603NPS | 10/19/2020 | 59239 | 110007 | 115517 | 114425 | 114425 | 439.93 | 1 | 1519 |
| 20COX5604NPS | 10/19/2020 | 59153 | 191643 | 182966 | 181818 | 181818 | 786.31 | 1 | 2700 |
| 20COX5606NPS | 10/19/2020 | 50820 | 318673 | 323744 | 307548 | 307548 | 979.16 | 1 | 4435 |
| 20COX5607NPS | 10/19/2020 | 107194 | 56425 | 109941 | 107775 | 107775 | 374.03 | 3 | 1418 |
| 20COX5609NPS | 10/19/2020 | 81736 | 217100 | 214076 | 206456 | 206456 | 676.72 | 1 | 3207 |
| 20COX5610NPS | 10/18/2020 | 28198 | 146656 | 109836 | 106100 | 106100 | 402.51 | 2 | 1684 |
| 20COX5611NPS | 10/19/2020 | 129686 | 183264 | 242267 | 240788 | 240788 | 649.77 | 1 | 3346 |
| 20COX5613NPS | 10/19/2020 | 93020 | 78923 | 92736 | 48933 | 48933 | 5778.52 | 3 | 7882 |
| 20COX5621NPS | 10/19/2020 | 53551 | 87370 | 56309 | 54358 | 54358 | 261.34 | 1 | 823 |
| 20COX5626NPS | 10/20/2020 | 78922 | 56363 | 81639 | 81197 | 81197 | 357.49 | 1 | 1031 |
| 20COX5627NPS | 10/20/2020 | 13494535 | 13496929 | 26970619 | 26554691 | 26554691 | 57220.83 | 1 | 328767 |
| 20COX5628NPS | 10/20/2020 | 6795725 | 6796448 | 13578501 | 13405077 | 13405077 | 29538.02 | 1 | 177306 |
| 20COX5629NPS | 10/20/2020 | 28147 | 6350145 | 6367330 | 6191275 | 6191275 | 15647.31 | 1 | 93913 |
| 20COX5630NPS | 10/20/2020 | 6104183 | 6105378 | 12196837 | 11712181 | 11712181 | 26049.99 | 1 | 159656 |
| 20COX5631NPS | 10/20/2020 | 7022547 | 7023499 | 14027861 | 12780424 | 12780424 | 28071.65 | 1 | 180751 |
| 20COX5632NPS | 10/20/2020 | 56384 | 211339 | 182900 | 173699 | 173699 | 851.50 | 1 | 2593 |
| 20COX5633NPS | 10/20/2020 | 6740014 | 11280 | 6739357 | 6271763 | 6271763 | 16086.97 | 1 | 95399 |
| 20COX5636NPS | 10/20/2020 | 47888 | 93003 | 64749 | 59137 | 59137 | 295.06 | 1 | 864 |
| 20COX5638NPS | 10/20/2020 | 6903531 | 6904795 | 13798004 | 13716257 | 13716257 | 59160.06 | 1 | 167621 |
| 20COX5639NPS | 10/20/2020 | 13481810 | 5632 | 13481164 | 13329928 | 13329928 | 33980.19 | 1 | 207867 |
| 20COX5640NPS | 10/20/2020 | 90165 | 56331 | 104141 | 102097 | 102097 | 295.02 | 1 | 1304 |
| 20COX5643NPS | 10/21/2020 | 73268 | 87347 | 90141 | 89252 | 89252 | 391.44 | 2 | 1244 |
| 20COX5644NPS | 10/21/2020 | 47918 | 247980 | 211029 | 209640 | 209640 | 612.84 | 1 | 3295 |
| 20COX5645NPS | 10/21/2020 | 166081 | 53487 | 180087 | 178689 | 178689 | 531.94 | 1 | 2521 |
| 20COX5646NPS | 10/21/2020 | 87339 | 205681 | 208324 | 206458 | 206458 | 977.02 | 2 | 3056 |
| 20COX5647NPS | 10/21/2020 | 242334 | 33811 | 202867 | 196945 | 196945 | 655.39 | 1 | 3030 |
| 20COX5649NPS | 10/21/2020 | 9106602 | 22548 | 9106248 | 9021308 | 9021308 | 22656.42 | 1 | 145738 |
| 20COX5650NPS | 10/21/2020 | 5847784 | 5848606 | 11673974 | 11533286 | 11533286 | 25591.53 | 1 | 153047 |
| 20COX5652NPS | 10/21/2020 | 5167964 | 5169308 | 10315419 | 10130432 | 10130432 | 44258.77 | 1 | 137467 |
| 20COX5653NPS | 10/21/2020 | 8656727 | 8658322 | 17291312 | 16982296 | 16982296 | 74436.99 | 1 | 227384 |
| 20COX5654NPS | 10/21/2020 | 6584070 | 6585084 | 13153004 | 12909436 | 12909436 | 56742.74 | 1 | 169299 |
| 20COX5655NPS | 10/21/2020 | 6655308 | 6656168 | 13299841 | 13229472 | 13229472 | 57633.97 | 1 | 173081 |
| 20COX5656NPS | 10/21/2020 | 30987 | 67581 | 44997 | 44673 | 44673 | 222.97 | 1 | 606 |
| 20COX5657NPS | 10/21/2020 | 247831 | 22536 | 205583 | 205478 | 205478 | 1026.56 | 6 | 2526 |
| 20COX5663NPS | 10/22/2020 | 28177 | 36642 | 53482 | 52865 | 52865 | 237.72 | 1 | 612 |
| 20COX5664NPS | 10/22/2020 | 11138780 | 11274 | 11138350 | 11097003 | 11097003 | 55330.31 | 1 | 146943 |
| 20COX5668NPS | 10/22/2020 | 217061 | 2815 | 180404 | 179491 | 179491 | 895.29 | 2 | 2752 |
| 20COX5671NPS | 10/22/2020 | 208451 | 10239233 | 10390027 | 10231873 | 10231873 | 50626.92 | 1 | 138398 |
| 20COX5672NPS | 10/22/2020 | 9582637 | 9584011 | 19149851 | 19001571 | 19001571 | 82450.01 | 1 | 229604 |
| 20COX5673NPS | 10/22/2020 | 10479466 | 10481058 | 20942227 | 20740219 | 20740219 | 89623.50 | 1 | 249288 |
| 20COX5674NPS | 10/22/2020 | 9166945 | 9167551 | 18319691 | 18040745 | 18040745 | 78909.14 | 1 | 213198 |
| 20COX5675NPS | 10/22/2020 | 8459 | 11278 | 14079 | 13645 | 13645 | 64.58 | 1 | 188 |
| 20COX5676NPS | 10/22/2020 | 22499 | 2817 | 22499 | 21617 | 21617 | 107.28 | 1 | 303 |
| 20COX5681NPS | 10/23/2020 | 9488273 | 9489718 | 18959059 | 18878780 | 18878780 | 81379.87 | 1 | 216840 |
| 20COX5682NPS | 10/22/2020 | 61953 | 5652 | 30987 | 30469 | 30469 | 151.22 | 1 | 441 |
| 20COX5683NPS | 10/23/2020 | 10455087 | 10455849 | 20897776 | 20618575 | 20618575 | 89223.91 | 1 | 255830 |
| 20COX5686NPS | 10/22/2020 | 169119 | 124012 | 222537 | 221807 | 221807 | 976.50 | 1 | 2482 |
| 20COX5687NPS | 10/23/2020 | 9849135 | 9850341 | 19682622 | 19403869 | 19403869 | 83603.00 | 1 | 242429 |
| 20COX5689NPS | 10/22/2020 | 10359776 | 10361341 | 20698318 | 20100314 | 20100314 | 88509.15 | 1 | 259343 |
| 20COX5691NPS | 10/23/2020 | 9567507 | 9568840 | 19114608 | 19031153 | 19031153 | 83364.63 | 1 | 228848 |
| 20COX5692NPS | 10/23/2020 | 11264341 | 11265903 | 22505668 | 22435680 | 22435680 | 98282.21 | 2 | 268536 |
| 20COX5695NPS | 10/23/2020 | 47916 | 166300 | 171709 | 170390 | 170390 | 832.42 | 3 | 2231 |
| 20COX5696NPS | 10/23/2020 | 10105209 | 10107950 | 20190417 | 20057392 | 20057392 | 86275.79 | 1 | 227097 |
| 20COX5701NPS | 10/23/2020 | 81713 | 126814 | 126692 | 124781 | 124781 | 571.08 | 11 | 1638 |
| 20COX5703NPS | 10/22/2020 | 11022076 | 11023735 | 22023466 | 21952307 | 21952307 | 95985.18 | 1 | 268184 |
| 20COX5704NPS | 10/23/2020 | 163330 | 2817 | 135171 | 134951 | 134951 | 672.38 | 1 | 1929 |
| 20COX5708NPS | 10/23/2020 | 2811 | 10141730 | 10122574 | 10067097 | 10067097 | 50221.71 | 1 | 143446 |
| 20COX5710NPS | 10/23/2020 | 9962996 | 9964425 | 19906932 | 19181012 | 19181012 | 82769.77 | 1 | 212427 |
| 20COX5714NPS | 10/23/2020 | 59395 | 38317 | 61199 | 55865 | 55865 | 326.30 | 2 | 808 |
| 20COX5715NPS | 10/23/2020 | 51807 | 76722 | 82318 | 81686 | 81686 | 522.91 | 4 | 1593 |
| 20COX5716NPS | 10/23/2020 | 52254 | 2633181 | 2676857 | 2614110 | 2614110 | 20710.02 | 1 | 66449 |
| 20COX5717NPS | 10/23/2020 | 117342 | 23117 | 111577 | 111382 | 111382 | 793.88 | 3 | 2296 |
| 20COX5718NPS | 10/23/2020 | 63349 | 7700 | 32641 | 32581 | 32581 | 264.57 | 4 | 761 |
| 20COX5723NPS | 10/24/2020 | 16351 | 123424 | 92935 | 92399 | 92399 | 586.43 | 3 | 1946 |
| 20COX5725NPS | 10/24/2020 | 2535309 | 2535880 | 5064728 | 4992815 | 4992815 | 26503.95 | 2 | 75505 |
| 20COX5726NPS | 10/24/2020 | 11425 | 85617 | 77854 | 77548 | 77548 | 637.38 | 1 | 1816 |
| 20COX5729NPS | 10/24/2020 | 19157 | 65215 | 55510 | 55282 | 55282 | 336.57 | 1 | 1366 |
| 20COX5730NPS | 10/24/2020 | 13482 | 96163 | 76790 | 76474 | 76474 | 621.19 | 1 | 1873 |
| 20COX5731NPS | 10/24/2020 | 41957 | 1916 | 36233 | 36005 | 36005 | 295.17 | 4 | 848 |
| 20COX5732NPS | 10/24/2020 | 131788 | 3876 | 106548 | 106473 | 106473 | 522.01 | 1 | 2497 |
| 20COX5734NPS | 10/24/2020 | 20975 | 183075 | 157851 | 157080 | 157080 | 1288.06 | 2 | 3716 |
| 20COX5735NPS | 10/24/2020 | 28612 | 2272287 | 2266059 | 2252418 | 2252418 | 18413.17 | 1 | 53635 |
| 20COX5737NPS | 10/24/2020 | 2755707 | 2756078 | 5506140 | 5495305 | 5495305 | 29888.56 | 1 | 74982 |
| 20COX5738NPS | 10/24/2020 | 11565 | 28945 | 32726 | 32279 | 32279 | 239.91 | 1 | 733 |
| 20COX5740NPS | 10/24/2020 | 1905 | 1252431 | 1249355 | 1232457 | 1232457 | 10076.49 | 4 | 28144 |
| 20COX5741NPS | 10/24/2020 | 2378499 | 0 | 2378285 | 2358247 | 2358247 | 19086.70 | 1 | 59654 |
| 20COX5742NPS | 10/24/2020 | 2283591 | 2283919 | 4562725 | 4553303 | 4553303 | 24779.98 | 1 | 68324 |
| 20COX5743NPS | 10/24/2020 | 1247463 | 1247635 | 2491559 | 2484853 | 2484853 | 14439.47 | 1 | 38926 |
| 20COX5744NPS | 10/24/2020 | 9456 | 126830 | 107567 | 107076 | 107076 | 885.86 | 1 | 2410 |
| 20COX5745NPS | 10/24/2020 | 1083011 | 1083146 | 2163571 | 2154712 | 2154712 | 11956.65 | 1 | 29935 |
| 20COX5746NPS | 10/24/2020 | 2846023 | 2846302 | 5686716 | 5674643 | 5674643 | 29206.29 | 1 | 83420 |
| 20COX5747NPS | 10/24/2020 | 2371585 | 2371853 | 4734861 | 4718537 | 4718537 | 25818.86 | 1 | 72956 |
| 20COX5749NPS | 10/26/2020 | 2150270 | 2150465 | 4295141 | 4286240 | 4286240 | 23217.65 | 1 | 65467 |
| 20COX5750NPS | 10/26/2020 | 5767 | 73008 | 69049 | 67885 | 67885 | 551.37 | 2 | 1639 |
| 20COX5757NPS | 10/26/2020 | 7686 | 1812709 | 1813442 | 1787247 | 1787247 | 14465.89 | 1 | 44322 |
| 20COX5761NPS | 10/27/2020 | 1771926 | 1772149 | 3539227 | 3428104 | 3428104 | 20604.02 | 1 | 58950 |
| 20COX5764NPS | 10/27/2020 | 182861 | 13187 | 152668 | 152070 | 152070 | 1264.97 | 2 | 3564 |
| 20COX5767NPS | 10/27/2020 | 182125 | 34868 | 183986 | 183059 | 183059 | 1303.59 | 1 | 3595 |
| 20COX5768NPS | 10/27/2020 | 116003 | 1943 | 86993 | 86169 | 86169 | 695.50 | 2 | 2036 |
| 20COX5771NPS | 10/27/2020 | 3893 | 62208 | 58229 | 57093 | 57093 | 457.39 | 1 | 1424 |
| 20COX5773NPS | 10/27/2020 | 3842 | 19194 | 19152 | 19040 | 19040 | 155.05 | 1 | 453 |
| 20COX5775NPS | 10/27/2020 | 1578335 | 17678 | 1578192 | 1567147 | 1567147 | 12371.14 | 5 | 39116 |
| 20COX5776NPS | 10/27/2020 | 0 | 76183 | 58506 | 58296 | 58296 | 464.73 | 1 | 1378 |
| 20COX5783NPS | 10/27/2020 | 84399 | 209082 | 231574 | 230652 | 230652 | 1603.09 | 2 | 4544 |
| 20COX5784NPS | 10/28/2020 | 5849 | 54468 | 31043 | 30822 | 30822 | 245.40 | 1 | 668 |
| 20COX5785NPS | 10/28/2020 | 5658 | 97997 | 71345 | 70786 | 70786 | 586.57 | 1 | 1508 |
| 20COX5786NPS | 10/27/2020 | 728563 | 3796 | 728499 | 728003 | 728003 | 5986.71 | 26 | 14621 |
| 20COX5787NPS | 10/27/2020 | 2047349 | 2047494 | 4090668 | 4077032 | 4077032 | 23389.06 | 1 | 70125 |
| 20COX5788NPS | 10/27/2020 | 31364 | 2069 | 31362 | 30868 | 30868 | 206.43 | 1 | 615 |
| 20COX5789NPS | 10/28/2020 | 67058 | 28707 | 70839 | 70392 | 70392 | 535.37 | 1 | 1404 |
| 20COX5790NPS | 10/28/2020 | 15227 | 5712 | 15227 | 15195 | 15195 | 124.72 | 1 | 335 |
| 20COX5794NPS | 10/28/2020 | 142261 | 830415 | 938695 | 934370 | 934370 | 6773.23 | 1 | 17477 |
| 20COX5796NPS | 10/28/2020 | 143996 | 55636 | 145756 | 145255 | 145255 | 1054.45 | 5 | 3132 |
| 20COX5797NPS | 10/28/2020 | 64661 | 89333 | 93043 | 92373 | 92373 | 601.11 | 2 | 1449 |
| 20COX5801NPS | 10/28/2020 | 926652 | 926910 | 1850110 | 1834772 | 1834772 | 3230.56 | 7 | 8905 |
| 20COX5803NPS | 10/28/2020 | 601492 | 601566 | 1201546 | 1190692 | 1190692 | 6195.50 | 2 | 15308 |
| 20COX5805NPS | 10/28/2020 | 1115470 | 1115742 | 2227722 | 2186977 | 2186977 | 4803.37 | 15 | 12999 |
| 20COX5807NPS | 10/30/2020 | 436504 | 436568 | 871699 | 860366 | 860366 | 5023.42 | 1 | 13955 |
| 20COX5813NPS | 10/30/2020 | 2043959 | 2044390 | 4083396 | 4065362 | 4065362 | 3045.48 | 3 | 8222 |
| 20COX5814NPS | 10/30/2020 | 2397744 | 2392310 | 4789991 | 4745468 | 4745468 | 889.52 | 3 | 2494 |
| 20COX5818NPS | 10/30/2020 | 894338 | 894485 | 1786559 | 1752604 | 1752604 | NA | NA | NA |
| 20COX5825NPS | 10/31/2020 | 9322423 | 9323771 | 18628479 | 18572947 | 18572947 | 40967.60 | 1 | 222375 |
| 20COX5826NPS | 10/30/2020 | 6933544 | 6934399 | 13856724 | 13819193 | 13819193 | 30494.86 | 1 | 141396 |
| 20COX5827NPS | 10/31/2020 | 8716897 | 8718415 | 17415464 | 17348825 | 17348825 | 38144.94 | 1 | 199423 |
| 20COX5828NPS | 10/31/2020 | 8518978 | 8520570 | 17011718 | 16875261 | 16875261 | 72648.86 | 1 | 198871 |
| 20COX5829NPS | 10/31/2020 | 1644385 | 1644563 | 3284721 | 3275773 | 3275773 | 1859.15 | 1 | 4909 |
| 20COX5831NPS | 10/31/2020 | 160635 | 53539 | 160549 | 160081 | 160081 | 736.54 | 1 | 2073 |
| 20COX5832NPS | 10/31/2020 | 253690 | 259315 | 433759 | 432247 | 432247 | 1895.49 | 4 | 5141 |
| 20COX5834NPS | 10/30/2020 | 228190 | 109870 | 247822 | 243943 | 243943 | 1125.02 | 2 | 3199 |
| 20COX5835NPS | 10/31/2020 | 163470 | 76037 | 166149 | 165629 | 165629 | 766.46 | 3 | 2207 |
| 20COX5836NPS | 10/31/2020 | 234049 | 225590 | 374855 | 373282 | 373282 | 1619.73 | 1 | 4421 |
| 20COX5841NPS | 10/30/2020 | 183211 | 56371 | 188671 | 187930 | 187930 | 873.64 | 1 | 2298 |
| 20COX5842NPS | 10/31/2020 | 180438 | 129704 | 228162 | 227181 | 227181 | 1004.95 | 1 | 2686 |
| 20COX5843NPS | 10/31/2020 | 205634 | 67578 | 205520 | 205097 | 205097 | 965.98 | 2 | 2355 |
| 20COX5847NPS | 10/31/2020 | 374795 | 185968 | 481662 | 480532 | 480532 | 2219.76 | 1 | 6682 |
| 20COX5848NPS | 10/31/2020 | 166469 | 64881 | 166397 | 165881 | 165881 | 754.89 | 5 | 2170 |
| 20COX5849NPS | 10/31/2020 | 157901 | 67735 | 156823 | 153462 | 153462 | 699.82 | 3 | 1945 |
| 20COX5854NPS | 10/31/2020 | 191644 | 70447 | 191614 | 191236 | 191236 | 883.49 | 5 | 2604 |
| 20COX5855NPS | 11/1/2020 | 8378253 | 8378628 | 16744523 | 16593472 | 16593472 | 71377.13 | 15 | 208266 |
| 20COX5856NPS | 11/1/2020 | 177591 | 115553 | 211242 | 210724 | 210724 | 942.62 | 1 | 2272 |
| 20COX5858NPS | 11/1/2020 | 197286 | 109929 | 219722 | 218202 | 218202 | 1004.71 | 1 | 3257 |
| 20COX5860NPS | 11/1/2020 | 979660 | 979732 | 1956734 | 1951962 | 1951962 | 12299.12 | 4 | 32574 |
| 20COX5861NPS | 11/1/2020 | 9127543 | 9128179 | 18244880 | 18061677 | 18061677 | 38957.36 | 1 | 210008 |
| 20COX5882NPS | 11/2/2020 | 185930 | 76048 | 185846 | 184436 | 184436 | 857.29 | 5 | 2416 |
| 20COX5963NPS | 11/3/2020 | 1607159 | 1607527 | 3209719 | 3199010 | 3199010 | 6978.55 | 1 | 20031 |
| 20COX5964NPS | 11/3/2020 | 330048 | 330088 | 658068 | 592500 | 592500 | 3584.49 | 1 | 9829 |
| 20COX5967NPS | 11/3/2020 | 266166 | 266250 | 531557 | 511268 | 511268 | 1214.17 | 1 | 3419 |
| 20COX5968NPS | 11/3/2020 | 919181 | 919489 | 1835389 | 1592419 | 1592419 | 7719.14 | 2 | 21720 |
| 20COX5971NPS | 11/3/2020 | 1662264 | 1662373 | 3321178 | 3239894 | 3239894 | 16160.06 | 1 | 47392 |
| 20COX5972NPS | 11/3/2020 | 2076178 | 2076208 | 4148471 | 3954237 | 3954237 | 19883.19 | 1 | 60598 |
| 20COX5973NPS | 11/3/2020 | 1511500 | 1511734 | 3019884 | 2897129 | 2897129 | 14985.07 | 2 | 44312 |
| 20COX5975NPS | 11/2/2020 | 1375613 | 1375724 | 2749058 | 2634281 | 2634281 | 13147.24 | 1 | 38638 |
| 20COX5977NPS | 11/3/2020 | 1100681 | 1100798 | 2199830 | 2161819 | 2161819 | 11541.41 | 1 | 32242 |
| 20COX5981NPS | 11/3/2020 | 1127701 | 1127583 | 2253362 | 2241871 | 2241871 | 11221.87 | 1 | 32212 |
| 20COX5982NPS | 11/3/2020 | 1297814 | 1297949 | 2593082 | 2575344 | 2575344 | 13200.03 | 1 | 38366 |
| 20COX5983NPS | 11/3/2020 | 1447501 | 1447627 | 2892369 | 2875344 | 2875344 | 14783.44 | 1 | 43865 |
| 20COX5984NPS | 11/3/2020 | 1258513 | 1258572 | 2514988 | 2450878 | 2450878 | 12278.27 | 1 | 36608 |
| 20COX5985NPS | 11/3/2020 | 1445564 | 1445706 | 2888524 | 2818809 | 2818809 | 13413.18 | 1 | 40299 |
| 20COX6001NPS | 11/6/2020 | 1722604 | 1723069 | 3443289 | 3414138 | 3414138 | 11794.15 | 2 | 35587 |
| 20COX6003NPS | 11/6/2020 | 1561251 | 1561368 | 3118182 | 3044635 | 3044635 | 16976.51 | 1 | 46891 |
| 20COX6004NPS | 11/6/2020 | 1308623 | 1308731 | 2615530 | 2591412 | 2591412 | 13922.89 | 1 | 39430 |
| 20COX6005NPS | 11/6/2020 | 1525671 | 1525791 | 3048633 | 3042743 | 3042743 | 16224.95 | 1 | 44866 |
| 20COX6006NPS | 11/6/2020 | 1381888 | 1382018 | 2761250 | 2741330 | 2741330 | 14564.19 | 1 | 39659 |
| 20COX6007NPS | 11/6/2020 | 1378998 | 1379235 | 2755480 | 2744964 | 2744964 | 14616.49 | 1 | 41003 |
| 20COX6009NPS | 11/6/2020 | 1481705 | 1481946 | 2961287 | 2911507 | 2911507 | 14648.24 | 4 | 43721 |
| 20COX6010NPS | 11/6/2020 | 247144 | 247441 | 494188 | 492265 | 492265 | 1374.00 | 10 | 6214 |
| 20COX6011NPS | 11/5/2020 | 3060700 | 3060952 | 6116190 | 6025449 | 6025449 | 31077.76 | 1 | 88551 |
| 20COX6013NPS | 11/6/2020 | 1057963 | 1058045 | 2114574 | 2088647 | 2088647 | 10876.23 | 1 | 32040 |
| 20COX6017NPS | 11/6/2020 | 1065921 | 1065886 | 2130283 | 2126309 | 2126309 | 10896.39 | 2 | 30998 |
| 20COX6018NPS | 11/6/2020 | 1340309 | 1340378 | 2678216 | 2659703 | 2659703 | 14295.79 | 3 | 40044 |
| 20COX6019NPS | 11/6/2020 | 1423972 | 1424192 | 2844998 | 2805619 | 2805619 | 14032.31 | 2 | 43871 |
| 20COX6020NPS | 11/6/2020 | 1245183 | 1245397 | 2488397 | 2447082 | 2447082 | 12705.98 | 1 | 37887 |
| 20COX6023NPS | 11/6/2020 | 1044023 | 1043997 | 2085925 | 2053192 | 2053192 | 11742.93 | 1 | 32150 |
| 20COX6025NPS | 11/6/2020 | 1197879 | 1197823 | 2393762 | 2384908 | 2384908 | 13561.13 | 1 | 38329 |
| 20COX6026NPS | 11/6/2020 | 935060 | 934995 | 1868450 | 1862840 | 1862840 | 10969.30 | 1 | 29044 |
| 20COX6029NPS | 11/6/2020 | 1348212 | 1348379 | 2694221 | 2671263 | 2671263 | 14984.33 | 4 | 41379 |
| 20COX6031NPS | 11/6/2020 | 1078307 | 1078371 | 2154044 | 2109308 | 2109308 | 12107.87 | 1 | 35467 |
| 20COX6035NPS | 11/7/2020 | 1731253 | 1731468 | 3459807 | 3401331 | 3401331 | 15532.85 | 1 | 45724 |
| 20COX6036NPS | 11/7/2020 | 1315461 | 1315498 | 2627707 | 2609141 | 2609141 | 14183.04 | 4 | 39727 |
| 20COX6037NPS | 11/7/2020 | 1458548 | 1458694 | 2911491 | 2878319 | 2878319 | 16646.97 | 1 | 42402 |
| 20COX6041NPS | 11/7/2020 | 1467334 | 1467441 | 2931059 | 2912663 | 2912663 | 16436.86 | 1 | 44065 |
| 20COX6042NPS | 11/7/2020 | 1055186 | 1055097 | 2108952 | 2099920 | 2099920 | 11870.21 | 4 | 31532 |
| 20COX6043NPS | 11/6/2020 | 1256108 | 1256005 | 2509884 | 2488485 | 2488485 | 12116.28 | 4 | 35174 |
| 20COX6044NPS | 11/7/2020 | 1289247 | 1289290 | 2576303 | 2555445 | 2555445 | 12153.11 | 1 | 36975 |
| 20COX6045NPS | 11/7/2020 | 1481226 | 1481402 | 2959910 | 2944916 | 2944916 | 14921.91 | 6 | 43955 |
| 20COX6046NPS | 11/7/2020 | 1378470 | 1378571 | 2754929 | 2737997 | 2737997 | 13950.09 | 1 | 38816 |
| 20COX6048NPS | 11/7/2020 | 1482151 | 1482123 | 2960055 | 2708626 | 2708626 | 16494.65 | 1 | 44252 |
| 20COX6051NPS | 11/7/2020 | 1382451 | 1382549 | 2762398 | 2749365 | 2749365 | 14789.00 | 5 | 42483 |
| 20COX6052NPS | 11/7/2020 | 1321027 | 1321131 | 2638766 | 2586459 | 2586459 | 13835.33 | 1 | 37207 |
| 20COX6053NPS | 11/7/2020 | 1287013 | 1287290 | 2571534 | 2565198 | 2565198 | 12491.18 | 3 | 35784 |
| 20COX6056NPS | 11/7/2020 | 1098602 | 1098606 | 2195266 | 2177051 | 2177051 | 10937.83 | 34 | 30390 |
| 20COX6057NPS | 11/7/2020 | 1577388 | 1577348 | 3152237 | 3147153 | 3147153 | 16563.63 | 4 | 45491 |
| 20COX6058NPS | 11/7/2020 | 1433839 | 1434039 | 2863575 | 2853362 | 2853362 | 16251.05 | 2 | 44936 |
| 20COX6059NPS | 11/7/2020 | 1207403 | 1215286 | 2389867 | 2340196 | 2340196 | 13186.43 | 1 | 34908 |
| 20COX6062NPS | 11/7/2020 | 1378474 | 1378659 | 2754766 | 2750460 | 2750460 | 15432.40 | 1 | 41599 |
| 20COX6065NPS | 11/7/2020 | 1253388 | 1253782 | 2502358 | 2490700 | 2490700 | 13086.46 | 1 | 36064 |
| 20COX6066NPS | 11/7/2020 | 1208880 | 1208993 | 2415070 | 2397226 | 2397226 | 13092.67 | 4 | 35208 |
| 20COX6068NPS | 11/6/2020 | 967212 | 967163 | 1932792 | 1928866 | 1928866 | 9788.16 | 2 | 27963 |
| 20COX6069NPS | 11/6/2020 | 1258477 | 1259094 | 2507120 | 2453438 | 2453438 | 13229.20 | 2 | 36259 |
| 20COX6070NPS | 11/6/2020 | 1459149 | 1459164 | 2915573 | 2865574 | 2865574 | 15047.91 | 1 | 38450 |
| 20COX6071NPS | 11/6/2020 | 1038108 | 1038223 | 2074171 | 2070714 | 2070714 | 11745.46 | 44 | 31144 |
| 20COX6072NPS | 11/6/2020 | 1091124 | 1091291 | 2178987 | 2138149 | 2138149 | 12245.42 | 1 | 34360 |
| 20COX6073NPS | 11/6/2020 | 1271308 | 1271505 | 2540354 | 2533622 | 2533622 | 14386.51 | 1 | 38902 |
| 20COX6074NPS | 11/6/2020 | 1424672 | 1425000 | 2845709 | 2838421 | 2838421 | 15300.23 | 4 | 42700 |
| 20COX6075NPS | 11/6/2020 | 1294752 | 1295186 | 2587137 | 2576399 | 2576399 | 13277.90 | 8 | 37087 |
| 20COX6078NPS | 11/6/2020 | 1197033 | 1197132 | 2391206 | 2386076 | 2386076 | 13993.65 | 2 | 37528 |
| 20COX6080NPS | 11/6/2020 | 14090648 | 14115026 | 28168728 | 28031746 | 28031746 | 94677.94 | 1 | 283388 |
| 20COX6116NPS | 11/15/2020 | 1157078 | 1157190 | 2311662 | 15850 | 15850 | 126.96 | 1 | 746 |
| 20COX6119NPS | 11/16/2020 | 1043863 | 1043943 | 2084936 | 1970578 | 1970578 | 10659.67 | 1 | 28893 |
| 20COX6121NPS | 11/16/2020 | 1294618 | 1294744 | 2586058 | 2503594 | 2503594 | NA | NA | NA |
| 20COX6124NPS | 11/16/2020 | 1232450 | 1232582 | 2463035 | 2330554 | 2330554 | 9896.10 | 2 | 28862 |
| 20COX6125NPS | 11/16/2020 | 1236482 | 1236553 | 2471451 | 2441540 | 2441540 | 10056.97 | 1 | 31566 |
| 20COX6126NPS | 11/16/2020 | 1678616 | 1678909 | 3354986 | 3341947 | 3341947 | 7148.49 | 1 | 22349 |
| 20COX6129NPS | 11/16/2020 | 2063543 | 2064971 | 4121111 | 7522 | 7522 | 23.37 | 1 | 84 |
| 20COX6130NPS | 11/16/2020 | 1279709 | 1280449 | 2555978 | 29869 | 29869 | 85.07 | 1 | 309 |
| 20COX6131NPS | 11/16/2020 | 1354937 | 1355043 | 2707032 | 2695791 | 2695791 | 10375.34 | 2 | 28700 |
| 20COX6133NPS | 11/16/2020 | 1481163 | 1481155 | 2959820 | 2943767 | 2943767 | 288.19 | 1 | 766 |
| 20COX6134NPS | 11/16/2020 | 1447198 | 1447488 | 2893117 | 2877061 | 2877061 | 9855.05 | 1 | 37135 |
| 20COX6135NPS | 11/15/2020 | 1242482 | 1242544 | 2483829 | 2473219 | 2473219 | 533.40 | 2 | 1665 |
| 20COX6139NPS | 11/16/2020 | 1484236 | 1484385 | 2965756 | 2789308 | 2789308 | 12159.16 | 1 | 35615 |
| 20COX6142NPS | 11/16/2020 | 1555753 | 1555906 | 3109414 | 3088846 | 3088846 | NA | NA | NA |
| 20COX6143NPS | 11/16/2020 | 1499047 | 1499209 | 2995604 | 2978517 | 2978517 | 10091.36 | 1 | 29321 |
| 20COX6146NPS | 11/16/2020 | 1535890 | 1535969 | 3069419 | 3062058 | 3062058 | 12580.80 | 1 | 34905 |
| 20COX6147NPS | 11/16/2020 | 1509401 | 1509303 | 3016798 | 2991962 | 2991962 | 12454.79 | 1 | 33467 |
| 20COX6148NPS | 11/16/2020 | 1157729 | 1157799 | 2312750 | 2246317 | 2246317 | 9459.53 | 2 | 26241 |
| 20COX6154NPS | 11/16/2020 | 1779179 | 1779439 | 3555273 | 3534559 | 3534559 | 8165.63 | 1 | 24733 |
| 20COX6160NPS | 11/18/2020 | 1463757 | 1463838 | 2925186 | 2606056 | 2606056 | 10948.40 | 1 | 35023 |
| 20COX6162NPS | 11/18/2020 | 2530024 | 2530702 | 5050160 | 47222 | 47222 | 190.72 | 2 | 642 |
| 20COX6164NPS | 11/18/2020 | 3486047 | 3487615 | 6963747 | 5221841 | 5221841 | 6982.95 | 27 | 71419 |
| 20COX6165NPS | 11/18/2020 | 8617825 | 8619559 | 17218791 | 17087986 | 17087986 | 73464.67 | 1 | 195328 |
| 20COX6169NPS | 11/18/2020 | 6654466 | 6654552 | 13300713 | 13260411 | 13260411 | 57350.90 | 2 | 158984 |
| 20COX6173NPS | 11/18/2020 | 2846559 | 2847013 | 5690026 | 3196443 | 3196443 | 4689.92 | 2 | 16651 |
| 20COX6174NPS | 11/18/2020 | 1333520 | 1333616 | 2663523 | 2301586 | 2301586 | 10162.22 | 2 | 27772 |
| 20COX6175NPS | 11/18/2020 | 7798972 | 7798818 | 15590332 | 15287242 | 15287242 | 65464.43 | 1 | 184355 |
| 20COX6176NPS | 11/18/2020 | 8095254 | 8097082 | 16167261 | 16003677 | 16003677 | 68532.72 | 1 | 165848 |
| 20COX6178NPS | 11/18/2020 | 1503676 | 1503653 | 3004367 | 2805983 | 2805983 | 11598.39 | 4 | 33461 |
| 20COX6181NPS | 11/18/2020 | 1817223 | 1817324 | 3629368 | 3591337 | 3591337 | 10454.58 | 1 | 28103 |
| 20COX6183NPS | 11/17/2020 | 640811 | 641233 | 1280110 | 1149130 | 1149130 | 37810.28 | 1 | 195189 |
| 20COX6220NPS | 11/19/2020 | 9364636 | 9366227 | 18714876 | 18406380 | 18406380 | 77805.46 | 1 | 223774 |
| 20COX6221NPS | 11/19/2020 | 9221255 | 9222331 | 18427436 | 18067163 | 18067163 | 76765.30 | 1 | 211065 |
| 20COX6223NPS | 11/19/2020 | 11477137 | 11491440 | 22944476 | 22483111 | 22483111 | 72511.18 | 1 | 279579 |
| 20COX6225NPS | 11/19/2020 | 8702669 | 8702662 | 17395300 | 17092064 | 17092064 | 72497.86 | 1 | 198215 |
| 20COX6227NPS | 11/19/2020 | 10158423 | 10159919 | 20297687 | 19965337 | 19965337 | 85021.66 | 1 | 235598 |
| 20COX6230NPS | 11/19/2020 | 1141628 | 1141694 | 2281461 | 2165385 | 2165385 | 9588.63 | 2 | 28870 |
| 20COX6231NPS | 11/19/2020 | 7155004 | 7155808 | 14301502 | 14174410 | 14174410 | 60057.94 | 1 | 176192 |
| 20COX6233NPS | 11/19/2020 | 7735199 | 7736690 | 15457302 | 14846201 | 14846201 | 64144.70 | 1 | 191124 |
| 20COX6234NPS | 11/19/2020 | 7735334 | 7736513 | 15460195 | 15246039 | 15246039 | 66027.42 | 1 | 197379 |
| 20COX6393NPS | 11/30/2020 | 1469350 | 1469398 | 2935504 | 2927318 | 2927318 | 11889.80 | 5 | 30979 |
| 20COX6394NPS | 11/30/2020 | 5183763 | 5190449 | 10361293 | 10294016 | 10294016 | 35284.31 | 1 | 136482 |
| 20COX6395NPS | 11/30/2020 | 9290238 | 9295928 | 18562521 | 17115205 | 17115205 | 57603.96 | 2 | 246857 |
| 20COX6396NPS | 11/30/2020 | 6914688 | 6924378 | 13821484 | 13604849 | 13604849 | 46301.23 | 1 | 178566 |
| 20COX6397NPS | 11/30/2020 | 1385863 | 1385986 | 2769078 | 2758035 | 2758035 | 12163.21 | 1 | 32606 |
| 20COX6399NPS | 11/30/2020 | 1271631 | 1271718 | 2540865 | 2518737 | 2518737 | 11507.61 | 1 | 28392 |
| 20COX6401NPS | 11/29/2020 | 2480492 | 2501424 | 4909505 | 4808384 | 4808384 | 22292.92 | 1 | 59397 |
| 20COX6402NPS | 11/30/2020 | 1413992 | 1414094 | 2824845 | 2784860 | 2784860 | 12804.48 | 1 | 35108 |
| 20COX6403NPS | 11/30/2020 | 1176337 | 1176399 | 2349568 | 2337284 | 2337284 | 10777.42 | 1 | 30436 |
| 20COX6406NPS | 11/30/2020 | 8957663 | 8972408 | 17903545 | 17419044 | 17419044 | 58300.11 | 1 | 201734 |
| 20COX6407NPS | 11/30/2020 | 631537 | 631549 | 1261927 | 1243471 | 1243471 | 5652.74 | 1 | 17473 |
| 20COX6409NPS | 11/30/2020 | 1044779 | 1044772 | 2087882 | 2046784 | 2046784 | 9371.14 | 4 | 25834 |
| 20COX6410NPS | 11/30/2020 | 8634258 | 8657503 | 17259339 | 16896779 | 16896779 | 56376.96 | 1 | 231484 |
| 20COX6414NPS | 11/30/2020 | 1040836 | 1040861 | 2079970 | 2075350 | 2075350 | 9497.19 | 2 | 25793 |
| 20COX6416NPS | 11/30/2020 | 1215949 | 1215990 | 2429583 | 2420876 | 2420876 | 11026.99 | 6 | 29344 |
| 20COX6417NPS | 11/30/2020 | 1475656 | 1475789 | 2948874 | 2903274 | 2903274 | 12961.53 | 1 | 35660 |
| 20COX6418NPS | 11/30/2020 | 578802 | 578828 | 1156463 | 1151642 | 1151642 | 5306.29 | 1 | 14045 |
| 20COX6419NPS | 11/30/2020 | 1472786 | 1472943 | 2940476 | 2924554 | 2924554 | 13217.30 | 1 | 39360 |
| 20COX6421NPS | 11/30/2020 | 1943744 | 1943842 | 3883762 | 3872903 | 3872903 | 18077.83 | 1 | 47867 |
| 20COX6422NPS | 11/30/2020 | 1573605 | 1573757 | 3143316 | 3132616 | 3132616 | 14047.79 | 1 | 34772 |
| 20COX6423NPS | 11/30/2020 | 1497170 | 1497311 | 2990850 | 2942416 | 2942416 | 13391.20 | 4 | 37389 |
| 20COX6424NPS | 11/30/2020 | 5517104 | 5526502 | 11026588 | 10440730 | 10440730 | 36430.93 | 1 | 137263 |
| 20COX6425NPS | 12/1/2020 | 563594 | 563629 | 1123820 | 1119397 | 1119397 | 5556.29 | 2 | 17359 |
| 20COX6426NPS | 11/30/2020 | 1194185 | 1194284 | 2385128 | 2376383 | 2376383 | 10905.00 | 1 | 30194 |
| 20COX6428NPS | 12/1/2020 | 573444 | 573477 | 1142281 | 1127092 | 1127092 | 5362.07 | 1 | 16521 |
| 20COX6429NPS | 12/1/2020 | 524696 | 524704 | 1045009 | 1043407 | 1043407 | 4929.17 | 1 | 15350 |
| 20COX6430NPS | 12/1/2020 | 11447223 | 11451422 | 22874810 | 22580736 | 22580736 | 74908.07 | 1 | 294817 |
| 20COX6433NPS | 12/1/2020 | 504648 | 504665 | 1005388 | 1000631 | 1000631 | 4763.15 | 1 | 15314 |
| 20COX6434NPS | 12/1/2020 | 580258 | 580273 | 1156007 | 1143146 | 1143146 | 5451.66 | 5 | 16829 |
| 20COX6436NPS | 12/1/2020 | 533817 | 533834 | 1063997 | 1058046 | 1058046 | 5009.92 | 1 | 15916 |
| 20COX6437NPS | 12/1/2020 | 1143992 | 1144003 | 2285525 | 1987863 | 1987863 | 8983.50 | 45 | 25525 |
| 20COX6438NPS | 12/1/2020 | 578431 | 578453 | 1153317 | 1143181 | 1143181 | 5364.71 | 2 | 16975 |
| 20COX6439NPS | 12/2/2020 | 468386 | 468409 | 934303 | 926343 | 926343 | 4322.37 | 1 | 13782 |
| 20COX6440NPS | 12/2/2020 | 575412 | 575441 | 1146285 | 1136608 | 1136608 | 5402.17 | 2 | 17089 |
| 20COX6441NPS | 12/2/2020 | 474203 | 474228 | 945157 | 930674 | 930674 | 4482.20 | 1 | 14433 |
| 20COX6442NPS | 12/2/2020 | 2668479 | 2668611 | 5330430 | 5029249 | 5029249 | 23279.27 | 1 | 65452 |
| 20COX6443NPS | 12/2/2020 | 1410088 | 1410531 | 2813839 | 2791100 | 2791100 | 12227.93 | 1 | 35566 |
| 20COX6444NPS | 12/2/2020 | 479238 | 479259 | 955530 | 954206 | 954206 | 4665.25 | 1 | 14656 |
| 20COX6446NPS | 12/2/2020 | 431307 | 431322 | 860030 | 854741 | 854741 | 3992.99 | 1 | 13176 |
| 20COX6447NPS | 12/2/2020 | 1360154 | 1360158 | 2717603 | 2670933 | 2670933 | 12453.69 | 1 | 32024 |
| 20COX6448NPS | 12/2/2020 | 1572071 | 1572215 | 3135847 | 3104940 | 3104940 | 14560.37 | 2 | 37636 |
| 20COX6450NPS | 12/2/2020 | 524427 | 524446 | 1045890 | 1031917 | 1031917 | 4900.18 | 2 | 16001 |
| 20COX6451NPS | 12/1/2020 | 473101 | 473117 | 942700 | 935443 | 935443 | 4445.57 | 3 | 13901 |
| 20COX6452NPS | 12/2/2020 | 589337 | 589347 | 1172834 | 1159376 | 1159376 | 5687.25 | 1 | 16091 |
| 20COX6454NPS | 12/2/2020 | 430283 | 430285 | 857220 | 853429 | 853429 | 4188.51 | 2 | 13111 |
| 20COX6455NPS | 12/2/2020 | 515833 | 515847 | 1028183 | 1023192 | 1023192 | 4974.13 | 2 | 15588 |
| 20COX6456NPS | 11/30/2020 | 1653654 | 1659001 | 3302351 | 8587 | 8587 | 23.89 | 1 | 80 |
| 20COX6457NPS | 12/2/2020 | 988381 | 988611 | 1973942 | 1923724 | 1923724 | 10868.63 | 1 | 29227 |
| 20COX6458NPS | 12/2/2020 | 473444 | 207019 | 679220 | 676151 | 676151 | 4178.87 | 1 | 13337 |
| 20COX6466NPS | 12/2/2020 | 447427 | 447440 | 891694 | 888945 | 888945 | 4186.78 | 1 | 12977 |
| 20COX6470NPS | 12/5/2020 | 1658416 | 1659418 | 3314363 | 2252454 | 2252454 | 66993.71 | 1 | 301606 |
| 20COX6474NPS | 12/6/2020 | 517281 | 517289 | 1030689 | 1028453 | 1028453 | 5036.19 | 1 | 15508 |
| 20COX6476NPS | 12/6/2020 | 758889 | 759056 | 1515980 | 1509040 | 1509040 | 8287.13 | 3 | 21187 |
| 20COX6477NPS | 12/6/2020 | 548896 | 548908 | 1093258 | 1017607 | 1017607 | 4956.03 | 1 | 15904 |
| 20COX6480NPS | 12/6/2020 | 428498 | 428502 | 853220 | 848849 | 848849 | 4171.97 | 1 | 13341 |
| 20COX6481NPS | 12/6/2020 | 1093745 | 1093900 | 2185542 | 2180385 | 2180385 | 12374.40 | 1 | 31971 |
| 20COX6482NPS | 12/6/2020 | 480467 | 480464 | 957349 | 956336 | 956336 | 4843.65 | 1 | 15453 |
| 20COX6483NPS | 12/7/2020 | 530002 | 530035 | 1055902 | 1047143 | 1047143 | 5105.37 | 1 | 16118 |
| 20COX6487NPS | 12/7/2020 | 1108533 | 1108636 | 2213831 | 2120018 | 2120018 | 9778.37 | 1 | 23869 |
| 20COX6488NPS | 12/6/2020 | 1204125 | 1204201 | 2405858 | 2330718 | 2330718 | 10531.67 | 1 | 29297 |
| 20COX6489NPS | 12/7/2020 | 1364939 | 1365308 | 2727113 | 2708849 | 2708849 | 14581.10 | 3 | 39227 |
| 20COX6490NPS | 12/7/2020 | 1029135 | 1029266 | 2056077 | 2015956 | 2015956 | 11312.93 | 35 | 30927 |
| 20COX6492NPS | 12/7/2020 | 1303056 | 1302961 | 2603356 | 2592194 | 2592194 | 14688.25 | 1 | 38394 |
| 20COX6493NPS | 12/7/2020 | 476479 | 476494 | 948863 | 917504 | 917504 | 4480.26 | 1 | 13938 |
| 20COX6494NPS | 12/7/2020 | 460264 | 460264 | 918067 | 915852 | 915852 | 4428.77 | 1 | 13987 |
| 20COX6497NPS | 12/7/2020 | 523348 | 523370 | 1041686 | 1035735 | 1035735 | 4844.47 | 4 | 16070 |
| 20COX6499NPS | 12/7/2020 | 1945857 | 1946011 | 3887612 | 3797309 | 3797309 | 20378.70 | 2 | 53717 |
| 20COX6500NPS | 12/7/2020 | 501683 | 501722 | 998698 | 990250 | 990250 | 4693.03 | 1 | 14760 |
| 20COX6501NPS | 12/7/2020 | 1125266 | 1125498 | 2247484 | 2075335 | 2075335 | 11110.23 | 3 | 31465 |
| 20COX6502NPS | 12/7/2020 | 1934798 | 1935072 | 3865719 | 3684906 | 3684906 | 19888.73 | 3 | 56125 |
| 20COX6503NPS | 12/7/2020 | 473104 | 473095 | 941881 | 926273 | 926273 | 4408.55 | 1 | 14729 |
| 20COX6504NPS | 12/7/2020 | 426286 | 426277 | 849304 | 848622 | 848622 | 4174.37 | 1 | 13258 |
| 20COX6505NPS | 12/7/2020 | 992971 | 993120 | 1984372 | 1978992 | 1978992 | 9791.62 | 1 | 26609 |
| 20COX6506NPS | 12/7/2020 | 552215 | 552236 | 1099036 | 1088078 | 1088078 | 5265.75 | 2 | 17121 |
| 20COX6507NPS | 12/6/2020 | 460406 | 460408 | 917685 | 911953 | 911953 | 4427.32 | 2 | 12961 |
| 20COX6508NPS | 12/6/2020 | 1035134 | 1035241 | 2068022 | 2048102 | 2048102 | 9451.06 | 2 | 26728 |
| 20COX6509NPS | 12/7/2020 | 1072103 | 1072119 | 2142688 | 2114300 | 2114300 | 9547.04 | 1 | 26421 |
| 20COX6510NPS | 12/7/2020 | 1226458 | 1226623 | 2449956 | 2359143 | 2359143 | 12767.70 | 32 | 35493 |
| 20COX6511NPS | 12/7/2020 | 1247842 | 1248378 | 2493032 | 2439196 | 2439196 | 12883.22 | 1 | 40115 |
| 20COX6512NPS | 12/7/2020 | 893746 | 894040 | 1785936 | 1731746 | 1731746 | 9548.99 | 1 | 25535 |
| 20COX6513NPS | 12/7/2020 | 1461822 | 1462153 | 2919392 | 2821995 | 2821995 | 16002.23 | 4 | 43047 |
| 20COX6514NPS | 12/8/2020 | 1045875 | 1046255 | 2088593 | 2024777 | 2024777 | 11409.77 | 1 | 29669 |
| 20COX6516NPS | 12/8/2020 | 1555440 | 1555721 | 3107741 | 2977735 | 2977735 | 17053.10 | 1 | 45506 |
| 20COX6517NPS | 12/8/2020 | 1082512 | 1082602 | 2161880 | 2071870 | 2071870 | 11389.39 | 1 | 37556 |
| 20COX6518NPS | 12/8/2020 | 1057505 | 1057754 | 2109276 | 1910635 | 1910635 | 8672.22 | 2 | 26547 |
| 20COX6519NPS | 12/8/2020 | 1483450 | 1483564 | 2963502 | 2906513 | 2906513 | 13162.83 | 5 | 38689 |
| 20COX6520NPS | 12/8/2020 | 1546625 | 1547122 | 3089003 | 2998627 | 2998627 | 17061.52 | 1 | 46599 |
| 20COX6521NPS | 12/8/2020 | 1495295 | 1495437 | 2988045 | 2981704 | 2981704 | 15989.24 | 1 | 41906 |
| 20COX6522NPS | 12/8/2020 | 406652 | 406755 | 812396 | 809945 | 809945 | 4562.52 | 1 | 11685 |
| 20COX6524NPS | 12/8/2020 | 446329 | 446349 | 889934 | 885044 | 885044 | 4213.87 | 1 | 13258 |
| 20COX6525NPS | 12/8/2020 | 1801034 | 1801430 | 3597835 | 3478130 | 3478130 | 19189.26 | 8 | 55779 |
| 20COX6526NPS | 12/8/2020 | 455726 | 455729 | 908569 | 903052 | 903052 | 4285.78 | 2 | 13546 |
| 20COX6527NPS | 12/8/2020 | 1322302 | 1322514 | 2641431 | 2504594 | 2504594 | 14469.03 | 1 | 39157 |
| 20COX6528NPS | 12/8/2020 | 436031 | 436054 | 869175 | 866095 | 866095 | 4087.33 | 1 | 13226 |
| 20COX6529NPS | 12/8/2020 | 500445 | 500474 | 997233 | 990181 | 990181 | 4598.25 | 1 | 14585 |
| 20COX6532NPS | 12/7/2020 | 3032223 | 3032184 | 6048798 | 624416 | 624416 | 9861.59 | 1 | 82333 |
| 20COX6534NPS | 12/8/2020 | 1178725 | 1178960 | 2355467 | 2345966 | 2345966 | 10400.16 | 55 | 28746 |
| 20COX6536NPS | 12/8/2020 | 529877 | 529904 | 1055066 | 1040243 | 1040243 | 4876.50 | 1 | 15172 |
| 20COX6540NPS | 12/9/2020 | 480014 | 480239 | 958425 | 934655 | 934655 | 5412.98 | 1 | 14330 |
| 20COX6541NPS | 12/9/2020 | 1499295 | 1499562 | 2996454 | 2972204 | 2972204 | 15559.07 | 3 | 36145 |
| 20COX6542NPS | 12/9/2020 | 1074538 | 1074906 | 2144859 | 2055984 | 2055984 | 12099.32 | 2 | 31213 |
| 20COX6544NPS | 12/9/2020 | 550882 | 550796 | 1100674 | 1062183 | 1062183 | 6102.97 | 1 | 17161 |
| 20COX6545NPS | 12/9/2020 | 1051361 | 1051590 | 2099123 | 2031254 | 2031254 | 11567.54 | 1 | 31196 |
| 20COX6548NPS | 12/9/2020 | 1605473 | 1606167 | 3208531 | 3157904 | 3157904 | 11117.74 | 1 | 35548 |
| 20COX6549NPS | 12/9/2020 | 1017711 | 1017891 | 2033478 | 1993920 | 1993920 | 10685.70 | 1 | 29658 |
| 20COX6551NPS | 12/9/2020 | 1432507 | 1432822 | 2861616 | 2820185 | 2820185 | 15597.31 | 1 | 38320 |
| 20COX6552NPS | 12/9/2020 | 947252 | 947595 | 1892655 | 1880508 | 1880508 | 10362.64 | 1 | 27882 |
| 20COX6557NPS | 12/9/2020 | 470405 | 470554 | 939348 | 902796 | 902796 | 5110.81 | 2 | 13432 |
| 20COX6560NPS | 12/9/2020 | 532389 | 534295 | 1063973 | 176765 | 176765 | 2829.88 | 1 | 15815 |
| 20COX6565NPS | 12/10/2020 | 1882663 | 1883002 | 3761999 | 3742553 | 3742553 | 19348.15 | 2 | 52698 |
| 20COX6566NPS | 12/10/2020 | 913349 | 913485 | 1825051 | 1769565 | 1769565 | 9341.51 | 1 | 27095 |
| 20COX6569NPS | 12/10/2020 | 1833797 | 1840689 | 3663111 | 1843184 | 1843184 | 30627.93 | 1 | 190856 |
| 20COX6571NPS | 12/10/2020 | 580096 | 580314 | 1158213 | 1116744 | 1116744 | 6310.62 | 1 | 18742 |
| 20COX6572NPS | 12/10/2020 | 499700 | 499708 | 995414 | 976633 | 976633 | 4724.42 | 1 | 14846 |
| 20COX6573NPS | 12/10/2020 | 1073763 | 1073972 | 2145217 | 2138474 | 2138474 | 10864.05 | 40 | 27809 |
| 20COX6575NPS | 12/10/2020 | 1191133 | 1191508 | 2380130 | 2374191 | 2374191 | 13194.36 | 5 | 34385 |
| 20COX6576NPS | 12/10/2020 | 730367 | 730381 | 1457474 | 1456302 | 1456302 | 7181.02 | 1 | 22267 |
| 20COX6579NPS | 12/10/2020 | 477519 | 477516 | 952813 | 949017 | 949017 | 4647.11 | 1 | 14407 |
| 20COX6581NPS | 12/10/2020 | 467088 | 467097 | 932004 | 930996 | 930996 | 4577.27 | 1 | 13032 |
| 20COX6584NPS | 12/10/2020 | 1138935 | 1139127 | 2274849 | 1795377 | 1795377 | 8806.12 | 1 | 27156 |
| 20COX6587NPS | 12/11/2020 | 377904 | 377881 | 754360 | 753628 | 753628 | 3624.59 | 3 | 11181 |
| 20COX6588NPS | 12/11/2020 | 390609 | 390594 | 779831 | 778950 | 778950 | 3736.25 | 1 | 12074 |
| 20COX6589NPS | 12/11/2020 | 501571 | 501604 | 999588 | 971598 | 971598 | 4648.12 | 1 | 14558 |
| 20COX6591NPS | 12/11/2020 | 638460 | 638482 | 1273523 | 1272543 | 1272543 | 6137.56 | 1 | 17335 |
| 20COX6592NPS | 12/11/2020 | 691040 | 691265 | 1380304 | 1371802 | 1371802 | 7719.49 | 1 | 20151 |
| 20COX6594NPS | 12/11/2020 | 544473 | 544747 | 1087571 | 1079062 | 1079062 | 5985.01 | 1 | 18448 |
| 20COX6595NPS | 12/11/2020 | 614575 | 614749 | 1226846 | 1190714 | 1190714 | 6867.90 | 1 | 18222 |
| 20COX6598NPS | 12/11/2020 | 3829774 | 3847566 | 7663826 | 7291329 | 7291329 | 19020.18 | 1 | 51119 |
| 20COX6599NPS | 12/11/2020 | 584318 | 584474 | 1167241 | 1156889 | 1156889 | 6280.68 | 1 | 17503 |
| 20COX6600NPS | 12/11/2020 | 707245 | 707411 | 1412795 | 1401800 | 1401800 | 7742.62 | 34 | 20920 |
| 20COX6601NPS | 12/11/2020 | 622736 | 622687 | 1244346 | 1231146 | 1231146 | 6345.97 | 1 | 18070 |
| 20COX6602NPS | 12/11/2020 | 710931 | 711164 | 1420276 | 1415608 | 1415608 | 6911.90 | 1 | 17080 |
| 20COX6603NPS | 12/11/2020 | 782440 | 782627 | 1563677 | 1548755 | 1548755 | 7346.98 | 1 | 21307 |
| 20COX6607NPS | 12/10/2020 | 438566 | 438798 | 876398 | 872917 | 872917 | 4660.14 | 20 | 11749 |
| 20COX6615NPS | 12/11/2020 | 1079531 | 1079877 | 2156477 | 2077120 | 2077120 | 10134.34 | 1 | 27862 |
| 20COX6616NPS | 12/12/2020 | 792936 | 792927 | 1584459 | 1540296 | 1540296 | 8103.13 | 2 | 23050 |
| 20COX6618NPS | 12/12/2020 | 313824 | 313807 | 626376 | 624855 | 624855 | 3040.11 | 2 | 9485 |
| 20COX6619NPS | 12/12/2020 | 850181 | 850433 | 1699122 | 1694491 | 1694491 | 9201.74 | 5 | 24830 |
| 20COX6620NPS | 12/12/2020 | 999506 | 999718 | 1996489 | 1989842 | 1989842 | 10905.48 | 4 | 28327 |
| 20COX6621NPS | 12/12/2020 | 644688 | 645122 | 1286543 | 1278590 | 1278590 | 6937.87 | 1 | 18446 |
| 20COX6623NPS | 12/12/2020 | 1215333 | 1215658 | 2428409 | 2421004 | 2421004 | 11861.95 | 1 | 30492 |
| 20COX6624NPS | 12/12/2020 | 1131449 | 1131731 | 2260839 | 2253965 | 2253965 | 12326.99 | 1 | 32435 |
| 20COX6625NPS | 12/12/2020 | 803016 | 803317 | 1604608 | 1598819 | 1598819 | 8692.07 | 1 | 22914 |
| 20COX6626NPS | 12/12/2020 | 979885 | 980079 | 1957782 | 1952879 | 1952879 | 10821.10 | 1 | 28877 |
| 20COX6629NPS | 12/12/2020 | 937590 | 937808 | 1873300 | 1867609 | 1867609 | 10398.12 | 1 | 28092 |
| 20COX6630NPS | 12/12/2020 | 776167 | 776215 | 1551017 | 1546841 | 1546841 | 7948.47 | 3 | 19927 |
| 20COX6631NPS | 12/12/2020 | 1557081 | 1557419 | 3111284 | 3101114 | 3101114 | 15893.23 | 1 | 44528 |
| 20COX6632NPS | 12/12/2020 | 1026408 | 1026623 | 2050132 | 2044440 | 2044440 | 10994.59 | 1 | 28567 |
| 20COX6633NPS | 12/12/2020 | 1279837 | 1280158 | 2556819 | 2547670 | 2547670 | 13478.77 | 1 | 34600 |
| 20COX6634NPS | 12/12/2020 | 788347 | 788481 | 1575517 | 1572429 | 1572429 | 8309.68 | 3 | 22844 |
| 20COX6635NPS | 12/12/2020 | 421739 | 421708 | 841954 | 840464 | 840464 | 4053.88 | 1 | 12724 |
| 20COX6636NPS | 12/12/2020 | 966172 | 966338 | 1930563 | 1888817 | 1888817 | 8753.30 | 1 | 25643 |
| 20COX6647NPS | 12/14/2020 | 668408 | 668392 | 1334543 | 1332901 | 1332901 | 6399.89 | 1 | 19415 |
| 20COX6653NPS | 12/14/2020 | 518425 | 518425 | 1034480 | 1031200 | 1031200 | 4946.59 | 1 | 15818 |
| 20COX6655NPS | 12/14/2020 | 869178 | 869366 | 1736757 | 1692706 | 1692706 | 8920.85 | 1 | 23961 |
| 20COX6656NPS | 12/14/2020 | 609772 | 610027 | 1218101 | 1198095 | 1198095 | 6550.28 | 2 | 17717 |
| 20COX6659NPS | 12/14/2020 | 485013 | 485026 | 968254 | 965034 | 965034 | 4572.35 | 2 | 14667 |
| 20COX6660NPS | 12/14/2020 | 445673 | 445697 | 888746 | 869233 | 869233 | 4103.62 | 1 | 13328 |
| 20COX6661NPS | 12/14/2020 | 487869 | 487881 | 973565 | 969735 | 969735 | 4563.94 | 1 | 13774 |
| 20COX6663NPS | 12/14/2020 | 466584 | 466883 | 931839 | 915411 | 915411 | 5077.22 | 3 | 14966 |
| 20COX6664NPS | 12/14/2020 | 63366 | 63368 | 126542 | 126065 | 126065 | 708.80 | 2 | 1771 |
| 20COX6671NPS | 12/15/2020 | 356611 | 356598 | 711673 | 682075 | 682075 | 3264.57 | 1 | 10799 |
| 20COX6672NPS | 12/15/2020 | 415107 | 415127 | 827398 | 767796 | 767796 | 3713.58 | 2 | 11506 |
| 20MU10001NPS | 11/18/2021 | 651377 | 651377 | 1301121 | 1271188 | 1271188 | 5205.67 | 1 | 34810 |
| 20MU10007NPS | 11/19/2021 | 407835 | 407835 | 815013 | 641123 | 641123 | 2531.97 | 34 | 8402 |
| 20MU10008NPS | 11/19/2021 | 561764 | 561764 | 1122575 | 993694 | 993694 | 3910.97 | 1 | 22907 |
| 20MU10011NPS | 11/20/2021 | 870337 | 870337 | 1737663 | 1732739 | 1732739 | 7016.84 | 1 | 49025 |
| 20MU10013NPS | 11/21/2021 | 1025379 | 1025379 | 2046366 | 2020362 | 2020362 | 8209.99 | 1 | 61948 |
| 20MU10014NPS | 11/21/2021 | 126187 | 126187 | 247514 | 240372 | 240372 | 1026.14 | 1 | 5205 |
| 20MU10016NPS | 11/21/2021 | 845971 | 845971 | 1688943 | 1685698 | 1685698 | 6818.35 | 1 | 36987 |
| 20MU10017NPS | 11/21/2021 | 399073 | 399073 | 797590 | 796281 | 796281 | 3204.57 | 1 | 9253 |
| 20MU10020NPS | 11/23/2021 | 440748 | 440748 | 880848 | 878879 | 878879 | 3489.13 | 1 | 10430 |
| 20MU10021NPS | 11/23/2021 | 1089229 | 1089229 | 2174953 | 2162187 | 2162187 | 8783.44 | 1 | 59175 |
| 20MU10024NPS | 11/24/2021 | 595693 | 595693 | 1190514 | 1154537 | 1154537 | 4563.05 | 1 | 22144 |
| 20MU10025NPS | 11/23/2021 | 1182299 | 1182299 | 2360738 | 2329780 | 2329780 | 9390.11 | 1 | 55456 |
| 20MU10027NPS | 11/24/2021 | 841733 | 841733 | 1681060 | 1666407 | 1666407 | 6763.79 | 1 | 48887 |
| 20MU10028NPS | 11/24/2021 | 961336 | 961336 | 1918137 | 1914110 | 1914110 | 7737.71 | 1 | 34612 |
| 20MU10029NPS | 11/24/2021 | 850466 | 850466 | 1697951 | 1691844 | 1691844 | 6922.14 | 1 | 30271 |
| 20MU10030NPS | 11/24/2021 | 1215101 | 1215101 | 2425352 | 2419124 | 2419124 | 9792.85 | 1 | 67547 |
| 20MU10031NPS | 11/24/2021 | 1094832 | 1094832 | 2185273 | 2178033 | 2178033 | 8929.33 | 1 | 58251 |
| 20MU10032NPS | 11/24/2021 | 81928 | 81928 | 163523 | 149452 | 149452 | 639.75 | 1 | 4099 |
| 20MU10033NPS | 11/24/2021 | 867321 | 867321 | 1731334 | 1705184 | 1705184 | 7025.79 | 1 | 48469 |
| 20MU10037NPS | 11/26/2021 | 1200820 | 1200820 | 2397253 | 2392830 | 2392830 | 9646.05 | 1 | 54562 |
| 20MU10038NPS | 11/27/2021 | 501355 | 501355 | 1002070 | 889352 | 889352 | 3509.94 | 1 | 11778 |
| 20MU10040NPS | 11/27/2021 | 484301 | 484301 | 967893 | 942902 | 942902 | 3729.42 | 22 | 13451 |
| 20MU10041NPS | 11/27/2021 | 538268 | 538268 | 1075853 | 1071164 | 1071164 | 4250.19 | 1 | 13575 |
| 20MU10042NPS | 11/27/2021 | 248230 | 248230 | 496084 | 495215 | 495215 | 1984.79 | 8 | 6102 |
| 20MU10044NPS | 11/28/2021 | 965613 | 965613 | 1927745 | 1924222 | 1924222 | 7800.85 | 1 | 26777 |
| 20MU10045NPS | 11/28/2021 | 801534 | 801534 | 1600116 | 1596622 | 1596622 | 6499.56 | 1 | 42631 |
| 20MU10046NPS | 11/28/2021 | 760754 | 760754 | 1518466 | 1515675 | 1515675 | 6133.30 | 1 | 23928 |
| 20MU10047NPS | 11/28/2021 | 630617 | 630617 | 1258824 | 1242841 | 1242841 | 5083.81 | 1 | 30330 |
| 20MU10048NPS | 11/28/2021 | 622812 | 622812 | 1243299 | 1207007 | 1207007 | 4928.03 | 1 | 24125 |
| 20MU10049NPS | 11/28/2021 | 1024770 | 1024770 | 2045672 | 2042018 | 2042018 | 8254.76 | 1 | 39504 |
| 20MU10050NPS | 11/28/2021 | 897384 | 897384 | 1792077 | 1789183 | 1789183 | 7260.60 | 1 | 37567 |
| 20MU10051NPS | 11/28/2021 | 506631 | 506631 | 1012435 | 929648 | 929648 | 3671.86 | 28 | 31425 |
| 20MU10052NPS | 11/28/2021 | 251168 | 251168 | 501862 | 501014 | 501014 | 2009.26 | 1 | 5503 |
| 20MU10053NPS | 11/29/2021 | 290880 | 290880 | 581107 | 579853 | 579853 | 2267.23 | 1 | 7940 |
| 20MU10054NPS | 11/29/2021 | 835926 | 835926 | 1668878 | 1665635 | 1665635 | 6776.19 | 1 | 38793 |
| 20MU10057NPS | 11/29/2021 | 942443 | 942443 | 1881213 | 1877622 | 1877622 | 7605.16 | 1 | 34727 |
| 20MU10059NPS | 11/29/2021 | 886212 | 886212 | 1768317 | 1764666 | 1764666 | 7117.64 | 1 | 24431 |
| 20MU10061NPS | 11/29/2021 | 153435 | 153435 | 306653 | 306103 | 306103 | 1249.90 | 1 | 3374 |
| 20MU10062NPS | 11/29/2021 | 503043 | 503043 | 1005416 | 999696 | 999696 | 3969.45 | 1 | 13425 |
| 20MU10063NPS | 11/29/2021 | 221547 | 221547 | 442686 | 442037 | 442037 | 1792.65 | 20 | 5039 |
| 20MU10065NPS | 11/29/2021 | 175629 | 175629 | 350894 | 350429 | 350429 | 1422.49 | 2 | 4089 |
| 20MU10066NPS | 11/29/2021 | 986155 | 986155 | 1969043 | 1965586 | 1965586 | 8010.38 | 1 | 34606 |
| 20MU10068NPS | 11/29/2021 | 970103 | 970103 | 1932677 | 1927477 | 1927477 | 7823.60 | 1 | 47951 |
| 20MU10071NPS | 11/29/2021 | 370453 | 370453 | 740262 | 736812 | 736812 | 2922.84 | 34 | 13132 |
| 20MU10072NPS | 11/29/2021 | 873979 | 873979 | 1743655 | 1739915 | 1739915 | 7044.49 | 1 | 24823 |
| 20MU10081NPS | 12/2/2021 | 634967 | 634967 | 1269268 | 1268136 | 1268136 | 5028.98 | 1 | 22019 |
| 20MU10082NPS | 12/3/2021 | 774376 | 774376 | 1545933 | 1543034 | 1543034 | 6259.28 | 1 | 23462 |
| 20MU10083NPS | 12/3/2021 | 354122 | 354122 | 706528 | 704818 | 704818 | 2849.22 | 1 | 10027 |
| 20MU10084NPS | 12/5/2021 | 475671 | 475671 | 949368 | 900907 | 900907 | 3645.07 | 4 | 18045 |
| 20MU10085NPS | 12/6/2021 | 621978 | 621978 | 1242312 | 1226613 | 1226613 | 4918.66 | 2 | 26344 |
| 20MU10086NPS | 12/6/2021 | 450814 | 450814 | 900116 | 841737 | 841737 | 3403.07 | 1 | 24101 |
| 20MU10087NPS | 12/7/2021 | 383614 | 383614 | 766941 | 758334 | 758334 | 2994.82 | 8 | 20335 |
| 20MU10088NPS | 12/7/2021 | 1059454 | 1059454 | 2115258 | 2110264 | 2110264 | 8454.56 | 1 | 49025 |
| 20MU10089NPS | 12/8/2021 | 593191 | 593191 | 1184802 | 1082009 | 1082009 | 4359.49 | 16 | 21165 |
| 20MU10090NPS | 12/9/2021 | 649833 | 649833 | 1299065 | 1293049 | 1293049 | 5102.32 | 1 | 26771 |
| 20MU10091NPS | 12/9/2021 | 528509 | 528509 | 1054792 | 999449 | 999449 | 3989.97 | 2 | 26774 |
| 20MU10092NPS | 12/9/2021 | 1194499 | 1194499 | 2385303 | 2287823 | 2287823 | 9222.20 | 18 | 58434 |
| 20MU10094NPS | 12/10/2021 | 760288 | 760288 | 1519807 | 1518029 | 1518029 | 6011.46 | 25 | 33770 |
| 20MU10097NPS | 12/12/2021 | 791522 | 791522 | 1579981 | 1576749 | 1576749 | 6346.28 | 1 | 35632 |
| 20MU10098NPS | 12/12/2021 | 708952 | 708952 | 1415453 | 1393565 | 1393565 | 5589.73 | 1 | 35584 |
| 20MU10099NPS | 12/12/2021 | 430653 | 430653 | 859113 | 719018 | 719018 | 2893.84 | 9 | 19713 |
| 20MU10101NPS | 12/13/2021 | 262781 | 262781 | 525296 | 523588 | 523588 | 2067.96 | 1 | 11096 |
| 20MU10102NPS | 12/13/2021 | 1052525 | 1052525 | 2102356 | 2086418 | 2086418 | 8384.42 | 1 | 55571 |
| 20MU10103NPS | 12/14/2021 | 1009958 | 1009958 | 2016199 | 2010762 | 2010762 | 8109.87 | 3 | 41919 |
| 20MU10104NPS | 12/14/2021 | 375535 | 375535 | 749802 | 679598 | 679598 | 2756.56 | 5 | 18143 |
| 20MU10106NPS | 12/14/2021 | 901100 | 901100 | 1799102 | 1794986 | 1794986 | 7261.03 | 1 | 39060 |
| 20MU10108NPS | 12/14/2021 | 322512 | 322512 | 644792 | 641643 | 641643 | 2542.92 | 8 | 15606 |
| 20MU10110NPS | 12/17/2021 | 1289357 | 1289357 | 2573684 | 2567952 | 2567952 | 10340.48 | 1 | 40933 |
| 20MU10112NPS | 12/17/2021 | 917621 | 917621 | 1832920 | 1823656 | 1823656 | 7241.34 | 1 | 45955 |
| 20MU10119NPS | 12/19/2021 | 502349 | 502349 | 1004211 | 1003303 | 1003303 | 3956.19 | 1 | 37643 |
| 20MU10120NPS | 12/20/2021 | 785913 | 785913 | 1568438 | 1486235 | 1486235 | 5888.24 | 1 | 36418 |
| 20MU10122NPS | 12/20/2021 | 661130 | 661130 | 1320664 | 1311064 | 1311064 | 5215.80 | 1 | 32034 |
| 20MU10125NPS | 12/21/2021 | 1093533 | 1093533 | 2183887 | 2164818 | 2164818 | 8619.80 | 1 | 51608 |
| 20MU10126NPS | 12/21/2021 | 992216 | 992216 | 1980742 | 1974450 | 1974450 | 7936.26 | 1 | 41048 |
| 20MU10127NPS | 12/21/2021 | 643261 | 643261 | 1283694 | 1100948 | 1100948 | 4360.52 | 1 | 26706 |
| 20MU10128NPS | 12/21/2021 | 490569 | 490569 | 978856 | 641719 | 641719 | 2561.50 | 1 | 12157 |
| 20MU10129NPS | 12/21/2021 | 894578 | 894578 | 1785957 | 1605144 | 1605144 | 6327.33 | 10 | 44653 |
| 20MU10130NPS | 12/21/2021 | 692780 | 692780 | 1383406 | 1362808 | 1362808 | 5462.25 | 1 | 33423 |
| 20MU10132NPS | 12/22/2021 | 855126 | 855126 | 1707781 | 1704698 | 1704698 | 6831.33 | 1 | 51775 |
| 20MU10133NPS | 12/22/2021 | 900403 | 900403 | 1797419 | 1659269 | 1659269 | 6641.58 | 1 | 48292 |
| 20MU10134NPS | 12/22/2021 | 3807240 | 3807240 | 7605023 | 7386904 | 7386904 | 29301.26 | 1 | 201838 |
| 20MU10135NPS | 12/22/2021 | 2524650 | 2524743 | 5043625 | 4339890 | 4339890 | 16355.38 | 1 | 45699 |
| 20MU10137NPS | 12/22/2021 | 743393 | 743393 | 1474930 | 21044 | 21044 | 39.66 | 1 | 1490 |
| 20MU10138NPS | 12/23/2021 | 969427 | 969427 | 1935677 | 1929657 | 1929657 | 7760.01 | 1 | 41104 |
| 20MU10140NPS | 12/23/2021 | 424801 | 424801 | 847627 | 684726 | 684726 | 2746.96 | 1 | 16153 |
| 20MU10141NPS | 12/23/2021 | 1043009 | 1043009 | 2082574 | 2046078 | 2046078 | 8150.98 | 1 | 51527 |
| 20MU10142NPS | 12/23/2021 | 664099 | 664099 | 1326228 | 1296335 | 1296335 | 5149.33 | 2 | 40966 |
| 20MU10143NPS | 12/23/2021 | 617799 | 617799 | 1233439 | 1160202 | 1160202 | 4635.06 | 2 | 34743 |
| 20MU10144NPS | 12/23/2021 | 1051768 | 1051768 | 2101073 | 2095425 | 2095425 | 8361.38 | 2 | 63197 |
| 20MU10145NPS | 12/24/2021 | 360203 | 360203 | 720099 | 717281 | 717281 | 2839.45 | 8 | 16432 |
| 20MU10149NPS | 12/26/2021 | 1985286 | 1985286 | 3962761 | 3952471 | 3952471 | 15948.78 | 1 | 114328 |
| 20MU10150NPS | 12/26/2021 | 880360 | 880360 | 1758559 | 1753896 | 1753896 | 7045.73 | 1 | 48710 |
| 20MU10153NPS | 12/27/2021 | 1145824 | 1145824 | 2287537 | 2278105 | 2278105 | 9187.70 | 1 | 45243 |
| 20MU10154NPS | 12/17/2021 | 2017734 | 2017734 | 4030313 | 4020500 | 4020500 | 16075.15 | 1 | 119781 |
| 20MU10155NPS | 12/27/2021 | 1219549 | 1219549 | 2434176 | 2429284 | 2429284 | 9675.80 | 1 | 36150 |
| 20MU10160NPS | 12/27/2021 | 391208 | 391208 | 780556 | 605937 | 605937 | 2445.54 | 2 | 21107 |
| 20MU10162NPS | 12/27/2021 | 794601 | 794601 | 1586481 | 1549472 | 1549472 | 6206.09 | 1 | 40632 |
| 20MU10163NPS | 12/27/2021 | 712376 | 712376 | 1422564 | 1419973 | 1419973 | 5789.19 | 1 | 20133 |
| 20MU10164NPS | 12/27/2021 | 947601 | 947601 | 1892159 | 1888046 | 1888046 | 7492.81 | 1 | 26458 |
| 20MU10167NPS | 12/28/2021 | 748828 | 748828 | 1495298 | 1492224 | 1492224 | 5971.64 | 1 | 18362 |
| 20MU10172NPS | 12/29/2021 | 681671 | 681671 | 1361725 | 1357542 | 1357542 | 5319.08 | 1 | 74298 |
| 20MU10175NPS | 12/29/2021 | 931914 | 931914 | 1861361 | 1851389 | 1851389 | 7358.61 | 1 | 49548 |
| 20MU10176NPS | 12/29/2021 | 808662 | 808662 | 1614250 | 1610988 | 1610988 | 6372.12 | 1 | 23770 |
| 20MU10177NPS | 12/29/2021 | 1106473 | 1106473 | 2209267 | 2168391 | 2168391 | 6772.35 | 1 | 33152 |
| 20MU10178NPS | 12/29/2021 | 1209377 | 1209377 | 2415189 | 2411214 | 2411214 | 7941.40 | 1 | 55198 |
| 20MU10179NPS | 12/29/2021 | 810196 | 810196 | 1618786 | 1612612 | 1612612 | 5580.92 | 1 | 57458 |
| 20MU10180NPS | 12/29/2021 | 491732 | 491732 | 981672 | 954327 | 954327 | 3730.63 | 1 | 38367 |
| 20MU10182NPS | 12/29/2021 | 641473 | 641473 | 1280565 | 1277977 | 1277977 | 4364.85 | 1 | 23128 |
| 20MU10186NPS | 12/29/2021 | 279103 | 279103 | 557863 | 487059 | 487059 | 1951.57 | 1 | 22090 |
| 20MU10189NPS | 12/29/2021 | 1479019 | 1479019 | 2952702 | 2947435 | 2947435 | 9117.82 | 1 | 59965 |
| 20MU10190NPS | 12/29/2021 | 652992 | 652992 | 1304055 | 1301787 | 1301787 | 4740.04 | 1 | 27633 |
| 20MU10191NPS | 12/30/2021 | 629317 | 629317 | 1256758 | 1218404 | 1218404 | 4894.17 | 3 | 27351 |
| 20MU10192NPS | 12/30/2021 | 900582 | 900582 | 1797615 | 1792780 | 1792780 | 7237.29 | 1 | 36697 |
| 20MU10194NPS | 12/30/2021 | 973859 | 973859 | 1944364 | 1940261 | 1940261 | 7732.36 | 1 | 24124 |
| 20MU10195NPS | 12/30/2021 | 1058062 | 1058062 | 2112577 | 2108966 | 2108966 | 8375.61 | 1 | 29784 |
| 20MU10196NPS | 12/30/2021 | 551012 | 551012 | 1100460 | 1074777 | 1074777 | 4299.86 | 1 | 16195 |
| 20MU10198NPS | 12/30/2021 | 750094 | 750094 | 1497274 | 1380622 | 1380622 | 5549.77 | 1 | 35553 |
| 20MU10200NPS | 12/30/2021 | 1068230 | 1068230 | 2133035 | 2128161 | 2128161 | 8467.74 | 1 | 63923 |
| 20MU10201NPS | 12/30/2021 | 413149 | 413149 | 825884 | 825037 | 825037 | 3280.84 | 1 | 10686 |
| 20MU10202NPS | 12/30/2021 | 1005599 | 1005599 | 2006674 | 2002007 | 2002007 | 8120.15 | 1 | 33637 |
| 20MU10203NPS | 12/30/2021 | 1063780 | 1063780 | 2121304 | 1766153 | 1766153 | 6961.26 | 1 | 37102 |
| 20MU10205NPS | 12/30/2021 | 1084355 | 1084355 | 2165058 | 2160659 | 2160659 | 8802.48 | 1 | 35712 |
| 20MU10206NPS | 12/30/2021 | 1086332 | 1086332 | 2168725 | 2164726 | 2164726 | 8653.46 | 1 | 31248 |
| 20MU10207NPS | 11/23/2021 | 1206195 | 1206195 | 2408940 | 2403582 | 2403582 | 9705.12 | 1 | 27674 |
| 20MU10209NPS | 12/30/2021 | 1384415 | 1384415 | 2763221 | 2751005 | 2751005 | 6924.27 | 1 | 60431 |
| 20MU10214NPS | 12/30/2021 | 263575 | 263575 | 526948 | 516173 | 516173 | 2040.12 | 18 | 13013 |
| 20MU10215NPS | 12/30/2021 | 735316 | 735316 | 1467118 | 1463749 | 1463749 | 5949.00 | 1 | 27337 |
| 20MU10216NPS | 12/30/2021 | 421171 | 421171 | 841858 | 840866 | 840866 | 3263.16 | 1 | 12230 |
| 20MU10217NPS | 12/30/2021 | 995980 | 995980 | 1988815 | 1983172 | 1983172 | 7855.00 | 1 | 44888 |
| 20MU10219NPS | 12/30/2021 | 790761 | 790761 | 1579018 | 1568399 | 1568399 | 6184.94 | 4 | 29361 |
| 20MU10220NPS | 12/30/2021 | 1137839 | 1137872 | 2273307 | 1942887 | 1942887 | 7263.28 | 1 | 17832 |
| 20MU10222NPS | 12/30/2021 | 957724 | 957724 | 1912004 | 1895248 | 1895248 | 7590.00 | 1 | 58153 |
| 20MU10224NPS | 12/30/2021 | 542638 | 542638 | 1084816 | 1083984 | 1083984 | 4295.12 | 2 | 26231 |
| 20MU10227NPS | 1/1/2022 | 1011614 | 1011614 | 2020255 | 2016726 | 2016726 | 8102.73 | 2 | 47161 |
| 20MU10228NPS | 1/2/2022 | 1722207 | 1722207 | 3441121 | 3435737 | 3435737 | 13825.29 | 1 | 77037 |
| 20MU10229NPS | 1/2/2022 | 808170 | 808170 | 1613062 | 1608232 | 1608232 | 6423.11 | 1 | 21642 |
| 20MU10230NPS | 1/2/2022 | 1140071 | 1140071 | 2276445 | 2265909 | 2265909 | 8956.17 | 1 | 33739 |
| 20MU10231NPS | 1/2/2022 | 1006468 | 1006468 | 2009847 | 2006130 | 2006130 | 8055.54 | 1 | 28925 |
| 20MU10232NPS | 1/2/2022 | 814441 | 814441 | 1626892 | 1613212 | 1613212 | 6445.28 | 1 | 40131 |
| 20MU10234NPS | 1/3/2022 | 972891 | 972891 | 1943251 | 1926169 | 1926169 | 7594.39 | 1 | 98273 |
| 20MU10235NPS | 1/3/2022 | 1120956 | 1120956 | 2239612 | 2235660 | 2235660 | 8924.20 | 1 | 76336 |
| 20MU10238NPS | 1/4/2022 | 1580941 | 1580941 | 3158052 | 3150065 | 3150065 | 12640.27 | 2 | 66096 |
| 20MU10239NPS | 1/4/2022 | 1187528 | 1187528 | 2372319 | 2356373 | 2356373 | 9478.05 | 1 | 67629 |
| 20MU10240NPS | 1/4/2022 | 1076990 | 1076990 | 2150910 | 2143118 | 2143118 | 8657.50 | 24 | 61727 |
| 20MU10242NPS | 1/4/2022 | 1000900 | 1000900 | 1998860 | 1994560 | 1994560 | 7906.20 | 1 | 29937 |
| 20MU10243NPS | 1/4/2022 | 915926 | 915926 | 1828961 | 1816827 | 1816827 | 7148.09 | 1 | 98834 |
| 20MU10266NPS | 1/7/2022 | 550022 | 550022 | 1099541 | 1096459 | 1096459 | 4338.08 | 1 | 36815 |
| 20MU10267NPS | 1/7/2022 | 616280 | 616280 | 1231926 | 1229332 | 1229332 | 4861.71 | 1 | 34801 |
| 20MU10271NPS | 1/7/2022 | 292756 | 293547 | 583628 | 289181 | 289181 | 1143.56 | 4 | 4009 |
| 20MU10289NPS | 1/10/2022 | 338457 | 338457 | 676594 | 670990 | 670990 | 2655.86 | 16 | 17780 |
| 20MU10291NPS | 1/10/2022 | 512018 | 512018 | 1023399 | 1021833 | 1021833 | 4046.63 | 1 | 23333 |
| 20MU10294NPS | 1/10/2022 | 414816 | 414816 | 829191 | 828016 | 828016 | 3272.03 | 2 | 16361 |
| 20MU10331NPS | 1/15/2022 | 2658134 | 2659444 | 5298595 | 4748385 | 4748385 | 18147.21 | 1 | 45141 |
| 20MU10335NPS | 1/15/2022 | 2981549 | 2982128 | 5948407 | 5150173 | 5150173 | 19852.80 | 9 | 40520 |
| 20MU10363NPS | 1/18/2022 | 748890 | 748890 | 1495327 | 1418377 | 1418377 | 5590.61 | 1 | 21899 |
| 20MU10368NPS | 1/18/2022 | 1151561 | 1151561 | 2299579 | 2294917 | 2294917 | 9074.22 | 1 | 32372 |
| 20MU10369NPS | 1/18/2022 | 888776 | 888776 | 1774464 | 1771022 | 1771022 | 7056.44 | 1 | 22660 |
| 20MU10370NPS | 1/18/2022 | 546644 | 546644 | 1087161 | 279590 | 279590 | 1080.05 | 1 | 4757 |
| 20MU10373NPS | 1/18/2022 | 671703 | 671703 | 1341854 | 1255790 | 1255790 | 4986.37 | 5 | 17175 |
| 20MU10374NPS | 1/18/2022 | 1190451 | 1190451 | 2376843 | 2365409 | 2365409 | 9408.97 | 1 | 32755 |
| 20MU10377NPS | 1/19/2022 | 1067784 | 1067784 | 2131793 | 2056988 | 2056988 | 8116.79 | 1 | 32760 |
| 20MU10381NPS | 1/19/2022 | 1206583 | 1206583 | 2409094 | 2370503 | 2370503 | 9371.38 | 2 | 38696 |
| 20MU10384NPS | 1/19/2022 | 804344 | 804344 | 1605400 | 1560073 | 1560073 | 8184.65 | 1 | 41504 |
| 20MU10385NPS | 1/19/2022 | 929662 | 929662 | 1855642 | 1851892 | 1851892 | 7570.88 | 1 | 29430 |
| 20MU10387NPS | 1/19/2022 | 1029743 | 1029743 | 2055320 | 2041582 | 2041582 | 8356.51 | 1 | 36780 |
| 20MU10388NPS | 1/19/2022 | 1014204 | 1014204 | 2024672 | 1874798 | 1874798 | 7501.34 | 1 | 28806 |
| 20MU10390NPS | 1/20/2022 | 1142327 | 1142327 | 2280871 | 2276486 | 2276486 | 9179.08 | 1 | 32832 |
| 20MU10391NPS | 1/20/2022 | 617810 | 617810 | 1233079 | 1084786 | 1084786 | 4378.57 | 1 | 19220 |
| 20MU10392NPS | 1/20/2022 | 959061 | 959061 | 1914298 | 1818874 | 1818874 | 7353.18 | 1 | 31713 |
| 20MU10393NPS | 1/20/2022 | 1194528 | 1194528 | 2384859 | 2374999 | 2374999 | 9345.45 | 1 | 52146 |
| 20MU10394NPS | 1/20/2022 | 1217668 | 1217668 | 2432328 | 2422634 | 2422634 | 9938.04 | 1 | 40424 |
| 20MU10397NPS | 1/20/2022 | 836617 | 836617 | 1670932 | 1667852 | 1667852 | 6789.63 | 1 | 20726 |
| 20MU10398NPS | 1/20/2022 | 1070791 | 1070791 | 2137859 | 2035863 | 2035863 | 8005.58 | 1 | 53099 |
| 20MU10399NPS | 1/20/2022 | 1139313 | 1139313 | 2273308 | 2080936 | 2080936 | 8307.63 | 1 | 43392 |
| 20MU10401NPS | 1/20/2022 | 1338069 | 1338069 | 2670547 | 2659191 | 2659191 | 10602.29 | 1 | 60941 |
| 20MU10402NPS | 1/20/2022 | 1836333 | 1836333 | 3668142 | 3583953 | 3583953 | 14365.75 | 1 | 88980 |
| 20MU10405NPS | 1/20/2022 | 1193171 | 1193171 | 2382649 | 2304524 | 2304524 | 9088.59 | 1 | 34661 |
| 20MU10406NPS | 1/21/2022 | 993279 | 993279 | 1982912 | 1979159 | 1979159 | 7907.86 | 2 | 29494 |
| 20MU10410NPS | 1/21/2022 | 721977 | 721977 | 1442130 | 1437191 | 1437191 | 5731.22 | 1 | 22952 |
| 20MU10411NPS | 1/21/2022 | 677967 | 677967 | 1353027 | 1350082 | 1350082 | 5423.60 | 1 | 18680 |
| 20MU10432NPS | 1/22/2022 | 596414 | 596414 | 1190466 | 1188060 | 1188060 | 4768.75 | 9 | 17455 |
| 20MU10433NPS | 1/22/2022 | 668655 | 668655 | 1335590 | 1333122 | 1333122 | 5355.54 | 1 | 17511 |
| 20MU10434NPS | 1/23/2022 | 676420 | 676420 | 1350171 | 1197291 | 1197291 | 4739.02 | 1 | 29956 |
| 20MU10435NPS | 1/23/2022 | 719754 | 719754 | 1437405 | 1382431 | 1382431 | 5469.96 | 1 | 27220 |
| 20MU10436NPS | 1/23/2022 | 950361 | 950361 | 1894326 | 1435854 | 1435854 | 5673.16 | 11 | 29543 |
| 20MU10437NPS | 1/23/2022 | 1250878 | 1250878 | 2498844 | 2474343 | 2474343 | 9802.74 | 1 | 49269 |
| 20MU10439NPS | 1/23/2022 | 979357 | 979357 | 1955179 | 1942705 | 1942705 | 7694.31 | 1 | 42858 |
| 20MU10440NPS | 1/23/2022 | 612775 | 612775 | 1223428 | 1221216 | 1221216 | 4900.33 | 1 | 16683 |
| 20MU10441NPS | 1/23/2022 | 905673 | 905673 | 1808675 | 1802482 | 1802482 | 7148.69 | 1 | 41672 |
| 20MU10442NPS | 1/23/2022 | 636327 | 636327 | 1270259 | 1267739 | 1267739 | 5067.21 | 1 | 19678 |
| 20MU10443NPS | 1/23/2022 | 310042 | 310042 | 619233 | 618148 | 618148 | 2491.44 | 1 | 6861 |
| 20MU10444NPS | 1/23/2022 | 880028 | 880028 | 1758047 | 1754771 | 1754771 | 6973.33 | 1 | 34272 |
| 20MU10445NPS | 1/24/2022 | 607753 | 607753 | 1213146 | 1210505 | 1210505 | 4848.85 | 1 | 16139 |
| 20MU10446NPS | 1/24/2022 | 687883 | 687883 | 1373338 | 1371022 | 1371022 | 5521.51 | 2 | 18570 |
| 20MU10448NPS | 1/24/2022 | 665495 | 665495 | 1328423 | 1325917 | 1325917 | 5305.56 | 2 | 19663 |
| 20MU10449NPS | 1/24/2022 | 787027 | 787027 | 1570931 | 1509033 | 1509033 | 5971.46 | 5 | 26909 |
| 20MU10450NPS | 1/24/2022 | 797701 | 797701 | 1592694 | 1588766 | 1588766 | 6319.71 | 1 | 33552 |
| 20MU10451NPS | 1/24/2021 | 645894 | 645894 | 1289362 | 1286799 | 1286799 | 5162.45 | 1 | 17847 |
| 20MU10452NPS | 1/24/2022 | 502922 | 502922 | 1003468 | 1001288 | 1001288 | 4036.36 | 1 | 12925 |
| 20MU10453NPS | 1/24/2022 | 508103 | 508103 | 1013752 | 1011477 | 1011477 | 4059.63 | 1 | 14538 |
| 20MU10454NPS | 1/24/2022 | 628417 | 628417 | 1254731 | 1252270 | 1252270 | 5013.43 | 2 | 17667 |
| 20MU10455NPS | 1/24/2022 | 289394 | 289394 | 577586 | 576282 | 576282 | 2332.09 | 6 | 7356 |
| 20MU10456NPS | 1/24/2022 | 354891 | 354891 | 708869 | 707489 | 707489 | 2850.35 | 1 | 9690 |
| 20MU10457NPS | 1/24/2022 | 908174 | 908174 | 1813761 | 1767262 | 1767262 | 6989.44 | 1 | 36753 |
| 20MU10458NPS | 1/24/2022 | 699365 | 699365 | 1396182 | 1318115 | 1318115 | 5224.39 | 11 | 22827 |
| 20MU10460NPS | 1/24/2022 | 972319 | 972319 | 1941948 | 1929167 | 1929167 | 7651.95 | 1 | 37947 |
| 20MU10461NPS | 1/24/2022 | 890486 | 890486 | 1777914 | 1751396 | 1751396 | 6934.42 | 2 | 42746 |
| 20MU10462NPS | 1/24/2022 | 847698 | 847698 | 1691902 | 1686162 | 1686162 | 6700.51 | 1 | 34289 |
| 20MU10463NPS | 1/24/2022 | 578673 | 578673 | 1154910 | 1152501 | 1152501 | 4609.58 | 1 | 16426 |
| 20MU10464NPS | 1/24/2022 | 967450 | 967450 | 1931838 | 1927797 | 1927797 | 7659.38 | 2 | 38222 |
| 20MU10465NPS | 1/24/2022 | 1026259 | 1026259 | 2049368 | 2014285 | 2014285 | 7966.67 | 1 | 40785 |
| 20MU10466NPS | 1/24/2022 | 639020 | 639020 | 1275756 | 1273364 | 1273364 | 5071.17 | 1 | 19428 |
| 20MU10467NPS | 1/24/2022 | 940471 | 940471 | 1877062 | 1637333 | 1637333 | 6481.16 | 1 | 26090 |
| 20MU10468NPS | 1/24/2022 | 576862 | 576862 | 1151822 | 1149763 | 1149763 | 4577.69 | 1 | 18866 |
| 20MU10469NPS | 1/24/2022 | 643857 | 643857 | 1285318 | 1282584 | 1282584 | 5123.86 | 1 | 21707 |
| 20MU10471NPS | 1/24/2022 | 293182 | 293182 | 585412 | 584256 | 584256 | 2364.10 | 3 | 7353 |
| 20MU10473NPS | 1/24/2022 | 1004858 | 1004858 | 2006211 | 2001890 | 2001890 | 7964.84 | 2 | 34145 |
| 20MU10474NPS | 1/24/2022 | 427340 | 427340 | 853167 | 795526 | 795526 | 3156.84 | 1 | 21067 |
| 20MU10475NPS | 1/24/2022 | 530347 | 530347 | 1057345 | 1054329 | 1054329 | 4208.21 | 1 | 13896 |
| 20MU10476NPS | 1/24/2022 | 899924 | 899924 | 1796259 | 1792483 | 1792483 | 7132.14 | 1 | 25996 |
| 20MU10477NPS | 1/24/2022 | 987295 | 987295 | 1971780 | 1872091 | 1872091 | 7390.81 | 1 | 28204 |
| 20MU10478NPS | 1/25/2022 | 971317 | 971317 | 1939415 | 1924405 | 1924405 | 7631.14 | 1 | 37619 |
| 20MU10479NPS | 1/25/2022 | 717412 | 717412 | 1432437 | 1428763 | 1428763 | 5705.51 | 7 | 23748 |
| 20MU10480NPS | 1/25/2022 | 785186 | 785186 | 1566609 | 1476478 | 1476478 | 5846.64 | 1 | 25879 |
| 20MU10482NPS | 1/25/2022 | 751157 | 751157 | 1499607 | 1496254 | 1496254 | 5968.65 | 8 | 21308 |
| 20MU10483NPS | 1/25/2022 | 572647 | 572647 | 1143298 | 1140957 | 1140957 | 4557.25 | 6 | 20927 |
| 20MU10484NPS | 1/25/2022 | 776420 | 776421 | 1549444 | 1545520 | 1545520 | 6179.43 | 1 | 20452 |
| 20MU10485NPS | 1/25/2022 | 869334 | 869334 | 1735350 | 1729400 | 1729400 | 6861.11 | 1 | 29746 |
| 20MU10487NPS | 1/25/2022 | 863957 | 863957 | 1724728 | 1582196 | 1582196 | 6264.70 | 1 | 33682 |
| 20MU10488NPS | 1/25/2022 | 871222 | 871222 | 1738711 | 1705107 | 1705107 | 6766.39 | 8 | 33831 |
| 20MU10489NPS | 1/25/2022 | 879708 | 879708 | 1756376 | 1744172 | 1744172 | 6927.56 | 1 | 31704 |
| 20MU10490NPS | 1/25/2022 | 783865 | 783865 | 1564444 | 1559936 | 1559936 | 6235.96 | 2 | 20269 |
| 20MU10491NPS | 1/25/2022 | 807296 | 807296 | 1611176 | 1607148 | 1607148 | 6402.50 | 1 | 29638 |
| 20MU10492NPS | 1/25/2022 | 607796 | 607796 | 1212734 | 1210087 | 1210087 | 4848.06 | 1 | 16470 |
| 20MU10493NPS | 1/25/2022 | 536976 | 536976 | 1072247 | 1069977 | 1069977 | 4289.69 | 14 | 12648 |
| 20MU10494NPS | 1/25/2022 | 841472 | 841472 | 1679697 | 1675993 | 1675993 | 6676.63 | 1 | 23450 |
| 20MU10495NPS | 1/25/2022 | 1086545 | 1086545 | 2170514 | 2159735 | 2159735 | 8555.11 | 1 | 40664 |
| 20MU10496NPS | 1/25/2022 | 941194 | 941194 | 1879963 | 1874702 | 1874702 | 7442.58 | 1 | 38004 |
| 20MU10498NPS | 1/25/2022 | 489727 | 489727 | 976958 | 974603 | 974603 | 3906.48 | 6 | 12743 |
| 20MU10501NPS | 1/25/2022 | 900197 | 900197 | 1797205 | 1785338 | 1785338 | 7070.64 | 1 | 36063 |
| 20MU10502NPS | 1/25/2022 | 945813 | 945814 | 1887606 | 1763670 | 1763670 | 6973.18 | 1 | 28055 |
| 20MU10503NPS | 1/25/2022 | 818486 | 818486 | 1634931 | 1631918 | 1631918 | 6488.14 | 1 | 24242 |
| 20MU10504NPS | 1/25/2022 | 735694 | 735694 | 1468809 | 1458022 | 1458022 | 5789.45 | 1 | 24423 |
| 20MU10505NPS | 1/25/2022 | 834503 | 834503 | 1664840 | 1420548 | 1420548 | 5597.50 | 1 | 29552 |
| 20MU10506NPS | 1/25/2022 | 769076 | 769076 | 1535238 | 1531697 | 1531697 | 6101.04 | 1 | 22730 |
| 20MU10507NPS | 1/25/2022 | 538529 | 538529 | 1074239 | 770162 | 770162 | 3054.19 | 36 | 18368 |
| 20MU10508NPS | 1/25/2022 | 786097 | 786097 | 1569429 | 1560195 | 1560195 | 6187.81 | 1 | 28493 |
| 20MU10510NPS | 1/25/2022 | 584441 | 584441 | 1166696 | 1164354 | 1164354 | 4643.02 | 1 | 18194 |
| 20MU10511NPS | 1/25/2022 | 647112 | 647112 | 1292015 | 1289466 | 1289466 | 5166.36 | 1 | 18530 |
| 20MU10513NPS | 1/25/2022 | 825669 | 825669 | 1648296 | 1645154 | 1645154 | 6576.73 | 1 | 23565 |
| 20MU10514NPS | 1/25/2022 | 638104 | 638104 | 1273486 | 1270180 | 1270180 | 5072.82 | 1 | 17221 |
| 20MU10515NPS | 1/25/2022 | 983958 | 983958 | 1963850 | 1951793 | 1951793 | 7758.39 | 1 | 29995 |
| 20MU10517NPS | 1/26/2022 | 749646 | 749646 | 1496846 | 1489822 | 1489822 | 5929.78 | 1 | 25127 |
| 20MU10518NPS | 1/26/2022 | 720445 | 720445 | 1439149 | 1433597 | 1433597 | 5664.01 | 1 | 30545 |
| 20MU10519NPS | 1/26/2022 | 661645 | 661645 | 1320515 | 1317810 | 1317810 | 5255.61 | 1 | 17455 |
| 20MU10522NPS | 1/26/2022 | 677104 | 677104 | 1351745 | 1349076 | 1349076 | 5380.10 | 1 | 19517 |
| 20MU10523NPS | 1/26/2022 | 883250 | 883250 | 1763645 | 1756302 | 1756302 | 6981.15 | 1 | 29082 |
| 20MU10524NPS | 1/26/2022 | 783883 | 783883 | 1564767 | 1559707 | 1559707 | 6208.73 | 1 | 21135 |
| 20MU10531NPS | 1/26/2022 | 960096 | 960096 | 1916884 | 1896878 | 1896878 | 7500.94 | 1 | 28883 |
| 20MU10532NPS | 1/26/2022 | 495465 | 495465 | 988945 | 986861 | 986861 | 3966.85 | 3 | 13133 |
| 20MU10533NPS | 1/26/2022 | 631234 | 631234 | 1259806 | 1257083 | 1257083 | 5037.25 | 1 | 16230 |
| 20MU10534NPS | 1/26/2022 | 914140 | 914140 | 1825148 | 1821518 | 1821518 | 7241.02 | 2 | 29500 |
| 20MU10535NPS | 1/26/2022 | 935072 | 935072 | 1866838 | 1855201 | 1855201 | 7369.13 | 18 | 28291 |
| 20MU10537NPS | 1/26/2022 | 427226 | 427226 | 852841 | 849580 | 849580 | 3397.35 | 4 | 10927 |
| 20MU10540NPS | 1/26/2022 | 677108 | 677108 | 1351500 | 1348506 | 1348506 | 5386.85 | 1 | 18091 |
| 20MU10541NPS | 1/26/2022 | 722572 | 722572 | 1442319 | 1439131 | 1439131 | 5723.83 | 1 | 23548 |
| 20MU10543NPS | 1/26/2022 | 410645 | 410645 | 819832 | 817671 | 817671 | 3285.26 | 1 | 9858 |
| 20MU10544NPS | 1/26/2022 | 818657 | 818656 | 1634248 | 1629579 | 1629579 | 6475.18 | 1 | 28713 |
| 20MU10545NPS | 1/26/2022 | 280587 | 280587 | 560338 | 559359 | 559359 | 2257.60 | 5 | 6391 |
| 20MU10546NPS | 1/26/2022 | 637489 | 637489 | 1272932 | 1270339 | 1270339 | 5061.50 | 1 | 20131 |
| 20MU10547NPS | 1/26/2022 | 868550 | 868550 | 1729011 | 361901 | 361901 | 1423.31 | 20 | 13455 |
| 20MU10548NPS | 1/26/2022 | 1081575 | 1081575 | 2159831 | 1890863 | 1890863 | 7532.94 | 1 | 40017 |
| 20MU10549NPS | 1/26/2022 | 1405584 | 1405584 | 2807902 | 2784155 | 2784155 | 11196.47 | 1 | 54335 |
| 20MU10551NPS | 1/27/2022 | 1845592 | 1845592 | 3684750 | 3671281 | 3671281 | 14764.59 | 1 | 68225 |
| 20MU10553NPS | 1/27/2022 | 1041834 | 1041834 | 2079472 | 2068012 | 2068012 | 8354.58 | 1 | 41389 |
| 20MU10555NPS | 1/27/2022 | 2149069 | 2149069 | 4291925 | 4273644 | 4273644 | 17211.85 | 1 | 89269 |
| 20MU10556NPS | 1/26/2022 | 1543597 | 1543597 | 3082286 | 2927474 | 2927474 | 11684.54 | 1 | 76809 |
| 20MU10559NPS | 1/27/2022 | 1206687 | 1206687 | 2410205 | 2398826 | 2398826 | 9639.42 | 1 | 46697 |
| 20MU10560NPS | 1/27/2022 | 1048967 | 1048967 | 2094798 | 2075890 | 2075890 | 8344.00 | 1 | 49171 |
| 20MU10562NPS | 1/26/2022 | 1074710 | 1074710 | 2146324 | 2129725 | 2129725 | 8533.55 | 1 | 57545 |
| 20MU10563NPS | 1/27/2022 | 1346345 | 1346345 | 2688811 | 2672578 | 2672578 | 10725.44 | 1 | 61132 |
| 20MU10564NPS | 1/27/2022 | 1578245 | 1578245 | 3153117 | 3144150 | 3144150 | 12588.70 | 1 | 61033 |
| 20MU10565NPS | 1/27/2022 | 1188485 | 1188485 | 2373282 | 2366839 | 2366839 | 9541.73 | 20 | 47112 |
| 20MU10566NPS | 1/27/2022 | 388107 | 388107 | 773870 | 403026 | 403026 | 1623.03 | 1 | 9792 |
| 20MU10567NPS | 1/25/2022 | 1246430 | 1246429 | 2486703 | 1771058 | 1771058 | 7092.68 | 1 | 39441 |
| 20MU10569NPS | 1/26/2022 | 1037167 | 1037167 | 2070986 | 1762375 | 1762375 | 7067.12 | 1 | 42447 |
| 20MU10570NPS | 1/27/2022 | 1158746 | 1158746 | 2312826 | 1820039 | 1820039 | 7263.49 | 1 | 39549 |
| 20MU10571NPS | 1/27/2022 | 622581 | 622581 | 1242035 | 933592 | 933592 | 3724.03 | 1 | 24217 |
| 20MU10572NPS | 1/27/2022 | 942601 | 942600 | 1881881 | 1545564 | 1545564 | 6174.61 | 1 | 37971 |
| 20MU10573NPS | 1/27/2022 | 1001355 | 1001355 | 2000864 | 1941926 | 1941926 | 7776.89 | 1 | 44363 |
| 20MU10574NPS | 1/27/2022 | 351929 | 351929 | 701537 | 448160 | 448160 | 1807.14 | 3 | 13917 |
| 20MU10576NPS | 1/28/2021 | 1231083 | 1231083 | 2458685 | 2353856 | 2353856 | 9447.51 | 2 | 71012 |
| 20MU10577NPS | 1/27/2022 | 1050722 | 1050722 | 2099418 | 2080774 | 2080774 | 8407.18 | 1 | 36086 |
| 20MU10580NPS | 1/27/2022 | 1581305 | 1581305 | 3156571 | 2800141 | 2800141 | 11158.86 | 1 | 104367 |
| 20MU10582NPS | 1/27/2022 | 2005554 | 2005554 | 4003947 | 3849756 | 3849756 | 15515.02 | 1 | 169099 |
| 20MU10584NPS | 1/27/2022 | 552826 | 552826 | 1102292 | 565841 | 565841 | 2272.98 | 7 | 15847 |
| 20MU10585NPS | 1/27/2022 | 1127541 | 1127541 | 2241324 | 507361 | 507361 | 1967.55 | 1 | 21482 |
| 20MU10587NPS | 1/27/2022 | 1296666 | 1296664 | 2586695 | 2049346 | 2049346 | 8243.10 | 1 | 78667 |
| 20MU10588NPS | 1/27/2022 | 1520806 | 1520806 | 3038398 | 2902791 | 2902791 | 11619.78 | 1 | 118415 |
| 20MU10589NPS | 1/27/2022 | 1564969 | 1564968 | 3112227 | 737119 | 737119 | 2902.80 | 1 | 29171 |
| 20MU10590NPS | 1/27/2022 | 1833395 | 1833395 | 3660031 | 3644515 | 3644515 | 14776.62 | 1 | 118547 |
| 20MU10592NPS | 1/28/2022 | 123913 | 123913 | 246881 | 86227 | 86227 | 346.16 | 1 | 2718 |
| 20MU10596NPS | 1/28/2022 | 1512911 | 1512911 | 3020697 | 2920425 | 2920425 | 11656.86 | 1 | 129900 |
| 20MU10598NPS | 1/28/2022 | 1029349 | 1029349 | 2055254 | 1696029 | 1696029 | 6728.44 | 1 | 84970 |
| 20MU10599NPS | 1/28/2022 | 1163990 | 1163990 | 2322987 | 1999550 | 1999550 | 7940.44 | 1 | 69880 |
| 20MU10601NPS | 1/28/2022 | 371719 | 371719 | 740919 | 356850 | 356850 | 1431.79 | 1 | 16242 |
| 20MU10602NPS | 1/28/2022 | 1293719 | 1293719 | 2582291 | 2370486 | 2370486 | 9506.61 | 1 | 100213 |
| 20MU10603NPS | 1/28/2022 | 1370487 | 1370487 | 2737528 | 2679203 | 2679203 | 10828.33 | 1 | 106409 |
| 20MU10604NPS | 1/28/2022 | 1402526 | 1402526 | 2800582 | 2697572 | 2697572 | 10875.56 | 1 | 101439 |
| 20MU10605NPS | 1/28/2022 | 471536 | 471536 | 939339 | 253184 | 253184 | 1010.00 | 1 | 9194 |
| 20MU10608NPS | 1/28/2022 | 792148 | 792148 | 1581703 | 1351240 | 1351240 | 5390.25 | 1 | 52036 |
| 20MU10609NPS | 1/28/2022 | 458281 | 458281 | 914487 | 632571 | 632571 | 2526.80 | 9 | 21289 |
| 20MU10612NPS | 1/28/2022 | 1642331 | 1642331 | 3280013 | 3190338 | 3190338 | 12819.14 | 1 | 122835 |
| 20MU10615NPS | 1/28/2022 | 721425 | 721424 | 1438556 | 846579 | 846579 | 3385.70 | 1 | 24160 |
| 20MU10616NPS | 1/28/2022 | 1562831 | 1562831 | 3118772 | 2706525 | 2706525 | 10942.41 | 1 | 100386 |
| 20MU10617NPS | 1/28/2022 | 1066833 | 1066833 | 2129082 | 1796567 | 1796567 | 7216.13 | 1 | 62757 |
| 20MU10620NPS | 1/28/2022 | 1273297 | 1273297 | 2542686 | 1975258 | 1975258 | 7852.99 | 1 | 48127 |
| 20MU10621NPS | 1/28/2022 | 896403 | 896403 | 1790081 | 1381309 | 1381309 | 5560.06 | 1 | 55275 |
| 20MU10622NPS | 1/28/2022 | 222005 | 222005 | 442852 | 265321 | 265321 | 1088.10 | 1 | 9047 |
| 20MU10623NPS | 1/28/2022 | 769493 | 769493 | 1536513 | 1315680 | 1315680 | 5283.57 | 1 | 54346 |
| 20MU10625NPS | 1/28/2022 | 871304 | 871303 | 1733943 | 621607 | 621607 | 2457.76 | 1 | 29535 |
| 20MU10626NPS | 1/28/2022 | 1741447 | 1741447 | 3475861 | 3389098 | 3389098 | 13811.35 | 3 | 96990 |
| 20MU10627NPS | 1/28/2022 | 887615 | 887615 | 1773030 | 1630243 | 1630243 | 6507.26 | 1 | 84188 |
| 20MU10628NPS | 1/29/2022 | 2737119 | 2737119 | 5465541 | 5056645 | 5056645 | 20363.98 | 1 | 263561 |
| 20MU10632NPS | 1/29/2022 | 1037681 | 1037681 | 2070631 | 1520239 | 1520239 | 6138.12 | 1 | 67326 |
| 20MU10634NPS | 1/29/2022 | 814742 | 814742 | 1624336 | 987747 | 987747 | 3964.53 | 16 | 33572 |
| 20MU10635NPS | 1/29/2022 | 568096 | 568096 | 1133674 | 915350 | 915350 | 3736.34 | 1 | 24179 |
| 20MU10637NPS | 1/29/2022 | 205352 | 205352 | 408673 | 75660 | 75660 | 304.19 | 1 | 2055 |
| 20MU10639NPS | 1/29/2022 | 267149 | 267149 | 532395 | 350794 | 350794 | 1411.19 | 4 | 13925 |
| 20MU10640NPS | 1/29/2022 | 1498976 | 1498975 | 2987652 | 2426006 | 2426006 | 9856.96 | 1 | 87883 |
| 20MU10656NPS | 1/30/2022 | 892641 | 892641 | 1782351 | 1713084 | 1713084 | 6890.18 | 1 | 95191 |
| 20MU10657NPS | 1/30/2022 | 84017 | 84017 | 167203 | 94585 | 94585 | 389.16 | 10 | 3027 |
| 20MU10661NPS | 1/30/2022 | 1258625 | 1258625 | 2512530 | 1966640 | 1966640 | 7888.18 | 4 | 62135 |
| 20MU10664NPS | 1/30/2022 | 698382 | 698382 | 1394433 | 1205534 | 1205534 | 4811.82 | 1 | 103304 |
| 20MU10665NPS | 1/30/2022 | 891411 | 891411 | 1779772 | 1686545 | 1686545 | 6743.54 | 1 | 58948 |
| 20MU10666NPS | 1/30/2022 | 438712 | 438712 | 875509 | 586755 | 586755 | 2364.09 | 16 | 27312 |
| 20MU10668NPS | 1/30/2022 | 504903 | 504903 | 1007900 | 807956 | 807956 | 3264.11 | 1 | 20685 |
| 20MU10669NPS | 1/30/2022 | 129339 | 129339 | 257999 | 85171 | 85171 | 347.31 | 3 | 2224 |
| 20MU10672NPS | 1/30/2022 | 2309782 | 2309782 | 4613590 | 4249325 | 4249325 | 17055.21 | 1 | 174702 |
| 20MU10681NPS | 1/30/2022 | 1787978 | 1787978 | 3570300 | 3502020 | 3502020 | 14262.56 | 1 | 114617 |
| 20MU10683NPS | 1/30/2022 | 137533 | 137533 | 274100 | 112749 | 112749 | 466.97 | 2 | 3869 |
| 20MU10684NPS | 1/30/2022 | 1609181 | 1609181 | 3214840 | 3109784 | 3109784 | 12512.39 | 1 | 96397 |
| 20MU10685NPS | 1/30/2022 | 491031 | 491031 | 978505 | 654179 | 654179 | 2649.10 | 1 | 27485 |
| 20MU10686NPS | 1/30/2022 | 406476 | 406476 | 811274 | 560881 | 560881 | 2277.25 | 1 | 21222 |
| 20MU10687NPS | 1/30/2022 | 848086 | 848085 | 1686458 | 401828 | 401828 | 1595.88 | 1 | 10391 |
| 20MU10688NPS | 1/30/2022 | 795055 | 795055 | 1586713 | 1155426 | 1155426 | 4652.27 | 1 | 46538 |
| 20MU10690NPS | 1/30/2022 | 1227567 | 1227567 | 2448442 | 1744118 | 1744118 | 6911.44 | 1 | 50484 |
| 20MU10691NPS | 1/30/2022 | 37555 | 37555 | 74846 | 65154 | 65154 | 265.18 | 8 | 1347 |
| 20MU10692NPS | 1/30/2022 | 513698 | 513698 | 1020520 | 172973 | 172973 | 671.16 | 3 | 5589 |
| 20MU10695NPS | 2/1/2022 | 675725 | 675725 | 1348306 | 944730 | 944730 | 3779.86 | 1 | 22038 |
| 20MU10696NPS | 2/1/2022 | 272532 | 272532 | 542804 | 162541 | 162541 | 668.36 | 1 | 6279 |
| 20MU10697NPS | 2/1/2022 | 903240 | 903240 | 1803075 | 1568716 | 1568716 | 6329.67 | 2 | 51208 |
| 20MU10698NPS | 2/1/2022 | 1227300 | 1227300 | 2450670 | 2242016 | 2242016 | 8914.79 | 1 | 52985 |
| 20MU10699NPS | 1/31/2022 | 1413857 | 1413857 | 2822887 | 2405478 | 2405478 | 9731.81 | 1 | 62001 |
| 20MU10704NPS | 2/1/2022 | 1200057 | 1200057 | 2394257 | 2048551 | 2048551 | 8146.66 | 1 | 57736 |
| 20MU10705NPS | 2/1/2022 | 1398796 | 1398796 | 2793118 | 2628789 | 2628789 | 10508.83 | 1 | 56225 |
| 20MU10706NPS | 2/1/2022 | 1074572 | 1074572 | 2146496 | 2040327 | 2040327 | 8189.04 | 1 | 65081 |
| 20MU10707NPS | 2/1/2022 | 1411253 | 1411253 | 2816455 | 2585263 | 2585263 | 10479.51 | 3 | 56435 |
| 20MU10708NPS | 2/1/2022 | 1082566 | 1082566 | 2160644 | 1866075 | 1866075 | 7473.44 | 2 | 44593 |
| 20MU10711NPS | 2/1/2022 | 279374 | 279374 | 557038 | 382401 | 382401 | 1551.26 | 2 | 9790 |
| 20MU10718NPS | 2/3/2022 | 904696 | 904696 | 1808367 | 1803615 | 1803615 | 7197.92 | 1 | 34214 |
| 20MU10720NPS | 2/4/2022 | 1441830 | 1441830 | 2882075 | 2879066 | 2879066 | 11422.53 | 1 | 45979 |
| 20MU10721NPS | 2/4/2022 | 1672656 | 1672656 | 3343450 | 3298419 | 3298419 | 12930.64 | 1 | 73712 |
| 20MU10724NPS | 2/4/2022 | 849262 | 849262 | 1697683 | 1693302 | 1693302 | 6694.40 | 1 | 33408 |
| 20MU10725NPS | 2/4/2022 | 800872 | 800872 | 1600821 | 1574853 | 1574853 | 6184.72 | 1 | 32150 |
| 20MU10726NPS | 2/4/2022 | 457850 | 457850 | 915003 | 867423 | 867423 | 3428.21 | 1 | 14743 |
| 20MU10727NPS | 2/4/2022 | 785910 | 785910 | 1570620 | 1529625 | 1529625 | 6045.66 | 1 | 31875 |
| 20MU10734NPS | 2/6/2022 | 979430 | 979430 | 1957716 | 1946043 | 1946043 | 7682.16 | 130 | 36328 |
| 20MU10736NPS | 2/6/2022 | 801966 | 801966 | 1603106 | 1600740 | 1600740 | 6316.14 | 1 | 38134 |
| 20MU10738NPS | 2/7/2022 | 818808 | 818808 | 1636673 | 1634880 | 1634880 | 6481.18 | 1 | 32259 |
| 20MU10748NPS | 2/7/2022 | 1051337 | 1051337 | 2101482 | 2089417 | 2089417 | 8246.93 | 1 | 53192 |
| 20MU10766NPS | 2/9/2022 | NA | NA | NA | NA | NA | NA | NA | NA |
| 20MU10769NPS | 2/9/2022 | 564431 | 564431 | 1128227 | 1126170 | 1126170 | 4460.16 | 60 | 20495 |
| 20MU10774NPS | 2/10/2022 | 610770 | 610770 | 1220567 | 1166608 | 1166608 | 4606.82 | 1 | 24364 |
| 20MU10775NPS | 2/10/2022 | 609327 | 609327 | 1216996 | 931972 | 931972 | 3664.51 | 32 | 20411 |
| 20MU10776NPS | 2/10/2022 | 1403248 | 1403248 | 2805252 | 2799303 | 2799303 | 11042.64 | 2 | 68742 |
| 20MU10778NPS | 2/10/2022 | 800101 | 800101 | 1598896 | 1481961 | 1481961 | 5817.34 | 66 | 28651 |
| 20MU10780NPS | 2/10/2022 | 1050468 | 1050468 | 2099746 | 2050719 | 2050719 | 8073.22 | 4 | 44429 |
| 20MU10784NPS | 2/11/2022 | 1820560 | 1820560 | 3639478 | 3598291 | 3598291 | 14249.96 | 2 | 83288 |
| 20MU10793NPS | 2/13/2022 | 840456 | 840456 | 1679783 | 1582982 | 1582982 | 6221.57 | 1 | 29662 |
| 20MU10795NPS | 2/14/2022 | 1081654 | 1081654 | 2162242 | 2147815 | 2147815 | 8461.54 | 1 | 40263 |
| 20MU10799NPS | 2/14/2022 | 306498 | 306498 | 610304 | 110881 | 110881 | 421.28 | 1 | 3937 |
| 20MU10808NPS | 2/15/2022 | 592991 | 592991 | 1185061 | 1078195 | 1078195 | 4241.83 | 1 | 26076 |
| 20MU10810NPS | 2/15/2022 | 368075 | 368075 | 734447 | 373127 | 373127 | 1458.91 | 2 | 6238 |
| 20MU10817NPS | 2/16/2022 | 872521 | 872521 | 1743967 | 1692225 | 1692225 | 6630.28 | 1 | 38467 |
| 20MU10818NPS | 2/16/2022 | 581890 | 581890 | 1160566 | 666801 | 666801 | 2611.88 | 1 | 11543 |
| 20MU10821NPS | 2/16/2022 | 773242 | 773242 | 1545495 | 1492234 | 1492234 | 5857.85 | 1 | 32272 |
| 20MU10827NPS | 2/17/2022 | 590955 | 590955 | 1178518 | 469205 | 469205 | 1831.91 | 18 | 8107 |
| 20MU10829NPS | 2/18/2022 | 423613 | 423613 | 843818 | 269546 | 269546 | 1044.35 | 9 | 5533 |
| 20MU10831NPS | 2/18/2022 | 558214 | 558214 | 1115555 | 1056938 | 1056938 | 4270.09 | 2 | 26035 |
| 20MU10833NPS | 2/18/2022 | 720874 | 720874 | 1438163 | 936560 | 936560 | 3676.82 | 1 | 16041 |
| 20MU10834NPS | 2/18/2022 | 1161555 | 1161555 | 2321833 | 2285643 | 2285643 | 9001.16 | 1 | 59087 |
| 20MU10835NPS | 2/19/2022 | 1097742 | 1097742 | 2194435 | 2173864 | 2173864 | 8582.52 | 1 | 45103 |
| 20MU10836NPS | 2/19/2022 | 697153 | 697153 | 1392552 | 1134193 | 1134193 | 4456.39 | 1 | 21425 |
| 20MU10837NPS | 2/19/2022 | 777388 | 777388 | 1553848 | 1444179 | 1444179 | 5669.21 | 2 | 28549 |
| 20MU10838NPS | 2/19/2022 | 821601 | 821601 | 1642398 | 1635326 | 1635326 | 6450.74 | 3 | 36530 |
| 20MU10848NPS | 2/22/2022 | 524402 | 524402 | 1048255 | 993296 | 993296 | 3921.08 | 55 | 17168 |
| 20MU10851NPS | 2/22/2022 | 542770 | 542770 | 1084229 | 886307 | 886307 | 3464.96 | 1 | 21425 |
| 20MU10852NPS | 2/22/2022 | 700454 | 700454 | 1400239 | 1352750 | 1352750 | 5306.38 | 1 | 29085 |
| 20MU10853NPS | 2/22/2022 | 1084123 | 1084123 | 2166812 | 2156314 | 2156314 | 8476.30 | 9 | 37516 |
| 20MU10855NPS | 2/23/2022 | 1032491 | 1032491 | 2063715 | 2061321 | 2061321 | 8149.70 | 1 | 48842 |
| 20MU10860NPS | 2/24/2022 | 768017 | 768017 | 1535218 | 1495008 | 1495008 | 5877.89 | 56 | 31654 |
| 20MU10867NPS | 2/25/2022 | 444121 | 444121 | 887534 | 812088 | 812088 | 3210.61 | 67 | 18241 |
| 20MU10872NPS | 2/26/2022 | 1879761 | 1879761 | 3751239 | 596500 | 596500 | 2404.66 | 3 | 14612 |
| 20MU10875NPS | 2/26/2022 | 2036896 | 2036896 | 4070399 | 2732313 | 2732313 | 10950.95 | 20 | 79835 |
| 20MU10878NPS | 2/27/2022 | 1913944 | 1913944 | 3823234 | 1542001 | 1542001 | 6177.09 | 1 | 36642 |
| 20MU10882NPS | 2/27/2022 | 4811475 | 4811475 | 9610386 | 6643561 | 6643561 | 26456.31 | 4 | 237908 |
| 20MU10885NPS | 2/27/2022 | 3338096 | 3338096 | 6670346 | 6558127 | 6558127 | 26438.46 | 1 | 161830 |
| 20MU10891NPS | 3/1/2022 | 1476711 | 1476711 | 2950925 | 1294096 | 1294096 | 5198.36 | 16 | 44891 |
| 20MU10892NPS | 3/2/2022 | 1827498 | 1827498 | 3651719 | 3397155 | 3397155 | 13614.49 | 1 | 79942 |
| 20MU10894NPS | 3/2/2022 | 1583705 | 1583705 | 3163750 | 1009800 | 1009800 | 4107.85 | 1 | 26208 |
| 20MU10901NPS | 3/4/2022 | 1703234 | 1703234 | 3403700 | 3240756 | 3240756 | 13060.85 | 3 | 89467 |
| 20MU10903NPS | 3/5/2022 | 2172423 | 2172423 | 4339016 | 1905292 | 1905292 | 7477.97 | 1 | 123626 |
| 20MU10908NPS | 3/7/2022 | 1557804 | 1557804 | 3112237 | 3099160 | 3099160 | 12634.37 | 21 | 58401 |
| 20MU10925NPS | 3/11/2022 | 2540219 | 2540219 | 5075697 | 3188472 | 3188472 | 12819.39 | 1 | 111535 |
| 20MU10926NPS | 3/12/2022 | 1707516 | 1707516 | 3412579 | 2868117 | 2868117 | 11601.05 | 3 | 102161 |
| 20MU10939NPS | 3/17/2022 | 2590502 | 2590502 | 5176246 | 3010100 | 3010100 | 12065.23 | 2 | 101943 |
| 20MU10940NPS | 3/11/2022 | 2942821 | 2942821 | 5879457 | 2422722 | 2422722 | 9752.10 | 2 | 58693 |
| 20MU10941NPS | 3/18/2022 | 2725370 | 2725370 | 5443398 | 1969416 | 1969416 | 7881.11 | 1 | 69605 |
| 20MU10945NPS | 3/21/2022 | 2716598 | 2716598 | 5427082 | 5099834 | 5099834 | 20521.36 | 1 | 237850 |
| 20MU10950NPS | 3/24/2022 | 2776906 | 2776906 | 5548125 | 1439080 | 1439080 | 5771.62 | 2 | 49169 |
| 20MU10953NPS | 3/28/2022 | 2500715 | 2500715 | 4995559 | 715405 | 715405 | 2853.01 | 1 | 31833 |
| 20MU10954NPS | 3/28/2022 | 1970600 | 1970600 | 3938114 | 3920500 | 3920500 | 15849.83 | 1 | 71214 |
| 20MU10956NPS | 3/29/2022 | 2171856 | 2171856 | 4338614 | 1105106 | 1105106 | 4416.11 | 1 | 27639 |
| 20MU10958NPS | 3/29/2022 | 2230707 | 2230707 | 4456774 | 945380 | 945380 | 3780.04 | 1 | 20693 |
| 20MU10964NPS | 3/31/2022 | 1651124 | 1651124 | 3298524 | 2476322 | 2476322 | 9898.73 | 20 | 84188 |
| 20MU10968NPS | 4/27/2022 | 2307269 | 2307269 | 4611161 | 4210251 | 4210251 | 16785.17 | 1 | 129439 |
| 20MU10973NPS | 4/13/2022 | 2162076 | 2162076 | 4320091 | 3704954 | 3704954 | 14856.58 | 25 | 115315 |
| 20MU10974NPS | 4/18/2022 | 2641268 | 2641268 | 5277607 | 5180625 | 5180625 | 20832.17 | 1 | 138299 |
| 20MU10975NPS | 4/22/2022 | 4008842 | 4008842 | 8011158 | 7525199 | 7525199 | 29988.91 | 1 | 279549 |
| 20MU10976NPS | 4/2/2022 | 2067010 | 2067010 | 4128309 | 3996279 | 3996279 | 16261.54 | 1 | 154412 |
| 20MU10978NPS | 4/9/2022 | 2414913 | 2414913 | 4826430 | 4648592 | 4648592 | 18685.40 | 1 | 142484 |
| 20MU10979NPS | 4/30/2022 | 1764744 | 1764744 | 3526647 | 3210028 | 3210028 | 12918.60 | 1 | 86974 |
| 20MU10983NPS | 4/7/2022 | 1580058 | 1580058 | 3156899 | 1516528 | 1516528 | 6077.60 | 1 | 48724 |
| 20MU10987NPS | 4/23/2022 | 1892847 | 1892847 | 3781963 | 1139340 | 1139340 | 4641.97 | 13 | 30831 |
| 20MU10988NPS | 4/15/2022 | 1652396 | 1652396 | 3302280 | 3184944 | 3184944 | 12747.00 | 1 | 117710 |
| 20MU10991NPS | 4/2/2022 | 1616820 | 1616820 | 3230796 | 3221342 | 3221342 | 13044.03 | 18 | 47263 |
| 20MU10993NPS | 4/4/2022 | 2632670 | 2632670 | 5261806 | 5209911 | 5209911 | 20946.71 | 32 | 141594 |
| 20MU10994NPS | 4/6/2022 | 2571025 | 2571025 | 5138459 | 5027319 | 5027319 | 20098.07 | 18 | 184711 |
| 20MU10997NPS | 4/5/2022 | 2638591 | 2638591 | 5270751 | 2616917 | 2616917 | 10461.79 | 1 | 67276 |
| 20MU10998NPS | 4/8/2022 | 1442105 | 1442105 | 2882541 | 2817003 | 2817003 | 11295.59 | 1 | 114800 |
| 20MU10999NPS | 4/9/2022 | 1877921 | 1877921 | 3752714 | 3603053 | 3603053 | 14423.55 | 1 | 103966 |
| 20MU11000NPS | 4/1/2022 | 1402439 | 1402439 | 2802818 | 2141464 | 2141464 | 8539.08 | 11 | 79499 |
| 20MU11002NPS | 4/2/2022 | 2480375 | 2480375 | 4957506 | 4599119 | 4599119 | 18345.89 | 1 | 146390 |
| 20MU11006NPS | 4/18/2022 | 2855355 | 2855355 | 5706970 | 5229651 | 5229651 | 20928.53 | 2 | 158489 |
| 20MU11007NPS | 4/18/2022 | 6221901 | 6221901 | 12435728 | 12290878 | 12290878 | 49015.56 | 1 | 333759 |
| 20MU11009NPS | 4/16/2022 | 2508007 | 2508007 | 5012323 | 4824881 | 4824881 | 19357.75 | 1 | 154365 |
| 20MU11012NPS | 4/1/2022 | 2523834 | 2523834 | 5044486 | 4485703 | 4485703 | 18035.86 | 1 | 129508 |
| 20MU11015NPS | 4/25/2022 | 1670180 | 1670180 | 3338414 | 3014466 | 3014466 | 12006.81 | 4 | 124405 |
| 20MU11016NPS | 4/12/2022 | 2611393 | 2611393 | 5218104 | 3614032 | 3614032 | 14434.80 | 23 | 117965 |
| 20MU11020NPS | 4/2/2022 | 2237625 | 2237625 | 4471802 | 4435003 | 4435003 | 18061.16 | 1 | 83980 |
| 20MU11021NPS | 4/13/2022 | 1607030 | 1607030 | 3211362 | 3171320 | 3171320 | 12818.31 | 11 | 83236 |
| 20MU11023NPS | 4/8/2022 | 1711353 | 1711353 | 3420279 | 3049447 | 3049447 | 12218.08 | 1 | 97422 |
| 20MU11025NPS | 4/18/2022 | 1388673 | 1388673 | 2772984 | 547319 | 547319 | 2188.04 | 1 | 15603 |
| 20MU11026NPS | 4/18/2022 | 2123213 | 2123213 | 4241981 | 1293042 | 1293042 | 5206.80 | 1 | 41659 |
| 20MU11031NPS | 6/2/2022 | 1794190 | 1794190 | 3584310 | 970489 | 970489 | 3883.81 | 1 | 38631 |
| 20MU11033NPS | 6/2/2022 | 2124930 | 2124930 | 4246789 | 3780606 | 3780606 | 15208.61 | 1 | 105470 |
| 20MU11034NPS | 6/2/2022 | 1840543 | 1840543 | 3677889 | 3664283 | 3664283 | 14791.57 | 1 | 79503 |
| 20MU11036NPS | 6/2/2022 | 1860622 | 1860622 | 3716704 | 3694016 | 3694016 | 14948.81 | 1 | 109410 |
| 20MU11038NPS | 6/2/2022 | 1553376 | 1553376 | 3103977 | 2845155 | 2845155 | 11322.74 | 1 | 103184 |
| 20MU11039NPS | 6/2/2022 | 1336407 | 1336407 | 2670640 | 2649977 | 2649977 | 10762.96 | 22 | 74639 |
| 20MU11041NPS | 6/3/2022 | 2104028 | 2104028 | 4199952 | 806719 | 806719 | 3080.57 | 1 | 16568 |
| 20MU11044NPS | 6/3/2022 | 380810 | 380810 | 760946 | 750916 | 750916 | 2968.53 | 1 | 11470 |
| 20MU11046NPS | 6/3/2022 | 379606 | 379606 | 758149 | 408007 | 408007 | 1605.83 | 45 | 11136 |
| 20MU11053NPS | 6/7/2022 | 254580 | 254580 | 508626 | 506214 | 506214 | 2005.22 | 1 | 9253 |
| 20MU11057NPS | 6/7/2022 | 460363 | 460363 | 920152 | 906925 | 906925 | 3594.87 | 79 | 14852 |
| 20MU11058NPS | 6/8/2022 | 592471 | 592471 | 1182779 | 776146 | 776146 | 3036.57 | 1 | 18915 |
| 20MU11059NPS | 6/8/2022 | 522857 | 522857 | 1044830 | 899423 | 899423 | 3557.54 | 115 | 18136 |
| 20MU11060NPS | 6/8/2022 | 565515 | 565515 | 1130201 | 1078498 | 1078498 | 4275.74 | 91 | 19519 |
| 20MU11061NPS | 6/9/2022 | 344774 | 344774 | 688924 | 668533 | 668533 | 2647.39 | 6 | 14849 |
| 20MU11062NPS | 6/9/2022 | 342869 | 342869 | 685189 | 683656 | 683656 | 2718.89 | 8 | 9406 |
| 20MU11063NPS | 6/10/2022 | 472563 | 472563 | 944439 | 931749 | 931749 | 3686.71 | 75 | 18569 |
| 20MU11064NPS | 6/12/2022 | 719359 | 719359 | 1437079 | 967922 | 967922 | 3820.00 | 79 | 18114 |
| 20MU11067NPS | 6/13/2022 | 606605 | 606605 | 1212105 | 909208 | 909208 | 3610.18 | 151 | 14341 |
| 20MU11068NPS | 6/13/2022 | 447081 | 447081 | 893405 | 890985 | 890985 | 3545.17 | 1 | 13473 |
| 20MU11070NPS | 6/13/2022 | 257735 | 257735 | 515021 | 514235 | 514235 | 2060.67 | 16 | 6175 |
| 20MU11071NPS | 6/14/2022 | 456772 | 456772 | 913108 | 908053 | 908053 | 3592.17 | 117 | 25077 |
| 20MU11073NPS | 6/15/2022 | 240813 | 240813 | 481348 | 474971 | 474971 | 1875.11 | 49 | 13514 |
| 20MU11074NPS | 6/15/2022 | 445797 | 445797 | 890963 | 889737 | 889737 | 3621.23 | 64 | 10809 |
| 20MU11075NPS | 6/15/2022 | 394817 | 394817 | 789121 | 787930 | 787930 | 3186.43 | 44 | 23380 |
| 20MU11077NPS | 6/15/2022 | 599923 | 599923 | 1199377 | 1198047 | 1198047 | 4668.83 | 1 | 56612 |
| 20MU11078NPS | 6/15/2022 | 400661 | 400661 | 800915 | 800156 | 800156 | 3135.59 | 2 | 12407 |
| 20MU11079NPS | 6/16/2022 | 731357 | 731357 | 1462020 | 1460680 | 1460680 | 5756.57 | 11 | 29333 |
| 20MU11080NPS | 6/16/2022 | 263624 | 263624 | 526974 | 521316 | 521316 | 2065.53 | 3 | 15838 |
| 20MU11081NPS | 6/16/2022 | 504626 | 504626 | 1008749 | 1007922 | 1007922 | 4009.31 | 9 | 29303 |
| 20MU11082NPS | 6/17/2022 | 545409 | 545409 | 1090447 | 1086727 | 1086727 | 4253.61 | 1 | 47690 |
| 20MU11087NPS | 6/18/2022 | 523649 | 523649 | 1046900 | 1045901 | 1045901 | 4109.02 | 5 | 38636 |
| 20MU11088NPS | 6/18/2022 | 567702 | 567702 | 1134838 | 1133864 | 1133864 | 4507.39 | 8 | 27426 |
| 20MU11090NPS | 6/21/2022 | 665407 | 665407 | 1330245 | 1328653 | 1328653 | 5248.73 | 1 | 57178 |
| 20MU11091NPS | 6/22/2022 | 324619 | 324619 | 648855 | 647956 | 647956 | 2549.75 | 1 | 9748 |
| 20MU11092NPS | 6/22/2022 | 627386 | 627386 | 1254306 | 1252871 | 1252871 | 4945.71 | 4 | 51462 |
| 20MU11093NPS | 6/22/2022 | 582558 | 582558 | 1164479 | 1161941 | 1161941 | 4594.51 | 6 | 42733 |
| 20MU11094NPS | 6/22/2022 | 549527 | 549527 | 1098569 | 1097465 | 1097465 | 4306.26 | 6 | 18620 |
| 20MU11098NPS | 6/23/2022 | 728566 | 728566 | 1456567 | 1455373 | 1455373 | 5723.63 | 5 | 28659 |
| 20MU11100NPS | 6/23/2022 | 438954 | 438954 | 877295 | 866231 | 866231 | 3401.62 | 1 | 44757 |
| 20MU11101NPS | 6/23/2022 | 276069 | 276069 | 551734 | 551159 | 551159 | 2238.40 | 9 | 6467 |
| 20MU11103NPS | 6/23/2022 | 604089 | 604089 | 1207739 | 1206831 | 1206831 | 4777.40 | 5 | 25400 |
| 20MU11104NPS | 6/23/2022 | 632010 | 632010 | 1263393 | 1262182 | 1262182 | 4983.03 | 1 | 18828 |
| 20MU11105NPS | 6/24/2022 | 493777 | 493777 | 987050 | 967976 | 967976 | 3827.94 | 2 | 34950 |
| 20MU11106NPS | 6/24/2022 | 395228 | 395228 | 789881 | 788850 | 788850 | 3133.66 | 13 | 13666 |
| 20MU11107NPS | 6/26/2022 | 662277 | 662277 | 1323913 | 1322769 | 1322769 | 5200.46 | 15 | 27122 |
| 20MU11109NPS | 6/27/2022 | 307294 | 307294 | 614085 | 613254 | 613254 | 2508.04 | 19 | 7085 |
| 20MU11110NPS | 6/27/2022 | 532573 | 532573 | 1064773 | 1062584 | 1062584 | 4167.38 | 6 | 53229 |
| 20MU11111NPS | 6/27/2022 | 384971 | 384971 | 769469 | 748392 | 748392 | 2958.35 | 1 | 19266 |
| 20MU11112NPS | 6/27/2022 | 637529 | 637529 | 1274556 | 1272621 | 1272621 | 5030.11 | 4 | 50455 |
| 20MU11113NPS | 6/27/2022 | 562947 | 562947 | 1125370 | 1124191 | 1124191 | 4412.40 | 4 | 21813 |
| 20MU11114NPS | 6/27/2022 | 540288 | 540288 | 1080231 | 1079088 | 1079088 | 4238.44 | 2 | 33416 |
| 20MU11117NPS | 6/28/2022 | 493633 | 493633 | 986746 | 985890 | 985890 | 3937.43 | 9 | 28494 |
| 20MU11118NPS | 6/28/2022 | 608880 | 608880 | 1217105 | 1215819 | 1215819 | 4771.87 | 1 | 16563 |
| 20MU11120NPS | 6/28/2022 | 503804 | 503804 | 1007213 | 1006351 | 1006351 | 3984.37 | 1 | 21852 |
| 20MU11123NPS | 6/28/2022 | 239468 | 239468 | 478626 | 477816 | 477816 | 1886.85 | 5 | 8109 |
| 20MU11125NPS | 6/28/2022 | 601251 | 601251 | 1201934 | 1200810 | 1200810 | 4733.68 | 1 | 17066 |
| 20MU11126NPS | 6/28/2022 | 490321 | 490321 | 980168 | 969035 | 969035 | 3831.93 | 1 | 25006 |
| 20MU11127NPS | 6/28/2022 | 628623 | 628623 | 1256490 | 1255295 | 1255295 | 4994.98 | 1 | 17607 |
| 20MU11128NPS | 6/29/2022 | 342162 | 342162 | 683957 | 674103 | 674103 | 2660.54 | 3 | 29052 |
| 20MU11129NPS | 6/29/2022 | 681213 | 681213 | 1361835 | 1360733 | 1360733 | 5341.98 | 2 | 47607 |
| 20MU11130NPS | 6/29/2022 | 580565 | 580565 | 1160599 | 1159681 | 1159681 | 4572.94 | 5 | 43145 |
| 20MU11131NPS | 6/29/2022 | 709623 | 709623 | 1418528 | 1384624 | 1384624 | 5477.30 | 3 | 56901 |
| 20MU11133NPS | 6/29/2022 | 586461 | 586461 | 1172530 | 1171028 | 1171028 | 4579.98 | 3 | 49452 |
| 20MU11134NPS | 6/29/2022 | 664618 | 664618 | 1328702 | 1326789 | 1326789 | 5253.93 | 1 | 53215 |
| 20MU11138NPS | 6/29/2022 | 539308 | 539308 | 1078120 | 1077193 | 1077193 | 4237.13 | 4 | 18079 |
| 20MU11139NPS | 6/29/2022 | 755898 | 755959 | 1511192 | 1505543 | 1505543 | 5919.46 | 6 | 50272 |
| 20MU11141NPS | 6/29/2022 | 644835 | 644835 | 1289058 | 1287637 | 1287637 | 5097.03 | 2 | 47046 |
| 20MU11142NPS | 6/30/2022 | 806235 | 806235 | 1611838 | 1610616 | 1610616 | 6385.83 | 10 | 39791 |
| 20MU11143NPS | 6/30/2022 | 865250 | 865250 | 1729468 | 1726850 | 1726850 | 6843.46 | 7 | 53981 |
| 20MU11144NPS | 6/30/2022 | 623949 | 623949 | 1247341 | 1246358 | 1246358 | 4902.52 | 9 | 25105 |
| 20MU11145NPS | 6/30/2022 | 567410 | 567410 | 1134303 | 1124537 | 1124537 | 4425.36 | 4 | 51674 |
| 20MU11148NPS | 7/1/2022 | 437202 | 437202 | 873797 | 872596 | 872596 | 3478.07 | 15 | 10637 |
| 20MU11149NPS | 7/1/2022 | 575771 | 575771 | 1150995 | 1149681 | 1149681 | 4556.26 | 8 | 18179 |
| 20MU11150NPS | 7/1/2022 | 542709 | 542709 | 1084793 | 1083868 | 1083868 | 4316.58 | 7 | 24711 |
| 20MU11151NPS | 7/1/2022 | 569511 | 569511 | 1138514 | 1137554 | 1137554 | 4441.87 | 1 | 21544 |
| 20MU11152NPS | 7/1/2022 | 560095 | 560095 | 1119545 | 1118419 | 1118419 | 4450.26 | 5 | 14100 |
| 20MU11153NPS | 7/1/2022 | 660505 | 660505 | 1320395 | 1316366 | 1316366 | 5186.96 | 2 | 53326 |
| 20MU11158NPS | 7/3/2022 | 649666 | 649666 | 1298777 | 1296824 | 1296824 | 5107.68 | 2 | 50607 |
| 20MU11159NPS | 7/4/2022 | 378728 | 378728 | 757027 | 745337 | 745337 | 2933.09 | 2 | 28946 |
| 20MU11160NPS | 7/4/2022 | 728769 | 728769 | 1456792 | 1455594 | 1455594 | 5743.67 | 10 | 28755 |
| 20MU11164NPS | 7/5/2022 | 626116 | 626116 | 1251785 | 1247716 | 1247716 | 4898.12 | 1 | 49072 |
| 20MU11165NPS | 7/5/2022 | 634963 | 634963 | 1269362 | 1264337 | 1264337 | 4998.19 | 5 | 56224 |
| 20MU11166NPS | 7/5/2022 | 607164 | 607164 | 1213794 | 1212504 | 1212504 | 4740.74 | 2 | 39777 |
| 20MU11168NPS | 7/5/2022 | 302074 | 302074 | 603714 | 602907 | 602907 | 2426.63 | 18 | 6998 |
| 20MU11169NPS | 7/5/2022 | 580877 | 580877 | 1161120 | 1159824 | 1159824 | 4552.17 | 6 | 17328 |
| 20MU11171NPS | 7/6/2022 | 284883 | 284883 | 569378 | 568760 | 568760 | 2319.72 | 4 | 7044 |
| 20MU11172NPS | 7/6/2022 | 588987 | 588987 | 1177356 | 1164521 | 1164521 | 4590.56 | 6 | 48854 |
| 20MU11173NPS | 7/6/2022 | 334209 | 334209 | 667811 | 666924 | 666924 | 2654.00 | 12 | 8937 |
| 20MU11174NPS | 7/6/2022 | 483410 | 483410 | 966351 | 948649 | 948649 | 3752.85 | 2 | 49331 |
| 20MU11175NPS | 7/6/2022 | 396577 | 396577 | 792710 | 791802 | 791802 | 3125.50 | 4 | 12213 |
| 20MU11176NPS | 7/6/2022 | 624851 | 624851 | 1249192 | 1247404 | 1247404 | 4914.79 | 9 | 48538 |
| 20MU11177NPS | 7/6/2022 | 517198 | 517198 | 1033960 | 1033044 | 1033044 | 4088.40 | 1 | 32919 |
| 20MU11178NPS | 7/6/2022 | 734529 | 734529 | 1468436 | 1466808 | 1466808 | 5770.79 | 6 | 54440 |
| 20MU11180NPS | 7/7/2022 | 540661 | 540661 | 1080801 | 1079481 | 1079481 | 4196.09 | 1 | 16957 |
| 20MU11181NPS | 7/7/2022 | 703635 | 703635 | 1406692 | 1405409 | 1405409 | 5486.81 | 1 | 33737 |
| 20MU11182NPS | 7/7/2022 | 500547 | 500547 | 1000551 | 999238 | 999238 | 3973.48 | 1 | 36361 |
| 20MU11184NPS | 7/7/2022 | 613639 | 613639 | 1226690 | 1225570 | 1225570 | 4855.19 | 1 | 21779 |
| 20MU11185NPS | 7/7/2022 | 318661 | 318661 | 636829 | 635727 | 635727 | 2506.65 | 1 | 10063 |
| 20MU11186NPS | 7/8/2022 | 625526 | 625526 | 1250412 | 1248959 | 1248959 | 4864.17 | 3 | 20019 |
| 20MU11187NPS | 7/8/2022 | 952371 | 952371 | 1903932 | 1847400 | 1847400 | 7236.42 | 1 | 47903 |
| 20MU11191NPS | 7/10/2022 | 634465 | 634465 | 1268057 | 1266822 | 1266822 | 5052.79 | 1 | 22130 |
| 20MU11192NPS | 7/11/2022 | 443068 | 443068 | 885701 | 826084 | 826084 | 3246.80 | 54 | 18866 |
| 20MU11193NPS | 7/11/2022 | 443471 | 443471 | 886261 | 885404 | 885404 | 3507.39 | 2 | 14949 |
| 20MU11194NPS | 7/11/2022 | 394254 | 394254 | 787886 | 622529 | 622529 | 2471.03 | 4 | 13222 |
| 20MU11197NPS | 7/12/2022 | 363598 | 363598 | 726769 | 725909 | 725909 | 2907.16 | 1 | 9465 |
| 20MU11198NPS | 7/12/2022 | 768345 | 768345 | 1536036 | 1534822 | 1534822 | 6104.13 | 1 | 40109 |
| 20MU11200NPS | 7/12/2022 | 401226 | 401226 | 801944 | 801090 | 801090 | 3155.08 | 7 | 11211 |
| 20MU11201NPS | 7/13/2022 | 464017 | 464017 | 927543 | 926399 | 926399 | 3673.90 | 1 | 13282 |
| 20MU11202NPS | 7/13/2022 | 427795 | 427795 | 855111 | 827101 | 827101 | 3256.26 | 38 | 37944 |
| 20MU11204NPS | 7/13/2022 | 699751 | 699751 | 1398680 | 1397416 | 1397416 | 5589.22 | 21 | 23999 |
| 20MU11207NPS | 7/14/2022 | 872286 | 872286 | 1743201 | 1741598 | 1741598 | 6968.62 | 98 | 29553 |
| 20MU11208NPS | 7/14/2022 | 385622 | 385622 | 770730 | 769876 | 769876 | 3093.36 | 1 | 9716 |
| 20MU11209NPS | 7/15/2022 | 391167 | 391167 | 781934 | 781051 | 781051 | 3022.49 | 2 | 11799 |
| 20MU11211NPS | 7/15/2022 | 644239 | 644239 | 1287723 | 1286388 | 1286388 | 5114.41 | 1 | 22567 |
| 20MU11213NPS | 7/16/2022 | 515038 | 515038 | 1029368 | 1028301 | 1028301 | 4098.16 | 10 | 13010 |
| 20MU11215NPS | 7/16/2022 | 550000 | 550000 | 1099091 | 878425 | 878425 | 3469.95 | 37 | 19857 |
| 20MU11216NPS | 7/16/2022 | 528819 | 528819 | 1057037 | 996731 | 996731 | 3921.05 | 1 | 34656 |
| 20MU11217NPS | 7/17/2022 | 425202 | 425202 | 849965 | 848968 | 848968 | 3334.39 | 1 | 11813 |
| 20MU11220NPS | 7/17/2022 | 566656 | 566656 | 1132558 | 1131352 | 1131352 | 4504.09 | 52 | 32272 |
| 20MU11222NPS | 7/17/2022 | 667760 | 667760 | 1334935 | 1333492 | 1333492 | 5254.05 | 4 | 19188 |
| 20MU11223NPS | 7/18/2022 | 557689 | 557689 | 1114986 | 1113984 | 1113984 | 4372.27 | 2 | 28539 |
| 20MU11225NPS | 7/18/2022 | 380498 | 380498 | 760568 | 759697 | 759697 | 3002.48 | 5 | 20227 |
| 20MU11226NPS | 7/18/2022 | 800971 | 800971 | 1601189 | 1598530 | 1598530 | 6353.21 | 1 | 37746 |
| 20MU11228NPS | 7/19/2022 | 284645 | 284645 | 568841 | 512019 | 512019 | 2024.52 | 1 | 16063 |
| 20MU11230NPS | 7/20/2022 | 899186 | 899186 | 1797245 | 1793399 | 1793399 | 7140.20 | 57 | 36697 |
| 20MU11231NPS | 7/20/2022 | 634489 | 634489 | 1268221 | 1267157 | 1267157 | 5042.63 | 26 | 25118 |
| 20MU11233NPS | 7/20/2022 | 863078 | 863078 | 1725440 | 1722808 | 1722808 | 6651.94 | 1 | 50191 |
| 20MU11235NPS | 7/21/2022 | 368719 | 368719 | 736996 | 736424 | 736424 | 2926.27 | 1 | 12744 |
| 20MU11236NPS | 7/21/2022 | 615316 | 615316 | 1229922 | 1210272 | 1210272 | 4773.64 | 71 | 31106 |
| 20MU11239NPS | 7/22/2022 | 830473 | 830473 | 1660134 | 1658833 | 1658833 | 6594.42 | 44 | 35518 |
| 20MU11240NPS | 7/22/2022 | 545713 | 545713 | 1090865 | 1065626 | 1065626 | 4197.80 | 88 | 18475 |
| 20MU11244NPS | 7/24/2022 | 452025 | 452025 | 903578 | 890983 | 890983 | 3512.38 | 49 | 21156 |
| 20MU11246NPS | 7/25/2022 | 603431 | 603431 | 1206204 | 1137481 | 1137481 | 4459.92 | 1 | 45602 |
| 20MU11247NPS | 7/25/2021 | 538640 | 538640 | 1076687 | 1075027 | 1075027 | 4258.21 | 1 | 21151 |
| 20MU11250NPS | 7/25/2022 | 596131 | 596131 | 1191584 | 1169174 | 1169174 | 4607.22 | 51 | 43088 |
| 20MU11251NPS | 7/25/2022 | 548090 | 548090 | 1095659 | 1094393 | 1094393 | 4315.28 | 1 | 17604 |
| 20MU11252NPS | 7/25/2022 | 659530 | 659530 | 1318258 | 1316830 | 1316830 | 5267.77 | 12 | 18363 |
| 20MU11253NPS | 7/26/2022 | 795392 | 795392 | 1590043 | 1580233 | 1580233 | 6242.54 | 83 | 35468 |
| 20MU11254NPS | 7/26/2022 | 718006 | 718006 | 1435119 | 1272830 | 1272830 | 5060.10 | 1 | 28348 |
| 20MU11258NPS | 7/26/2022 | 600894 | 600894 | 1201092 | 1199899 | 1199899 | 4778.78 | 47 | 31347 |
| 20MU11260NPS | 7/27/2022 | 348768 | 348768 | 697123 | 646701 | 646701 | 2562.67 | 38 | 13218 |
| 20MU11262NPS | 7/28/2022 | 862785 | 862785 | 1724889 | 1723393 | 1723393 | 6818.25 | 1 | 37493 |
| 20MU11263NPS | 7/28/2022 | 561321 | 561321 | 1122053 | 1120960 | 1120960 | 4453.78 | 49 | 24108 |
| 20MU11264NPS | 7/28/2022 | 606644 | 606644 | 1212673 | 1211625 | 1211625 | 4797.13 | 1 | 23257 |
| 20MU11265NPS | 7/28/2022 | 373150 | 373150 | 745851 | 744941 | 744941 | 2964.68 | 2 | 9926 |
| 20MU11267NPS | 7/28/2022 | 589304 | 589304 | 1177874 | 1176875 | 1176875 | 4657.31 | 8 | 19711 |
| 20MU11269NPS | 7/29/2022 | 606884 | 606884 | 1213054 | 1211900 | 1211900 | 4811.24 | 1 | 21794 |
| 20MU11271NPS | 7/29/2022 | 707282 | 707282 | 1413798 | 1412513 | 1412513 | 5597.26 | 5 | 20531 |
| 20MU11272NPS | 7/29/2022 | 436445 | 436445 | 872371 | 858502 | 858502 | 3390.02 | 7 | 18649 |
| 20MU11273NPS | 7/29/2022 | 332289 | 332289 | 664168 | 663370 | 663370 | 2652.62 | 3 | 9243 |
| 20MU11274NPS | 7/29/2022 | 495906 | 495906 | 991291 | 990290 | 990290 | 3925.15 | 1 | 32598 |
| 20MU11277NPS | 7/30/2022 | 735251 | 735251 | 1469774 | 1467032 | 1467032 | 5819.66 | 1 | 37777 |
| 20MU11278NPS | 7/30/2022 | 425501 | 425501 | 850607 | 849556 | 849556 | 3293.10 | 1 | 17167 |
| 20MU11279NPS | 7/31/2022 | 493947 | 493947 | 987095 | 884781 | 884781 | 3473.92 | 76 | 19599 |
| 20MU11280NPS | 7/31/2022 | 423050 | 423050 | 845522 | 844727 | 844727 | 3357.86 | 8 | 13289 |
| 20MU11284NPS | 7/31/2022 | 588442 | 588442 | 1176134 | 1174837 | 1174837 | 4659.95 | 1 | 16969 |
| 20MU11285NPS | 7/31/2022 | 367440 | 367440 | 734467 | 733387 | 733387 | 2913.25 | 5 | 10641 |
| 20MU11286NPS | 7/31/2022 | 527013 | 527013 | 1053470 | 1052359 | 1052359 | 4141.41 | 6 | 17508 |
| 20MU11288NPS | 7/31/2022 | 649768 | 649768 | 1298696 | 1297194 | 1297194 | 5181.30 | 24 | 18041 |
| 20MU11289NPS | 7/31/2022 | 688994 | 688994 | 1377292 | 1375656 | 1375656 | 5454.88 | 1 | 30960 |
| 20MU8035NPS | 1/2/2021 | 876407 | 876490 | 1750925 | 1733587 | 1733587 | 7821.53 | 2 | 21986 |
| 20MU8036NPS | 1/2/2021 | 1041452 | 1041589 | 2081013 | 2068233 | 2068233 | 8775.27 | 3 | 26842 |
| 20MU8037NPS | 1/2/2021 | 987508 | 987574 | 1973441 | 1961952 | 1961952 | 8030.15 | 1 | 23400 |
| 20MU8038NPS | 1/2/2021 | 930847 | 930930 | 1859955 | 1853749 | 1853749 | 8248.92 | 2 | 20268 |
| 20MU8043NPS | 1/4/2021 | 940785 | 940856 | 1878907 | 1871866 | 1871866 | 8306.06 | 3 | 20022 |
| 20MU8044NPS | 1/4/2021 | 1093537 | 1093711 | 2185340 | 2176459 | 2176459 | 9210.06 | 5 | 21909 |
| 20MU8046NPS | 1/4/2021 | 893145 | 893170 | 1784719 | 1735101 | 1735101 | 7667.73 | 5 | 18387 |
| 20MU8047NPS | 1/4/2021 | 841802 | 841867 | 1682386 | 1676913 | 1676913 | 7346.87 | 3 | 18331 |
| 20MU8050NPS | 1/4/2021 | 1300656 | 1300814 | 2598843 | 2515954 | 2515954 | 10702.93 | 5 | 29577 |
| 20MU8055NPS | 1/5/2021 | 1035008 | 1035139 | 2068319 | 2061111 | 2061111 | 8778.39 | 1 | 23444 |
| 20MU8057NPS | 1/5/2021 | 858892 | 858944 | 1716506 | 1706931 | 1706931 | 7583.24 | 1 | 19986 |
| 20MU8058NPS | 1/5/2021 | 97218 | 97264 | 194335 | 193380 | 193380 | 552.74 | 1 | 1465 |
| 20MU8059NPS | 1/5/2021 | 843931 | 843967 | 1686098 | 1671049 | 1671049 | 7462.54 | 5 | 19330 |
| 20MU8061NPS | 1/5/2021 | 1034170 | 1034274 | 2066703 | 2060542 | 2060542 | 8554.34 | 4 | 21303 |
| 20MU8063NPS | 1/5/2021 | 1065918 | 1066040 | 2129115 | 2121214 | 2121214 | 9435.78 | 3 | 25470 |
| 20MU8065NPS | 1/5/2021 | 767864 | 767921 | 1534186 | 1525846 | 1525846 | 6826.87 | 1 | 18690 |
| 20MU8068NPS | 1/5/2021 | 900509 | 900661 | 1797297 | 1789393 | 1789393 | 7943.58 | 5 | 19578 |
| 20MU8071NPS | 1/5/2021 | 648001 | 648061 | 1294176 | 1275776 | 1275776 | 5662.99 | 2 | 15485 |
| 20MU8072NPS | 1/5/2021 | 1316996 | 1317149 | 2631783 | 2573486 | 2573486 | 10911.48 | 1 | 37480 |
| 20MU8074NPS | 1/5/2021 | 809333 | 809517 | 1616920 | 1610470 | 1610470 | 6826.43 | 1 | 17970 |
| 20MU8078NPS | 1/6/2021 | 1008448 | 1008545 | 2014469 | 2006635 | 2006635 | 8856.75 | 1 | 23295 |
| 20MU8081NPS | 1/6/2021 | 1040796 | 1040851 | 2079371 | 2063786 | 2063786 | 9167.89 | 1 | 24325 |
| 20MU8083NPS | 1/6/2021 | 690273 | 690338 | 1378856 | 1370411 | 1370411 | 6119.46 | 5 | 15388 |
| 20MU8088NPS | 1/6/2021 | 639032 | 639131 | 1276675 | 1270061 | 1270061 | 5515.44 | 1 | 15889 |
| 20MU8093NPS | 1/7/2021 | 1022110 | 1022220 | 2042735 | 2029127 | 2029127 | 8555.05 | 2 | 23229 |
| 20MU8094NPS | 1/7/2021 | 1148290 | 1148444 | 2294764 | 2284089 | 2284089 | 9663.30 | 1 | 27389 |
| 20MU8097NPS | 1/7/2021 | 785618 | 785640 | 1569769 | 1556560 | 1556560 | 6828.68 | 1 | 17585 |
| 20MU8098NPS | 1/7/2021 | 989208 | 989283 | 1976954 | 1969049 | 1969049 | 8345.25 | 5 | 21209 |
| 20MU8105NPS | 1/8/2021 | 730947 | 730982 | 1460628 | 1449958 | 1449958 | 6270.08 | 1 | 17986 |
| 20MU8106NPS | 1/8/2021 | 966719 | 966804 | 1931420 | 1902973 | 1902973 | 8404.64 | 1 | 22203 |
| 20MU8107NPS | 1/8/2021 | 1176698 | 1176884 | 2351449 | 2341757 | 2341757 | 9870.44 | 3 | 26385 |
| 20MU8116NPS | 1/8/2021 | 510002 | 510021 | 1018678 | 1004263 | 1004263 | 4480.77 | 1 | 11878 |
| 20MU8117NPS | 1/8/2021 | 1019831 | 1019917 | 2038208 | 2031126 | 2031126 | 8628.27 | 1 | 24334 |
| 20MU8120NPS | 1/11/2021 | 1216288 | 1216426 | 2430476 | 2422151 | 2422151 | 10280.28 | 1 | 27802 |
| 20MU8123NPS | 1/11/2021 | 824422 | 824462 | 1647062 | 1634119 | 1634119 | 7389.12 | 1 | 21318 |
| 20MU8124NPS | 1/11/2021 | 3547708 | 3556164 | 7093726 | 3027038 | 3027038 | 20228.42 | 1 | 453350 |
| 20MU8125NPS | 1/11/2021 | 1283762 | 1283890 | 2565525 | 2543648 | 2543648 | 10865.49 | 1 | 29790 |
| 20MU8127NPS | 1/11/2021 | 890186 | 890294 | 1778246 | 1771685 | 1771685 | 7788.18 | 3 | 18006 |
| 20MU8136NPS | 1/12/2021 | 563933 | 564039 | 1126815 | 1120247 | 1120247 | 4797.74 | 1 | 13567 |
| 20MU8140NPS | 1/13/2021 | 969012 | 969118 | 1936232 | 1912022 | 1912022 | 8386.39 | 4 | 21939 |
| 20MU8141NPS | 1/13/2021 | 1447036 | 1448233 | 2888587 | 718265 | 718265 | 57185.29 | 1 | 116473 |
| 20MU8142NPS | 1/13/2021 | 1009921 | 1009941 | 2017552 | 2011081 | 2011081 | 8993.18 | 3 | 23564 |
| 20MU8148NPS | 1/13/2021 | 990357 | 990411 | 1978903 | 1967512 | 1967512 | 8800.55 | 1 | 21720 |
| 20MU8154NPS | 1/14/2021 | 711443 | 711522 | 1421473 | 1415557 | 1415557 | 6180.17 | 1 | 15480 |
| 20MU8157NPS | 1/14/2021 | 765882 | 765996 | 1530113 | 1521976 | 1521976 | 6644.75 | 2 | 14964 |
| 20MU8158NPS | 1/14/2021 | 957236 | 957325 | 1912634 | 1889566 | 1889566 | 8389.15 | 2 | 22384 |
| 20MU8161NPS | 1/14/2021 | 859216 | 859315 | 1716894 | 1684969 | 1684969 | 7223.58 | 1 | 20039 |
| 20MU8162NPS | 1/14/2021 | 2109765 | 2113090 | 4198463 | 1211086 | 1211086 | 52543.79 | 1 | 178213 |
| 20MU8165NPS | 1/14/2021 | 911138 | 911527 | 1820459 | 1812315 | 1812315 | 7507.42 | 3 | 19781 |
| 20MU8166NPS | 1/13/2021 | 1057167 | 1057219 | 2113070 | 2104999 | 2104999 | 8406.66 | 2 | 23675 |
| 20MU8168NPS | 1/15/2021 | 883260 | 883311 | 1764406 | 1747786 | 1747786 | 7830.94 | 2 | 19644 |
| 20MU8170NPS | 1/15/2021 | 1360418 | 1360643 | 2718827 | 2688353 | 2688353 | 11482.74 | 2 | 28238 |
| 20MU8173NPS | 1/15/2021 | 854742 | 854762 | 1707827 | 1702270 | 1702270 | 7550.45 | 2 | 18335 |
| 20MU8174NPS | 1/15/2021 | 771801 | 771831 | 1541864 | 1496138 | 1496138 | 6792.81 | 4 | 20193 |
| 20MU8176NPS | 1/15/2021 | 798667 | 798707 | 1595498 | 1578004 | 1578004 | 6979.52 | 1 | 18313 |
| 20MU8180NPS | 1/15/2021 | 813966 | 813987 | 1626378 | 1614527 | 1614527 | 7219.48 | 1 | 20380 |
| 20MU8184NPS | 1/15/2021 | 866392 | 866462 | 1730658 | 1719757 | 1719757 | 7737.95 | 1 | 21189 |
| 20MU8191NPS | 1/17/2021 | 1082921 | 1083031 | 2163523 | 2153574 | 2153574 | 9068.46 | 13 | 22343 |
| 20MU8193NPS | 1/18/2021 | 973747 | 973856 | 1945701 | 1939098 | 1939098 | 8479.55 | 1 | 21530 |
| 20MU8194NPS | 1/18/2021 | 1651269 | 1651408 | 3300199 | 3212492 | 3212492 | 13642.87 | 2 | 36919 |
| 20MU8201NPS | 1/18/2021 | 886238 | 886342 | 1769621 | 1760420 | 1760420 | 7840.13 | 1 | 21490 |
| 20MU8211NPS | 1/19/2021 | 804705 | 804802 | 1607468 | 1601624 | 1601624 | 7052.38 | 1 | 16763 |
| 20MU8212NPS | 1/19/2021 | 888876 | 888982 | 1776304 | 1760212 | 1760212 | 7593.54 | 1 | 17885 |
| 20MU8216NPS | 1/19/2021 | 1762308 | 1765284 | 3522524 | 1422769 | 1422769 | 35401.36 | 1 | 213567 |
| 20MU8221NPS | 1/19/2021 | 636149 | 636211 | 1269552 | 1256602 | 1256602 | 5672.29 | 1 | 15726 |
| 20MU8223NPS | 1/20/2021 | 901987 | 902140 | 1802670 | 1795605 | 1795605 | 7546.08 | 1 | 21356 |
| 20MU8224NPS | 1/20/2021 | 982943 | 983028 | 1963611 | 1912621 | 1912621 | 8502.29 | 4 | 24343 |
| 20MU8226NPS | 1/20/2021 | 1195638 | 1195776 | 2389154 | 2373606 | 2373606 | 10000.93 | 1 | 29117 |
| 20MU8229NPS | 1/20/2021 | 858436 | 858565 | 1715521 | 1704819 | 1704819 | 7423.68 | 1 | 17779 |
| 20MU8230NPS | 1/20/2021 | 892167 | 892226 | 1781907 | 1659829 | 1659829 | 7399.43 | 1 | 20820 |
| 20MU8246NPS | 1/22/2021 | 1582151 | 1582410 | 3161411 | 3148308 | 3148308 | 13322.78 | 5 | 32036 |
| 20MU8247NPS | 1/22/2021 | 1361847 | 1362011 | 2721893 | 2714876 | 2714876 | 11461.17 | 1 | 31965 |
| 20MU8249NPS | 1/22/2021 | 1036374 | 1036427 | 2070716 | 2012869 | 2012869 | 8759.48 | 1 | 25867 |
| 20MU8252NPS | 1/22/2021 | 1227955 | 1228099 | 2454246 | 2447008 | 2447008 | 10409.52 | 3 | 28201 |
| 20MU8253NPS | 1/22/2021 | 696903 | 696967 | 1392444 | 1372556 | 1372556 | 5951.47 | 4 | 13914 |
| 20MU8257NPS | 1/23/2021 | 874854 | 874907 | 1746963 | 1740689 | 1740689 | 7931.81 | 5 | 21034 |
| 20MU8258NPS | 1/23/2021 | 1060463 | 1060672 | 2119440 | 2103681 | 2103681 | 8894.89 | 1 | 24727 |
| 20MU8259NPS | 1/23/2021 | 873741 | 873816 | 1745976 | 1738023 | 1738023 | 7442.43 | 3 | 17636 |
| 20MU8261NPS | 1/23/2021 | 1099059 | 1099192 | 2196091 | 2120001 | 2120001 | 9014.27 | 1 | 24684 |
| 20MU8263NPS | 1/23/2021 | 884917 | 885222 | 1768158 | 1755360 | 1755360 | 7503.74 | 1 | 20448 |
| 20MU8264NPS | 1/23/2021 | 1096528 | 1096772 | 2191035 | 2174876 | 2174876 | 9227.70 | 1 | 28123 |
| 20MU8267NPS | 1/23/2021 | 886062 | 886186 | 1769775 | 1762361 | 1762361 | 7858.20 | 3 | 18276 |
| 20MU8269NPS | 1/25/2021 | 902854 | 902896 | 1802957 | 1778432 | 1778432 | 7890.69 | 1 | 19312 |
| 20MU8270NPS | 1/25/2021 | 849353 | 849358 | 1697055 | 1686354 | 1686354 | 7749.40 | 3 | 21552 |
| 20MU8271NPS | 1/24/2021 | 1025276 | 1025362 | 2049324 | 2039960 | 2039960 | 8162.91 | 1 | 22737 |
| 20MU8273NPS | 1/25/2021 | 928830 | 928909 | 1855992 | 1816608 | 1816608 | 7979.21 | 1 | 21245 |
| 20MU8275NPS | 1/25/2021 | 865313 | 865422 | 1728699 | 1698046 | 1698046 | 7473.94 | 2 | 20956 |
| 20MU8285NPS | 1/26/2021 | 1136442 | 1136602 | 2270785 | 2260442 | 2260442 | 9582.48 | 1 | 23038 |
| 20MU8286NPS | 1/25/2021 | 837823 | 837929 | 1673782 | 1663973 | 1663973 | 7245.42 | 7 | 17441 |
| 20MU8287NPS | 1/26/2021 | 934962 | 935010 | 1868200 | 1835876 | 1835876 | 8126.79 | 1 | 22303 |
| 20MU8288NPS | 1/26/2021 | 813380 | 813424 | 1624553 | 1611173 | 1611173 | 7355.67 | 11 | 19767 |
| 20MU8294NPS | 1/26/2021 | 8083579 | 8102507 | 16166179 | 15923656 | 15923656 | 48389.04 | 1 | 164541 |
| 20MU8295NPS | 1/26/2021 | 946691 | 946778 | 1891819 | 1869721 | 1869721 | 7976.28 | 1 | 22824 |
| 20MU8298NPS | 1/26/2021 | 834044 | 834077 | 1665739 | 1616675 | 1616675 | 7285.57 | 1 | 20309 |
| 20MU8302NPS | 1/26/2021 | 1190395 | 1190533 | 2378568 | 2366448 | 2366448 | 10035.54 | 1 | 27863 |
| 20MU8308NPS | 1/26/2021 | 825073 | 825084 | 1648895 | 1644706 | 1644706 | 7214.71 | 1 | 19704 |
| 20MU8310NPS | 1/27/2021 | 979472 | 979515 | 1956847 | 1943914 | 1943914 | 8564.97 | 4 | 21018 |
| 20MU8311NPS | 1/27/2021 | 922317 | 922449 | 1843084 | 1817599 | 1817599 | 8033.05 | 1 | 21293 |
| 20MU8329NPS | 1/28/2021 | 1037586 | 1037683 | 2073801 | 2065780 | 2065780 | 8711.11 | 1 | 24399 |
| 20MU8330NPS | 1/28/2021 | 788356 | 788594 | 1575215 | 1568490 | 1568490 | 6571.08 | 1 | 18275 |
| 20MU8338NPS | 1/28/2021 | 800034 | 800104 | 1597360 | 1589887 | 1589887 | 6943.08 | 3 | 19307 |
| 20MU8342NPS | 1/29/2021 | 1015462 | 1015578 | 2028725 | 2021057 | 2021057 | 8831.51 | 2 | 23913 |
| 20MU8344NPS | 1/29/2021 | 856954 | 857070 | 1713220 | 1704891 | 1704891 | 5650.62 | 1 | 14734 |
| 20MU8345NPS | 1/29/2021 | 862917 | 863006 | 1724304 | 1703187 | 1703187 | 7400.65 | 9 | 18490 |
| 20MU8348NPS | 1/29/2021 | 1637877 | 1640040 | 3272929 | 1149217 | 1149217 | 27962.85 | 1 | 186600 |
| 20MU8349NPS | 1/29/2021 | 1688886 | 1701528 | 3332892 | 528873 | 528873 | 36963.27 | 1 | 78691 |
| 20MU8359NPS | 1/29/2021 | 879500 | 879537 | 1757266 | 1745504 | 1745504 | 7782.72 | 1 | 22030 |
| 20MU8361NPS | 1/30/2021 | 1059974 | 1060183 | 2118589 | 2109581 | 2109581 | 8834.20 | 2 | 24490 |
| 20MU8363NPS | 1/30/2021 | 865790 | 865902 | 1729691 | 1719659 | 1719659 | 7614.33 | 2 | 18999 |
| 20MU8364NPS | 1/30/2021 | 1059797 | 1059894 | 2117617 | 2101694 | 2101694 | 9118.11 | 1 | 21896 |
| 20MU8365NPS | 1/30/2021 | 1771727 | 1771953 | 3539815 | 3506425 | 3506425 | 15455.72 | 1 | 42529 |
| 20MU8366NPS | 1/30/2021 | 1480713 | 1482941 | 2957138 | 807635 | 807635 | 43752.06 | 1 | 136004 |
| 20MU8367NPS | 1/30/2021 | 820065 | 820122 | 1637390 | 1508631 | 1508631 | 6739.00 | 1 | 18839 |
| 20MU8370NPS | 1/30/2021 | 980924 | 981105 | 1959656 | 1944687 | 1944687 | 8595.77 | 1 | 24444 |
| 20MU8536NPS | 3/22/2021 | 684252 | 684252 | 1367832 | 1357674 | 1357674 | 5330.28 | 1 | 30475 |
| 20MU8542NPS | 3/26/2021 | 463824 | 463824 | 927209 | 916847 | 916847 | 3591.82 | 9 | 21263 |
| 20MU8543NPS | 3/26/2021 | 274176 | 274176 | 548098 | 547358 | 547358 | 2133.28 | 20 | 12044 |
| 20MU8549NPS | 3/31/2021 | 436065 | 436065 | 871736 | 870450 | 870450 | 3059.27 | 1 | 12301 |
| 20MU8552NPS | 3/31/2021 | 332016 | 332016 | 663551 | 662622 | 662622 | 2491.24 | 7 | 8526 |
| 20MU8701NPS | 4/2/2021 | 667963 | 667963 | 1335179 | 1333230 | 1333230 | 4927.44 | 1 | 19119 |
| 20MU8702NPS | 4/2/2021 | 597531 | 597531 | 1194609 | 1193378 | 1193378 | 4655.73 | 1 | 21410 |
| 20MU8703NPS | 4/4/2021 | 513485 | 513485 | 1026472 | 1025440 | 1025440 | 4001.15 | 6 | 21190 |
| 20MU8708NPS | 4/5/2021 | 333063 | 333063 | 665806 | 654245 | 654245 | 2534.04 | 15 | 13337 |
| 20MU8710NPS | 4/6/2021 | 391764 | 391764 | 783162 | 782284 | 782284 | 3069.43 | 1 | 15500 |
| 20MU8711NPS | 4/6/2021 | 658224 | 658224 | 1315809 | 1313820 | 1313820 | 4877.46 | 2 | 17952 |
| 20MU8712NPS | 4/6/2021 | 454369 | 454369 | 908181 | 906972 | 906972 | 3492.35 | 1 | 13595 |
| 20MU8717NPS | 4/7/2021 | 351549 | 351549 | 702329 | 701292 | 701292 | 2789.57 | 1 | 16759 |
| 20MU8719NPS | 4/9/2021 | 433291 | 433291 | 866079 | 865019 | 865019 | 3360.89 | 1 | 14830 |
| 20MU8721NPS | 4/13/2021 | 2234149 | 2234149 | 4466380 | 4460377 | 4460377 | 17512.44 | 1 | 96527 |
| 20MU8722NPS | 4/13/2021 | 412148 | 412148 | 823856 | 822868 | 822868 | 3248.37 | 5 | 19256 |
| 20MU8724NPS | 4/14/2021 | 292568 | 292568 | 584757 | 583853 | 583853 | 2091.16 | 1 | 8143 |
| 20MU8728NPS | 4/15/2021 | 339895 | 339895 | 679430 | 665568 | 665568 | 2659.45 | 1 | 13669 |
| 20MU8730NPS | 4/15/2021 | 646596 | 646596 | 1292406 | 1290344 | 1290344 | 4883.61 | 1 | 19127 |
| 20MU8734NPS | 4/18/2021 | 255457 | 255457 | 510607 | 509939 | 509939 | 1977.25 | 1 | 7582 |
| 20MU8735NPS | 4/18/2021 | 503747 | 503747 | 1006944 | 1005955 | 1005955 | 3990.86 | 1 | 17455 |
| 20MU8743NPS | 4/21/2021 | 843318 | 843318 | 1685677 | 1683352 | 1683352 | 6417.80 | 1 | 22920 |
| 20MU8745NPS | 4/21/2021 | 379248 | 379248 | 758088 | 739211 | 739211 | 2919.65 | 1 | 17454 |
| 20MU8746NPS | 4/22/2021 | 344418 | 344418 | 688592 | 687663 | 687663 | 2640.19 | 1 | 10921 |
| 20MU8751NPS | 4/27/2021 | 416054 | 416054 | 831529 | 830520 | 830520 | 3282.57 | 32 | 10577 |
| 20MU8752NPS | 4/28/2021 | 519300 | 519300 | 1038142 | 1036492 | 1036492 | 3837.10 | 2 | 16173 |
| 20MU8753NPS | 4/28/2021 | 334359 | 334359 | 668021 | 667069 | 667069 | 2643.40 | 1 | 8676 |
| 20MU8756NPS | 4/25/2021 | 15406 | 15406 | 30776 | 30714 | 30714 | 121.99 | 1 | 356 |
| 20MU8757NPS | 4/29/2021 | 349037 | 349037 | 697620 | 696622 | 696622 | 2712.59 | 1 | 10398 |
| 20MU8758NPS | 4/29/2021 | 363297 | 363297 | 725683 | 724512 | 724512 | 2873.20 | 1 | 10133 |
| 20MU8761NPS | 4/30/2021 | 202526 | 202526 | 404664 | 346645 | 346645 | 1369.65 | 20 | 4263 |
| 20MU8764NPS | 4/30/2021 | 482341 | 482341 | 964259 | 948984 | 948984 | 3708.44 | 2 | 22753 |
| 20MU8768NPS | 5/11/2021 | 47462 | 47462 | 94760 | 94595 | 94595 | 376.28 | 3 | 1711 |
| 20MU8769NPS | 5/11/2021 | 523657 | 523657 | 1046633 | 1045200 | 1045200 | 4128.27 | 1 | 14165 |
| 20MU8770NPS | 5/12/2021 | 460314 | 460314 | 920212 | 919041 | 919041 | 3627.11 | 1 | 27448 |
| 20MU8775NPS | 5/14/2021 | 416575 | 416575 | 832396 | 667491 | 667491 | 2692.63 | 1 | 19691 |
| 20MU8776NPS | 5/14/2021 | 444855 | 444855 | 888315 | 159006 | 159006 | 628.18 | 1 | 3746 |
| 20MU8777NPS | 5/18/2021 | 333137 | 333137 | 665259 | 256594 | 256594 | 1017.00 | 7 | 6078 |
| 20MU8778NPS | 5/20/2021 | 396867 | 396867 | 793154 | 783760 | 783760 | 3135.72 | 1 | 20363 |
| 20MU8779NPS | 5/20/2021 | 464045 | 464045 | 927534 | 926367 | 926367 | 3662.66 | 5 | 14901 |
| 20MU8780NPS | 5/21/2021 | 1131253 | 1131253 | 2261443 | 2259028 | 2259028 | 8873.76 | 1 | 58016 |
| 20MU8782NPS | 5/23/2021 | 412294 | 412294 | 824153 | 823099 | 823099 | 3179.50 | 2 | 12060 |
| 20MU8786NPS | 5/27/2021 | 383436 | 383436 | 766342 | 694984 | 694984 | 2794.17 | 1 | 23843 |
| 20MU8787NPS | 5/28/2021 | 719447 | 719447 | 1438059 | 1436267 | 1436267 | 5682.39 | 1 | 18720 |
| 20MU8789NPS | 6/1/2021 | 590801 | 590801 | 1180964 | 1179405 | 1179405 | 4432.84 | 1 | 16714 |
| 20MU8790NPS | 6/1/2021 | 435394 | 435394 | 870072 | 866480 | 866480 | 3496.49 | 3 | 15667 |
| 20MU8791NPS | 6/1/2021 | 540158 | 540158 | 1079138 | 641159 | 641159 | 2566.09 | 1 | 24531 |
| 20MU8792NPS | 6/1/2021 | 426258 | 426258 | 851973 | 850879 | 850879 | 3312.08 | 2 | 10916 |
| 20MU8793NPS | 6/1/2021 | 607917 | 607917 | 1215147 | 1145428 | 1145428 | 4607.24 | 1 | 38204 |
| 20MU8794NPS | 6/2/2021 | 515229 | 515229 | 1029870 | 1022689 | 1022689 | 4127.75 | 4 | 27256 |
| 20MU8797NPS | 6/3/2021 | 472203 | 472203 | 943806 | 833088 | 833088 | 3347.14 | 1 | 21826 |
| 20MU8800NPS | 6/3/2021 | 551807 | 551807 | 1103029 | 1101623 | 1101623 | 4199.71 | 1 | 16628 |
| 20MU8801NPS | 6/4/2021 | 265083 | 265083 | 529847 | 529119 | 529119 | 2057.61 | 2 | 7985 |
| 20MU8805NPS | 6/7/2021 | 625574 | 625574 | 1250468 | 1248684 | 1248684 | 4588.09 | 1 | 16423 |
| 20MU8809NPS | 6/8/2021 | 422028 | 422028 | 843581 | 842187 | 842187 | 3239.30 | 1 | 10780 |
| 20MU8810NPS | 6/8/2021 | 340062 | 340062 | 679765 | 678922 | 678922 | 2681.32 | 1 | 9321 |
| 20MU8811NPS | 6/9/2021 | 1398993 | 1398993 | 2796040 | 2791484 | 2791484 | 10690.42 | 1 | 40997 |
| 20MU8812NPS | 6/9/2021 | 438868 | 438868 | 877236 | 876171 | 876171 | 3423.78 | 1 | 13649 |
| 20MU8813NPS | 6/9/2021 | 413726 | 413726 | 826896 | 816105 | 816105 | 3276.53 | 1 | 16526 |
| 20MU8816NPS | 6/10/2021 | 500830 | 500830 | 1000684 | 618615 | 618615 | 2486.27 | 1 | 15860 |
| 20MU8818NPS | 6/10/2021 | 503865 | 503865 | 1007063 | 1005767 | 1005767 | 4018.73 | 1 | 14093 |
| 20MU8820NPS | 6/10/2021 | 477609 | 477609 | 954577 | 942794 | 942794 | 3786.38 | 2 | 28647 |
| 20MU8822NPS | 6/7/2021 | 394383 | 394383 | 788337 | 778852 | 778852 | 3116.94 | 2 | 18983 |
| 20MU8823NPS | 6/12/2021 | 403326 | 403326 | 806182 | 805241 | 805241 | 3137.55 | 1 | 11678 |
| 20MU8824NPS | 6/12/2021 | 342059 | 342059 | 682292 | 103745 | 103745 | 410.56 | 1 | 3019 |
| 20MU8826NPS | 6/13/2021 | 428879 | 428879 | 857143 | 853736 | 853736 | 3462.16 | 1 | 15039 |
| 20MU8829NPS | 6/14/2021 | 377451 | 377451 | 754517 | 753503 | 753503 | 2941.34 | 20 | 10072 |
| 20MU8831NPS | 6/14/2021 | 366718 | 366718 | 733000 | 726518 | 726518 | 2926.80 | 1 | 13768 |
| 20MU8832NPS | 6/14/2021 | 439584 | 439584 | 878686 | 877503 | 877503 | 3053.93 | 1 | 12300 |
| 20MU8833NPS | 6/15/2021 | 421246 | 421246 | 842016 | 840849 | 840849 | 3120.72 | 1 | 11955 |
| 20MU8834NPS | 6/15/2021 | 351161 | 351161 | 701922 | 700980 | 700980 | 2644.42 | 1 | 10336 |
| 20MU8835NPS | 6/15/2021 | 558033 | 558033 | 1115412 | 1113878 | 1113878 | 4276.02 | 1 | 14736 |
| 20MU8837NPS | 6/16/2021 | 426484 | 426484 | 852512 | 846962 | 846962 | 3417.19 | 2 | 18060 |
| 20MU8838NPS | 6/16/2021 | 399723 | 399723 | 798954 | 787314 | 787314 | 3173.58 | 1 | 17039 |
| 20MU8839NPS | 6/16/2021 | 493664 | 493664 | 986816 | 983067 | 983067 | 3963.16 | 1 | 17554 |
| 20MU8842NPS | 6/18/2021 | 437970 | 437970 | 874947 | 515783 | 515783 | 2079.36 | 2 | 15055 |
| 20MU8843NPS | 6/18/2021 | 616063 | 616063 | 1231457 | 1229894 | 1229894 | 4819.55 | 23 | 16607 |
| 20MU8845NPS | 6/19/2021 | 552964 | 552964 | 1105254 | 1090834 | 1090834 | 4401.94 | 22 | 21790 |
| 20MU8846NPS | 6/19/2021 | 346720 | 346720 | 693088 | 692196 | 692196 | 2606.26 | 1 | 7003 |
| 20MU8847NPS | 6/19/2021 | 446295 | 446295 | 892019 | 890769 | 890769 | 3300.43 | 1 | 12167 |
| 20MU8848NPS | 6/19/2021 | 474334 | 474334 | 947087 | 495459 | 495459 | 1986.14 | 1 | 11822 |
| 20MU8850NPS | 6/20/2021 | 546718 | 546718 | 1092790 | 1091175 | 1091175 | 3914.27 | 1 | 16564 |
| 20MU8851NPS | 6/20/2021 | 364170 | 364170 | 727876 | 654651 | 654651 | 2629.94 | 1 | 20566 |
| 20MU8852NPS | 6/20/2021 | 770541 | 770541 | 1540348 | 1524017 | 1524017 | 6147.02 | 1 | 37201 |
| 20MU8853NPS | 6/20/2021 | 426922 | 426922 | 853430 | 844375 | 844375 | 3401.68 | 1 | 20581 |
| 20MU8854NPS | 6/21/2021 | 356617 | 356617 | 712846 | 711999 | 711999 | 2768.32 | 1 | 11615 |
| 20MU8856NPS | 6/21/2021 | 382158 | 382158 | 763828 | 762779 | 762779 | 2902.86 | 1 | 10372 |
| 20MU8857NPS | 6/21/2021 | 463453 | 463453 | 926251 | 920419 | 920419 | 3700.12 | 1 | 24119 |
| 20MU8858NPS | 6/21/2021 | 472043 | 472043 | 943631 | 942660 | 942660 | 3709.37 | 1 | 15622 |
| 20MU8859NPS | 6/21/2021 | 350551 | 350551 | 700628 | 699754 | 699754 | 2656.49 | 1 | 8775 |
| 20MU8860NPS | 6/21/2021 | 385368 | 385368 | 769312 | 281856 | 281856 | 1131.66 | 1 | 7933 |
| 20MU8861NPS | 6/21/2021 | 634158 | 634158 | 1267650 | 1265636 | 1265636 | 4678.04 | 1 | 16986 |
| 20MU8862NPS | 6/21/2021 | 426902 | 426902 | 852501 | 257691 | 257691 | 1034.23 | 1 | 7577 |
| 20MU8864NPS | 6/21/2021 | 555689 | 555689 | 1110669 | 1109296 | 1109296 | 4299.92 | 1 | 15551 |
| 20MU8865NPS | 6/21/2021 | 707133 | 707133 | 1413389 | 1411517 | 1411517 | 5344.68 | 3 | 19413 |
| 20MU8866NPS | 6/21/2021 | 524251 | 524251 | 1047850 | 1046551 | 1046551 | 3764.00 | 1 | 14734 |
| 20MU8867NPS | 6/22/2021 | 386396 | 386396 | 772305 | 755187 | 755187 | 3043.43 | 2 | 15658 |
| 20MU8869NPS | 6/22/2021 | 525836 | 525836 | 1050805 | 1048512 | 1048512 | 4274.55 | 1 | 12589 |
| 20MU8871NPS | 6/22/2021 | 558812 | 558812 | 1116924 | 1095491 | 1095491 | 4318.68 | 2 | 15354 |
| 20MU8872NPS | 6/22/2021 | 378131 | 378131 | 755656 | 717854 | 717854 | 2834.84 | 3 | 10496 |
| 20MU8874NPS | 6/22/2021 | 287354 | 287354 | 574230 | 573501 | 573501 | 2294.06 | 14 | 8379 |
| 20MU8875NPS | 6/22/2021 | 237583 | 237583 | 474802 | 474070 | 474070 | 1926.28 | 1 | 8104 |
| 20MU8876NPS | 6/23/2021 | 217018 | 217018 | 433735 | 432341 | 432341 | 1753.36 | 2 | 7621 |
| 20MU8879NPS | 6/23/2021 | 589265 | 589265 | 1177965 | 1174916 | 1174916 | 4626.91 | 1 | 24414 |
| 20MU8880NPS | 6/23/2021 | 394179 | 394179 | 787564 | 784182 | 784182 | 3181.45 | 13 | 16807 |
| 20MU8881NPS | 6/23/2021 | 232886 | 232886 | 465359 | 464583 | 464583 | 1892.85 | 1 | 6806 |
| 20MU8882NPS | 6/23/2021 | 568700 | 568700 | 1136500 | 1127835 | 1127835 | 4515.78 | 2 | 21763 |
| 20MU8883NPS | 6/23/2021 | 335649 | 335649 | 670918 | 668406 | 668406 | 2690.91 | 9 | 12938 |
| 20MU8884NPS | 6/23/2021 | 632031 | 632031 | 1263235 | 1259908 | 1259908 | 5091.40 | 1 | 24620 |
| 20MU8885NPS | 6/23/2021 | 659049 | 659049 | 1317461 | 1316032 | 1316032 | 5207.61 | 2 | 20529 |
| 20MU8886NPS | 6/23/2021 | 298421 | 298421 | 596385 | 595048 | 595048 | 2420.57 | 1 | 8781 |
| 20MU8887NPS | 6/23/2021 | 261769 | 261769 | 523063 | 522106 | 522106 | 2121.45 | 31 | 7770 |
| 20MU8890NPS | 6/24/2021 | 251956 | 251956 | 503565 | 502815 | 502815 | 2032.66 | 1 | 7390 |
| 20MU8892NPS | 6/24/2021 | 534533 | 534533 | 1068145 | 1066274 | 1066274 | 4344.19 | 1 | 15141 |
| 20MU8893NPS | 6/24/2021 | 616173 | 616173 | 1231597 | 1230183 | 1230183 | 4860.27 | 1 | 16790 |
| 20MU8959NPS | 7/1/2021 | 243781 | 243781 | 487149 | 486438 | 486438 | 1999.92 | 1 | 6031 |
| 20MU8960NPS | 7/1/2021 | 215742 | 215742 | 431067 | 430320 | 430320 | 1748.29 | 1 | 5917 |
| 20MU8961NPS | 7/21/2021 | 437438 | 437438 | 874148 | 872151 | 872151 | 3521.75 | 3 | 13618 |
| 20MU8962NPS | 7/1/2021 | 601988 | 601988 | 1203329 | 1200922 | 1200922 | 4732.26 | 1 | 18284 |
| 20MU8963NPS | 7/1/2021 | 523595 | 523595 | 1046664 | 1045594 | 1045594 | 4127.72 | 1 | 14511 |
| 20MU8965NPS | 7/1/2021 | 235697 | 235697 | 471045 | 470416 | 470416 | 1901.64 | 1 | 5461 |
| 20MU8966NPS | 7/1/2021 | 407586 | 407586 | 814465 | 812621 | 812621 | 3314.72 | 1 | 11272 |
| 20MU8967NPS | 7/1/2021 | 441175 | 441175 | 881819 | 879978 | 879978 | 3496.92 | 1 | 19014 |
| 20MU8968NPS | 7/1/2021 | 199607 | 199607 | 398913 | 398240 | 398240 | 1573.66 | 1 | 6684 |
| 20MU8969NPS | 7/1/2021 | 202659 | 202659 | 405114 | 385799 | 385799 | 1519.17 | 1 | 6598 |
| 20MU8970NPS | 7/1/2021 | 195051 | 195051 | 389853 | 386929 | 386929 | 1523.41 | 1 | 4961 |
| 20MU8971NPS | 7/1/2021 | 234259 | 234259 | 468302 | 467684 | 467684 | 1846.84 | 1 | 6167 |
| 20MU8972NPS | 7/1/2021 | 293314 | 293314 | 586263 | 585508 | 585508 | 2345.67 | 10 | 7491 |
| 20MU8973NPS | 7/1/2021 | 211026 | 211026 | 421729 | 421121 | 421121 | 1681.23 | 8 | 5113 |
| 20MU8974NPS | 7/1/2021 | 321034 | 321034 | 641700 | 636936 | 636936 | 2509.33 | 14 | 10514 |
| 20MU8976NPS | 7/1/2021 | 311865 | 311865 | 623493 | 616568 | 616568 | 2422.36 | 1 | 9485 |
| 20MU8977NPS | 7/1/2021 | 179111 | 179111 | 357981 | 357090 | 357090 | 1409.01 | 1 | 5855 |
| 20MU8978NPS | 7/2/2021 | 234254 | 234254 | 468150 | 467385 | 467385 | 1852.05 | 1 | 6023 |
| 20MU8987NPS | 7/3/2021 | 761055 | 761055 | 1521514 | 1518900 | 1518900 | 5994.27 | 1 | 25574 |
| 20MU8988NPS | 7/3/2021 | 125421 | 125421 | 250672 | 244075 | 244075 | 1006.53 | 18 | 3948 |
| 20MU8989NPS | 7/3/2021 | 388043 | 388043 | 775784 | 754807 | 754807 | 2982.36 | 31 | 12213 |
| 20MU8990NPS | 7/4/2021 | 732411 | 732411 | 1464094 | 1462286 | 1462286 | 5830.39 | 2 | 25837 |
| 20MU8991NPS | 7/4/2021 | 219877 | 219877 | 439447 | 438946 | 438946 | 1738.66 | 1 | 6157 |
| 20MU8994NPS | 7/5/2021 | 589616 | 589616 | 1178689 | 1176344 | 1176344 | 4666.05 | 1 | 25159 |
| 20MU8997NPS | 7/6/2021 | 783662 | 783662 | 1566651 | 1564304 | 1564304 | 6212.40 | 1 | 29906 |
| 20MU8998NPS | 7/6/2021 | 1406000 | 1406000 | 2810647 | 2799357 | 2799357 | 11108.04 | 4 | 58298 |
| 20MU9004NPS | 7/7/2021 | 533203 | 533203 | 1065951 | 1064122 | 1064122 | 4234.91 | 2 | 19593 |
| 20MU9007NPS | 7/7/2021 | 217659 | 217659 | 434997 | 434313 | 434313 | 1737.17 | 15 | 5222 |
| 20MU9008NPS | 7/7/2021 | 527095 | 527095 | 1053545 | 1052217 | 1052217 | 4174.94 | 4 | 13160 |
| 20MU9011NPS | 7/7/2021 | 323435 | 323435 | 646408 | 645518 | 645518 | 2583.46 | 1 | 8676 |
| 20MU9014NPS | 7/7/2021 | 322939 | 322939 | 645418 | 644433 | 644433 | 2552.20 | 1 | 8813 |
| 20MU9015NPS | 7/7/2021 | 284935 | 284935 | 569650 | 568944 | 568944 | 2225.01 | 21 | 8393 |
| 20MU9018NPS | 7/8/2021 | 398813 | 398813 | 797204 | 796303 | 796303 | 3135.22 | 1 | 11886 |
| 20MU9023NPS | 7/8/2021 | 247665 | 247665 | 494967 | 472104 | 472104 | 1880.77 | 23 | 10737 |
| 20MU9024NPS | 7/7/2021 | 922999 | 922999 | 1845323 | 1841007 | 1841007 | 7192.17 | 1 | 43582 |
| 20MU9026NPS | 7/8/2021 | 456532 | 456532 | 912555 | 911758 | 911758 | 3617.91 | 39 | 13112 |
| 20MU9027NPS | 7/8/2021 | 517668 | 517668 | 1034713 | 1028908 | 1028908 | 4068.24 | 1 | 19963 |
| 20MU9028NPS | 7/8/2021 | 546662 | 546662 | 1092679 | 1091633 | 1091633 | 4313.55 | 14 | 17324 |
| 20MU9030NPS | 7/8/2021 | 486172 | 486172 | 971736 | 970821 | 970821 | 3878.90 | 19 | 13685 |
| 20MU9031NPS | 7/8/2021 | 395936 | 395936 | 791482 | 790591 | 790591 | 3069.72 | 1 | 11471 |
| 20MU9034NPS | 7/8/2021 | 500672 | 500672 | 1000724 | 999439 | 999439 | 3924.02 | 1 | 18844 |
| 20MU9038NPS | 7/8/2021 | 370948 | 370948 | 741494 | 740559 | 740559 | 2952.27 | 1 | 10802 |
| 20MU9042NPS | 7/8/2021 | 1149767 | 1149767 | 2298335 | 2295798 | 2295798 | 9093.76 | 1 | 40233 |
| 20MU9046NPS | 7/8/2021 | 361511 | 361511 | 722413 | 720674 | 720674 | 2860.08 | 20 | 11857 |
| 20MU9048NPS | 7/7/2021 | 561350 | 561350 | 1122140 | 1121094 | 1121094 | 4453.13 | 1 | 16077 |
| 20MU9049NPS | 7/7/2021 | 1330534 | 1330534 | 2659788 | 2623162 | 2623162 | 10336.42 | 1 | 50205 |
| 20MU9050NPS | 7/9/2021 | 550032 | 550032 | 1099339 | 1098067 | 1098067 | 4371.36 | 14 | 16046 |
| 20MU9052NPS | 7/9/2021 | 384283 | 384283 | 768175 | 767489 | 767489 | 3028.06 | 1 | 10990 |
| 20MU9053NPS | 7/7/2021 | 706034 | 706034 | 1411070 | 1408421 | 1408421 | 5595.20 | 1 | 22195 |
| 20MU9054NPS | 7/9/2021 | 566531 | 566531 | 1132381 | 1131256 | 1131256 | 4465.91 | 1 | 18573 |
| 20MU9058NPS | 7/9/2021 | 641128 | 641128 | 1281546 | 1280408 | 1280408 | 5013.78 | 29 | 20096 |
| 20MU9060NPS | 7/9/2021 | 643088 | 643088 | 1285514 | 1284101 | 1284101 | 5088.37 | 1 | 18103 |
| 20MU9062NPS | 7/9/2021 | 725585 | 725585 | 1450562 | 1448890 | 1448890 | 5739.39 | 1 | 28501 |
| 20MU9063NPS | 7/9/2021 | 514941 | 514941 | 1029196 | 1028062 | 1028062 | 4125.83 | 115 | 15986 |
| 20MU9064NPS | 7/9/2021 | 283802 | 283802 | 567187 | 566529 | 566529 | 2280.34 | 21 | 7335 |
| 20MU9066NPS | 7/9/2021 | 388768 | 388768 | 777039 | 764213 | 764213 | 3032.52 | 1 | 12405 |
| 20MU9067NPS | 7/9/2021 | 475827 | 475827 | 951162 | 950015 | 950015 | 3753.36 | 14 | 15163 |
| 20MU9068NPS | 7/9/2021 | 518018 | 518018 | 1035580 | 1034191 | 1034191 | 4012.09 | 1 | 17057 |
| 20MU9069NPS | 7/9/2021 | 50799 | 50799 | 100907 | 100418 | 100418 | 403.12 | 6 | 1466 |
| 20MU9070NPS | 7/9/2021 | 321960 | 321960 | 643567 | 642785 | 642785 | 2585.81 | 1 | 8001 |
| 20MU9074NPS | 7/9/2021 | 473105 | 473105 | 945629 | 926617 | 926617 | 3667.28 | 1 | 15457 |
| 20MU9075NPS | 7/10/2021 | 570185 | 570185 | 1139697 | 1120508 | 1120508 | 4426.83 | 10 | 20855 |
| 20MU9086NPS | 7/10/2021 | 428914 | 428914 | 857459 | 856815 | 856815 | 3368.58 | 8 | 16368 |
| 20MU9089NPS | 7/11/2021 | 584155 | 584155 | 1167629 | 1127780 | 1127780 | 4455.54 | 4 | 21254 |
| 20MU9092NPS | 7/11/2021 | 426542 | 426542 | 852532 | 851546 | 851546 | 3382.53 | 25 | 17353 |
| 20MU9096NPS | 7/11/2021 | 328383 | 328383 | 656257 | 655500 | 655500 | 2605.71 | 19 | 14496 |
| 20MU9097NPS | 7/11/2021 | 510278 | 510278 | 1019906 | 1018268 | 1018268 | 4040.01 | 23 | 16922 |
| 20MU9098NPS | 7/11/2021 | 451991 | 451991 | 903346 | 897600 | 897600 | 3552.46 | 1 | 19348 |
| 20MU9102NPS | 7/12/2021 | 348089 | 348089 | 695792 | 694943 | 694943 | 2731.78 | 22 | 9946 |
| 20MU9104NPS | 7/12/2021 | 718965 | 718965 | 1437390 | 1435750 | 1435750 | 5481.13 | 1 | 27332 |
| 20MU9107NPS | 7/12/2021 | 720874 | 720874 | 1440895 | 1439113 | 1439113 | 5600.95 | 1 | 25906 |
| 20MU9108NPS | 7/12/2021 | 678376 | 678376 | 1356053 | 1346831 | 1346831 | 5370.94 | 1 | 30115 |
| 20MU9109NPS | 7/12/2021 | 381101 | 381101 | 761611 | 760790 | 760790 | 3027.73 | 1 | 12070 |
| 20MU9111NPS | 7/12/2021 | 470181 | 470181 | 939818 | 939008 | 939008 | 3725.43 | 1 | 13597 |
| 20MU9112NPS | 7/12/2021 | 664329 | 664329 | 1327897 | 1326505 | 1326505 | 5241.18 | 1 | 29256 |
| 20MU9114NPS | 7/12/2021 | 402113 | 402113 | 803745 | 801868 | 801868 | 3172.10 | 1 | 15062 |
| 20MU9115NPS | 7/12/2021 | 365852 | 365852 | 731321 | 730707 | 730707 | 2907.37 | 2 | 11817 |
| 20MU9117NPS | 7/12/2021 | 566077 | 566077 | 1131501 | 1130203 | 1130203 | 4481.03 | 1 | 21693 |
| 20MU9122NPS | 7/12/2021 | 490954 | 490954 | 981460 | 980293 | 980293 | 3741.57 | 1 | 19259 |
| 20MU9124NPS | 7/12/2021 | 113110 | 113110 | 226010 | 213539 | 213539 | 845.53 | 2 | 9271 |
| 20MU9125NPS | 7/12/2021 | 452901 | 452901 | 905202 | 902884 | 902884 | 3582.46 | 1 | 13557 |
| 20MU9130NPS | 7/12/2021 | 457164 | 457164 | 913951 | 913354 | 913354 | 3607.54 | 6 | 28886 |
| 20MU9132NPS | 7/13/2021 | 458238 | 458238 | 915914 | 914837 | 914837 | 3632.58 | 14 | 15061 |
| 20MU9133NPS | 7/13/2021 | 275076 | 275076 | 549641 | 525036 | 525036 | 2070.09 | 1 | 20301 |
| 20MU9134NPS | 7/13/2021 | 440836 | 440836 | 880917 | 879730 | 879730 | 3503.30 | 2 | 12472 |
| 20MU9135NPS | 7/13/2021 | 495328 | 495328 | 990163 | 989238 | 989238 | 3934.33 | 1 | 17398 |
| 20MU9137NPS | 7/13/2021 | 440760 | 440760 | 881084 | 880265 | 880265 | 3452.57 | 1 | 16019 |
| 20MU9138NPS | 7/13/2021 | 957689 | 957689 | 1914241 | 1912033 | 1912033 | 7602.10 | 1 | 27598 |
| 20MU9139NPS | 7/13/2021 | 217069 | 217069 | 433953 | 433577 | 433577 | 1687.27 | 11 | 9397 |
| 20MU9140NPS | 7/13/2021 | 566066 | 566066 | 1131033 | 1129701 | 1129701 | 4489.76 | 1 | 17751 |
| 20MU9141NPS | 7/13/2021 | 372934 | 372934 | 745363 | 744131 | 744131 | 2941.46 | 1 | 15633 |
| 20MU9142NPS | 7/13/2021 | 554080 | 554080 | 1107603 | 1106610 | 1106610 | 4351.60 | 11 | 20687 |
| 20MU9144NPS | 7/13/2021 | 512876 | 512876 | 1025173 | 1011421 | 1011421 | 4067.43 | 8 | 16863 |
| 20MU9145NPS | 7/13/2021 | 325095 | 325095 | 649677 | 648933 | 648933 | 2616.08 | 1 | 8637 |
| 20MU9146NPS | 7/13/2021 | 622737 | 622737 | 1244624 | 1243231 | 1243231 | 4926.97 | 2 | 21729 |
| 20MU9150NPS | 7/13/2021 | 146370 | 146370 | 292614 | 292404 | 292404 | 1144.58 | 1 | 5636 |
| 20MU9152NPS | 7/13/2021 | 422484 | 422484 | 844413 | 843656 | 843656 | 3347.25 | 1 | 12694 |
| 20MU9153NPS | 7/13/2021 | 767201 | 767201 | 1533760 | 1532452 | 1532452 | 5987.97 | 8 | 25334 |
| 20MU9157NPS | 7/14/2021 | 689979 | 689979 | 1379141 | 1377814 | 1377814 | 5387.86 | 1 | 19875 |
| 20MU9159NPS | 7/14/2021 | 440281 | 440281 | 880038 | 874675 | 874675 | 3454.16 | 1 | 15270 |
| 20MU9163NPS | 7/14/2021 | 388839 | 388839 | 777279 | 776236 | 776236 | 3019.93 | 9 | 12369 |
| 20MU9165NPS | 7/14/2021 | 532321 | 532321 | 1064021 | 1062674 | 1062674 | 4223.47 | 2 | 14396 |
| 20MU9169NPS | 7/14/2021 | 390234 | 390234 | 780003 | 779136 | 779136 | 3066.51 | 10 | 12043 |
| 20MU9178NPS | 7/15/2021 | 420186 | 420186 | 839915 | 838991 | 838991 | 3342.48 | 23 | 13002 |
| 20MU9179NPS | 7/15/2021 | 507488 | 507488 | 1014520 | 1013841 | 1013841 | 3986.96 | 9 | 30747 |
| 20MU9180NPS | 7/15/2021 | 753874 | 753874 | 1506869 | 1505413 | 1505413 | 5959.41 | 1 | 22524 |
| 20MU9181NPS | 7/15/2021 | 711115 | 711115 | 1421409 | 1419984 | 1419984 | 5624.80 | 23 | 30611 |
| 20MU9182NPS | 7/15/2021 | 339422 | 339422 | 678421 | 677276 | 677276 | 2686.23 | 1 | 11707 |
| 20MU9184NPS | 7/15/2021 | 468703 | 468703 | 936951 | 935969 | 935969 | 3673.40 | 1 | 15978 |
| 20MU9185NPS | 7/15/2021 | 310658 | 310658 | 620858 | 619776 | 619776 | 2463.98 | 22 | 10835 |
| 20MU9186NPS | 7/15/2021 | 445811 | 445811 | 891117 | 889999 | 889999 | 3518.44 | 1 | 12629 |
| 20MU9188NPS | 7/15/2021 | 334053 | 334053 | 667602 | 666857 | 666857 | 2687.78 | 9 | 8790 |
| 20MU9190NPS | 7/15/2021 | 330992 | 330992 | 661588 | 660742 | 660742 | 2628.14 | 1 | 10214 |
| 20MU9194NPS | 7/15/2021 | 452673 | 452673 | 904868 | 904034 | 904034 | 3592.64 | 1 | 12209 |
| 20MU9195NPS | 7/15/2021 | 148915 | 148915 | 297668 | 297450 | 297450 | 1187.47 | 12 | 4496 |
| 20MU9196NPS | 7/15/2021 | 1238684 | 1238684 | 2475736 | 2473674 | 2473674 | 9209.77 | 1 | 32156 |
| 20MU9199NPS | 7/16/2021 | 704882 | 704882 | 1408591 | 1407054 | 1407054 | 5639.56 | 36 | 22865 |
| 20MU9201NPS | 7/16/2021 | 675928 | 675928 | 1350690 | 1349044 | 1349044 | 5355.76 | 1 | 19816 |
| 20MU9212NPS | 7/16/2021 | 297577 | 297577 | 594092 | 593181 | 593181 | 2408.32 | 1 | 7040 |
| 20MU9215NPS | 7/16/2021 | 312958 | 312958 | 625530 | 614719 | 614719 | 2412.04 | 1 | 13761 |
| 20MU9217NPS | 7/16/2021 | 946702 | 946702 | 1891500 | 1889061 | 1889061 | 7510.32 | 1 | 23115 |
| 20MU9218NPS | 7/16/2021 | 271209 | 271209 | 541954 | 541111 | 541111 | 2194.95 | 1 | 6207 |
| 20MU9219NPS | 7/16/2021 | 470650 | 470650 | 940420 | 939076 | 939076 | 3741.98 | 14 | 10490 |
| 20MU9221NPS | 7/16/2021 | 269402 | 269402 | 538262 | 537533 | 537533 | 2189.43 | 25 | 5453 |
| 20MU9222NPS | 7/16/2021 | 413633 | 413633 | 825841 | 824369 | 824369 | 3362.28 | 1 | 8978 |
| 20MU9223NPS | 7/16/2021 | 332234 | 332234 | 663817 | 663117 | 663117 | 2643.05 | 1 | 9270 |
| 20MU9225NPS | 7/16/2021 | 300729 | 300729 | 600904 | 600257 | 600257 | 2384.46 | 17 | 7925 |
| 20MU9226NPS | 7/17/2021 | 392198 | 392198 | 783653 | 782635 | 782635 | 3167.16 | 3 | 9736 |
| 20MU9227NPS | 7/17/2021 | 373393 | 373393 | 746131 | 745248 | 745248 | 2970.52 | 9 | 8868 |
| 20MU9228NPS | 7/17/2021 | 353835 | 353835 | 707198 | 706400 | 706400 | 2829.09 | 1 | 8519 |
| 20MU9229NPS | 7/17/2021 | 387161 | 387161 | 773510 | 772554 | 772554 | 2885.82 | 14 | 10927 |
| 20MU9230NPS | 7/17/2021 | 1047284 | 1047284 | 2093485 | 2088335 | 2088335 | 7435.95 | 1 | 34847 |
| 20MU9231NPS | 7/17/2021 | 363316 | 363316 | 726134 | 725273 | 725273 | 2896.45 | 1 | 9783 |
| 20MU9238NPS | 7/17/2021 | 476753 | 476753 | 952991 | 947964 | 947964 | 3723.90 | 35 | 17334 |
| 20MU9239NPS | 7/17/2021 | 390271 | 401994 | 791775 | 787249 | 787249 | 3147.83 | 1 | 12182 |
| 20MU9240NPS | 7/17/2021 | 366706 | 366706 | 732854 | 732133 | 732133 | 2911.91 | 26 | 11172 |
| 20MU9244NPS | 7/18/2021 | 345519 | 345519 | 690615 | 689521 | 689521 | 2529.84 | 14 | 7362 |
| 20MU9248NPS | 7/18/2021 | 430161 | 430161 | 859802 | 857371 | 857371 | 3397.63 | 29 | 14691 |
| 20MU9249NPS | 7/18/2021 | 255090 | 255090 | 509689 | 509003 | 509003 | 2078.62 | 24 | 5882 |
| 20MU9251NPS | 7/18/2021 | 386496 | 386496 | 772507 | 759849 | 759849 | 1217.64 | 1 | 10237 |
| 20MU9262NPS | 7/19/2021 | 332744 | 332744 | 664681 | 663539 | 663539 | 2661.34 | 1 | 7972 |
| 20MU9264NPS | 7/19/2021 | 378104 | 378104 | 755815 | 753338 | 753338 | 2971.23 | 1 | 13557 |
| 20MU9266NPS | 7/19/2021 | 292888 | 292888 | 585285 | 584412 | 584412 | 2324.60 | 30 | 6521 |
| 20MU9269NPS | 7/19/2021 | 433477 | 433477 | 866242 | 865402 | 865402 | 3448.58 | 1 | 12993 |
| 20MU9272NPS | 7/19/2021 | 105926 | 105926 | 211645 | 211339 | 211339 | 841.35 | 10 | 2545 |
| 20MU9273NPS | 7/19/2021 | 245457 | 245457 | 490324 | 489713 | 489713 | 1951.65 | 3 | 5863 |
| 20MU9274NPS | 7/19/2021 | 573670 | 573670 | 1146510 | 1145324 | 1145324 | 4575.20 | 1 | 14857 |
| 20MU9277NPS | 7/19/2021 | 219773 | 219773 | 439151 | 438535 | 438535 | 1756.58 | 1 | 5507 |
| 20MU9278NPS | 7/19/2021 | 604848 | 604848 | 1208878 | 1207281 | 1207281 | 4906.94 | 53 | 18462 |
| 20MU9280NPS | 7/19/2021 | 335794 | 335794 | 671270 | 661407 | 661407 | 2598.53 | 8 | 18719 |
| 20MU9282NPS | 7/19/2021 | 239223 | 239223 | 478059 | 477374 | 477374 | 1916.86 | 1 | 5276 |
| 20MU9287NPS | 7/19/2021 | 630044 | 630044 | 1259141 | 1257800 | 1257800 | 5026.10 | 1 | 16748 |
| 20MU9289NPS | 7/19/2021 | 473566 | 473566 | 946307 | 945194 | 945194 | 3768.44 | 1 | 14353 |
| 20MU9291NPS | 7/19/2021 | 363205 | 363205 | 725752 | 724925 | 724925 | 2900.11 | 81 | 8938 |
| 20MU9292NPS | 7/19/2021 | 401221 | 401221 | 801885 | 801003 | 801003 | 3203.06 | 31 | 11681 |
| 20MU9294NPS | 7/20/2021 | 564097 | 564097 | 1127332 | 1126010 | 1126010 | 4495.37 | 1 | 14493 |
| 20MU9296NPS | 7/20/2021 | 484373 | 484373 | 968125 | 966612 | 966612 | 3843.85 | 1 | 18622 |
| 20MU9302NPS | 7/20/2021 | 785519 | 785519 | 1570168 | 1568193 | 1568193 | 6163.84 | 2 | 22631 |
| 20MU9303NPS | 7/20/2021 | 508478 | 508478 | 1016299 | 995848 | 995848 | 3956.17 | 1 | 30532 |
| 20MU9308NPS | 7/20/2021 | 548062 | 548062 | 1095177 | 1093905 | 1093905 | 4358.35 | 2 | 15405 |
| 20MU9313NPS | 7/20/2021 | 497503 | 497503 | 994272 | 993278 | 993278 | 3946.54 | 1 | 12434 |
| 20MU9314NPS | 7/20/2021 | 376459 | 376459 | 752335 | 751281 | 751281 | 2985.55 | 80 | 8987 |
| 20MU9317NPS | 7/20/2021 | 409720 | 409720 | 818994 | 815672 | 815672 | 3277.20 | 33 | 17527 |
| 20MU9328NPS | 7/20/2021 | 335354 | 335354 | 670046 | 669201 | 669201 | 2670.49 | 1 | 8664 |
| 20MU9330NPS | 7/20/2021 | 373703 | 373703 | 747043 | 742931 | 742931 | 1895.43 | 1 | 11652 |
| 20MU9336NPS | 7/20/2021 | 445058 | 445058 | 889382 | 888062 | 888062 | 3611.12 | 48 | 10681 |
| 20MU9337NPS | 7/20/2021 | 339713 | 339713 | 679031 | 678189 | 678189 | 2701.48 | 10 | 8449 |
| 20MU9339NPS | 7/21/2021 | 272485 | 272485 | 544646 | 520954 | 520954 | 2058.07 | 1 | 9004 |
| 20MU9340NPS | 7/20/2021 | 427033 | 427033 | 853516 | 852407 | 852407 | 3380.81 | 1 | 12376 |
| 20MU9341NPS | 7/21/2021 | 429405 | 429405 | 858269 | 857159 | 857159 | 3398.12 | 13 | 20352 |
| 20MU9342NPS | 7/21/2021 | 472331 | 472331 | 944066 | 942864 | 942864 | 3784.92 | 1 | 12464 |
| 20MU9345NPS | 7/21/2021 | 299771 | 299771 | 598935 | 598157 | 598157 | 2399.55 | 23 | 6324 |
| 20MU9346NPS | 7/21/2021 | 456365 | 456365 | 912207 | 911021 | 911021 | 3603.39 | 28 | 15714 |
| 20MU9354NPS | 7/21/2021 | 299071 | 299071 | 597677 | 596960 | 596960 | 2429.83 | 22 | 7043 |
| 20MU9355NPS | 7/21/2021 | 445112 | 445112 | 889729 | 885437 | 885437 | 3559.99 | 1 | 19014 |
| 20MU9356NPS | 7/21/2021 | 600990 | 600990 | 1201345 | 1199354 | 1199354 | 4730.59 | 1 | 20557 |
| 20MU9357NPS | 7/21/2021 | 556315 | 556315 | 1111769 | 1110506 | 1110506 | 4399.01 | 1 | 24766 |
| 20MU9360NPS | 7/21/2021 | 496266 | 496266 | 991686 | 990208 | 990208 | 4014.80 | 1 | 11155 |
| 20MU9362NPS | 7/21/2021 | 460588 | 460588 | 920621 | 919412 | 919412 | NA | NA | NA |
| 20MU9367NPS | 7/22/2021 | 316880 | 316880 | 633200 | 632269 | 632269 | 2551.53 | 29 | 7531 |
| 20MU9369NPS | 7/22/2021 | 363902 | 363902 | 727269 | 726339 | 726339 | 2889.14 | 1 | 9155 |
| 20MU9371NPS | 7/23/2021 | 529012 | 529012 | 1057258 | 1055843 | 1055843 | 4196.85 | 1 | 16073 |
| 20MU9372NPS | 7/23/2021 | 345260 | 345260 | 689947 | 689161 | 689161 | 2761.00 | 33 | 10427 |
| 20MU9374NPS | 7/23/2021 | 337051 | 337051 | 673390 | 672568 | 672568 | 2707.03 | 56 | 6747 |
| 20MU9376NPS | 7/22/2021 | 343318 | 343318 | 686248 | 685282 | 685282 | 2707.44 | 1 | 11108 |
| 20MU9381NPS | 7/23/2021 | 818356 | 818356 | 1635676 | 1630786 | 1630786 | 6521.43 | 1 | 31978 |
| 20MU9383NPS | 7/23/2021 | 378081 | 378081 | 755599 | 754750 | 754750 | 3037.30 | 1 | 8912 |
| 20MU9385NPS | 7/23/2021 | 417599 | 417599 | 834465 | 833718 | 833718 | 3319.56 | 16 | 12996 |
| 20MU9386NPS | 7/23/2021 | 322711 | 322711 | 644954 | 643820 | 643820 | 2573.82 | 17 | 8313 |
| 20MU9387NPS | 7/24/2021 | 449665 | 449665 | 898796 | 891968 | 891968 | 3505.88 | 1 | 30687 |
| 20MU9390NPS | 7/24/2021 | 544551 | 544551 | 1088203 | 1087057 | 1087057 | 4348.65 | 33 | 13589 |
| 20MU9393NPS | 7/24/2021 | 299021 | 299021 | 597519 | 596700 | 596700 | 2423.24 | 29 | 7187 |
| 20MU9394NPS | 7/23/2021 | 620582 | 620582 | 1240591 | 1236518 | 1236518 | 4884.74 | 11 | 29550 |
| 20MU9396NPS | 7/24/2021 | 289044 | 289044 | 577657 | 577063 | 577063 | 2296.59 | 11 | 7429 |
| 20MU9397NPS | 7/24/2021 | 456993 | 456993 | 913357 | 912517 | 912517 | 3633.83 | 1 | 11564 |
| 20MU9400NPS | 7/25/2021 | 475353 | 475353 | 950213 | 949215 | 949215 | 3759.98 | 1 | 12734 |
| 20MU9401NPS | 7/25/2021 | 99693 | 99693 | 199166 | 198810 | 198810 | 804.59 | 1 | 2019 |
| 20MU9407NPS | 7/26/2021 | 272361 | 272361 | 544334 | 543456 | 543456 | 2179.62 | 1 | 6046 |
| 20MU9408NPS | 7/26/2021 | 305287 | 305287 | 609991 | 609155 | 609155 | 2450.58 | 28 | 7886 |
| 20MU9410NPS | 7/26/2021 | 529313 | 529313 | 1057650 | 1056092 | 1056092 | 4227.69 | 1 | 11188 |
| 20MU9411NPS | 7/26/2021 | 391880 | 391880 | 783126 | 778541 | 778541 | 3076.35 | 2 | 11921 |
| 20MU9413NPS | 7/26/2021 | 597513 | 597513 | 1193975 | 1192197 | 1192197 | 4757.35 | 1 | 14105 |
| 20MU9418NPS | 7/26/2021 | 427726 | 427726 | 854823 | 802842 | 802842 | 3156.62 | 2 | 12899 |
| 20MU9425NPS | 7/26/2021 | 236157 | 236157 | 471954 | 471238 | 471238 | 1894.04 | 1 | 5847 |
| 20MU9427NPS | 7/26/2021 | 530398 | 530398 | 1059990 | 1022654 | 1022654 | 4040.86 | 1 | 12600 |
| 20MU9431NPS | 7/26/2021 | 162685 | 162685 | 325047 | 324592 | 324592 | 1323.66 | 1 | 3716 |
| 20MU9432NPS | 7/26/2021 | 361173 | 361173 | 721631 | 720573 | 720573 | 2877.64 | 1 | 8749 |
| 20MU9433NPS | 7/26/2021 | 387059 | 387059 | 773519 | 772100 | 772100 | 3074.96 | 1 | 8991 |
| 20MU9435NPS | 7/26/2021 | 703825 | 703825 | 1406538 | 1403966 | 1403966 | 5546.03 | 1 | 21404 |
| 20MU9437NPS | 7/26/2021 | 473234 | 473234 | 945632 | 944193 | 944193 | 3756.85 | 2 | 11649 |
| 20MU9438NPS | 7/26/2021 | 366885 | 366885 | 733045 | 731249 | 731249 | 2908.90 | 23 | 9135 |
| 20MU9441NPS | 7/26/2021 | 311148 | 311148 | 621834 | 620988 | 620988 | 2458.20 | 1 | 8498 |
| 20MU9442NPS | 7/26/2021 | 300126 | 300126 | 599754 | 598887 | 598887 | 1875.48 | 14 | 5084 |
| 20MU9449NPS | 7/27/2021 | 215818 | 215818 | 431254 | 430563 | 430563 | 1748.38 | 1 | 5316 |
| 20MU9452NPS | 7/27/2021 | 365398 | 365398 | 730496 | 716886 | 716886 | 2827.69 | 34 | 17286 |
| 20MU9453NPS | 7/27/2021 | 422205 | 422205 | 843931 | 839612 | 839612 | 3307.46 | 39 | 14292 |
| 20MU9454NPS | 7/27/2021 | 487700 | 487700 | 974591 | 973439 | 973439 | 3943.35 | 1 | 15448 |
| 20MU9455NPS | 7/27/2021 | 418341 | 418341 | 835981 | 834927 | 834927 | 3332.44 | 1 | 10708 |
| 20MU9457NPS | 7/27/2021 | 455979 | 455979 | 911548 | 910097 | 910097 | 3590.54 | 56 | 18642 |
| 20MU9458NPS | 7/27/2021 | 533831 | 533831 | 1066986 | 864590 | 864590 | 4074.76 | 34 | 19160 |
| 20MU9459NPS | 7/27/2021 | 265332 | 265332 | 530366 | 509502 | 509502 | 2011.67 | 2 | 10950 |
| 20MU9463NPS | 7/27/2021 | 242698 | 242698 | 485002 | 484331 | 484331 | 1926.08 | 5 | 6619 |
| 20MU9464NPS | 7/27/2021 | 410532 | 410532 | 820561 | 818724 | 818724 | 3226.88 | 31 | 18256 |
| 20MU9468NPS | 7/27/2021 | 214646 | 214646 | 429036 | 428324 | 428324 | 1723.46 | 1 | 5521 |
| 20MU9473NPS | 7/27/2021 | 314414 | 314414 | 628448 | 627702 | 627702 | 2517.21 | 2 | 7552 |
| 20MU9475NPS | 7/27/2021 | 341502 | 341502 | 682480 | 681650 | 681650 | 2698.31 | 1 | 10089 |
| 20MU9479NPS | 7/27/2021 | 262975 | 262975 | 525686 | 517721 | 517721 | 2026.26 | 1 | 9820 |
| 20MU9486NPS | 7/28/2021 | 317533 | 317533 | 634541 | 633455 | 633455 | 2502.19 | 1 | 9118 |
| 20MU9487NPS | 7/28/2021 | 250671 | 250671 | 500924 | 500150 | 500150 | 1996.67 | 1 | 6736 |
| 20MU9488NPS | 7/28/2021 | 436781 | 436781 | 872990 | 871595 | 871595 | 3521.45 | 1 | 24452 |
| 20MU9489NPS | 7/28/2021 | 308621 | 308621 | 616735 | 615897 | 615897 | 2511.63 | 112 | 8304 |
| 20MU9491NPS | 7/28/2021 | 258926 | 258926 | 517597 | 484987 | 484987 | 1917.28 | 4 | 10799 |
| 20MU9492NPS | 7/28/2021 | 344777 | 344777 | 689173 | 681291 | 681291 | 2694.15 | 1 | 12477 |
| 20MU9498NPS | 7/28/2021 | 306260 | 306260 | 611835 | 611015 | 611015 | 2482.63 | 27 | 7521 |
| 20MU9500NPS | 7/28/2021 | 226705 | 226705 | 453056 | 452427 | 452427 | 1815.16 | 1 | 5503 |
| 20MU9501NPS | 7/28/2021 | 424924 | 424924 | 849381 | 843895 | 843895 | 3311.74 | 1 | 27577 |
| 20MU9502NPS | 7/28/2021 | 354824 | 354824 | 709260 | 706373 | 706373 | 2852.10 | 39 | 15629 |
| 20MU9504NPS | 7/29/2021 | 519368 | 519368 | 1037987 | 1036659 | 1036659 | 4117.32 | 22 | 17209 |
| 20MU9506NPS | 7/29/2021 | 311011 | 311011 | 621491 | 620708 | 620708 | 2470.44 | 1 | 8333 |
| 20MU9507NPS | 7/22/2021 | 387308 | 387308 | 773244 | 772042 | 772042 | 3067.83 | 1 | 9836 |
| 20MU9511NPS | 7/29/2021 | 271013 | 271013 | 541694 | 540662 | 540662 | 2156.57 | 1 | 7280 |
| 20MU9513NPS | 7/29/2021 | 361735 | 361735 | 723123 | 718215 | 718215 | 2823.75 | 11 | 15955 |
| 20MU9514NPS | 7/29/2021 | 194492 | 194492 | 388728 | 388179 | 388179 | 1528.28 | 6 | 5595 |
| 20MU9515NPS | 7/29/2021 | 273599 | 273599 | 546697 | 545939 | 545939 | 2207.51 | 25 | 6678 |
| 20MU9516NPS | 7/29/2021 | 435778 | 435778 | 870986 | 870078 | 870078 | 3451.61 | 1 | 12972 |
| 20MU9517NPS | 7/29/2021 | 375145 | 375145 | 749737 | 748866 | 748866 | 2966.90 | 16 | 10798 |
| 20MU9520NPS | 7/29/2021 | 378395 | 378395 | 756448 | 754328 | 754328 | 2976.30 | 19 | 36374 |
| 20MU9521NPS | 7/29/2021 | 334506 | 334506 | 668604 | 667805 | 667805 | 2648.04 | 1 | 9891 |
| 20MU9523NPS | 7/29/2021 | 327375 | 327375 | 654404 | 648283 | 648283 | 2549.29 | 1 | 13074 |
| 20MU9527NPS | 7/29/2021 | 372879 | 372879 | 745351 | 741379 | 741379 | 2928.45 | 1 | 14020 |
| 20MU9529NPS | 7/29/2021 | 298600 | 298600 | 596675 | 595919 | 595919 | 2416.91 | 1 | 7757 |
| 20MU9530NPS | 7/29/2021 | 320967 | 320967 | 641504 | 634773 | 634773 | 2530.13 | 2 | 14023 |
| 20MU9539NPS | 7/29/2021 | 376460 | 376460 | 752477 | 739982 | 739982 | 2957.30 | 36 | 19076 |
| 20MU9541NPS | 7/29/2021 | 277834 | 277834 | 555180 | 554534 | 554534 | 2212.86 | 21 | 7877 |
| 20MU9542NPS | 7/29/2021 | 313353 | 313353 | 626341 | 617252 | 617252 | 2457.14 | 31 | 13221 |
| 20MU9543NPS | 7/29/2021 | 476572 | 476572 | 952579 | 950261 | 950261 | 3794.62 | 2 | 21925 |
| 20MU9544NPS | 7/29/2021 | 242135 | 242135 | 483944 | 482551 | 482551 | 1929.70 | 1 | 6623 |
| 20MU9545NPS | 7/29/2021 | 303470 | 303470 | 606385 | 605474 | 605474 | 2402.57 | 1 | 8305 |
| 20MU9548NPS | 7/30/2021 | 433124 | 433124 | 865687 | 864158 | 864158 | 3420.78 | 4 | 17822 |
| 20MU9549NPS | 7/30/2021 | 298581 | 298581 | 596704 | 595782 | 595782 | 2367.24 | 1 | 7366 |
| 20MU9551NPS | 7/30/2021 | 525544 | 525544 | 1050385 | 1049007 | 1049007 | 4211.31 | 1 | 18264 |
| 20MU9555NPS | 7/30/2021 | 511751 | 511751 | 1023022 | 1020732 | 1020732 | 4016.10 | 37 | 26085 |
| 20MU9558NPS | 7/30/2021 | 397369 | 397369 | 794311 | 781505 | 781505 | 3067.37 | 1 | 14261 |
| 20MU9560NPS | 7/30/2021 | 313015 | 313015 | 625483 | 624409 | 624409 | 2481.70 | 1 | 8377 |
| 20MU9566NPS | 7/30/2021 | 308723 | 308723 | 616888 | 615992 | 615992 | 2440.60 | 1 | 8240 |
| 20MU9568NPS | 7/30/2021 | 436241 | 436241 | 871974 | 857328 | 857328 | 3361.74 | 1 | 26543 |
| 20MU9569NPS | 7/30/2021 | 519955 | 519955 | 1039170 | 1036652 | 1036652 | 4124.46 | 1 | 16545 |
| 20MU9583NPS | 8/5/2021 | 467396 | 467396 | 934281 | 933305 | 933305 | 3701.68 | 1 | 13164 |
| 20MU9584NPS | 8/5/2021 | 355891 | 355891 | 711497 | 710381 | 710381 | 2816.01 | 3 | 19169 |
| 20MU9585NPS | 8/5/2021 | 465644 | 465644 | 930665 | 929821 | 929821 | 3699.44 | 24 | 11999 |
| 20MU9587NPS | 8/6/2021 | 673224 | 673224 | 1345712 | 1344032 | 1344032 | 5320.39 | 1 | 31918 |
| 20MU9589NPS | 8/9/2021 | 212344 | 212344 | 424461 | 419943 | 419943 | 1665.21 | 1 | 6334 |
| 20MU9590NPS | 8/9/2021 | 268776 | 268776 | 537055 | 536364 | 536364 | 2171.90 | 1 | 6545 |
| 20MU9591NPS | 8/9/2021 | 877842 | 877842 | 1754141 | 1752054 | 1752054 | 7057.13 | 1 | 20402 |
| 20MU9592NPS | 8/9/2021 | 357674 | 357674 | 714999 | 712266 | 712266 | 2819.04 | 1 | 15722 |
| 20MU9596NPS | 8/12/2021 | 432756 | 432756 | 864995 | 864119 | 864119 | 3403.88 | 1 | 13701 |
| 20MU9598NPS | 8/13/2021 | 238431 | 238431 | 476582 | 475896 | 475896 | 1882.07 | 15 | 5841 |
| 20MU9602NPS | 8/15/2021 | 479917 | 479917 | 959333 | 909730 | 909730 | 3606.08 | 36 | 14869 |
| 20MU9603NPS | 8/16/2021 | 1355526 | 1355526 | 2709886 | 2636730 | 2636730 | 10422.82 | 1 | 42493 |
| 20MU9604NPS | 8/11/2021 | 86162 | 86162 | 172210 | 167997 | 167997 | 664.78 | 3 | 5271 |
| 20MU9605NPS | 8/16/2021 | 852523 | 852523 | 1704138 | 1702237 | 1702237 | 6817.97 | 1 | 27334 |
| 20MU9606NPS | 8/16/2021 | 510973 | 510973 | 1021426 | 1020301 | 1020301 | 4049.35 | 1 | 14847 |
| 20MU9608NPS | 8/16/2021 | 473501 | 473501 | 946638 | 931595 | 931595 | 3666.76 | 1 | 14304 |
| 20MU9610NPS | 8/16/2021 | 294090 | 294090 | 587834 | 587133 | 587133 | 2325.61 | 1 | 8478 |
| 20MU9611NPS | 8/17/2021 | 590112 | 590112 | 1179585 | 1178072 | 1178072 | 4655.63 | 1 | 17935 |
| 20MU9612NPS | 8/17/2021 | 914662 | 914662 | 1828187 | 1825692 | 1825692 | 7187.32 | 1 | 28042 |
| 20MU9615NPS | 8/17/2021 | 352455 | 352455 | 704518 | 693085 | 693085 | 2746.61 | 33 | 12157 |
| 20MU9616NPS | 8/18/2021 | 255850 | 255850 | 511431 | 510786 | 510786 | 2023.25 | 1 | 7013 |
| 20MU9617NPS | 8/18/2021 | 165647 | 165647 | 331024 | 308166 | 308166 | 1214.42 | 1 | 6352 |
| 20MU9618NPS | 8/20/2021 | 965734 | 965734 | 1930632 | 1914825 | 1914825 | 7583.72 | 2 | 39704 |
| 20MU9619NPS | 8/20/2021 | 592449 | 592449 | 1184190 | 1138118 | 1138118 | 4491.85 | 1 | 16669 |
| 20MU9620NPS | 8/20/2021 | 271868 | 271868 | 543273 | 542472 | 542472 | 2179.76 | 9 | 6350 |
| 20MU9621NPS | 8/20/2021 | 478867 | 478867 | 957124 | 955915 | 955915 | 3812.79 | 56 | 12540 |
| 20MU9622NPS | 8/20/2021 | 321911 | 321911 | 643295 | 642363 | 642363 | 2551.70 | 1 | 7795 |
| 20MU9625NPS | 8/21/2021 | 492436 | 492436 | 984266 | 926241 | 926241 | 3624.64 | 1 | 19368 |
| 20MU9627NPS | 8/23/2021 | 180182 | 180182 | 360012 | 359483 | 359483 | 1451.11 | 1 | 3924 |
| 20MU9630NPS | 8/23/2021 | 296446 | 296446 | 592437 | 436394 | 436394 | 1717.96 | 24 | 7122 |
| 20MU9631NPS | 8/23/2021 | 301440 | 301440 | 602413 | 601502 | 601502 | 2417.67 | 2 | 7200 |
| 20MU9632NPS | 8/23/2021 | 374234 | 374234 | 747788 | 746625 | 746625 | 2998.75 | 33 | 9197 |
| 20MU9633NPS | 8/23/2021 | 798750 | 798750 | 1596510 | 1581550 | 1581550 | 6255.83 | 1 | 20122 |
| 20MU9634NPS | 8/23/2021 | 238092 | 238092 | 475881 | 475060 | 475060 | 1919.54 | 14 | 5329 |
| 20MU9635NPS | 8/23/2021 | 250947 | 250947 | 501516 | 500657 | 500657 | 2009.35 | 18 | 6194 |
| 20MU9636NPS | 8/23/2021 | 491753 | 491753 | 982782 | 944526 | 944526 | 3732.60 | 1 | 10875 |
| 20MU9637NPS | 8/23/2021 | 385440 | 385440 | 769908 | 768437 | 768437 | 3054.59 | 1 | 9002 |
| 20MU9639NPS | 8/24/2021 | 303401 | 303401 | 606309 | 605104 | 605104 | 2431.67 | 1 | 6426 |
| 20MU9642NPS | 8/24/2021 | 575010 | 575010 | 1149207 | 1111515 | 1111515 | 4382.75 | 1 | 14655 |
| 20MU9643NPS | 8/24/2021 | 341157 | 341157 | 681723 | 680701 | 680701 | 2712.53 | 1 | 7995 |
| 20MU9644NPS | 8/24/2021 | 585962 | 585962 | 1170664 | 1027690 | 1027690 | 4059.15 | 3 | 11767 |
| 20MU9645NPS | 8/24/2021 | 548947 | 548947 | 1096970 | 1075338 | 1075338 | 4244.33 | 69 | 17015 |
| 20MU9646NPS | 8/25/2021 | 605139 | 605139 | 1209451 | 1202740 | 1202740 | 4769.77 | 42 | 14129 |
| 20MU9647NPS | 8/25/2021 | 428219 | 428219 | 855868 | 854575 | 854575 | 3417.86 | 29 | 10465 |
| 20MU9653NPS | 8/26/2021 | 82824 | 82824 | 165484 | 165244 | 165244 | 668.99 | 7 | 1983 |
| 20MU9654NPS | 8/26/2021 | 461472 | 461472 | 922064 | 917780 | 917780 | 3662.96 | 1 | 13662 |
| 20MU9655NPS | 8/26/2021 | 328197 | 328197 | 655719 | 654534 | 654534 | 2609.40 | 27 | 7393 |
| 20MU9656NPS | 8/26/2021 | 214480 | 214480 | 428611 | 427953 | 427953 | 1730.50 | 9 | 5034 |
| 20MU9658NPS | 8/26/2021 | 216221 | 216221 | 432111 | 431464 | 431464 | 1735.01 | 1 | 5090 |
| 20MU9660NPS | 8/26/2021 | 354811 | 354811 | 708962 | 707972 | 707972 | 2860.55 | 1 | 9071 |
| 20MU9661NPS | 8/27/2021 | 262930 | 262930 | 525343 | 524468 | 524468 | 2102.06 | 2 | 5720 |
| 20MU9662NPS | 8/27/2021 | 309656 | 309656 | 618791 | 617903 | 617903 | 2481.22 | 22 | 7162 |
| 20MU9663NPS | 8/27/2021 | 701701 | 701701 | 1401515 | 321089 | 321089 | 1266.06 | 4 | 5799 |
| 20MU9664NPS | 8/27/2021 | 572316 | 572316 | 1143879 | 927613 | 927613 | 3649.48 | 1 | 11870 |
| 20MU9665NPS | 8/27/2021 | 407979 | 407979 | 815140 | 814019 | 814019 | 3296.04 | 1 | 9588 |
| 20MU9666NPS | 8/27/2021 | 366932 | 366932 | 733198 | 732035 | 732035 | 2931.12 | 1 | 8357 |
| 20MU9668NPS | 8/27/2021 | 1342509 | 1342509 | 2683035 | 2675399 | 2675399 | 10628.02 | 1 | 31510 |
| 20MU9669NPS | 8/27/2021 | 522972 | 522972 | 1045054 | 1014618 | 1014618 | 4019.81 | 67 | 11855 |
| 20MU9670NPS | 8/27/2021 | 367523 | 367523 | 734375 | 733279 | 733279 | 2967.17 | 33 | 9347 |
| 20MU9672NPS | 8/28/2021 | 347944 | 347944 | 695356 | 694442 | 694442 | 2775.21 | 1 | 7962 |
| 20MU9674NPS | 8/29/2021 | 392766 | 392766 | 785102 | 770083 | 770083 | 3043.42 | 1 | 23688 |
| 20MU9675NPS | 8/30/2021 | 271662 | 271662 | 543051 | 535840 | 535840 | 2118.56 | 1 | 15527 |
| 20MU9676NPS | 8/30/2021 | 622889 | 622889 | 1245084 | 1244031 | 1244031 | 4938.66 | 3 | 31926 |
| 20MU9677NPS | 8/30/2021 | 644907 | 644907 | 1289072 | 1288000 | 1288000 | 5111.05 | 1 | 24962 |
| 20MU9678NPS | 8/30/2021 | 429690 | 429690 | 858926 | 852990 | 852990 | 3375.46 | 1 | 25095 |
| 20MU9679NPS | 8/30/2021 | 406462 | 406462 | 812431 | 811575 | 811575 | 3210.20 | 1 | 10949 |
| 20MU9680NPS | 8/30/2021 | 216796 | 216796 | 433241 | 432716 | 432716 | 1747.47 | 1 | 5647 |
| 20MU9681NPS | 8/30/2021 | 563009 | 563009 | 1125509 | 1123778 | 1123778 | 4444.40 | 1 | 24580 |
| 20MU9682NPS | 8/30/2021 | 541319 | 541319 | 1082083 | 1081138 | 1081138 | 4300.53 | 16 | 19387 |
| 20MU9683NPS | 8/30/2021 | 423461 | 423461 | 846095 | 845061 | 845061 | 3414.93 | 1 | 10016 |
| 20MU9685NPS | 8/30/2021 | 614146 | 614146 | 1227728 | 1216589 | 1216589 | 4811.74 | 19 | 29678 |
| 20MU9686NPS | 8/30/2021 | 725212 | 725212 | 1449801 | 1448692 | 1448692 | 5739.81 | 16 | 26246 |
| 20MU9687NPS | 8/30/2021 | 464986 | 464986 | 929472 | 928577 | 928577 | 3685.12 | 1 | 13248 |
| 20MU9688NPS | 8/30/2021 | 589972 | 589972 | 1179363 | 1168520 | 1168520 | 4632.93 | 7 | 23095 |
| 20MU9689NPS | 8/30/2021 | 286887 | 286887 | 573238 | 572494 | 572494 | 2316.19 | 1 | 6615 |
| 20MU9690NPS | 8/30/2021 | 435613 | 435613 | 870447 | 869259 | 869259 | 3511.33 | 2 | 9977 |
| 20MU9691NPS | 8/30/2021 | 330572 | 330572 | 660782 | 658168 | 658168 | 2609.95 | 6 | 11298 |
| 20MU9693NPS | 8/30/2021 | 192758 | 192758 | 385222 | 384687 | 384687 | 1548.96 | 1 | 4539 |
| 20MU9694NPS | 8/30/2021 | 190395 | 190395 | 380477 | 379972 | 379972 | 1540.81 | 1 | 4369 |
| 20MU9695NPS | 8/30/2021 | 654585 | 654585 | 1308434 | 1307347 | 1307347 | 5169.64 | 11 | 26139 |
| 20MU9697NPS | 8/30/2021 | 438055 | 438055 | 875638 | 874793 | 874793 | 3459.40 | 8 | 12390 |
| 20MU9698NPS | 8/30/2021 | 1303446 | 1303446 | 2605758 | 2603421 | 2603421 | 10318.66 | 2 | 55455 |
| 20MU9700NPS | 8/30/2021 | 684871 | 684871 | 1368711 | 1366972 | 1366972 | 5500.70 | 1 | 16438 |
| 20MU9703NPS | 9/2/2021 | 737281 | 737281 | 1473726 | 1460993 | 1460993 | 5772.75 | 1 | 24311 |
| 20MU9705NPS | 9/2/2021 | 526400 | 526400 | 1052260 | 1051199 | 1051199 | 4172.13 | 38 | 13350 |
| 20MU9707NPS | 9/2/2021 | 621848 | 621848 | 1243244 | 1240794 | 1240794 | 4914.96 | 1 | 22839 |
| 20MU9708NPS | 9/2/2021 | 816529 | 816529 | 1632219 | 1629039 | 1629039 | 6385.62 | 1 | 25859 |
| 20MU9709NPS | 9/3/2021 | 501546 | 501546 | 1002473 | 1001116 | 1001116 | 3970.26 | 1 | 13630 |
| 20MU9712NPS | 9/6/2021 | 284400 | 284400 | 568180 | 567399 | 567399 | 2270.04 | 1 | 7129 |
| 20MU9713NPS | 9/7/2021 | 367920 | 367920 | 735309 | 734475 | 734475 | 2935.08 | 1 | 9961 |
| 20MU9714NPS | 9/7/2021 | 294861 | 294861 | 589354 | 588631 | 588631 | 2374.13 | 20 | 6855 |
| 20MU9717NPS | 9/8/2021 | 363146 | 363146 | 725885 | 715774 | 715774 | 2843.00 | 1 | 10857 |
| 20MU9718NPS | 9/8/2021 | 321541 | 321541 | 642739 | 641911 | 641911 | 2582.52 | 8 | 8080 |
| 20MU9719NPS | 9/8/2021 | 824603 | 824603 | 1648459 | 1646302 | 1646302 | 6480.86 | 6 | 23127 |
| 20MU9721NPS | 9/9/2021 | 955883 | 955883 | 1910804 | 1907892 | 1907892 | 7522.41 | 1 | 28404 |
| 20MU9722NPS | 9/9/2021 | 560823 | 560823 | 1120920 | 1119483 | 1119483 | 4465.97 | 1 | 15443 |
| 20MU9725NPS | 9/10/2021 | 312910 | 312910 | 625317 | 624460 | 624460 | 2500.66 | 1 | 8639 |
| 20MU9726NPS | 9/10/2021 | 365979 | 365979 | 731348 | 674762 | 674762 | 2670.05 | 46 | 12943 |
| 20MU9727NPS | 9/10/2021 | 346208 | 346208 | 691991 | 673338 | 673338 | 2661.72 | 1 | 13783 |
| 20MU9728NPS | 9/10/2021 | 594590 | 594590 | 1188421 | 1187064 | 1187064 | 4699.78 | 1 | 16267 |
| 20MU9730NPS | 9/11/2021 | 644826 | 644826 | 1288853 | 1287378 | 1287378 | 5102.03 | 1 | 15348 |
| 20MU9731NPS | 9/13/2021 | 533409 | 533409 | 1066453 | 1065078 | 1065078 | 4226.79 | 1 | 15959 |
| 20MU9732NPS | 9/13/2021 | 200164 | 200164 | 399962 | 399458 | 399458 | 1607.14 | 9 | 4694 |
| 20MU9733NPS | 9/13/2021 | 127239 | 127239 | 254248 | 238839 | 238839 | 941.77 | 1 | 3319 |
| 20MU9734NPS | 9/13/2021 | 511728 | 511728 | 1022528 | 1021180 | 1021180 | 4038.29 | 1 | 15710 |
| 20MU9735NPS | 9/13/2021 | 652271 | 652271 | 1303444 | 1301842 | 1301842 | 5134.22 | 1 | 21245 |
| 20MU9736NPS | 9/13/2021 | 356098 | 356098 | 711538 | 710634 | 710634 | 2832.02 | 1 | 9306 |
| 20MU9737NPS | 9/13/2021 | 961961 | 961961 | 1922729 | 1920682 | 1920682 | 7597.58 | 2 | 29221 |
| 20MU9738NPS | 9/14/2021 | 502843 | 502843 | 1005017 | 1003603 | 1003603 | 3997.20 | 69 | 14434 |
| 20MU9739NPS | 9/14/2021 | 566631 | 566631 | 1132344 | 1130995 | 1130995 | 4479.99 | 1 | 16005 |
| 20MU9743NPS | 9/15/2021 | 663808 | 663808 | 1326742 | 1325227 | 1325227 | 5253.76 | 1 | 17609 |
| 20MU9747NPS | 9/15/2021 | 233529 | 233529 | 466699 | 444088 | 444088 | 1762.47 | 14 | 9346 |
| 20MU9749NPS | 9/15/2021 | 366382 | 366382 | 732309 | 731490 | 731490 | 2927.41 | 1 | 9742 |
| 20MU9750NPS | 9/16/2021 | 444149 | 444149 | 887784 | 865333 | 865333 | 3419.60 | 68 | 17123 |
| 20MU9751NPS | 9/16/2021 | 374677 | 374677 | 748829 | 747967 | 747967 | 2983.74 | 1 | 9984 |
| 20MU9752NPS | 9/16/2021 | 323175 | 323175 | 645827 | 645042 | 645042 | 2572.56 | 1 | 8607 |
| 20MU9754NPS | 9/17/2021 | 603991 | 603991 | 1207220 | 1205921 | 1205921 | 4779.52 | 2 | 17677 |
| 20MU9755NPS | 9/17/2021 | 509612 | 509612 | 1018429 | 1017310 | 1017310 | 4027.66 | 1 | 14535 |
| 20MU9756NPS | 9/17/2021 | 485130 | 485130 | 969596 | 968242 | 968242 | 3818.90 | 3 | 15101 |
| 20MU9758NPS | 9/17/2021 | 520714 | 520714 | 1040752 | 1039687 | 1039687 | 4133.54 | 3 | 13753 |
| 20MU9759NPS | 9/17/2021 | 398961 | 398961 | 797341 | 796379 | 796379 | 3177.26 | 1 | 10480 |
| 20MU9761NPS | 9/17/2021 | 676705 | 676705 | 1352585 | 1350306 | 1350306 | 5380.57 | 2 | 20622 |
| 20MU9762NPS | 9/17/2021 | 496955 | 496955 | 993305 | 991404 | 991404 | 3933.35 | 2 | 14549 |
| 20MU9764NPS | 9/18/2021 | 515500 | 515500 | 1030380 | 1028965 | 1028965 | 4094.60 | 43 | 14592 |
| 20MU9765NPS | 9/18/2021 | 560574 | 560574 | 1120632 | 1098318 | 1098318 | 4307.89 | 2 | 18279 |
| 20MU9766NPS | 9/19/2021 | 266697 | 266697 | 533073 | 528470 | 528470 | 2089.46 | 9 | 8963 |
| 20MU9767NPS | 9/19/2021 | 424619 | 424619 | 848661 | 847438 | 847438 | 3406.98 | 74 | 10663 |
| 20MU9768NPS | 9/19/2021 | 491278 | 491278 | 981762 | 980518 | 980518 | 3904.74 | 63 | 12232 |
| 20MU9769NPS | 9/19/2021 | 381715 | 381715 | 762866 | 761805 | 761805 | 3036.20 | 1 | 10325 |
| 20MU9772NPS | 9/20/2021 | 593535 | 593535 | 1186378 | 1181855 | 1181855 | 4635.35 | 2 | 28457 |
| 20MU9773NPS | 9/20/2021 | 595718 | 595718 | 1190724 | 1189145 | 1189145 | 4693.20 | 1 | 17810 |
| 20MU9774NPS | 9/20/2021 | 486044 | 486044 | 971425 | 970393 | 970393 | 3850.40 | 1 | 13548 |
| 20MU9775NPS | 9/21/2021 | 361273 | 361273 | 721967 | 721112 | 721112 | 2897.88 | 45 | 9191 |
| 20MU9776NPS | 9/22/2021 | 935540 | 935540 | 1870175 | 1867950 | 1867950 | 7394.64 | 1 | 33364 |
| 20MU9780NPS | 9/23/2021 | 543495 | 543495 | 1086332 | 1085108 | 1085108 | 4334.55 | 19 | 14719 |
| 20MU9781NPS | 9/23/2021 | 1049800 | 1049800 | 2098520 | 2084577 | 2084577 | 8180.96 | 2 | 49014 |
| 20MU9782NPS | 9/23/2021 | 665307 | 665307 | 1329727 | 1328075 | 1328075 | 5242.41 | 3 | 19701 |
| 20MU9785NPS | 9/24/2021 | 582408 | 582408 | 1164108 | 1162864 | 1162864 | 4609.69 | 1 | 16753 |
| 20MU9786NPS | 9/24/2021 | 351215 | 351215 | 701962 | 681539 | 681539 | 2698.23 | 55 | 12931 |
| 20MU9789NPS | 9/24/2021 | 681228 | 681228 | 1361880 | 1322094 | 1322094 | 5211.48 | 1 | 19803 |
| 20MU9794NPS | 9/25/2021 | 705805 | 705805 | 1410736 | 1407480 | 1407480 | 5554.92 | 1 | 20489 |
| 20MU9796NPS | 9/27/2021 | 608917 | 608917 | 1217041 | 1215531 | 1215531 | 4839.66 | 1 | 15132 |
| 20MU9797NPS | 9/27/2021 | 270756 | 270756 | 541187 | 540642 | 540642 | 2130.83 | 1 | 7400 |
| 20MU9798NPS | 9/27/2021 | 590259 | 590259 | 1179595 | 1177967 | 1177967 | 4647.31 | 1 | 18758 |
| 20MU9799NPS | 9/27/2021 | 174093 | 174093 | 347993 | 328818 | 328818 | 1309.80 | 1 | 7764 |
| 20MU9800NPS | 9/27/2021 | 262206 | 262206 | 524095 | 523393 | 523393 | 2053.20 | 13 | 7161 |
| 20MU9801NPS | 9/27/2021 | 164648 | 164648 | 328931 | 328502 | 328502 | 1300.97 | 17 | 4334 |
| 20MU9803NPS | 9/28/2021 | 182440 | 182440 | 364458 | 363958 | 363958 | 1440.96 | 17 | 5125 |
| 20MU9804NPS | 9/28/2021 | 106597 | 106597 | 213006 | 212630 | 212630 | 840.79 | 1 | 3124 |
| 20MU9805NPS | 9/28/2021 | 198853 | 198853 | 397345 | 396771 | 396771 | 1597.94 | 1 | 5002 |
| 20MU9806NPS | 9/28/2021 | 203762 | 203762 | 407228 | 406618 | 406618 | 1616.00 | 19 | 5324 |
| 20MU9807NPS | 9/28/2021 | 159788 | 159788 | 319339 | 318889 | 318889 | 1270.13 | 15 | 3978 |
| 20MU9808NPS | 9/28/2021 | 176696 | 176696 | 353170 | 335486 | 335486 | 1325.60 | 5 | 7001 |
| 20MU9809NPS | 9/28/2021 | 204750 | 204750 | 409218 | 408355 | 408355 | 1613.78 | 1 | 5809 |
| 20MU9810NPS | 9/29/2021 | 271611 | 271611 | 542749 | 493890 | 493890 | 1943.42 | 1 | 9304 |
| 20MU9811NPS | 9/29/2021 | 160664 | 160664 | 321038 | 320524 | 320524 | 1267.70 | 11 | 4882 |
| 20MU9813NPS | 9/29/2021 | 122466 | 122466 | 244688 | 244346 | 244346 | 974.75 | 12 | 3141 |
| 20MU9814NPS | 9/29/2021 | 157011 | 157011 | 313723 | 313316 | 313316 | 1246.97 | 1 | 3801 |
| 20MU9818NPS | 9/29/2021 | 229887 | 229887 | 459543 | 458994 | 458994 | 1782.43 | 5 | 6079 |
| 20MU9820NPS | 9/29/2021 | 189203 | 189203 | 378065 | 377584 | 377584 | 1499.40 | 16 | 4998 |
| 20MU9822NPS | 9/29/2021 | 218529 | 218529 | 436589 | 435991 | 435991 | 1727.18 | 2 | 5141 |
| 20MU9824NPS | 9/29/2021 | 200884 | 200884 | 401399 | 400830 | 400830 | 1592.07 | 1 | 4954 |
| 20MU9826NPS | 9/29/2021 | 218696 | 218696 | 437074 | 436384 | 436384 | 1721.37 | 1 | 6040 |
| 20MU9828NPS | 9/29/2021 | 298820 | 298820 | 597333 | 581503 | 581503 | 2289.59 | 1 | 9057 |
| 20MU9829NPS | 9/29/2021 | 221235 | 221235 | 442173 | 441593 | 441593 | 1750.82 | 1 | 5616 |
| 20MU9831NPS | 9/29/2021 | 213647 | 213647 | 427015 | 426083 | 426083 | 1683.30 | 4 | 6087 |
| 20MU9833NPS | 9/29/2021 | 369699 | 369699 | 738888 | 687767 | 687767 | 2714.09 | 1 | 10447 |
| 20MU9834NPS | 10/4/2021 | 154937 | 154937 | 309640 | 309159 | 309159 | 1217.98 | 1 | 4261 |
| 20MU9839NPS | 10/4/2021 | 320642 | 320642 | 640878 | 625589 | 625589 | 2458.29 | 8 | 11280 |
| 20MU9840NPS | 10/4/2021 | 221443 | 221443 | 442602 | 441974 | 441974 | 1743.00 | 11 | 6590 |
| 20MU9841NPS | 10/4/2021 | 171915 | 171915 | 343093 | 342084 | 342084 | 1350.86 | 1 | 4969 |
| 20MU9843NPS | 10/5/2021 | 165196 | 165196 | 330089 | 329608 | 329608 | 1309.13 | 1 | 4370 |
| 20MU9844NPS | 10/6/2021 | 121272 | 121272 | 242329 | 241948 | 241948 | 960.08 | 5 | 2810 |
| 20MU9846NPS | 10/6/2021 | 146801 | 146801 | 293377 | 292969 | 292969 | 1159.24 | 13 | 3858 |
| 20MU9848NPS | 10/6/2021 | 195340 | 195340 | 390299 | 389809 | 389809 | 1558.69 | 17 | 4810 |
| 20MU9849NPS | 10/6/2021 | 173238 | 173238 | 345939 | 303352 | 303352 | 1194.50 | 4 | 6431 |
| 20MU9850NPS | 10/6/2021 | 135843 | 135843 | 271527 | 250152 | 250152 | 978.45 | 1 | 5125 |
| 20MU9851NPS | 10/7/2021 | 144681 | 144681 | 289098 | 288738 | 288738 | 1145.07 | 1 | 3785 |
| 20MU9852NPS | 10/7/2021 | 146619 | 146619 | 293000 | 292632 | 292632 | 1161.97 | 4 | 3115 |
| 20MU9853NPS | 10/7/2021 | 209349 | 209349 | 418346 | 417450 | 417450 | 1642.44 | 20 | 8603 |
| 20MU9854NPS | 10/7/2021 | 135620 | 135620 | 270994 | 270646 | 270646 | 1062.39 | 1 | 4236 |
| 20MU9855NPS | 10/7/2021 | 130894 | 130894 | 261561 | 260695 | 260695 | 1029.47 | 1 | 4658 |
| 20MU9856NPS | 10/7/2021 | 119520 | 119520 | 238821 | 238514 | 238514 | 950.57 | 4 | 2816 |
| 20MU9857NPS | 10/7/2021 | 152314 | 152314 | 304340 | 303853 | 303853 | 1197.61 | 1 | 3511 |
| 20MU9858NPS | 10/8/2021 | 212673 | 212673 | 424861 | 398627 | 398627 | 1580.15 | 17 | 6704 |
| 20MU9861NPS | 10/8/2021 | 197460 | 197460 | 394581 | 344430 | 344430 | 1357.12 | 5 | 6151 |
| 20MU9862NPS | 10/8/2021 | 216378 | 216378 | 432418 | 431867 | 431867 | 1710.23 | 2 | 5385 |
| 20MU9863NPS | 10/8/2021 | 265326 | 265326 | 530200 | 529520 | 529520 | 2092.59 | 24 | 7626 |
| 20MU9864NPS | 10/9/2021 | 233539 | 233539 | 466797 | 464043 | 464043 | 1830.02 | 1 | 6979 |
| 20MU9867NPS | 10/11/2021 | 112296 | 112296 | 224409 | 224111 | 224111 | 889.47 | 5 | 2890 |
| 20MU9868NPS | 10/11/2021 | 128793 | 128793 | 257373 | 257043 | 257043 | 1021.17 | 1 | 3354 |
| 20MU9869NPS | 10/11/2021 | 3608114 | 3610936 | 7193025 | 6209046 | 6209046 | 23702.14 | 1 | 49005 |
| 20MU9871NPS | 10/11/2021 | 279284 | 279284 | 558135 | 555564 | 555564 | 2184.03 | 23 | 11143 |
| 20MU9872NPS | 10/11/2021 | 1925904 | 1926213 | 3842396 | 3415461 | 3415461 | 12584.94 | 1 | 29779 |
| 20MU9874NPS | 10/13/2021 | 139439 | 139439 | 278702 | 275211 | 275211 | 1088.30 | 1 | 6036 |
| 20MU9876NPS | 10/13/2021 | 218267 | 218267 | 436241 | 400057 | 400057 | 1575.82 | 7 | 5994 |
| 20MU9879NPS | 10/13/2021 | 191908 | 191908 | 383299 | 382632 | 382632 | 1517.29 | 32 | 4846 |
| 20MU9880NPS | 10/13/2021 | 115124 | 115124 | 230055 | 229595 | 229595 | 906.64 | 12 | 3565 |
| 20MU9881NPS | 10/14/2021 | 196090 | 196090 | 391720 | 391192 | 391192 | 1550.64 | 7 | 5405 |
| 20MU9882NPS | 10/14/2021 | 127208 | 127208 | 254156 | 253759 | 253759 | 1006.64 | 1 | 3414 |
| 20MU9883NPS | 10/14/2021 | 227084 | 227084 | 453889 | 423328 | 423328 | 1665.98 | 10 | 8099 |
| 20MU9884NPS | 10/14/2021 | 211240 | 211240 | 422073 | 421380 | 421380 | 1670.27 | 22 | 6763 |
| 20MU9885NPS | 10/14/2021 | 314885 | 314885 | 629365 | 586448 | 586448 | 2299.61 | 1 | 9658 |
| 20MU9888NPS | 10/15/2021 | 2540829 | 2541419 | 5069514 | 4810310 | 4810310 | 18030.85 | 2 | 51550 |
| 20MU9890NPS | 10/16/2021 | 234182 | 234182 | 468101 | 467491 | 467491 | 1844.76 | 1 | 6553 |
| 20MU9892NPS | 10/18/2021 | 1026526 | 1026800 | 2046540 | 1906561 | 1906561 | 7552.02 | 1 | 14980 |
| 20MU9893NPS | 10/18/2021 | 463161 | 463161 | 925900 | 911823 | 911823 | 3606.07 | 17 | 18988 |
| 20MU9894NPS | 10/18/2021 | 748115 | 748115 | 1495484 | 1493427 | 1493427 | 5888.38 | 1 | 22433 |
| 20MU9895NPS | 10/18/2021 | 481307 | 481307 | 961816 | 924696 | 924696 | 3716.79 | 1 | 15918 |
| 20MU9896NPS | 10/19/2021 | 366679 | 366679 | 732756 | 731650 | 731650 | 2922.80 | 3 | 8682 |
| 20MU9897NPS | 10/20/2021 | 4641148 | 4642094 | 9270231 | 8415515 | 8415515 | 31787.77 | 2 | 102403 |
| 20MU9898NPS | 10/20/2021 | 520399 | 520399 | 1040289 | 1031754 | 1031754 | 4109.55 | 1 | 18644 |
| 20MU9900NPS | 10/20/2021 | 203411 | 203411 | 406621 | 406144 | 406144 | 1601.73 | 1 | 9633 |
| 20MU9902NPS | 10/20/2021 | 3830780 | 3830592 | 7655022 | 7120829 | 7120829 | 26831.16 | 3 | 49117 |
| 20MU9903NPS | 10/21/2021 | 245956 | 245956 | 491571 | 490938 | 490938 | 1959.68 | 2 | 5995 |
| 20MU9906NPS | 10/21/2021 | 641229 | 641229 | 1281928 | 1275612 | 1275612 | 4998.88 | 2 | 37566 |
| 20MU9907NPS | 10/22/2021 | 210907 | 210907 | 421561 | 421059 | 421059 | 1673.95 | 14 | 5705 |
| 20MU9910NPS | 10/22/2021 | 397047 | 397047 | 793738 | 792557 | 792557 | 3163.26 | 1 | 11050 |
| 20MU9911NPS | 10/22/2021 | 211561 | 211561 | 422643 | 422099 | 422099 | 1691.00 | 1 | 5945 |
| 20MU9913NPS | 10/24/2021 | 738921 | 738921 | 1477090 | 1475301 | 1475301 | 5851.08 | 1 | 19494 |
| 20MU9915NPS | 10/25/2021 | 500608 | 500608 | 1000519 | 938436 | 938436 | 3690.82 | 1 | 17148 |
| 20MU9916NPS | 10/26/2021 | 218867 | 218867 | 437424 | 436783 | 436783 | 1733.01 | 5 | 5689 |
| 20MU9917NPS | 10/26/2021 | 722645 | 722645 | 1444554 | 1442662 | 1442662 | 5690.07 | 35 | 21480 |
| 20MU9918NPS | 10/21/2021 | 433039 | 433039 | 865586 | 846459 | 846459 | 3340.64 | 3 | 12852 |
| 20MU9919NPS | 10/26/2021 | 339383 | 339383 | 678255 | 677261 | 677261 | 2705.19 | 16 | 7943 |
| 20MU9920NPS | 10/27/2021 | 560056 | 560056 | 1119561 | 1118113 | 1118113 | 4474.51 | 1 | 19007 |
| 20MU9923NPS | 10/28/2021 | 394598 | 394598 | 788796 | 781174 | 781174 | 3082.41 | 1 | 15349 |
| 20MU9924NPS | 10/28/2021 | 639177 | 639177 | 1277953 | 1275195 | 1275195 | 5122.15 | 1 | 23600 |
| 20MU9928NPS | 10/29/2021 | 232352 | 232352 | 464346 | 463697 | 463697 | 1843.70 | 1 | 5799 |
| 20MU9929NPS | 10/29/2021 | 598373 | 598373 | 1196061 | 1194545 | 1194545 | 4711.77 | 1 | 17798 |
| 20MU9931NPS | 10/30/2021 | 303358 | 303358 | 606350 | 605502 | 605502 | 2399.36 | 21 | 8630 |
| 20MU9933NPS | 10/30/2021 | 282059 | 282059 | 563647 | 492189 | 492189 | 1942.32 | 1 | 9623 |
| 20MU9934NPS | 10/30/2021 | 293535 | 293535 | 586727 | 585860 | 585860 | 2293.45 | 1 | 13046 |
| 20MU9936NPS | 10/31/2021 | 319050 | 319050 | 637688 | 611126 | 611126 | 2415.31 | 22 | 8300 |
| 20MU9937NPS | 10/31/2021 | 1420757 | 1420757 | 2840120 | 2834921 | 2834921 | 11219.75 | 1 | 43726 |
| 20MU9938NPS | 10/31/2021 | 407531 | 407531 | 814436 | 813350 | 813350 | 3234.61 | 1 | 9997 |
| 20MU9939NPS | 10/31/2021 | 1277176 | 1277176 | 2553192 | 2540946 | 2540946 | 10136.45 | 1 | 50094 |
| 20MU9940NPS | 10/31/2021 | 1684245 | 1684245 | 3367122 | 3348277 | 3348277 | 13164.54 | 1 | 76450 |
| 20MU9942NPS | 11/1/2021 | 1153516 | 1153516 | 2304701 | 2299228 | 2299228 | 9154.23 | 1 | 33148 |
| 20MU9944NPS | 11/3/2021 | 672196 | 672196 | 1342989 | 1339756 | 1339756 | 5337.48 | 1 | 15886 |
| 20MU9945NPS | 11/3/2021 | 370995 | 370995 | 741235 | 738059 | 738059 | 2965.09 | 1 | 8932 |
| 20MU9947NPS | 11/3/2021 | 83502 | 83502 | 166547 | 83497 | 83497 | 322.91 | 1 | 2091 |
| 20MU9949NPS | 11/4/2021 | 630505 | 630505 | 1259578 | 1228815 | 1228815 | 4891.46 | 3 | 18419 |
| 20MU9952NPS | 11/6/2021 | 480267 | 480267 | 959272 | 892105 | 892105 | 3537.62 | 5 | 16497 |
| 20MU9954NPS | 11/7/2021 | 715329 | 715329 | 1429218 | 1374870 | 1374870 | 5469.16 | 2 | 17699 |
| 20MU9956NPS | 11/8/2021 | 516657 | 516657 | 1032796 | 1004377 | 1004377 | 3975.84 | 1 | 22887 |
| 20MU9957NPS | 11/8/2021 | 76239 | 76239 | 152111 | 103642 | 103642 | 404.98 | 1 | 5667 |
| 20MU9958NPS | 11/8/2021 | 707985 | 707985 | 1414530 | 1404314 | 1404314 | 5571.87 | 1 | 16522 |
| 20MU9961NPS | 11/9/2021 | 71635 | 71635 | 142529 | 123139 | 123139 | 483.63 | 2 | 3242 |
| 20MU9962NPS | 11/9/2021 | 926086 | 926086 | 1849978 | 1848046 | 1848046 | 7386.48 | 1 | 21395 |
| 20MU9963NPS | 11/9/2021 | 805831 | 805831 | 1609707 | 1593995 | 1593995 | 6358.03 | 86 | 20543 |
| 20MU9968NPS | 11/11/2021 | 541258 | 541258 | 1080682 | 652990 | 652990 | 2551.71 | 2 | 9921 |
| 20MU9970NPS | 11/11/2021 | 633669 | 633669 | 1265972 | 1242571 | 1242571 | 4955.20 | 1 | 13492 |
| 20MU9971NPS | 11/11/2021 | 916198 | 916198 | 1830483 | 1828410 | 1828410 | 7305.75 | 1 | 19779 |
| 20MU9972NPS | 11/11/2021 | 407320 | 407320 | 813359 | 663126 | 663126 | 2601.52 | 8 | 14250 |
| 20MU9973NPS | 11/11/2021 | 717908 | 717908 | 1434041 | 1275356 | 1275356 | 5011.64 | 1 | 13231 |
| 20MU9982NPS | 11/14/2021 | 1394817 | 1394817 | 2788448 | 2764770 | 2764770 | 10981.96 | 6 | 55830 |
| 20MU9999NPS | 11/17/2021 | 4811475 | 4811475 | 9610386 | 6643561 | 6643561 | 4963.06 | 0 | 237908 |

**Supplementary Table 2. Lineages detected among study samples**

| **Pango Lineage** | **Count** | **First detection** | **Last detection** |
| --- | --- | --- | --- |
| B.1.2 | 373 | 7/5/2020 | 10/1/2021 |
| AY.103 | 283 | 5/23/2021 | 1/27/2022 |
| AY.3 | 219 | 5/18/2021 | 1/29/2022 |
| BA.1.1 | 186 | 9/28/2020 | 4/9/2022 |
| B.1.234 | 118 | 8/20/2020 | 1/28/2021 |
| B.1.1 | 99 | 7/1/2020 | 1/26/2021 |
| B.1 | 92 | 7/1/2020 | 11/28/2021 |
| AY.44 | 59 | 6/18/2021 | 1/27/2022 |
| BA.1.15 | 51 | 12/22/2021 | 2/27/2022 |
| BA.1 | 50 | 12/17/2021 | 2/17/2022 |
| B.1.1.7 | 46 | 10/5/2020 | 7/7/2021 |
| B.1.240 | 45 | 7/2/2020 | 1/26/2021 |
| BA.2.12.1 | 39 | 4/6/2022 | 7/31/2022 |
| B.1.617.2 | 37 | 6/12/2021 | 12/23/2021 |
| BA.5.5 | 31 | 4/27/2022 | 7/31/2022 |
| B.1.311 | 26 | 7/8/2020 | 4/5/2021 |
| AY.25 | 25 | 7/26/2021 | 12/20/2021 |
| BA.1.20 | 25 | 1/28/2021 | 3/2/2022 |
| B.1.509 | 24 | 7/7/2020 | 1/29/2021 |
| BA.4.6 | 21 | 6/12/2022 | 7/31/2022 |
| BA.2.3 | 19 | 2/27/2022 | 6/2/2022 |
| AY.100 | 17 | 7/12/2021 | 12/30/2021 |
| AY.39 | 16 | 7/21/2021 | 12/22/2021 |
| BA.5.2.1 | 16 | 6/15/2022 | 7/31/2022 |
| BA.2 | 13 | 12/5/2020 | 7/31/2022 |
| BA.4.1 | 12 | 6/18/2022 | 7/31/2022 |
| B.1.324 | 11 | 8/12/2020 | 10/31/2020 |
| B.1.577 | 9 | 8/28/2020 | 12/11/2020 |
| B.1.1.519 | 7 | 10/19/2020 | 3/31/2021 |
| BA.5.1 | 6 | 6/22/2022 | 7/17/2022 |
| AY.75 | 5 | 8/29/2021 | 9/16/2021 |
| B.1.1.135 | 5 | 7/1/2020 | 10/10/2020 |
| B.1.1.316 | 5 | 1/18/2021 | 1/27/2021 |
| B.1.243 | 5 | 8/28/2020 | 1/18/2021 |
| B.1.377 | 5 | 10/16/2020 | 1/19/2021 |
| B.1.400 | 5 | 9/9/2020 | 12/9/2020 |
| BA.4 | 5 | 6/8/2022 | 7/11/2022 |
| BA.5.2 | 5 | 7/25/2021 | 7/29/2022 |
| AY.4 | 4 | 11/17/2020 | 8/23/2021 |
| B.1.1.33 | 4 | 10/9/2020 | 10/17/2020 |
| B.1.544 | 4 | 11/3/2020 | 12/1/2020 |
| B.1.565 | 4 | 10/28/2020 | 12/11/2020 |
| BA.1.17 | 4 | 12/27/2021 | 2/6/2022 |
| BA.5.6 | 4 | 6/28/2022 | 7/25/2022 |
| AY.118 | 3 | 7/21/2021 | 10/27/2021 |
| AY.119 | 3 | 10/26/2021 | 2/10/2022 |
| AY.26 | 3 | 7/28/2021 | 9/13/2021 |
| AY.3.1 | 3 | 11/23/2021 | 12/12/2021 |
| B.1.1.416 | 3 | 1/26/2021 | 1/27/2021 |
| B.1.609 | 3 | 12/10/2020 | 1/8/2021 |
| BA.5.1.1 | 3 | 7/4/2022 | 7/30/2022 |
| BE.3 | 3 | 6/27/2022 | 7/1/2022 |
| AY.119.2 | 2 | 8/16/2021 | 9/24/2021 |
| AY.122 | 2 | 8/23/2021 | 11/28/2021 |
| AY.20 | 2 | 9/7/2021 | 10/22/2021 |
| AY.46.4 | 2 | 8/30/2021 | 1/30/2022 |
| AY.47 | 2 | 7/19/2021 | 10/13/2021 |
| AY.49 | 2 | 8/16/2021 | 8/16/2021 |
| B.1.1.432 | 2 | 9/7/2020 | 9/9/2020 |
| B.1.1.464 | 2 | 9/6/2020 | 11/7/2020 |
| B.1.110.3 | 2 | 10/15/2020 | 12/7/2020 |
| B.1.265 | 2 | 10/23/2020 | 10/27/2020 |
| B.1.349 | 2 | 10/2/2020 | 1/26/2021 |
| B.1.568 | 2 | 12/12/2020 | 1/30/2021 |
| B.1.623 | 2 | 4/15/2021 | 4/28/2021 |
| BA.1.1.18 | 2 | 1/15/2022 | 1/15/2022 |
| BA.1.17.2 | 2 | 1/25/2022 | 1/25/2022 |
| BA.5 | 2 | 7/5/2022 | 7/22/2022 |
| BF.24 | 2 | 7/26/2022 | 7/26/2022 |
| C.23 | 2 | 10/10/2020 | 10/19/2020 |
| AY.14 | 1 | 7/28/2021 | 7/28/2021 |
| AY.25.1 | 1 | 12/9/2021 | 12/9/2021 |
| AY.25.1.1 | 1 | 11/29/2021 | 11/29/2021 |
| AY.39.1 | 1 | 12/20/2021 | 12/20/2021 |
| AY.62 | 1 | 7/9/2021 | 7/9/2021 |
| B.1.1.186 | 1 | 12/5/2021 | 12/5/2021 |
| B.1.1.291 | 1 | 7/9/2020 | 7/9/2020 |
| B.1.1.529 | 1 | 6/7/2022 | 6/7/2022 |
| B.1.206 | 1 | 7/7/2020 | 7/7/2020 |
| B.1.241 | 1 | 1/5/2021 | 1/5/2021 |
| B.1.361 | 1 | 11/7/2020 | 11/7/2020 |
| B.1.396 | 1 | 12/10/2020 | 12/10/2020 |
| B.1.416 | 1 | 1/19/2021 | 1/19/2021 |
| B.1.429 | 1 | 8/13/2020 | 8/13/2020 |
| BA.1.1.10 | 1 | 2/19/2022 | 2/19/2022 |
| BA.1.18 | 1 | 12/27/2021 | 12/27/2021 |
| BA.2.20 | 1 | 4/1/2022 | 4/1/2022 |
| BA.2.65 | 1 | 4/22/2022 | 4/22/2022 |
| BA.2.9 | 1 | 4/13/2022 | 4/13/2022 |
| BA.4.2 | 1 | 6/9/2022 | 6/9/2022 |
| BA.4.4 | 1 | 7/18/2022 | 7/18/2022 |
| BA.4.6.5 | 1 | 7/28/2022 | 7/28/2022 |
| BA.5.1.25 | 1 | 6/30/2022 | 6/30/2022 |
| BA.5.2.21 | 1 | 7/28/2022 | 7/28/2022 |
| BF.1 | 1 | 7/27/2022 | 7/27/2022 |
| BF.10 | 1 | 6/13/2022 | 6/13/2022 |
| BF.9 | 1 | 7/21/2022 | 7/21/2022 |
| BG.5 | 1 | 6/15/2022 | 6/15/2022 |
| XAC | 1 | 4/25/2022 | 4/25/2022 |
| XAP | 1 | 4/8/2022 | 4/8/2022 |

**Supplementary Table 3. Host and virus features**

| **Features** | **Description** | **Feature Type** | **Imputation Method*** |
| --- | --- | --- | --- |
| **Identifier (not analyzed)** |  |  |  |
| BarCode | Identifier |  |  |
| swab | Date of swab collection |  |  |
| **Host characteristics** |  |  |  |
| age | Age at time of swab collection | Binned by: 0-17 years, 18-49 years, 50-64 years, ≥65 years. Label encoding as 0-17 years = 0, 18-49 years = 1, 50-64 years = 2, >65 years = 3. | None |
| sex | Sex | 0 = Female, 1 = Male | logistic regression |
| race | Race: "AMERICAN INDIAN OR ALASKAN NATIVE", "ASIAN", "BLACK OR AFRICAN AMERICAN", "NATIVE HAWAIIAN OR OTHER PACIFIC ISLAND", "SOME OTHER RACE", "WHITE" | One-hot encoding: Each level encoded separately as yes = 1, no = 0 | polytomous regression |
| ethnicity | Ethnicity: "Hispanic, Latino, or Spanish Origin" = 1, "Not Hispanic, Latino, or Spanish Origin" = 0 | yes = 1, no = 0 | logistic regression |
| lassification | Urban-rural classification: "Isolated", "Large rural", "Small rural", "Urban" as defined by RUCA2 codes and University of Washington Rural Health Research Center https://depts.washington.edu/uwruca/ruca-uses.php | Label encoding as: Isolated = 0, Small rural = 1, Large rural = 2, Urban = 3. | polytomous regression |
| flu vaccinated | None, Vaccinated at least 2 weeks prior to swab date and within 2 years of swab date | yes = 1, no = 0 | logistic regression |
| covid vaccinated | None, Partial (1 COVID-19 vaccine), Full (2 COVID-19 vaccines or 1 Jansen), Boosted (3+ COVID-19 vaccines, or 1 Jansen and 2+ COVID-19 vaccines) at least 2 weeks prior to swab date | Label encoding as: None = 0, Partial = 1, Full = 2, Boosted = 3 | polytomous regression |
| pregnant | Pregnant during COVID-19 positive test | yes = 1, no = 0 | None |
| immunosuppression | Defined as an individual with a diagnosis of HIV/AIDS, Immunodeficiency, chronic steroid use (>= 1 month), transplant, primary, secondary, or combined immunodeficiency, or individuals taking immunosuppressants or have a CD4 count of < 500. | yes = 1, no = 0 | logistic regression |
| metabolic syndrome comorbidities | Metabolic syndrome-associated factors: obesity, Hypertension, Diabetes, hyperlipidemia | Numeric (Number of metabolic syndrome associated factors listed under description between 0 to 4) | polytomous regression |
| cardiovascular disease | Cardiovascular disease factors: heart failure, atherosclerosis/coronary artery disease, cardiomyopathies, atrial fibrillation/flutter, ischemic heart disease, chronic kidney disease | yes = 1, no = 0 | logistic regression |
| liver disease | Viral hepatitis and liver disease | yes = 1, no = 0 | logistic regression |
| chronic lung disease | Chronic Lung Disease | yes = 1, no = 0 | logistic regression |
| mental health disorder | Mental Health Disorder | yes = 1, no = 0 | logistic regression |
| ventilation | Ventilation required during hospital admission | yes = 1, no = 0 | None |
| sepsis | Sepsis during hospital admission | yes = 1, no = 0 | None |
| cpr | Required cardiopulmonary resuscitation during hospital admission | yes = 1, no = 0 | None |
| ards | Experienced acute respiratory distress syndrome during hospital admission | yes = 1, no = 0 | None |
| co-viral infection | Co-viral infections during hospital admission | yes = 1, no = 0 | logistic regression |
| co-bacterial infection | Co-bacterial infections during hospital admission | yes = 1, no = 0 | logistic regression |
| sxs 1 | Respiratory Symptoms | yes = 1, no = 0 | logistic regression |
| sxs 2 | Gastrointestinal Symptoms | yes = 1, no = 0 | logistic regression |
| sxs 3 | Constitutional Symptoms | yes = 1, no = 0 | logistic regression |
| sxs 4 | Cardiac Symptoms | yes = 1, no = 0 | logistic regression |
| **Virus characteristics** |  |  |  |
| ct | Cycle threshold value | Label encoding: Binned by high (Ct ≤ 25) = 2, moderate (26 ≤ Ct ≤ 30) = 1, and low (Ct > 30) = 0. | Impute with average of each lineage. The average CT-value of the WHO variant was used if Pango Lineage was not available. |
| amino acid substitutions | Amino acid substitutions appearing on the consensus sequence compared to Wuhan-Hu-1 (NCBI Accession: 045512.2) | Each substitution encoded separately as yes = 1, no = 0 | Sequences containing missing or ambiguous bases are encoded with 0.5. |
| **Outcomes** |  |  |  |
| Hospitalized | Admitted to the hospital | yes = 1, no = 0 | None |
| ICUs | Admitted to the intensive care unit (ICU) | yes = 1, no = 0 | None |
| Long COVID | Individual continued to experience COVID-19 symptoms 4-8 weeks after swab date | yes = 1, no = 0 | Individuals without a follow up clinical visit are also encoded with 0. |
| *Imputation using Multivariate Imputation by Chained Equations (MICE) logistic regression, predictive mean matching, or polytomous regression unless otherwise stated | | | |

**Supplementary Table 4. Evaluated machine learning algorithms and hyperparameters**

| **Algorithm** | **Scikit-learn** | **Hyperparameter Grid** |
| --- | --- | --- |
| Random Forest | RandomForestClassifier() | model__n_estimators: [10, 100, 200, 300, 500, 700, 1000], |
|  |  | model__max_depth: [None, 3, 5, 10, 20, 30, 50, 100], |
|  |  | model__max_features: [0.1, 0.25, 0.5], |
|  |  | model__criterion: [gini, entropy] |
| Gradient Boosting | GradientBoostingClassifier() | model__n_estimators: [50, 100, 150, 500, 700], |
|  |  | model__learning_rate: [0.01, 0.1, 0.2], |
|  |  | model__max_depth: [2, 3, 4, 5] |
|  |  | max_features=log2 |
| Support Vector Classification | SVC() | model__C: [0.001, 0.01, 0.1, 1], |
|  |  | model__kernel: [linear, rbf, poly], |
|  |  | model__degree: [1, 3, 5] |
|  |  | probability=True |
|  |  | gamma=0.1 |
|  |  | coef0=10.0 |
| Extra Trees | ExtraTreesClassifier() | criterion=entropy |
|  |  | max_features="log2" |
|  |  | model__n_estimators: [100, 200, 300, 1000, 1500], |
|  |  | model__max_depth: [None, 10, 20, 30] |
| Logistic Regression | LogisticRegression() | max_iter=10000 |
|  |  | model__C: [0.1, 1, 1.5, 2, 5, 10, 20], |
|  |  | model__penalty: [l1, l2], |
|  |  | model__solver: [liblinear, saga] |
| Neural Network: Multilayer perceptron (MLP) | MLPClassifier | max_iter=10000 |
|  |  | model__solver: [adam], |
|  |  | model__learning_rate: [constant, invscaling, adaptive], |
|  |  | model__hidden_layer_sizes: [(10, 7, 3), (30, 20, 12), (50, 35, 25), (70, 50, 35)], |
|  |  | model__alpha: [0.0001, 0.001], |
|  |  | model__activation: [identity, logistic, tanh, relu] |
| Naïve Bayes | GaussianNB() |  |
| Decision Tree | DecisionTreeClassifier() | model__max_depth: [None, 1, 2, 10, 20, 30], |
|  |  | model__min_samples_split: [2, 5, 10], |
|  |  | model__splitter: [random, best], |
|  |  | model__min_samples_leaf: [1, 2, 10] |
| K Nearest Neighbors | KNeighborsClassifier() | model__n_neighbors: [3, 5, 7], |
|  |  | model__weights: [uniform, distance], |

**Supplementary Table 5. Model performance for hospitalization prediction**

Available in the attached spreadsheet.

**Supplementary Table 6. Model performance for ICU prediction**

Available in the attached spreadsheet.

**Supplementary Table 7. Model performance for long COVID prediction**

Available in the attached spreadsheet.

**References**

1. Data from: COVID-19 Hospital Data from the National Hospital Care Survey.

2. Data from: COVID-19 Hospitalization Metrics, by Day. *Weekly COVID-19 Activity Report: Data for Download*.
